# Signatures of host–pathogen evolutionary conflict reveal MISTR—A conserved MItochondrial STress Response network

**DOI:** 10.1371/journal.pbio.3001045

**Published:** 2020-12-28

**Authors:** Mahsa Sorouri, Tyron Chang, Palmy Jesudhasan, Chelsea Pinkham, Nels C. Elde, Dustin C. Hancks

**Affiliations:** 1 Department of Immunology, University of Texas Southwestern Medical Center, Dallas, Texas, United States of America; 2 Institute of Biomedical Studies, Baylor University, Waco, Texas, United States of America; 3 Genetics, Development, and Disease PhD Program, University of Texas Southwestern Medical Center, Dallas, Texas, United States of America; 4 Eccles Institute of Human Genetics, The University of Utah Medical School, Utah, United States of America; New York University, UNITED STATES

## Abstract

Host–pathogen conflicts leave genetic signatures in genes that are critical for host defense functions. Using these “molecular scars” as a guide to discover gene functions, we discovered a vertebrate-specific MItochondrial STress Response (MISTR) circuit. MISTR proteins are associated with electron transport chain (ETC) factors and activated by stress signals such as interferon gamma (IFNγ) and hypoxia. Upon stress, ultraconserved microRNAs (miRNAs) down-regulate MISTR1(NDUFA4) followed by replacement with paralogs MItochondrial STress Response AntiViral (MISTRAV) and/or MItochondrial STress Response Hypoxia (MISTRH). While cells lacking MISTR1(NDUFA4) are more sensitive to chemical and viral apoptotic triggers, cells lacking MISTRAV or expressing the squirrelpox virus-encoded vMISTRAV exhibit resistance to the same insults. Rapid evolution signatures across primate genomes for *MISTR1(NDUFA4)* and *MISTRAV* indicate recent and ongoing conflicts with pathogens. MISTR homologs are also found in plants, yeasts, a fish virus, and an algal virus indicating ancient origins and suggesting diverse means of altering mitochondrial function under stress. The discovery of MISTR circuitry highlights the use of evolution-guided studies to reveal fundamental biological processes.

## Abstract 2

Host-pathogen conflicts leave genetic signatures in genes that are critical for host defense functions. This study uses these “molecular scars” as a guide to identify a vertebrate-specific mitochondrial stress response circuit that interacts with the electron transport chain and is activated by stress signals such as interferon-gamma and hypoxia.

## Introduction

Innate immunity is a critical frontline host defense mechanism in response to pathogen infection. At the onset of infections in vertebrates, a set of more than 400 genes is transcriptionally up-regulated by interferon and thus termed interferon-stimulated genes (ISGs). ISGs display diverse, key host defense functions such as activation of cell death programs and recruitment of immune cells (e.g., dendritic cells) [1,2]. Although the identities of many of these genes are established, the functions of the majority of these gene products (as well as their relationship with other cellular factors) are unknown [3,4]. To guide the characterization of poorly characterized ISGs, we used rare signatures associated with pivotal host defense factors, such as positive selection and viral-encoded homolog, to identify gene products with essential roles in immune responses [5,6]. Our rationale for the use of specific signatures as a guide for discovery stems from genes like the interferon-inducible double-stranded RNA sensor oligoadenylate synthetase 1 (OAS1) which (1) displays signatures of rapid evolution [7,8]—a hallmark of repeated conflicts with pathogens—and (2) is encoded by a virus [9].

Viruses can encode proteins that mimic host proteins to manipulate cellular functions and inactivate immune defenses. This form of mimicry is commonly achieved by the acquisition of a host-coding sequence through horizontal gene transfer (HGT) followed by subfunctionalization via cycles of mutation and selection [10]. Importantly, many viral mimics can be identified based on residual sequence identity [11]. Along with inhibitors of immune function, mimics of cellular master regulators have been identified in virus genomes (e.g., *vSRC*, *vMYC*, and *vRAS*) [reviewed in [12]]. Our study was motivated by the identification of a viral ortholog encoded by the large double-stranded DNA (dsDNA) virus squirrelpox for the ORFan ISG, *C15ORF48* [also known as *normal mucosal esophageal-specific gene product 1* (*NMES1*) [13], mouse *AA467197*]. Our experiments indicate that C15ORF48 and related proteins (1) are regulated by stress signals; (2) localize to mitochondria; and (3) are important in the fundamental host defense response of apoptosis. Hereafter, C15ORF48 is referred to as *MI**tochondrial* *ST**ress* *R**esponse* *A**nti**V**iral* (*MISTRAV*) and the viral homolog as *v**iral MISTRAV* (*SQPV078/vMISTRAV*).

Our characterization of cellular MISTRAV function unexpectedly revealed a stress-response circuit involving its paralogs MISTR1 [also known as NADH dehydrogenase ubiquinone 1 alpha subcomplex subunit 4 (NDUFA4)] and MItochondrial STress Response Hypoxia (MISTRH) [also known as NADH dehydrogenase ubiquinone 1 alpha subcomplex subunit 4 like-2 (NDUFA4L2)], which are linked through regulation by the ultraconserved microRNAs (miRNAs) *miR-147b* and *miR-210*. Our data indicate MISTRAV and the virus-encoded vMISTRAV are mitochondrial proteins in agreement with paralogs MISTR1(NDUFA4) [14] and MISTRH [15] being putative supernumerary electron transport chain (ETC) factors. Functional analysis in cell lines shows that loss of MISTRAV is associated with a reduction in apoptosis. Correspondingly, a mutation resulting in a >30-fold increase in levels of the *MISTRAV*-embedded *miR-147b* triggers a more robust activation of apoptosis activated by either the cell death agonist staurosporine or vesicular stomatitis virus (VSV). Genetic and functional analysis reveals that the rapidly evolving paralog of *MISTRAV*—*MISTR1(NDUFA4)*—is a major target of the ultraconserved *miR-147b* as well as the hypoxia-inducible *miR-210* [16] which targets the same microRNA response element (MRE) as *miR-147b*.

We propose a model for the vertebrate-specific MItochondrial STress Response (MISTR) circuit. Individual *MISTR* genes are broadly distributed with homologs in plants, animals, and parasites, along with 2 additional MISTR homologs encoded by giant DNA viruses, one that infects algae and the other fish. In addition to augmenting host immune defenses, MISTR may be a modular system with the capacity to respond to diverse stressors through regulation by specific miRNAs that down-regulate MISTR1 (NDUFA4), while concurrent induction of MISTR paralogs replaces MISTR1 (NDUFA4) to shape the mitochondrial response to perturbations.

## Results

### *MISTR* proteins are encoded by highly diverged large DNA viruses

**Fig 1:**
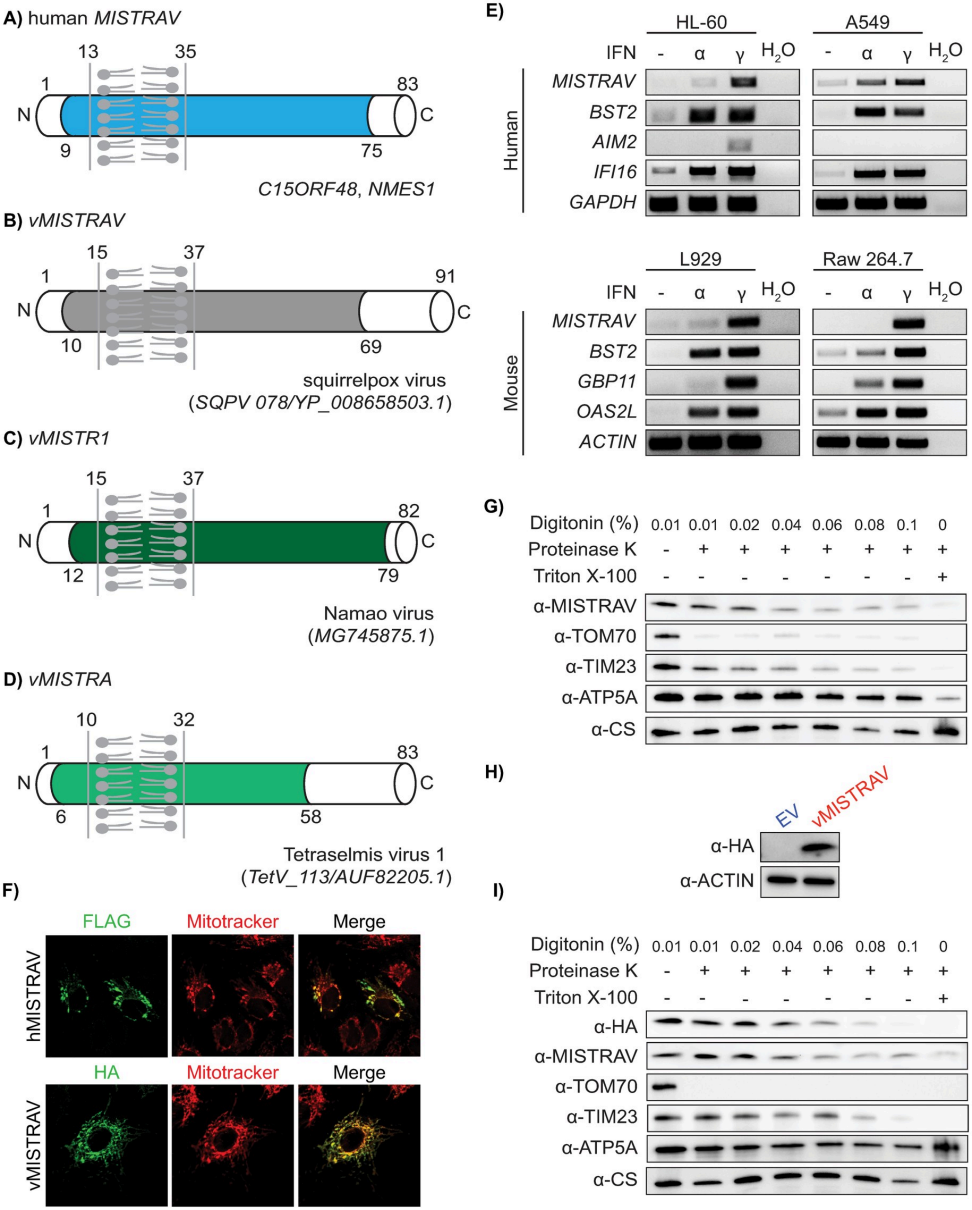
MISTRAV is a small IFNγ-stimulated mitochondrial factor also encoded by divergent viruses. **A)** Diagram of *MISTRAV* with predicted domains indicated. Colored domain represents B12D, NADH: ubiquinone reductase complex I MLRQ subunit family (pfam:06522). TMEM domain predicted using TMHMM (http://www.cbs.dtu.dk/services/TMHMM/). **B)** Diagram of *vMISTRAV*, the *MISTRAV* homolog encoded by squirrelpox, annotated with predicted domains. **C)** Diagram of *vMISTR1*, the MISTR1 homolog identified in the genome of a giant virus isolated from lake sturgeon. **D)** Diagram of *vMISTRA*, the MISTR homolog identified in the genome of a giant virus which infects algae. **E)** RT-PCR using cDNA produced from RNA from interferon-treated human and mouse cell lines. *BST2*, *AIM2*, *IFI16*, *GBP11*, and *OAS2L* are ISG controls. **F)** Confocal images of A549 cells transfected with constructs encoding *hMISTRAV-FLAG* or *vMISTRAV-HA*. **G)** Protease protection assay of mitochondria isolated from WT A549 cells. TOM70 is an OMM protein with a cytosolic α-TOM70 epitope. TIM23 and ATP5A are IMM proteins. The α-TIM23 epitope resides in the IMS, and the α-ATP5A epitope is in the mitochondrial matrix. CS is a mitochondrial matrix protein. The α-MISTRAV epitope spans from the TMEM to the carboxyl terminus of the protein, which is predicted to reside partly in the IMM with the majority in the IMS. **H)** Western blot for vMISTRAV-HA using lysates from stably expressing cells. **I)** Protease protection assay of mitochondria isolated from A549 cells stably expressing vMISTRAV-HA. HA-tag is on the carboxyl terminus of vMISTRAV. CS, citrate synthase; EV, empty vector; IFNγ, interferon gamma; IMM, inner mitochondrial membrane; IMS, intermembrane space; ISG, interferon-stimulated gene; MISTRAV, MItochondrial STress Response AntiViral; OMM, outer mitochondrial membrane; RT-PCR, reverse transcription PCR; TMEM, transmembrane; *vMISTR1*, *viral MISTR1*; *vMISTRA*, *viral MISTR* *Algae*; *vMISTRAV*, *viral MISTRAV*; WT, wild-type.

Human *MISTRAV* (*C15ORF48*, *NMES1*) encodes an 83 amino acid (AA) protein with a short N-terminus and a longer carboxyl terminus demarcated by an intervening predicted single-pass transmembrane (TMEM) domain (Fig 1A). Domain analysis indicates that *MISTRAV* belongs to the poorly characterized B12D, NADH: ubiquinone reductase complex I MLRQ subunit family (pfam:06522). Using blastp analysis, we identified a 91 AA predicted ORF (SQPV78/YP_008658503.1) with high identity to human MISTRAV [47% (38/81) amino acid identity, 66% positives (54/81)] in the squirrelpox genome, hereafter *vMISTRAV* (Fig 1B).

Reciprocal blastp analysis indicates that *vMISTRAV* was presumably acquired by HGT derived from a host copy of *MISTRAV*. Specifically, using vMISTRAV AA sequence as a query returns numerous host MISTRAV sequences—and not sequences of MISTRAV paralogs—from diverse species (additional details in S1 Text). Consistently, domain analysis indicates vMISTRAV has a similar primary structure to host MISTRAV with all of the above listed domains (Fig 1B).

Subsequent database searches detected 2 additional MISTR ORFs encoded by other viruses. Using tblastn, we identified an unannotated 82 amino acid ORF (Fig 1C) encoded by the genome of the large DNA virus isolated from lake sturgeon, Namao virus (MG745875.1) [17]. Reciprocal blastp analysis indicated that this ORF shares the most homology with the *MISTRAV* paralog *MISTR1* and hereafter is referred to as *vMISTR1*. The second viral *MISTR* ORF (*TetV-113/AUF82205*.*1*), hereafter *v**iral MISTR* *A**lgae* (*vMISTRA*), was identified in the genome of the giant DNA virus [18] Tetraselmis virus 1 (TetV-1)—a mimivirid—that infects the cosmopolitan green alga *Tetraselmis* (Fig 1D). *vMISTRA* encodes a predicted 83 AA ORF with a primary sequence similar to cellular MISTR factors encoded by *Tetraselmis* and contains all of the domains mentioned above for MISTRAV and vMISTRAV. A clustal amino acid alignment using 3 *Tetraselmis* MISTR protein sequences from the database indicates that vMISTRA displays the greatest homology with A0A061RM32 in UniProt (40% identity by blastp) (S1 Fig). Thus, sequences resembling MISTR proteins appear to have been independently acquired 3 different times by unrelated viruses that infect a diverse range of hosts from algae to mammals.

### *MISTRAV* is up-regulated by interferon and localizes to the mitochondria

A hallmark shared by many immune defense factors critical to modulating infections is the up-regulation by immune signals such as interferon. To test whether *MISTRAV* is an ISG, we performed reverse transcription PCR (RT-PCR) on RNA extracted from various human and mouse cell lines treated with either Type I (interferon alpha [IFNα]) or Type II Interferon (interferon gamma [IFNγ]). While *MISTRAV* was induced by IFNα in A549 lung epithelial cells, it was primarily up-regulated by IFNγ in the other human and mouse cell lines we tested (Fig 1E). Thus, cellular *MISTRAV* displays 2 key hallmarks of crucial immune factors like OAS1: up-regulation by immune signals and viral homologs (*vMISTRAV*, *vMISTRA*, *and vMISTR1*).

Both human and mouse (known as AA467197) MISTRAV have evidence for mitochondrial localization. The inventory of mammalian mitochondrial genes—MitoCarta [19,20]—detected MISTRAV in mitochondria across various tissues: small intestine, large intestine, stomach, placenta, and testis. In addition, MISTRAV is related to 2 known mitochondrial factors [MISTR1 (NDUFA4) and MISTRH] thought to be supernumerary factors implicated in ETC function [14,15,21]. A very recent study noted overexpressed mouse MISTRAV localized to mitochondria using immunofluorescence as well as analysis of mitochondrial and cytoplasmic fractions in HeLa cells [22]. Our immunofluorescence of transiently transfected human MISTRAV-FLAG and vMISTRAV-HA in A549 cells revealed strong co-localization with the mitochondrial marker, MitoTracker (Fig 1F). A549 cells were selected because they are often used as a model for immune activation [23] and viral infections (e.g., coronaviruses, influenza, and poxviruses) [24].

To determine submitochondrial localization of cellular MISTRAV and vMISTRAV, we performed protease protection assays on biochemically purified mitochondria from wild-type (WT) A549 cells (Fig 1G) and A549 cells stably expressing vMISTRAV (Fig 1H and 1I). The α-MISTRAV epitope spans from the TMEM to carboxyl terminus of the protein (AA: 22–71), which is predicted to reside partly in the inner mitochondrial membrane (IMM) with majority in the mitochondrial intermembrane space (IMS). Furthermore, the vMISTRAV construct was designed with an HA-tag on the carboxyl terminus of the protein. The IMM protein TIM23 also has an IMS-residing epitope. The degradation patterns for MISTRAV and vMISTRAV resembled the pattern observed for TIM23, which indicates that both proteins localize to the IMM with their carboxyl termini oriented in the IMS.

### *MISTRAV* belongs to a gene family rapidly evolving in primates

**Fig 2:**
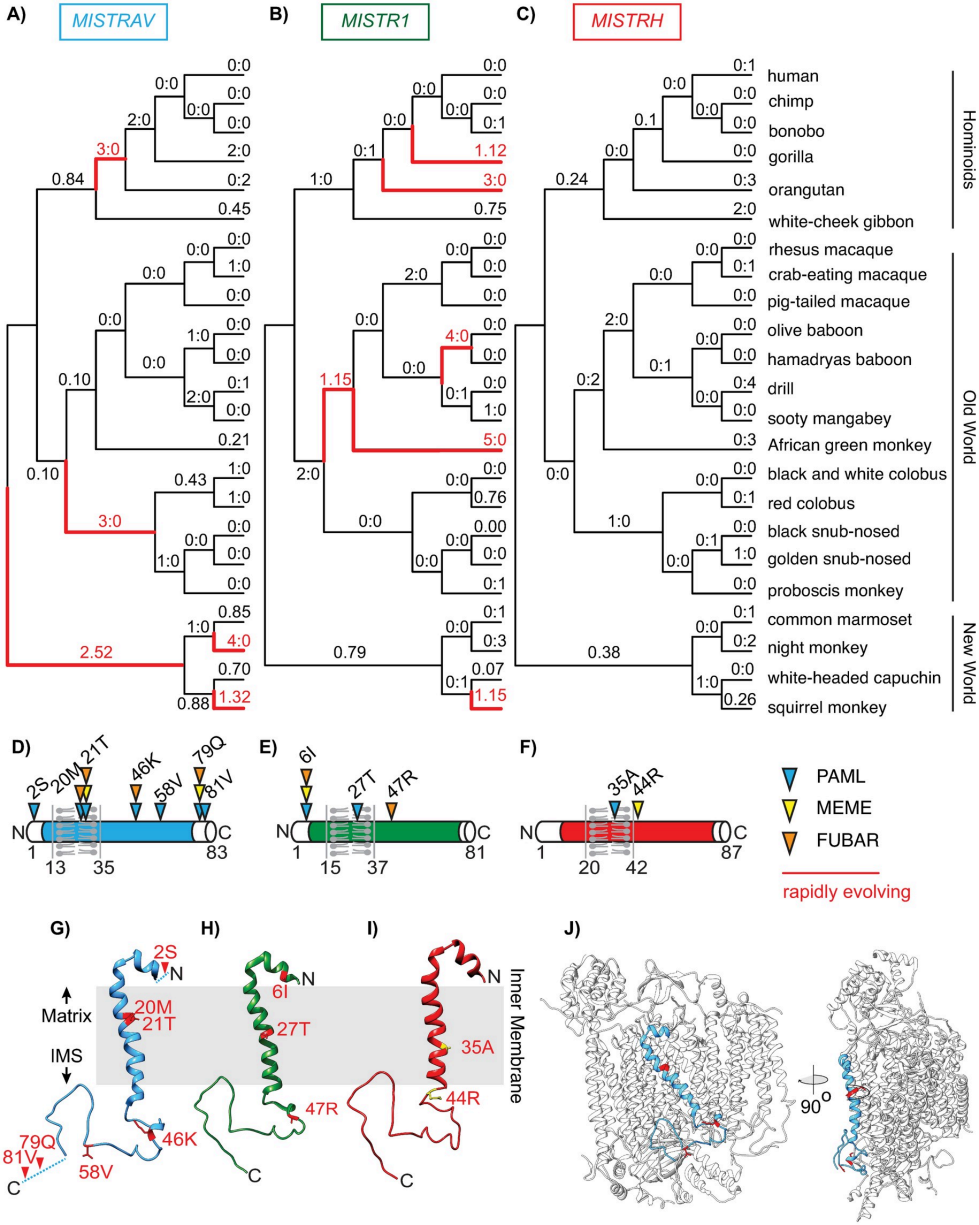
Rapid evolution of *MISTRAV* and its paralog *MISTR1* (*NDUFA4*) in primate genomes. Estimated dN/dS values predicted using FreeRatio analysis in PAML [28] across primate lineages for **A)** *MISTRAV*, **B)** *MISTR1* (NDUFA4), and **C)** *MISTRH*. Rapidly evolving lineages (dN/dS > 1 or greater than or equal to 3 nonsynonymous amino acid substitutions: synonymous amino acid substitutions) are marked by red branches. **D)** *MISTRAV* **E)** *MISTR1* (*NDUFA4*), and **F)** *MISTRH* amino acid positions predicted to be rapidly evolving (colored triangles) from PAML, MEME [30], and FUBAR [31] analysis. Numbering and residue are relative to the human reference sequence. Rapidly evolving sites for **G)** *MISTRAV* (red), **H)** *MISTR1* (*NDUFA4*) (red), and **I)** *MISTRH* (yellow) mapped onto the predicted structure of MISTR1 (NDUFA4). Models were generated using SWISS-MODEL (https://swissmodel.expasy.org/) based on the published structure of Complex IV of the ETC containing MISTR1/NDUFA4 (PDB:5Z62) [33]. **J)** Model of MISTRAV (blue) within Complex IV structure (silver). ETC, electron transport chain; IMS, intermembrane space; MISTRAV, MItochondrial STress Response AntiViral; MISTRH, MItochondrial STress Response Hypoxia; NDUFA4, NADH dehydrogenase ubiquinone 1 alpha subcomplex subunit 4.

*MISTRAV* and its poorly characterized paralogs *MISTR1* (*NDUFA4*) and *MISTRH*—are conserved at least over 450 million years of evolution as evidenced by the presence of clear orthologs in the zebrafish and spotted gar genomes (S2 Fig). To gain insights into the recent evolution of all 3 MISTR proteins, we carried out evolutionary analysis using sequences for primate orthologs spanning more than 35 million years of divergence (Fig 2, S1 Table, S1 Text). Specifically, we tested if MISTR proteins display signatures of positive selection as evidenced by elevated rates of nonsynonymous amino acid substitution relative to synonymous substitution rates (dN/dS > 1).

Positive selection may indicate that a cellular protein is in genetic conflict due to repeated targeting by pathogen-encoded inhibitors over evolutionary time [5,25]. Cellular factors displaying positive selection signatures are known to play key roles in biology such as determining infection outcomes and activation of host defenses [25]. Well-established examples of this include the major HIV-1 restriction factors APOBEC3G [26] and TRIM5α [27]. Strong selective pressure imposed by a virus on the infected host results in the increased frequency of host genetic variants in the population which are less susceptible to binding by a pathogen-encoded inhibitor.

Comparative analyses of 23 primate orthologs using codon-based models implemented in PAML [28] (Fig 2, S1 Text) revealed that both *MISTRAV* [M7 versus M8 (F3X4) *p* < 0.0012] and *MISTR1* (*NDUFA4*) [M7 versus M8 (F3X4) *p* < 0.0046] but not *MISTRH* [M7 versus M8 (F3X4) *p* < 1.0000] display gene-wide rapid evolution patterns. Our findings for *MISTR1* (*NDUFA4*) are consistent with a previous study, which included analysis of 4 hominid orthologs that identified elevated dN/dS in this gene [29]. Furthermore, these signatures in *MISTRAV* and *MISTR1* (*NDUFA4*) appear independent of any potential relaxed constraint within the predicted TMEM domain as the signal is maintained when that domain is removed in additional tests [*MISTRAV*—M7 versus M8 (F3X4) *p* < 0.0040, *MISTR1* (*NDUFA4*)—M7 versus M8 (F3X4) *p* < 0.0040]. Calculating dN/dS values across the primate phylogeny using PAML identified multiple, distinct lineages in all 3 primate families [Hominoids (HOM), Old World monkeys (OWMs), and New World monkeys (NWMs)] with robust and recurrent signatures of rapid evolution for both *MISTRAV* and *MISTR1* (*NDUFA4*).

Signatures of positive selection at specific amino acid residues can reveal key protein surfaces in genetic conflict with other factors (e.g., proteins), and the number of surfaces with elevated dN/dS values is hypothesized to correlate with the number of interfaces [5]. Using PAML [28], MEME [30], and FUBAR [31], we estimated dN/dS per amino acid site for *MISTR* genes (Fig 2D–2F). These analyses revealed 7 different amino acid positions (approximately 8% of the whole protein) distributed through *MISTRAV* with evidence of positive selection including 2 sites (21T and 79Q) identified by all 3 analyses (Fig 2D). For *MISTR1* (*NDUFA4*), 3 amino acid positions had elevated dN/dS values, with 6I being notable for its detection by all 3 analyses (Fig 2E).

Protein modeling with SWISS-MODEL (https://swissmodel.expasy.org/) [32] (Fig 2G–2I) using the only predicted structure of Complex IV to include MISTR1 (NDUFA4) [PDB:5Z62] [33] illustrates that MISTR TMEM domains are accessible for interfacing with cellular proteins. Thus, rapid evolution in the TMEM is unlikely to reflect relaxed constraint. Collectively, the rapid evolution signature observed for *MISTRAV* and *MISTR1* (*NDUFA4*) resemble that of other host factors that can dictate the outcomes of infections.

### Functional analyses support a role for *MISTRAV* and its encoded *miR-147b* in apoptosis

**Fig 3:**
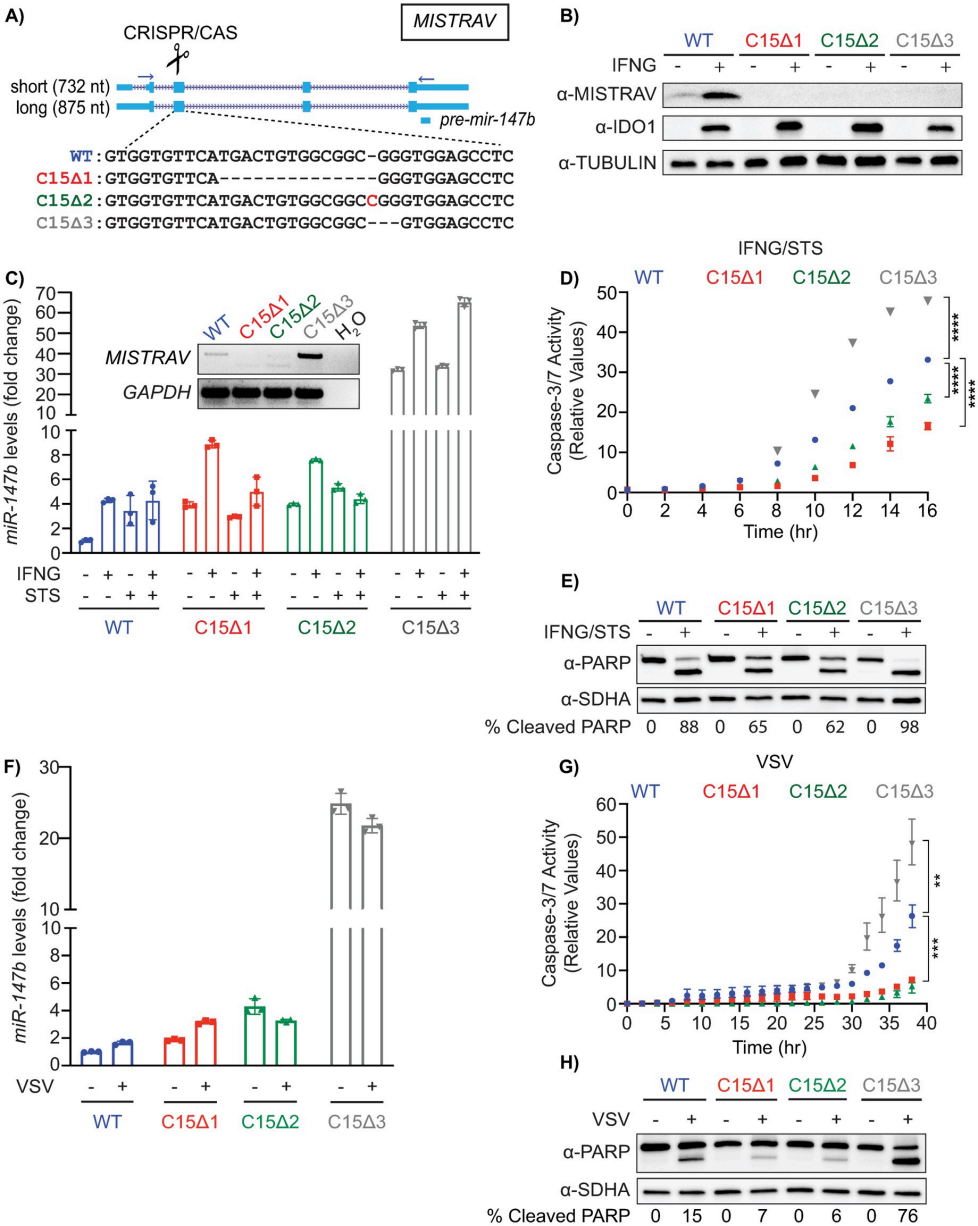
Loss-of-function analysis reveals a role for MISTRAV and its embedded miRNA—*miR-147b*—in apoptosis. **A)** Diagram of the *MISTRAV* locus from the UCSC genome browser (http://genome.ucsc.edu/) [95]. Two major transcripts are predicted for *MISTRAV*, which we term short (5 exons/predicted mRNA length 732 nt) and long (4 exons/predicted mRNA length 875 nt). The location of *pre-mir-147b* is marked by the blue box below predicted protein-coding mRNAs. Sequences of CRISPR-induced mutations targeted to exon 2 (relative to the long isoform of *MISTRAV*) in A549 cells, which result in predicted frameshifts. Deleted nucleotides are indicated by hypen (-) and inserted nucleotide is highlighted in red. **B)** Western blot analysis using protein lysates from IFNγ-treated A549 cells and *MISTRAV* deletion clones. IDO1 is an ISG control [46]. **C)** RT-PCR analysis using primers (horizontal blue arrows) in A) on cDNA produced from total RNA extracted from IFNγ-treated A549 WT and *MISTRAV* KO cells and *miR-147b* TaqMan qPCR using RNA extracted from A549 WT *and MISTRAV* KO cell lines treated with IFNγ, STS, or both for 16 hours. *miR-423* was used as the endogenous control. Fold changes in *miR-147b* levels are relative to the *miR-147b* level in WT untreated cells. **D)** Relative caspase-3/7 activity in A549 WT and *MISTRAV* KO cells pretreated with IFNγ for 24 hours followed by STS treatment for 16 hours; caspase-3/7 activity was normalized to the number of cells at the initial treatment time point measured by IncuCyte. **E)** Western blot analysis of cleaved PARP in WT and *MISTRAV* KO cells treated with IFNγ and STS. SDHA serves as loading control. **F)** *miR-147b* TaqMan qPCR using RNA extracted from A549 WT and *MISTRAV* KO cells infected with VSV-LUC for 18 hours. *miR-423* was used as the endogenous control. Fold changes in *miR-147b* levels are relative to the *miR-147b* level in WT mock-infected cells. **G)** Relative caspase-3/7 activity in A549 WT and *MISTRAV* KO cells infected with VSV-LUC at an MOI of 0.01; caspase-3/7 activity was normalized to the number of cells at the initial treatment time point measured by IncuCyte. **H)** Western blot analysis of cleaved PARP in WT and *MISTRAV* KO cells infected with VSV-LUC at an MOI of 0.01 for 18 hours. SDHA serves as loading control. In C), D), F), and G), data represent means ± SD (*n* = 3 replicates). Statistical significance in D) and G) was determined by a 2-tailed unpaired *t* test, **p ≤ 0.01, ***p ≤ 0.001, ****p ≤ 0.0001. Densitometry analysis of PARP levels in E) and H) was performed using Image Lab version 6.0.1 (Bio-Rad). % Cleaved PARP = (cleaved PARP/(Full + Cleaved PARP)) * 100. The underlying data for panels C–H can be found in S1 Data. IFNγ, interferon gamma; ISG, interferon-stimulated gene; KO, knockout; miRNA, microRNA; MISTRAV, MItochondrial STress Response AntiViral; MOI, multiplicity of infection; qPCR, quantitative PCR; RT-PCR, reverse transcription PCR; STS, staurosporine; VSV-LUC, vesicular stomatitis virus-luciferase; WT, wild-type.

To investigate *MISTRAV* biology, we generated 3 A549 clonal cell lines—C15∆1, C15∆2, and C15∆3—with distinct indels that disrupted the *MISTRAV* ORF using CRISPR/Cas (Fig 3A). Western blot analysis confirmed loss of MISTRAV protein in all 3 clones (Fig 3B). To maintain expression of a poorly characterized miRNA encoded by the 3′ UTR of *MISTRAV* (*miR-147b*) [34], we targeted the guide RNAs (gRNAs) to exon 2 relative to the long *MISTRAV* isoform (Fig 3A, 875 nucleotides [nt])—a location where a frameshift in the RNA would be predicted to escape nonsense-mediated decay.

RT-PCR indicated that C15∆1 and C15∆2 cells lack full-length (FL) *MISTRAV* RNA expression in IFNγ-treated cells at steady state, while C15∆3 cells display a fortuitous and drastic increase of the same transcript (Fig 3C, inset). miRNA quantitative PCR (qPCR) demonstrated that C15∆1 and C15∆2 maintain *miR-147b* at levels comparable to WT with expression of *miR-147b* in C15∆3 approximately 30 fold greater than WT (Fig 3C). Thus, C15∆1 and C15∆2 lack MISTRAV protein while maintaining the miRNA, while C15∆3 also lacks MISTRAV but has a gain of function in *miR-147b* expression.

Based on *MISTRAV* mitochondrial localization (Fig 1F and 1G) and numerous documented connections between immune responses involving cell death mediated through mitochondria [35], we reasoned that MISTRAV might shape apoptotic responses. To test this hypothesis, hallmarks of apoptotic cells were assayed for WT and knockout (KO) cells that were (1) treated with IFNγ followed by the addition of the commonly used activator of apoptosis, staurosporine, or (2) infected with the model RNA virus VSV as a natural cell death trigger [36,37]. Assays were normalized to either untreated controls or to the number of cells being tested to account for differences in proliferation rates (S3A Fig).

Interestingly, we observed that C15∆1 and C15∆2 displayed reduced sensitivity to staurosporine, while C15∆3 showed increased sensitivity compared to WT cells as indicated by corresponding changes in cell viability (S3B Fig), caspase-3/7 cleavage activity over time (Fig 3D), and PARP cleavage (Fig 3E). *MISTRAV* KO cells displayed similar defects in apoptosis induced by infection with a recombinant VSV that expresses luciferase (vesicular stomatitis virus-luciferase [VSV-LUC], multiplicity of infection [MOI]: 0.01) (S3C Fig). These defects included differences in caspase-3/7 cleavage activity (Fig 3G) and PARP cleavage (Fig 3H). Using luciferase activity as a reporter for VSV replication, we did not observe differences in viral replication (S3D Fig). Consistent with the ability of VSV to antagonize host gene expression [38], we did not observe major increases in *miR-147b* levels following infection (Fig 3F) as we did in the IFNγ/staurosporine-treated cells (Fig 3C). These data suggest a role for MISTRAV and *miR-147b* in chemical- and viral-induced apoptosis.

### Ultraconserved miRNAs link MISTR paralogs

**Fig 4:**
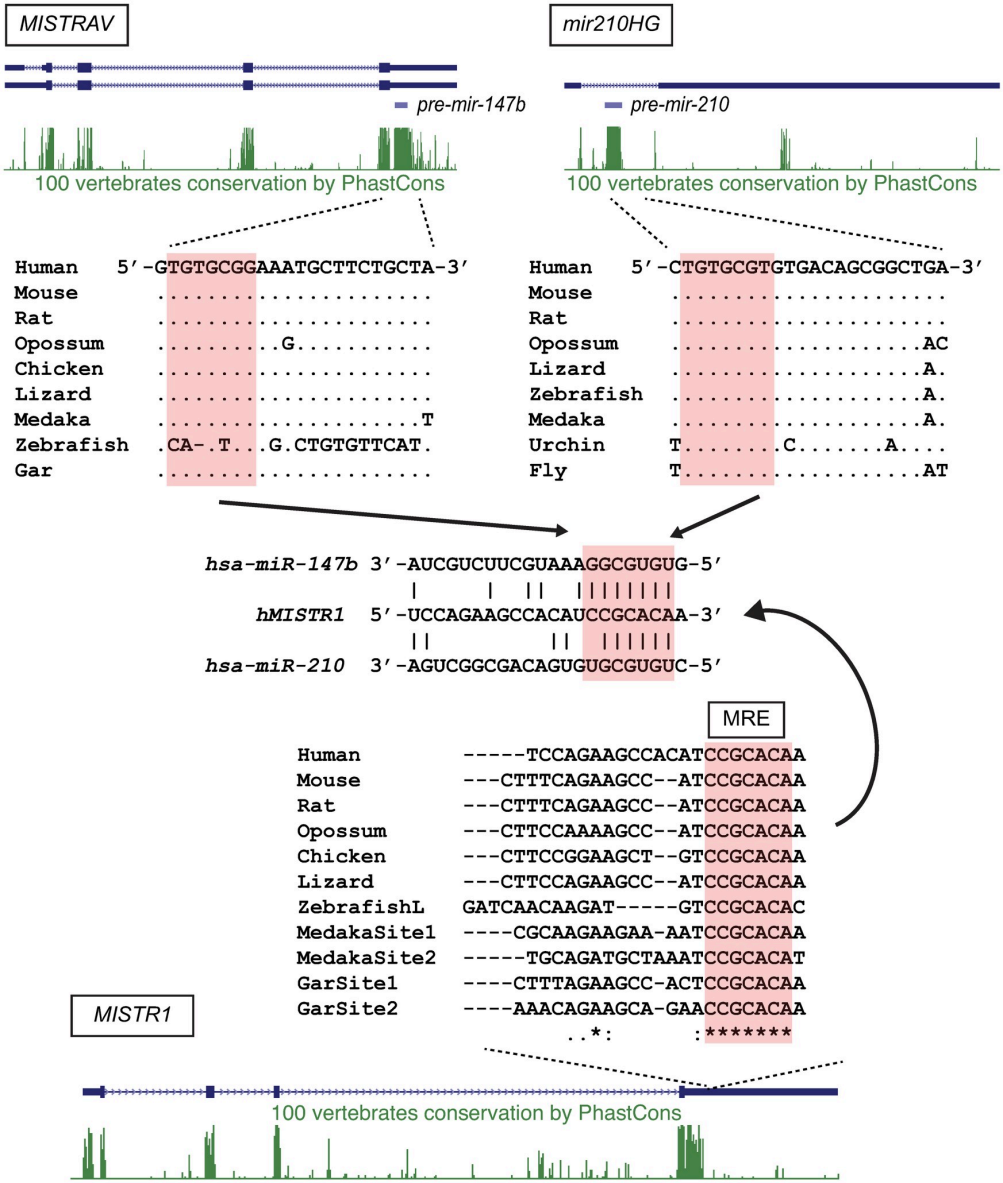
Ultraconserved miRNAs are predicted to target a vertebrate-specific MRE in *MISTR1* (*NDUFA4*). Human *MISTRAV*, *mir210HG*, and *MISTR1* (*NDUFA4*) loci with predicted gene structures and PhastCons (green peaks [96]) track from the UCSC genome browser are shown. Orthologous sequences were retrieved from the NCBI sequence database (S1 Text). Predicted seeds and MRE are marked by salmon-colored boxes. miRNA, microRNA; MISTRAV, MItochondrial STress Response AntiViral; MRE, microRNA response element; NDUFA4, NADH dehydrogenase ubiquinone 1 alpha subcomplex subunit 4.

To gain insights into the increased levels of apoptosis in C15∆3 cells associated with *miR-*147b [*miR-147* in mouse [34]], we performed comparative miRNA target analysis. A recent survey indicates that the *miR-147b* seed sequence is conserved in vertebrate orthologs [39]. Strikingly, our sequence analysis demonstrated that all 22 nts of *miR-147b* miRNA are identical between human and spotted gar, which represents around 450 million years of divergence from a common ancestor [40] (Fig 4). Interestingly, although the *MISTRAV* locus is present in the zebrafish genome, *miR-147b* sequence may be nonfunctional because of disruptive indels (Fig 4, S4 Fig).

miRNA target analysis uncovered 36 [(mirdb.org) [41,42]] and 19 [Targetscan (www.targetscan.org) [43]] *miR-147b* predicted targets (S2 Table), of which only 2 were shared by both databases: *C11orf87* and the *MISTRAV* paralog, *MISTR1* (*NDUFA4*). The predicted MRE in the 3′ UTR of the *MISTRAV* paralog, *MISTR1* (*NDUFA4*), is an 8mer seed that is perfectly conserved out to fish genomes (Fig 4). In addition, (1) the 8mer has been duplicated in some fish *MISTR1* (*NDUFA4*) orthologs (e.g., gar and medaka) (Fig 4); and (2) zebrafish maintains the predicted MRE for *miR-147b* perhaps due to interactions with the unrelated *miR-210* which has an overlapping MRE.

*miR-210* is known to be highly up-regulated by hypoxia-inducible factor 1 alpha (HIF-1α) during low-oxygen conditions and thought to be critical for the hypoxic response [16]. Assays using an MRE reporter encoding the human *MISTR1* (*NDUFA4*) 3′ UTR [44] support the functionality of this shared MRE, yet the significance has remained an open question. Evolutionary analysis indicates that the *miR-210* seed is perfectly conserved in bilateria for sequences sampled, with 19/22 nts identical between the human and *Drosophila* orthologs (Fig 4) and 21/22 nts identical between human and fish orthologs. Thus, the *MISTR1* (*NDUFA4*) 3′ UTR encodes a highly conserved MRE potentially targeted by 2 distinct ultraconserved miRNAs with an overlapping seed sequence, one of which is encoded by the paralog *MISTRAV*.

### MISTR1 (NDUFA4) is regulated by stress-inducible miRNAs

**Fig 5:**
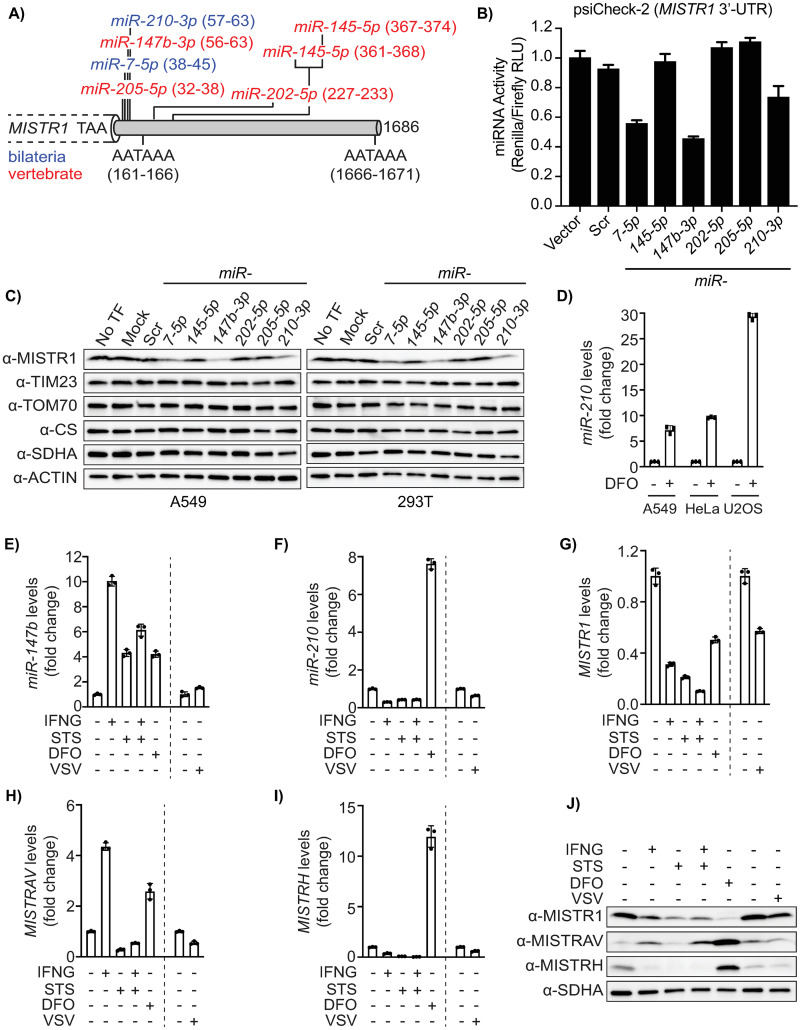
MISTR1 (NDUFA4) is a target of multiple conserved miRNAs, ubiquitously expressed, and down-regulated by stress. **A)** Diagram of predicted MREs in the FL human *MISTR1* (*NDUFA4*) 3′ UTR. Numbering is relative to the first nucleotide downstream of the stop codon for the *MISTR1* (*NDUFA4*) human reference sequence. MREs are colored by miRNA seed conservation determined by [39]: bilateria (blue) and vertebrate (red). Identified core polyA signal sequence motifs (5′-AATAAA-3′) are highlighted. **B)** miRNA reporter assays for miRNAs predicted to target *MISTR1* (*NDUFA4*). psiCheck2 encoding the FL human *MISTR1* (*NDUFA4*) 3′ UTR and candidate miRNAs were sequentially transfected into HEK293T cells followed by measurement of luciferase activity. Data represent means ± SD (*n* = 3 replicates). **C)** Western blot for endogenous MISTR1 (NDUFA4) levels in HEK293T and A549 using lysates from cells transfected with miRNAs predicted to bind the *MISTR1* (*NDUFA4*) 3′ UTR. Mitochondrial proteins residing in different mitochondrial compartments serve as controls. Actin is a non-mitochondrial loading control. **D)** *miR-210* TaqMan qPCR of 3 cell lines (A549, HeLa, U2OS) following 24 hours of DFO treatment. Fold changes in *miR-210* levels in DFO-treated cells are relative to the *miR-210* level in untreated cells. Total RNA extracted from A549 WT cells treated with IFNγ, STS, or both for 16 hours, and DFO for 24 hours, as well as A549 WT cells mock-infected or infected with VSV-LUC for 18 hours was analyzed by TaqMan qPCR for *miR-147b* **(E)** and *miR-210* **(F)** levels. The same RNA samples were also analyzed by SYBR green qPCR for *MISTR1 (NDUFA4)* **(G)**, *MISTRAV* **(H)**, and *MISTRH* **(I)** levels. *miR-423* served as an endogenous control for the miRNA qPCR assays (D-F), and *18S rRNA* served as an endogenous control for the mRNA qPCR assays (G–I). Fold changes in miRNA or mRNA levels from IFNγ-, STS-, IFNγ/STS-, and DFO-treated cells are relative to the corresponding miRNA or mRNA levels in the DMSO-treated cells. Fold changes in miRNA or mRNA levels from the VSV-infected cells are relative to the miRNA or mRNA levels of the corresponding miRNA or mRNA in the mock-infected cells, as indicated by the vertical dashed line on the graphs. Data represent means ± SD (*n* = 3 replicates). **J)** Western blot for endogenous MISTR1 (NDUFA4), MISTRAV, and MISTRH levels using lysates from A549 WT cells treated with IFNγ, STS, or both for 16 hours, and DFO for 24 hours, as well as A549 WT cells mock-infected or infected with VSV-LUC for 18 hours. α-SDHA blot serves as a loading control for mitochondrial protein stability. The underlying data for panels B and D–I can be found in S1 Data. DFO, deferoxamine mesylate; FL, full-length; IFNγ, interferon gamma; miRNA, microRNA; MISTRAV, MItochondrial STress Response AntiViral; MISTRH, MItochondrial STress Response Hypoxia; MRE, microRNA response element; NDUFA4, NADH dehydrogenase ubiquinone 1 alpha subcomplex subunit 4; qPCR, quantitative PCR; STS, staurosporine; VSV-LUC, vesicular stomatitis virus-luciferase; WT, wild-type.

TargetScan predicts 7 MREs in the *MISTR1* (*NDUFA4*) 3′ UTR for 6 distinct miRNAs [*miR-7-5p*, *miR-145-5p* (2 sites), *miR-147b-3p*, *miR-202-5p*, *miR-205-5p*, and *miR-210-3p*], which have seed sequences that are highly conserved in vertebrates with a subset extending in sequence conservation to bilateria (Fig 5A, S2C Table) [39]. A total of 5 out of the 6 miRNAs predicted by TargetScan were also predicted by miRDB—114 total miRNAs (S2D Table). Next, we tested whether a subset of these miRNAs, as determined by the overlap from MRE prediction analysis, targeted MISTR1 (NDUFA4) in a series of experiments.

MRE reporter assays using a luciferase reporter with the entire predicted 1,685-bp human *MISTR1* (*NDUFA4*) 3′ UTR (Fig 5B) revealed that transient co-transfection of *miR-7-5p*, *miR-147b-3p*, and *miR-210-3p* in HEK293T cells resulted in dramatic knockdown (40% to 65% of vector alone). Correspondingly, western blots with lysates from HEK293T and A549 cells transiently transfected with *miR-7-5p*, *miR-147b-3p*, and *miR-210-3p* (Fig 5C) demonstrated knockdown of endogenous MISTR1 (NDUFA4) protein. The knockdown of MISTR1 by these miRNAs appeared direct and not due to general effects on mitochondria as evidenced by comparable levels of mitochondrial factors from distinct compartments across the lysates (Fig 5C).

We identified 2 polyA signal canonical hexamers (AATAAA; 161–166, 1666–1671 relative to human 3′ UTR) in the *MISTR1* (*NDUFA4*) 3′ UTR, which divide the first 4 MREs from the 3 downstream sites (Fig 5A). Interestingly, the miRNAs that did not result in knockdown are located downstream of the first polyA signal, while those that did cause knockdown are located upstream of the first polyA signal. Therefore, the *MISTR1* (*NDUFA4*) 3′ UTR encodes several predicted MREs for conserved miRNAs, of which a subset is functional in cell culture assays.

### MISTR factors and corresponding miRNAs are differentially regulated by stress

To better understand the regulation and relationship of MISTR factors with *miR-147b* and *miR-210*, we performed a series of gene expression analysis using qPCR and western blot. First, we examined the regulation of *miR-210* under hypoxic conditions in 3 different cell lines (A549, HeLa, and U2OS). Consistent with previous work [16], treatment with deferoxamine mesylate (induces chemical hypoxia) led to a dramatic up-regulation (approximately 10- to 30-fold) of *miR-210* as detected by qPCR in all 3 cell lines (Fig 5D) [16]. Next, we performed gene expression analysis on A549 cells exposed to the following stress signals: IFNγ, staurosporine, IFNγ and staurosporine, deferoxamine mesylate, mock-infected, and VSV. While *miR-210* (Fig 5F) and *MISTRH* (Fig 5I) up-regulation appeared specific to deferoxamine mesylate, *miR-147b*, which shares a seed sequence with *miR-210* and is encoded by *MISTRAV*, was strongly induced under all test conditions except VSV (Fig 5E). In contrast, *MISTRAV* levels were only elevated after treatment with either IFNγ or deferoxamine mesylate. To our knowledge, this is the first report of (chemical) hypoxia induction for both *miR-147b* and *MISTRAV*.

Consistent with induction of *miR-147b* and *miR-210* under the conditions assayed, steady-state levels of *MISTR1* RNA were down-regulated by all stressors tested (Fig 5G). The less dramatic down-regulation of *MISTR1* in VSV-infected cells compared to staurosporine-treated cells, where *miR-147b* levels are high, may be attributed in part to global mRNA degradation known to occur during apoptosis [45]. Relatedly, some of the observed down-regulation in mRNA levels for the other tested MISTR factors under apoptotic conditions may also be due to global mRNA degradation [46].

Interestingly, although both *MISTRAV* and *miR-147b* are encoded by the same transcript, levels of these 2 factors did not correlate under all test conditions (Fig 5H). For example, *miR-147b* and *MISTRAV* levels were both strongly induced by IFNγ, but in staurosporine- and IFNγ/staurosporine-treated samples, *miR-147b* levels remained high despite near-complete loss of *MISTRAV* RNA. Steady-state protein levels of MISTR factors following treatment with these stressors correspond to regulation trends at the mRNA level (Fig 5J). Overall, these data suggest that stress-mediated up-regulation of *miR-147b* and *miR-210* correlates with the down-regulation of MISTR1 (NDUFA4) mRNA and protein levels. MISTR1 (NDUFA4) down-regulation is accompanied by up-regulation of MISTRH and/or MISTRAV depending on the stress insult.

### Loss of the OXPHOS factor MISTR1 (NDUFA4) results in increased sensitivity to apoptotic triggers

**Fig 6:**
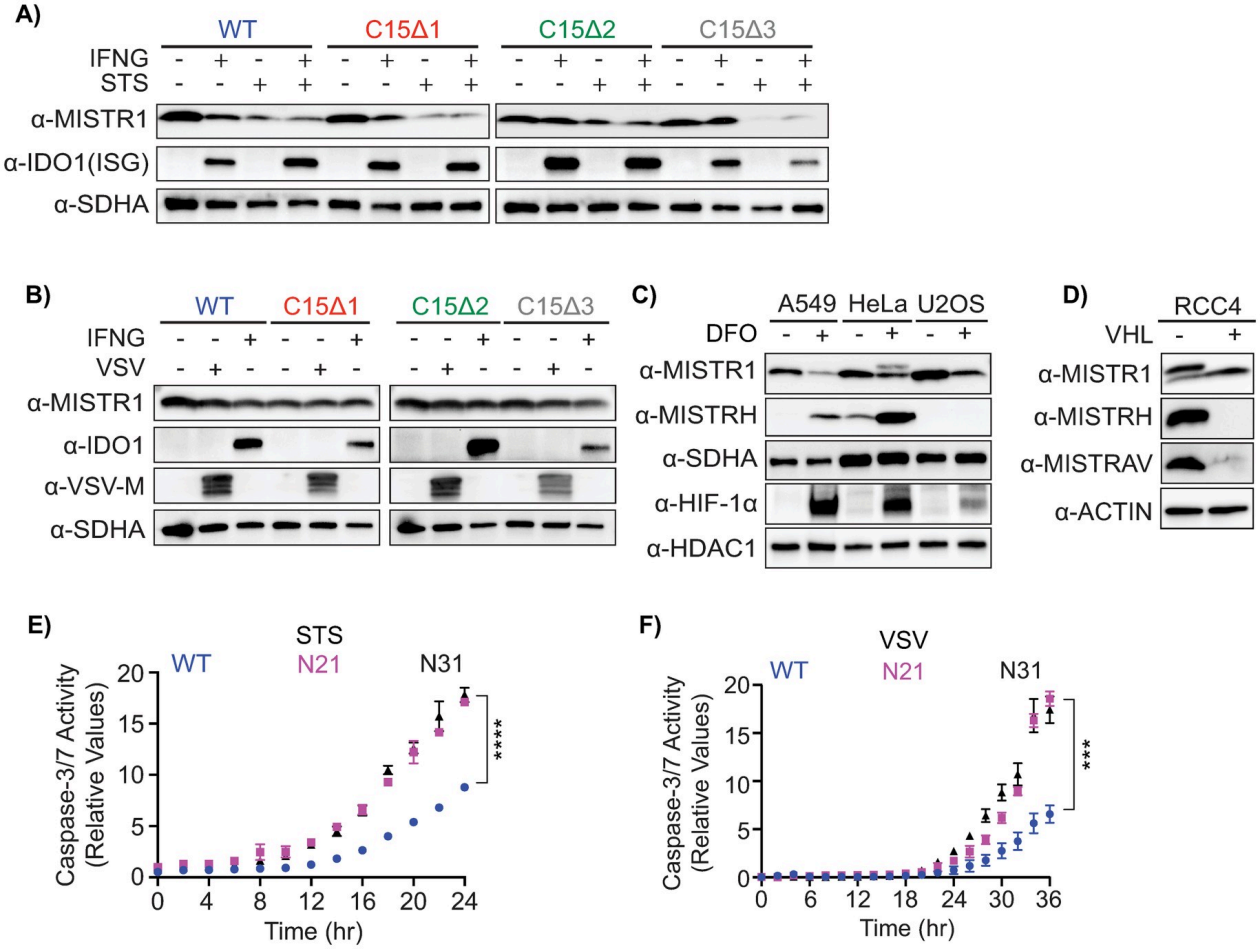
MISTR1 (NDUFA4) is down-regulated by stress, and the absence of MISTR1 (NDUFA4) enhances apoptotic responses. **A)** Western blot for endogenous MISTR1 (NDUFA4) levels using lysates from A549 WT or *MISTRAV* KO cells treated with IFNγ, STS, or both for 16 hours. **B)** Western blot for endogenous MISTR1 (NDUFA4) levels using lysates from A549 WT or *MISTRAV* KO cells mock-infected or infected with VSV-LUC for 18 hours. α-SDHA blot serves as a control for mitochondrial protein stability. **C)** Western blot analysis of MISTR1 (NDUFA4) and MISTRH levels 24 hours after chemical hypoxia induction by DFO. The upper band in the DFO-treated HeLa lane in the α-MISTR1 (NDUFA4) blot is MISTRH. SDHA, mitochondrial control; nuclear HIF-1α, hypoxia control; HDAC1, nuclear protein control. **D)** Western blot analysis of MISTR1 (NDUFA4), MISTRH, and MISTRAV levels in the RCC4 kidney cancer cell line with or without stable rescue expression of VHL. The upper band in the RCC4 (−VHL) lane in the α-MISTR1 (NDUFA4) blot is MISTRH. **E)** Relative caspase-3/7 activity in A549 WT and *MISTR1* (*NDUFA4*) KO cells treated with STS or **F)** infected with VSV-LUC. Caspase-3/7 activity was normalized to the number of cells at the initial treatment time point measured by IncuCyte. Data represent means ± SD (*n* = 3 replicates). Statistical significance was determined by a 2-tailed unpaired *t* test, ***p ≤ 0.001, ****p ≤ 0.0001. The underlying data for panels E and F can be found in S1 Data. DFO, deferoxamine mesylate; HIF-1α, hypoxia-inducible factor 1 alpha; IFNγ, interferon gamma; KO, knockout; MISTRAV, MItochondrial STress Response AntiViral; MISTRH, MItochondrial STress Response Hypoxia; NDUFA4, NADH dehydrogenase ubiquinone 1 alpha subcomplex subunit 4; STS, staurosporine; VHL, Von Hippel–Lindau; VSV-LUC, vesicular stomatitis virus-luciferase; WT, wild-type.

The regulation of MISTR1 and its relationship with *miR-147b* and *miR-210* suggest that down-regulation of this factor may be important in the cellular response to stress. To further study the role of MISTR1 (NDUFA4) in stress, we examined its regulation under additional conditions. First, we performed western blots on lysates from A549 WT and *MISTRAV* KO cells treated with staurosporine and/or IFNγ (Fig 6A). We observed dramatic down-regulation of MISTR1 (NDUFA4) following treatment with staurosporine or staurosporine/IFNγ and to a lesser extent with IFNγ alone in some lysates depending on clonal line (Fig 6A). We also observed a nearly complete loss of MISTR1 (NDUFA4) in C15∆3 mutant cells, which overexpress *miR-147b*. MISTR1 (NDUFA4) down-regulation appears either specific or rapid in comparison to levels of the mitochondrial Complex II protein SDHA, which are largely unchanged under the same conditions (Fig 6A). Assay of lysates from these same lines infected with VSV indicated no major differences in MISTR1, which we speculate may be indirectly due to VSV blocking host gene expression (Fig 6B). This speculation is supported by lack of IDO1 induction—a host defense factor up-regulated by IFNα [46] and IFNγ (Fig 3B)—in the same lysates. These data suggest that down-regulation of MISTR1 (NDUFA4) may promote apoptosis under conditions of stress induced by staurosporine but perhaps not during VSV infection.

Next, we assayed MISTR1 (NDUFA4) protein levels under hypoxic conditions. Following the induction of chemical hypoxia by deferoxamine mesylate treatment in 3 different cell lines, we observed down-regulation of MISTR1 (NDUFA4) concomitant with an up-regulation of HIF-1α and MISTRH (Fig 6C), which correspond to *miR-210* induction described earlier (Fig 5D). To assay the contribution of HIF-1α to the opposing expression of MISTR1 (NDUFA4) and MISTRH, we leveraged that HIF-signaling is constitutively active in many kidney cancers due to loss of the Von Hippel–Lindau (VHL) tumor suppressor [47]. Specifically, we performed western blot analysis on lysates from characterized RCC4 kidney cancer cells which lack VHL and RCC4 cells stably expressing VHL [48]. Compared to RCC4 cells with stable expression of VHL, RCC4 cells that lack VHL displayed decreased levels of MISTR1 and increased levels of both MISTRAV and MISTRH (Fig 6D). Up-regulation of MISTRAV in the RCC4 cells lacking VHL and its reduction to baseline levels in the RCC4 cells stably expressing VHL is in agreement with our earlier finding of MISTRAV up-regulation in A549 cells treated with deferoxamine mesylate (Fig 5H and 5J) and suggests that in addition to MISTRH, MISTRAV levels may be regulated by HIF signaling.

To test the role of MISTR1 in chemical- and viral-induced apoptosis directly, we generated 2 *MISTR1 (NDUFA4)* KO A549 clonal cell lines (N21 and N31) (S5A–S5C Fig). A control experiment showed that *MISTR1 (NDUFA4)* KO cells exhibit rates of proliferation similar to WT cells (S5D Fig). We hypothesized that *MISTR1 (NDUFA4) KO* cells would be more sensitive to apoptotic triggers for 2 related reasons: (1) cells lacking MISTRAV protein (C15∆1 and C15∆2) display decreased sensitivity to apoptotic triggers (staurosporine and VSV); and (2) MISTR1 (NDUFA4) is down-regulated during stress which is in contrast to MISTRAV’s up-regulation under the same conditions. Consistently, assay of *MISTR1 (NDUFA4)* KO cells following either staurosporine treatment (Fig 6E, S5E Fig) or VSV infection (Fig 6F, S5F Fig) using IncuCyte live-cell analysis to track caspase-3/7 activity and western blot to assess PARP cleavage indicated that these cells are more sensitive to both of these apoptotic triggers compared to WT cells. We did not observe differences in VSV replication in *MISTR1* (*NDUFA4*) KO cells using luciferase assays (S5G Fig) [15]. These data highlight a role for the constitutively expressed oxidative phosphorylation (OXPHOS) factor MISTR1 (NDUFA4) in the progression of apoptosis in response to staurosporine- and VSV-induced apoptosis.

### A broad phylogenetic distribution of MISTR proteins

**Fig 7:**
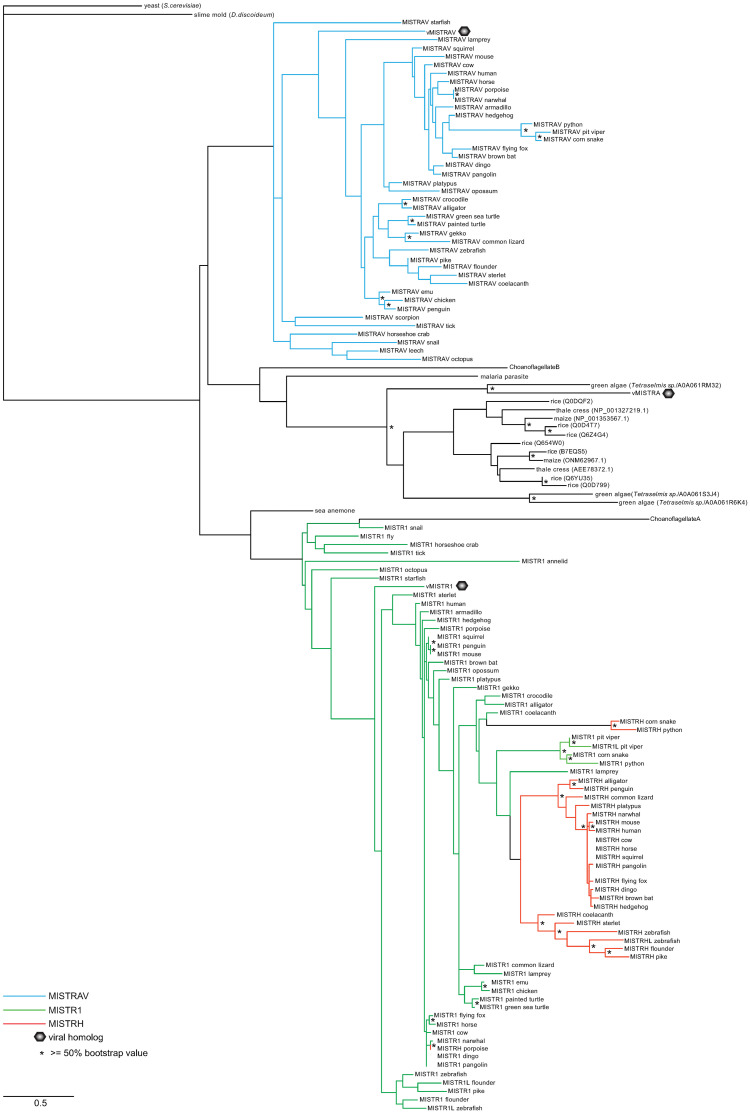
A broad phylogenetic distribution of MISTR sequences. An inferred tree built using 132 MISTR amino acid sequences by maximum-likelihood analysis using PhyML [50] (http://www.atgc-montpellier.fr/phyml/) with the VT +G model as selected by SMS and 100 bootstrap replicates. Sequences were extracted from the NCBI sequence database, Uniprot (https://www.uniprot.org/), and [14] (S3 Table, S2 Text). Bootstrap percentages from the analysis greater than 50 are indicated by asterisks. Scale for amino acid substitutions per site—bottom. MISTR, MItochondrial STress Response; MISTRAV, MItochondrial STress Response AntiViral; MISTRH, MItochondrial STress Response Hypoxia; SMS, Smart Model Selection.

To examine the implications of our findings in an evolutionary context, we surveyed the breadth of MISTR proteins across eukaryotic genomes. While a recent study detected *MISTR1* (*NDUFA4*) homologs in yeasts, including Baker’s and fission yeast, as well as *Plasmodium* [14], major gaps in the distribution and evolution of these proteins remain. We identified additional predicted proteins across animals and plants displaying homology to MISTR variants (S3 Table). These data indicate that *MISTRAV*, *MISTR1* (*NDUFA4*), and *MISTRH* sequences are all conserved in vertebrate genomes with duplications present in the zebrafish genome for *MISTR1* (*NDUFA4*) and *MISTRH*, a phenomenon common to genes of teleost fish [49]. Interestingly, we find evidence for MISTR1 and MISTRAV, but not MISTRH, homologs in protostomes. To gain additional insights related to sequence evolution of these factors, we generated multiple inferred trees for 132 (Fig 7), 157 (S6 Fig), or 185 (S7 Fig) MISTR AA sequences using maximum-likelihood phylogenetic analysis with PhyML [50–51]. The inferred trees suggest that MISTR1 (NDUFA4) and MISTRH proteins are more similar with MISTRAV being more divergent. These data illustrate that MISTR is widely distributed in genomes of diverse eukaryotes and has undergone repeated diversification, including ancestral duplications, as well as more recent evolutionary innovations.

Phylogenetic analysis can reveal hosts that viral homologs may be derived from based on relatedness. To examine the potential origins of our 3 viral MISTR homologs, we included vMISTRAV (Fig 1B), vMISTR1 (Fig 1C), and vMISTRA (Fig 1D) in these analyses. The distinct clustering of vMISTRAV with the cellular MISTRAV clade suggests this factor is derived from host *MISTRAV* and not *MISTR1* (*NDUFA4*) or *MISTRH*. While mammals were extensively sampled (S7 Fig) with the goal of uncovering a relationship between vMISTRAV and a cellular MISTRAV, no clear origin of vMISTRAV was revealed by this analysis. A tree of 132 sequences places vMISTRAV with low branch support as an outgroup of vertebrate MISTRAV sequences. Consistent with blast sequence comparisons, which indicated identity to fish MISTR1 (NDUFA4) homologs, vMISTR1 clusters with the MISTR1 homologs. In a tree of 132 AA sequences, it is positioned as an outgroup of vertebrate MISTR1 sequences, albeit, with low branch support. While the specific pairings of vMISTRAV and vMISTR1 within the tree are not maintained as the number of sequences used changes, the clustering of both viral sequences with either MISTRAV or MISTR1 homologs, respectively, is robust and maintained regardless of the sequences analyzed. In contrast, the placement of *vMISTRA* from TetV-1 with the *Tetraselmis* algae protein (A0A061RM32), which has strong bootstrap support, indicates origin of this viral protein from *Tetraselmis* or a related species. Collectively, these data indicate that these viral proteins originate from 3 independent HGT events of very divergent MISTR proteins.

### vMISTRAV antagonizes apoptotic responses

**Fig 8:**
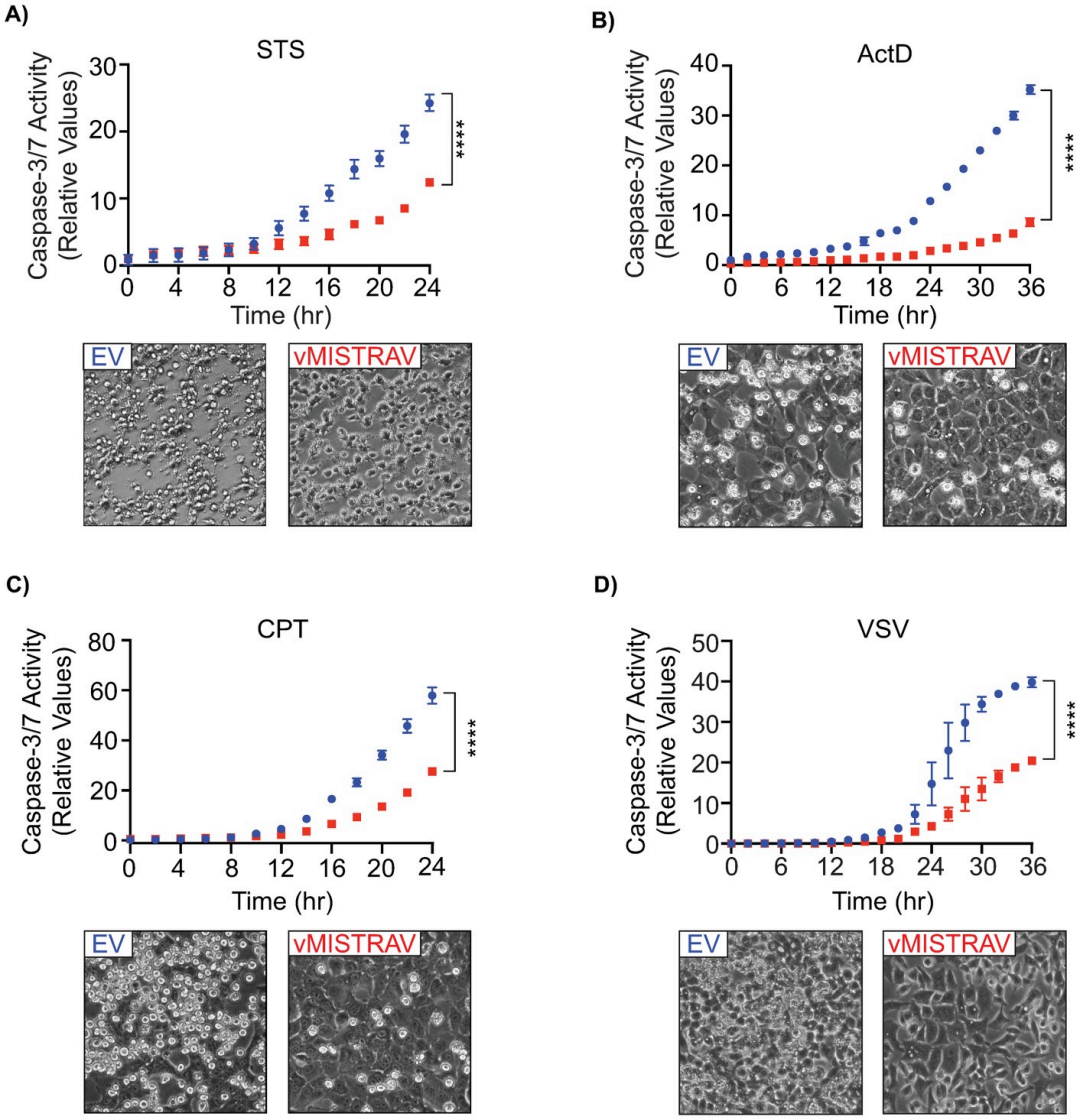
vMISTRAV antagonizes apoptotic responses. Relative caspase-3/7 activity and phase contrast images of EV and vMISTRAV-expressing cells following treatment with distinct activators of apoptosis: **A)** STS, **B)** ActD, **C)** CPT, or **D)** infection with VSV-LUC. Phase contrast images were taken 16 hours after treatment with STS or ActD, 24 hours after treatment with CPT, and 18 hours postinfection with VSV-LUC. Caspase-3/7 activity was normalized to the number of cells at the initial treatment time point measured by IncuCyte. Relative caspase-3/7 data represent means ± SD (*n* = at least 3 replicates). Statistical significance was determined by a 2-tailed unpaired *t* test, ****p ≤ 0.0001. The underlying data for panels A–D can be found in S1 Data. Actd, actinomycin D; CPT, camptothecin; EV, empty vector; STS, staurosporine; VSV-LUC, vesicular stomatitis virus-luciferase.

Our data indicate a role for cellular MISTR proteins in stress responses like apoptosis. As our loss-of-function analysis suggests that MISTRAV protein is proapoptotic, we hypothesized that vMISTRAV may counteract these responses. To test this, we leveraged our A549 cells stably expressing the squirrelpox protein with a carboxyl-terminal HA epitope tag (Fig 1H). Overexpression studies of viral proteins, such as CrmA [51,52], vFLIPs [53], MCO66L [54], p35 [55], and E1B19kd [56], have been extremely valuable in understanding host defenses including seminal findings related to the key antiviral response of apoptosis. As a control, we confirmed these cells grew at the same rate as control cells expressing an empty vector (EV) (S8A Fig). Next, we assayed hallmarks of apoptosis in these cells following either treatment with 1 of 3 different chemical activators of apoptosis—staurosporine, actinomycin D, and camptothecin—or infection with VSV. Consistent with our hypothesis, we observed antiapoptotic activity associated with vMISTRAV as indicated by marked decreases in relative caspase-3/7 activity (Fig 8) as well as decreases in the percentage of cleaved PARP (S8B and S8C Fig) compared to EV controls. The antiapoptotic activity of vMISTRAV was robust as evidenced by differences in cells undergoing apoptosis with and without expression of this factor (Fig 8). We did not observe differences in VSV replication in vMISTRAV cells using luciferase assays (S8D Fig). These data suggest that vMISTRAV counteracts chemical apoptosis triggered by distinct mechanisms as well as VSV-triggered apoptosis.

## Discussion

### *MISTRAV* displays hallmarks of a critical immune defense function

A high priority of immunological research is to assign functions, define interactions, and uncover regulatory mechanisms of factors regulated by immune signals like ISGs. To address this major knowledge gap, we applied hallmarks of genetic conflict to identify MISTRAV as a candidate of interest. Our characterization of a combination of hallmarks common to crucial immune factors paired with functional analysis revealed MISTR factors as highly conserved, but mostly uncharacterized, cellular proteins important for the key host defense process of apoptosis. We define *MISTRAV* as an IFNγ-inducible gene (Fig 1E) and protein (Fig 3B), which builds on previous work showing that *MISTRAV* is induced by other immune signals: LPS, poly I:C, and PAM3SCK4 in primary mouse and human macrophage cell lines [34], LPS in human primary effector dendritic cells [57], and IFNα [4]. Interestingly, our phylogenetic analysis (Fig 7) suggests that MISTRAV-like factors predate many of these signaling pathways including canonical IFNα [58] and IFNγ signaling [59], which emerged early in vertebrate evolution.

Several lines of evidence suggest that cellular MISTRAV and its function/s are targeted for inactivation by multiple pathogens. Specifically, signatures of rapid evolution we detected in primate genomes for *MISTRAV* (Fig 2A and 2D) point to repeated antagonistic interactions on multiple protein surfaces. The signatures of positive selection observed for the nuclear-encoded MISTRAV and MISTR1, but not MISTRH, may also be due to other conflicts such as interactions with rapidly evolving mitochondrial factors, but each scenario will have to be interrogated experimentally. Regardless, the 3 MISTRAV domains, which remain functionally undefined (N-terminus, TMEM domain, and carboxyl terminus), all display evolutionary patterns consistent with genetic conflicts [6,26]. We predict that rapidly evolving surfaces on opposite sides of the TMEM, which may be otherwise shielded by the mitochondrial inner membrane, represent unique surfaces targeted by distinct factors such as pathogen-encoded inhibitors.

While positive selection predicts direct inhibitors of MISTRAV and MISTR1 (NDUFA4) functions, the presence of 3 viral homologs (*vMISTRAV*, *vMISTR1*, and *vMISTRA*) supports the idea that viruses also counteract this defense pathway via mimicry. Independent acquisition of related proteins by viruses that infect highly divergent hosts is thought to be extremely rare with the largest evolutionary span thus far being distinct copies of interleukin (IL)-10 encoded by herpesviruses which infect fish and mammals [60]. To our knowledge, these are the first ETC-associated genes known to be acquired by viruses. Notably, while *MISTRAV*, *OAS1* [7,8], *cGAS*, *MX1* [61], *APOBEC3G* [26], *ZAP* [62,63], *BST* (tetherin) [64,65], and *PKR* [6] are all rapidly evolving and up-regulated by interferon, only *MISTRAV* and *OAS1* [9] homologs are known to be encoded in virus genomes.

These observations indicate that the MISTR pathway provides a vital cellular defense that can influence the outcome of infections. In agreement with our hypothesis, deletion of *MISTRAV* (Fig 3D, 3E, 3G and 3H, S3B and S3C Fig) as well as *MISTR1* (*NDUFA4*) (Fig 6E and 6F, S5E and S5F Fig) result in defects in chemical- and VSV-induced apoptosis. Relatedly, it has been demonstrated that haploinsuffciency in cardiomyocytes of NDUFA13—a subunit of Complex I—leads to decreased apoptosis following ischemia/reperfusion injury [66]. This finding along with our studies highlights that ETC accessory factors may shape programmed cell death outcomes.

### MISTR1 (NDUFA4) bridges the electron transport chain and stress responses

MISTR1 (NDUFA4) has been shown to associate with ETC complexes and is presumed to act as a structural component, but additional functional roles are a matter of debate [14,67–69]. MISTR1 (NDUFA4) loss of function caused by a homozygous splice donor mutation is associated with the neurological disorder Leigh’s syndrome [68]. In addition, deletion of MISTR1 (NDUFA4) has been identified in a quantitative trait locus (QTL) analysis associated with diet-induced diabetes in a rat model characterized by ETC dysfunction [70]. MISTR1’s annotation as NDUFA4 comes from initial findings that it co-purifies with Complex I [71]. More recent work has provided evidence for a primary Complex IV association [14]. The presence of MISTR1 (NDUFA4) on the external surface (Fig 2J) of Complex IV was interpreted as a means of regulating higher-order ETC complex formation into supercomplexes [33]. Our data implicate a role for MISTR1 (NDUFA4) as a critical step for cells to respond to stresses including chemical- and viral-induced apoptosis (Fig 6E and 6F). High levels of conservation of *MISTR1* (*NDUFA4*) MREs for *miR-210* and *miR-147b* (Fig 4) suggest the necessity of down-regulating MISTR1 (NDUFA4) during immune signaling and hypoxia (Fig 5). Furthermore, the discovery of vMISTR1 indicates that functions of MISTR1 are targeted during infection to favor viral replication at least for Namao virus.

### MISTR is a vertebrate-specific stress response circuit

**Fig 9:**
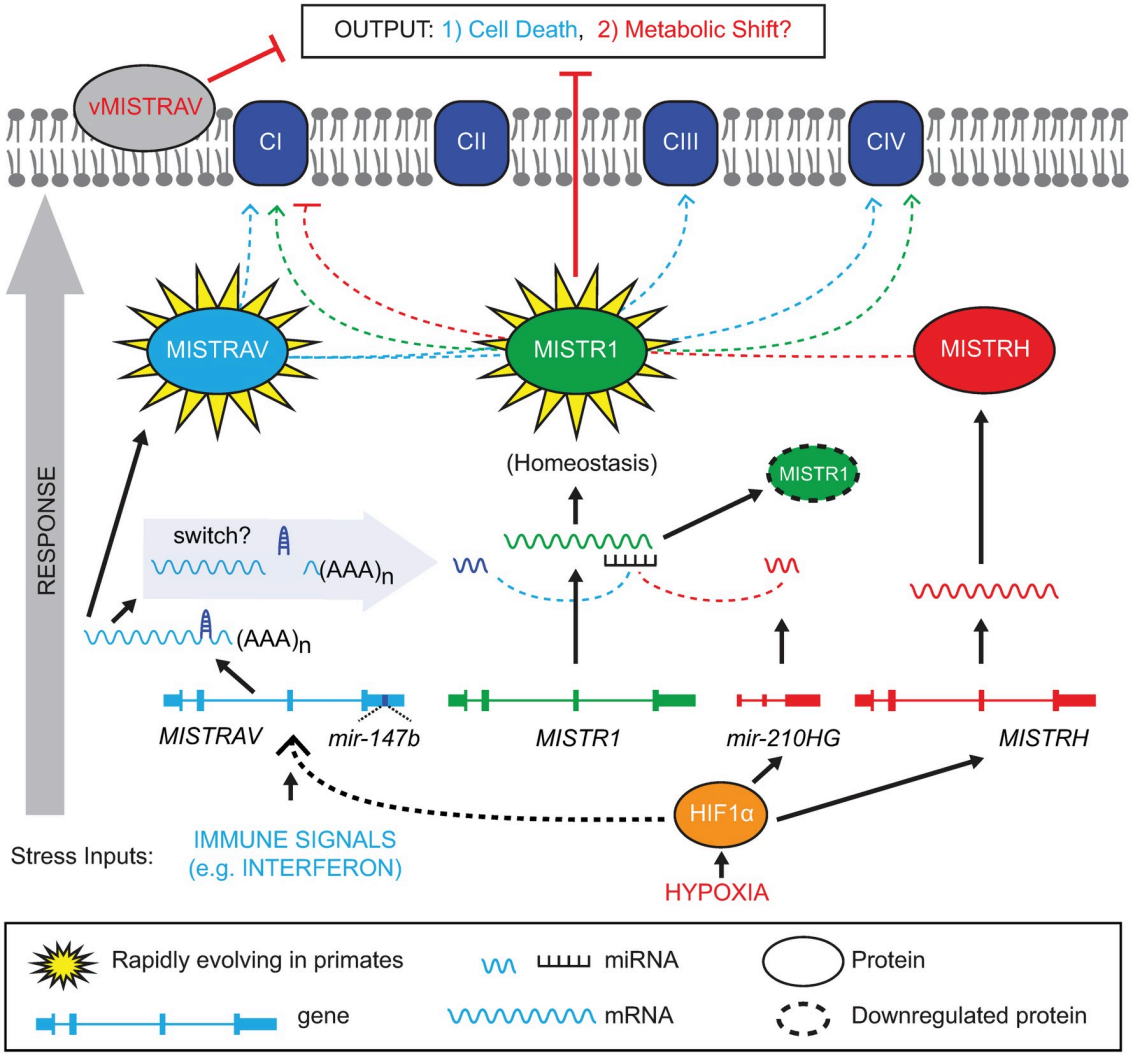
MISTR circuit model. Schematic diagram of the MISTR network showing published interactions with the ETC complexes and vertebrate MISTR proteins (MISTRAV, MISTR1 (NDUFA4), and MISTRH). MISTR loci and RNA produced from them including *miR-147b* and *miR-210* are also illustrated. The presence of MISTR1 is important to maintain homeostasis and counteract stress responses like cell death and metabolic shift. Immune signals such as interferon induce transcription of *MISTRAV* (Figs 1E and 3B) resulting in the production of *MISTRAV* RNA. MISTRAV protein localizes to the mitochondria (Fig 1F and 1G), to promote host defense. In the model, *miR-147b* production acts to inactivate MISTRAV translation and down-regulate MISTR1 (NDUFA4)—represented by MISTR1 with dashed border—to facilitate the apoptotic response (Figs 3, 5 and 6). Viral-encoded homologs (vMISTRAV) counteract the response by inhibiting apoptosis through mimicry of MISTR components. In the case of hypoxic stress, *MISTRH* and *mir-210HG* are transcribed from distinct loci to produce MISTRH [15], which can inhibit Complex I activity [15] (dashed red line), while *miR-210* [16] down-regulates MISTR1 (NDUFA4) to promote the cellular hypoxic response. Our data also suggest that in addition to *miR-210* and MISTRH, *miR-147b* and MISTRAV are also up-regulated by hypoxic stress (Figs 5 and 6). The outputs are color coded to link the respective arms of the response: MISTRAV (cell death/light blue/this study) and MISTRH (metabolic shift/red/[15]). Rapid evolution of *MISTRAV* and *MISTR1* (NDUFA4) (Fig 2) is highlighted by yellow stars. Blue dashed lines from MISTRAV indicate potential ETC complex interactions from published data including protein–protein interactions proposed from mass spec analysis [21]. The green and red-dashed lines from MISTR1 [14,33,67–69] and MISTRH [15], respectively, to ETC complexes represent reported interactions/associations. Gray arrow on the left, indicates the direction of the response (i.e., stress input to output). Although MISTR proteins may be embedded components in the mitochondrial inner membrane undergoing stress regulated and miRNA-mediated exchanges, they are shown as circles for clarity in the model. ETC, electron transport chain; miRNA, microRNA; MISTR, MItochondrial STress Response; MISTRH, MItochondrial STress Response Hypoxia; NDUFA4, NADH dehydrogenase ubiquinone 1 alpha subcomplex subunit 4.

Integrating evolutionary analysis with experimental genetics and related functional analysis led us to define a model for the MISTR circuit (Fig 9). While some previous studies hinted at potential interactions for MISTR components, functional connections were largely unknown. For instance, *miR-147b* and *miR-210* were shown to share a seed sequence and to have the ability to down-regulate an MRE reporter encoding the *MISTR1* (*NDUFA4*) 3′ UTR when transfected [44]. In addition, *miR-147b* functions were recently associated with the tricarboxylic acid (TCA) cycle [72], but the observation that *miR-147b* was encoded by the same gene as a *MISTR1* (*NDUFA4*) paralog had not been reported (Fig 4) [44]. Likewise, the overexpression of endogenous MISTRH correlating with loss of MISTR1 (NDUFA4) protein (Fig 6D) had been observed in clear cell Renal Cell Carcinoma (ccRCC) tumor samples and ccRCC cell lines [73], a disease characterized by hyperactive HIF signaling [47], but up-regulation of MISTRAV and the requirement of HIF in the regulation of this newly proposed circuit had not been tested (Fig 6D).

Our model predicts that MISTR1 (NDUFA4) is a ubiquitously expressed sensor of stress. Specific stress signals induce miRNA expression leading to the down-regulation of MISTR1 (NDUFA4) and its replacement by inducible paralogs to promote apoptosis or some form of stress tolerance. The presence or absence of different MISTR factors may alter either OXPHOS activity or ETC complex composition/abundance or both to permit downstream events in response to stress. Indeed, caspase-3 cleavage of NDUFS1—a component of Complex I—is necessary for the loss of OXPHOS activity during extrinsic (tumor necrosis factor [TNF]) and intrinsic apoptosis [74]. Whether MISTR factors shape OXPHOS activity as a means to drive antiviral responses like apoptosis remains an open question. Striking conservation of the miRNAs targeting *MISTR1* (*NDUFA4*) and cognate MREs (Fig 4) indicate that MISTR-like responses are likely common in many diverse vertebrate species. Interestingly, the potential co-expression of MISTRAV, MISTRH, *miR-147b*, and *miR-210* during hypoxic conditions may indicate complementary or perhaps partial redundant functions of these factors under stress. Not mutually exclusive, this regulation may also suggest additional biology for these factors during the immune response as viruses are known to activate the hypoxic response. Infections with several viruses such as vaccinia virus [75], human papillomavirus [76,77], Kaposi’s sarcoma-associated herpesvirus [78], Epstein–Barr virus [79–81], hepatitis B virus (HBV) [82], and hepatitis C virus (HCV) [83] have been reported to induce a hypoxic response under normoxic conditions through stabilization of HIF-1α.

The embedded nature of *miR-147b* suggests undefined mechanisms regulating the activity of this small RNA. In principle, processing of *miR-147b* from the *MISTRAV* RNA should uncouple the mRNA cap from the polyA tail rendering translation of MISTRAV infeasible by depleting the number of available mRNAs used to produce protein. This relationship would predict inverse expression patterns. Indeed, *miR-147b* levels are high when *MISTRAV* RNA levels are extremely low in staurosporine and IFNγ-/staurosporine-treated cells (Fig 5E and 5H). Interestingly, during IFNγ as well as deferoxamine mesylate treatment, both *miR-147b* and *MISTRAV* levels are high. The “either/or” and “co-expression” patterns across conditions may imply regulatory mechanisms that maintain *MISTRAV* RNA at steady levels allowing for processing of this transcript to *miR-147b* under staurosporine-induced apoptosis. Consistent with this prediction and our findings (Fig 3D, 3E, 3G and 3H, S3B and S3C Fig), MISTRAV and *miR-147b* are likely to have related but separate functions, which is similar to 2 other reports of exonic miRNAs and their host genes [84,85]. Furthermore, posttranscriptional mechanisms might also regulate mature *miR-147b* activity or its ability to target *MISTR1* (*NDUFA4*). Strikingly, despite the high levels of *miR-147b* in C15∆3 (Fig 3C), including at baseline, gross down-regulation of MISTR1 (NDUFA4)—associated with the gain-of-function mutation in C15∆3—does not occur until staurosporine is present (Fig 6A).

In contrast to the linked nature of *MISTRAV/miR-147b*, *miR-210* and *MISTRH* are encoded at distinct loci in an arrangement more permissive to complementary functions. Specifically, *miR-210* is located within an intron of an uncharacterized noncoding RNA—called *miR-210HG* in humans. Here, processing of *miR-210* would not be predicted to inactivate the host gene. Therefore, *miR-210* and *miR-210HG* may share currently uncharacterized complementary functions. The distinct arrangements of *miR-147b* and *miR-210* are consistent with differences in cellular responses to hypoxia and immune signals produced during infection. Namely, under hypoxia, the cell will buffer itself from low-oxygen conditions enabling survival, while during infections there are more drastic, escalating levels of responses culminating in apoptosis to eliminate virus-infected cells.

Our data indicate that loss of MISTRAV reduces apoptosis in response to VSV infection, while MISTR1 deletion results in increased sensitivity to VSV-induced apoptosis with neither situation resulting in changes in VSV replication. A possible VSV antagonist of MISTR functions could account for this, but this antagonist would have to function in a manner that does not antagonize the roles of these proteins in apoptosis. We anticipate future studies by us or others will likely uncover decreases in viral replication by examining additional viruses and different cell types. It is possible that no viral replication phenotype is observed unless an animal model is used perhaps due to cell-to-cell communication or relatedly, the defect in apoptosis has a non-cell autonomous activity which leads to inhibition of viral replication. Nevertheless, MISTR factors join a select class of factors, which includes *IL-10* [60], *RAS* [12], and *BCL2*-proteins [86], that have been acquired by viruses more than once. Putting these findings together, the MISTR system represents an evolutionarily dynamic circuit interfacing with fundamental cellular processes to mediate stress responses that can be targeted by viruses.

## Materials and methods

Detailed reagent information is available in S5 Table.

### Sequence analysis

Domain searches were performed using Interpro (https://www.ebi.ac.uk/interpro/), NCBI Conserved Domains (https://www.ncbi.nlm.nih.gov/Structure/cdd/wrpsb.cgi), and TMHMM for TMEM domain prediction (http://www.cbs.dtu.dk/services/TMHMM/).

### Rapid evolution analysis

Primate nucleotide sequences were retrieved from the NCBI database (S1 Table, S1 Text). Multiple sequence alignments (MSAs) were performed using Muscle in Geneious 11.1.5 (BioMatters, New Zealand). Indels were removed from alignments by manual trimming. To obtain dN/dS lineage estimates, the MSA for each gene and newick phylogenetic tree of sampled primates (based on known relationships [87]) served as input for FreeRatio analysis implemented in PAML [28]. PAML NSsites analysis was carried out with 2 codon frequency models F3X4 and F61. Analyses were also performed using MEME [30] and FUBAR [31] from Datamonkey (http://www.datamonkey.org) [88] to predict rapidly evolving sites. Additional summary of findings is present in S1 Text.

### Phylogenetic analysis

MISTR amino acid sequences and related information were retrieved from NCBI using blastp and tblastn, Uniprot, and [14] (S3 Table). Any discrepancies in whether a protein was a specific MISTR (e.g., MISTR1 versus MISTRH) were interrogated using reciprocal blastp analysis. Homologs for species were selected for analysis to assay these poorly characterized factors. MSA of amino acid sequences were performed using Clustal Omega implemented in Geneious Prime with manual adjustments as needed. The amino acid alignments for 132, 157, and 185 AA sequences are present in S2 Text. Phylogenetic analysis was performed using PhyML. Model selection was performed by Smart Model Selection (SMS) [89] integrated into PhyML. The VT +G model was selected for tree building using 100 bootstrap replicates. FigTree v1.4.2 (http://tree.bio.ed.ac.uk/software/figtree/) was used for tree visualization.

### Cell lines

HeLa, HL-60, L929, Raw 264.7, and HEK293T cell lines were obtained from ATCC. RCC4 (+/−) VHL cell lines were purchased from Sigma (USA). A549 and U2OS cells were generous gifts from Dr. Susan Weiss of the University of Pennsylvania and Dr. Don Gammon of the University of Texas Southwestern Medical Center, respectively. All cell lines except RCC4 were cultured in Corning DMEM with L-Glutamine, 4.5 g/L Glucose, and Sodium Pyruvate supplemented with 10% FBS and 1X Gibco Antibiotic-Antimycotic solution. The Antibiotic-Antimycotic solution was replaced with 0.5 mg/mL G418 in the media for RCC4 cells. All cell lines were maintained at 37°C in a humidified incubator at 5% CO_2_.

### Mitochondrial isolation and protease protection assay

Mitochondria were isolated from WT A549 cells and A549 cells stably expressing HA-tagged vMISTRAV using Mitochondrial Isolation Kit for Cultured Cells (Abcam, USA) following the manufacturer’s protocol. Concentration of the isolated mitochondria was determined using a Bradford assay and adjusted to 1 mg/mL. Protease protection assays were performed following previously published protocols [90,91]. Briefly, 30 μg aliquots of mitochondria were solubilized with increasing concentrations of digitonin or 1% Triton X-100 for 10 minutes on ice. Proteinase K was added to a final concentration of 100 μg/mL and incubated on ice for 30 minutes. Proteinase K digestion was stopped by addition of 5 mM PMSF. Samples were subjected to western blot analysis.

### Cell culture treatments

The following were added to cells at the indicated concentrations unless otherwise noted: Staurosporine [1 μM (Abcam)], IFNα [1,000 U/mL (PBL Assay Science, USA)], Interferon Gamma [1,000 U/mL (ThermoFisher, USA)], Actinomycin D [1 μg/mL (Cayman Chemical, USA)], Camptothecin [1 μM (Tocris, USA)], and Deferoxamine mesylate [300 μM (Abcam)].

### VSV infections and detection

A549 cells were plated at 5 × 10^3^ cells/well in opaque white 96-well plates (Corning, USA) in 75 μL of media. The following day, media was removed, and cells were inoculated with 37.5 μL of growth medium containing VSV-LUC (generous gift from Dr. Sean Whelan) [92] at an MOI of 0.01 for 1 hour at 37°C. After the 1-hour incubation, 37.5 μL of media was added to bring up the volume in each well to 75 μL. Viral replication was assessed 18 hours postinfection using the Bright-Glo Luciferase Assay System (Promega, USA) following the manufacturer’s instructions. For western blot and qPCR analysis of infected cells, cells were plated in 6-well plates. The next day, spent media was replaced with 1 mL of fresh media containing VSV-LUC at an MOI of 0.01. After the 1-hour incubation, 1 mL of media was added to bring the volume in each well up to 2 mL. Cells were collected for analysis at 18 hours postinfection.

### RT-PCR

Total RNA was extracted using the *Quick*-RNA Miniprep Kit (Zymo, USA) according to the manufacturer’s instructions. A total of 1 μg of total RNA was reverse-transcribed using the Maxima First Strand cDNA Synthesis Kit (ThermoFisher) for 10 minutes at 25°C, 30 minutes at 50°C, and 5 minutes at 85°C. The 20-μL cDNA reaction was subsequently diluted with water to a final volume of 100 μL. Moreover, 1 to 2 μL of cDNA was used for 25-μL PCR reactions using the GoTaq Hot Start Master Mix (Promega) (primer sequences in S4 Table). Cycling parameters consisted of an initial denaturation of 95°C for 2 minutes, followed by 28 to 30 cycles of 95°C for 30 seconds, 50°C for 30 seconds, and 72°C for 30 seconds finishing with a final elongation at 72°C for 2 minutes. PCR products (20 μL each) were resolved by 2% agarose gel electrophoresis and visualized using ethidium bromide.

### CRISPR knockouts

For *MISTRAV* KOs, DNA oligos encoding gRNAs were synthesized (IDT, USA) and cloned into pSpCas9(BB)-2A-Puro vectors (gift from Feng Zhang, Addgene #62988) according to the protocol here [93]. gRNAs were positioned in exon 2 (long isoform) with the expectation based on rules of nonsense-mediated decay such that frameshifts here would be predicted to disrupt the *MISTRAV* ORF while maintaining expression of *miR-147b*. A549 cells were transfected with the gRNA construct, followed by puromycin (Invivogen, USA) selection. Subsequently, limited dilution was performed to establish clonal cell lines. Clones of interest were identified by PCR on genomic DNA harvested with the *Quick*-DNA Miniprep Kit (Zymo) from expanded cell lines using primers flanking exon 2 followed by Sanger sequencing of amplicons by Genewiz (USA). For *MISTR1* (*NDUFA4*) KOs, gRNAs (IDT) were transfected with Cas9 and tracRNA from IDT into A549 cells. Clones were isolated via limiting dilution. gRNAs (S4 Table) were designed using crispr.mit.edu and idt.com.

### vMISTRAV stable cell line

vMISTRAV was synthesized (IDT) as a gene block with a carboxyl-terminal HA tag and cloned into pMSCV PIG (Puro IRES GFP empty vector)—a gift from David Bartel (Addgene plasmid # 21654). Retroviruses were generated using the retroPack system (Takara, USA) according to manufacturer’s instructions. Following infection of A549 cells, puro selection was performed to select for vMISTRAV-expressing cells.

### Western blot analysis

Cells were collected and lysed with RIPA Lysis and Extraction Buffer (ThermoFisher) supplemented with 1X Halt Protease Inhibitor Cocktail (ThermoFisher). For the HIF-1α western blots, nuclear fractions were extracted using Abcam’s Nuclear Fractionation Protocol. Cells cultured in 10-cm dishes were scraped in 500 μL of ice-cold Buffer A (10 mM HEPES, 1.5 mM MgCl2, 10 mM KCl, 0.5 mM DTT, 0.05% NP40, pH 7.9, and 1X Halt Protease Inhibitor Cocktail), transferred to 1.5 mL microcentrifuge tubes, and incubated on ice for 10 minutes. Lysates were centrifuged at 4°C at 3,000 rpm for 10 minutes. Each pellet was resuspended in 374 μL ice-cold Buffer B (5 mM HEPES, 1.5 mM MgCl2, 0.2 mM EDTA, 0.5 mM DTT, 26% glycerol (v/v), pH 7.9, and 1X Halt Protease Inhibitor Cocktail) and 26 μL of 4.6M NaCl (final NaCl concentration: 300 mM), homogenized using a syringe with a narrow-gauge needle (27G), and incubated on ice for 30 minutes. Lysates were centrifuged at 4°C at 24,000 × g for 20 minutes. The supernatant containing the nuclear fraction was transferred to a new tube. Protein concentrations of the extracts were measured using a Bradford assay. Protein samples were subjected to SDS-PAGE and wet-transferred to a 0.2 μM Immobilon-PSQ PVDF membrane (Millipore, USA) at 200 mA for 90 minutes. Membranes were blocked with blocking buffer (5% BSA or milk in TBST) for 1 hour at RT and then incubated with primary antibodies at 4°C overnight. The following primary antibodies were used: SDHA (D6J9M) XP Rabbit mAB (CST, USA), PARP (CST), NDUFA4 (ThermoFisher), IDO (Novus Biologicals, USA), C15orf48 (Aviva Systems Biology, USA), HA (Sigma), NDUFA4L2 (ThermoFisher), HIF-1α (Proteintech, USA), HDAC1 (Proteintech), TIM23 (Proteintech), citrate synthase (CS; Santa Cruz Biotechnology, USA), ATP5A (Santa Cruz Biotechnology), TOM70 (Abclonal, USA), VSV-M (Kerafast, USA), βIII-tubulin (ThermoFisher), and β-actin (Sigma). Membranes were washed 3 times with TBST and then incubated with secondary antibodies for 1 hour at RT. Goat Anti-Rabbit IgG (Bio-Rad, USA) and Goat Anti-Mouse IgG (Bio-Rad) were used as secondary antibodies. Membranes were washed 3 times with TBST and then incubated with Pierce ECL Plus Western Blotting Substrate (ThermoFisher). Blots were imaged using the ChemiDoc MP Imager (Bio-Rad).

### miRNA qPCR

Total RNA was extracted from cultured cells using the mirVana miRNA Isolation kit (Ambion, USA) following the manufacturer’s protocol. For each sample, 10 ng of total RNA was used as input for cDNA synthesis using the TaqMan Advanced miRNA cDNA Synthesis Kit (ThermoFisher). *hsa-miR-147b-3p* and *hsa-miR-210-3p* levels were assessed by TaqMan Advanced miRNA Assays (ThermoFisher) and TaqMan Fast Advanced miRNA master mix (ThermoFisher). *hsa-mir-423-5p* (ThermoFisher) served as the endogenous control for analysis of miRNA expression. PCR was run in an Applied Biosystems QuantStudio 7 Real-Time PCR instrument (ThermoFisher) following the manufacturer’s instructions.

### mRNA qPCR

Total RNA was extracted from cultured cells using the mirVana miRNA Isolation kit (Ambion) following the manufacturer’s protocol. Reverse transcription was performed on 1 μg of total RNA using the Maxima First Strand cDNA Synthesis Kit (ThermoFisher) as described above. *MISTRAV*, *MISTR1*, and *MISTRH* mRNA levels were assessed by a SYBR green qPCR assay using the PowerUp SYBR Green Master Mix (ThermoFisher) (primer sequences in S4 Table). The *18s rRNA* served as the endogenous control for analysis of mRNA expression. PCR was run in an Applied Biosystems QuantStudio 7 Real-Time PCR instrument following the manufacturer’s instructions.

### Cell viability assays

A549 cells were plated at 1 × 10^4^ cells/well in opaque white 96-well plates (Corning) in 100 μL of media. After 24 hours, spent medium was aspirated and replaced with 75 μL of fresh media supplemented with 1,000 U/mL IFNγ (ThermoFisher). Moreover, 24 hours following IFNγ addition, 25 μL of media containing staurosporine (Abcam) was added (final staurosporine treatment concentration: 1 μM). Cell viability was assessed 16 hours later using CellTiter-Glo (Promega) following the manufacturer’s instructions.

### IncuCyte analysis of caspase-3/7 activity

For experiments on the *MISTRAV* KO clones, 5 × 10^3^ cells were seeded and primed with IFNγ as above. Moreover, 24 hours post-IFNγ addition, 25 μL of media containing staurosporine and CellEvent Caspase-3/7 Green Detection Reagent (ThermoFisher) at final treatment concentrations of 1 μM and 2.5 μM, respectively, was added. For experiments on the *MISTR1* (*NDUFA4*) KO and the vMISTRAV cell lines, 5 × 10^3^ cells/well were plated in opaque white 96-well plates (Corning) in 75 μL of media. The next day, 25 μL of media containing the appropriate drug and caspase-3/7 detection reagent was added (*MISTR1* (*NDUFA4*) KO cell lines: 1 μM staurosporine, 2.5 μM CellEvent Caspase-3/7 Green Detection Reagent; EV and vMISTRAV cell lines: 1 μM staurosporine, 1 μg/mL actinomycin D, 1 μM camptothecin, and 5 μM IncuCyte Caspase-3/7 Red Apoptosis Assay Reagent). To determine the cell number at the initial treatment time point, 25 μL of media containing Vybrant DyeCycle Green Stain or SYTO 60 Red Fluorescent Nucleic Acid Stain (final concentration: 1 μM) was added to a set of wells for each cell line. Infection experiments were carried out as described in the “VSV infections and detection” section above. After inoculating the cells with virus for 1 hour and bringing the volume in each well up to 75 μL, 25 μL of media containing the appropriate caspase-3/7 detection reagent was added to each well. Plates were placed in an IncuCyte S3 Live-Cell Analysis System (Essen Bioscience, USA) with a 10× objective in a standard cell culture incubator at 37°C and 5% CO_2_. Four images/well were collected every 2 hours in phase contrast and fluorescence. The integrated object counting algorithm was used to count fluorescent objects/mm^2^ for each time point. Relative caspase-3/7 activity was determined by dividing the number of caspase-3/7 objects/mm^2^ at each time point by the number of cells/mm^2^ at the initial treatment time point multiplied by 100.

### Chemical hypoxia induction

A day after plating cells in 6-well plates or 10-cm dishes, chemical hypoxia was induced by treating cells with 300-μM deferoxamine mesylate. After 24 hours, cells were either collected in RIPA buffer or subjected to nuclear fractionation protocol as described above.

### miRNA and MRE analysis

Predicted MREs in MISTR1 (NDUFA4) were retrieved from Targetscan [43] and mirDB [41] (S2 Table). miRNA and MISTR1 (NDUFA4) sequences were retrieved from NCBI (S1 Text).

### Transfection of miRNAs and miRNA reporter luciferase assays

HEK293T cells were seeded at 1 × 10^4^ cells/well in opaque white 96-well plates (Corning) in 75 μL of media. The next day, cells were transfected with 50 ng/well of the psiCHECK-2 (Promega) construct using the FuGENE HD Transfection Reagent (Promega), following the manufacturer’s instructions. After 24 hours, cells were transfected with 1 pmol/well of miRNA mimics (ThermoFisher) using Lipofectamine RNAiMAX Transfection Reagent (ThermoFisher) according to manufacturer’s instructions. The following miRNA mimics were used: *hsa-miR-210-3p*, *hsa-miR-7-5p*, *hsa-miR-202-5p*, *hsa-miR-145-5p*, *hsa-miR-205-5p*, *hsa-miR-147b-3p*, and Negative Control #1 (ThermoFisher). Moreover, 48 hours after miRNA transfection, firefly and *Renilla* luciferase activities were measured using the Dual-glo Luciferase assay (Promega).

### Constructs

hMISTRAV-FLAG and vMISTRAV-HA vectors were generated as follows. Briefly, hMISTRAV and vMISTRAV reference sequences were synthesized as gBlocks (IDT) with carboxyl-terminal epitope-tags and KpnI/PmeI sites. The gBlocks were cloned into pcDNA6/myc-His B (Invitrogen, USA) using KpnI/PmeI sites. Clones were confirmed by Sanger sequencing.

### Confocal images

A549 WT cells were plated at 3 × 10^3^ cells/well in an 8-well chambered cover glass. The next day, 250 ng of plasmid DNA (hMISTRAV-FLAG, vMISTRAV-HA) was mixed with Opti-MEM and FuGENE HD Transfection reagent at a ratio of 3:1 FuGENE HD:plasmid DNA. After incubating for 5 minutes, complexes were added dropwise to the cells and left to incubate for 48 hours. At the time of collection, media was removed and replaced with fresh media containing 250 nM of MitoTracker Deep Red FM to stain mitochondria. Following a 30-minute incubation, media was removed, and cells were washed once with 1X PBS before fixation with 4% paraformaldehyde for 10 minutes. Paraformaldehyde was removed, and cells were washed twice with 1X PBS for 5 minutes each. Following washes, cells were permeabilized with 0.1% Triton X-100 in 1X PBS for 10 minutes rocking at room temperature. Next, cells were washed twice with 0.05% Tween-20 in 1X PBS for 5 minutes at room temperature. Once the last wash was complete, blocking buffer (1% BSA, 0.05% Tween-20, 1X PBS) was added and incubated for an hour at room temperature. Following incubation, primary antibody (Anti-FLAG or Anti-HA) was diluted according to manufacturer’s instructions in blocking buffer and added to cells to incubate overnight at 4°C. The next day, cells were subjected to three 5-minute washes in 0.05% Tween-20 in 1X PBS. The appropriate secondary antibodies were diluted 1:500 in blocking buffer and added to cells to incubate for 1 hour at room temperature in dark. Cells were washed 3 times in 0.05% Tween-20 in 1X PBS for 5 minutes each, where DAPI stain was added to the last 5 minute wash to stain the nuclei. After washes, fresh PBS was added to the cells. Cells were imaged on an Olympus Fluoview FV10i-LIV, capturing multiple Z-stacks using the 60× objective (1 representative image shown). Images were processed to final form in the associated Olympus image analysis program.

### Protein modeling

A recently published predicted structure of Complex IV (PDB:5Z62) [33], which contains MISTR1 (NDUFA4), was used for modeling. The structures of MISTR paralogs (MISTRAV and MISTRH) were predicted using Swiss-Model [32]. UCSF Chimera (https://www.cgl.ucsf.edu/chimera/) [94] was used for visualization, mapping rapidly evolving sites, and analysis.

### Statistical analysis

Experimental data are presented at means ± SD. Statistical significance was determined by 2-tailed unpaired Student *t* test. GraphPad Prism software (version 8.3.0) was used for statistical analysis.

## Supporting information

S1 Fig Sequence analysis of TetV-1 MISTR (vMISTRA). **A)** Clustal omega amino acid alignment of TetV-1 MISTR with 3 Tetraselmis MISTR protein sequences from the database. **B)** blastp analysis of TetV-1 MISTR—Query—with Tetraselmis MISTR (A0A061RM32)—Subject. MISTR, MItochondrial STress Response; TetV-1, Tetraselmis virus 1; vMISTRA, viral MISTR Algae.
(TIF)

S2 Fig MISTR factors are conserved in vertebrates. Clustal omega amino acid alignment of MISTRAV, MISTR1 (NDUFA4), and MISTRH sequences. Hs, *Homo sapiens* (Human); Mm, *Mus musculus* (mouse); Dr, *Danio rerio* (zebrafish); Lo, *Lepisosteus oculatus* (spotted gar). Accession numbers are for NCBI. MISTR, MItochondrial STress Response; MISTRAV, MItochondrial STress Response AntiViral; MISTRH, MItochondrial STress Response Hypoxia; NDUFA4, NADH dehydrogenase ubiquinone 1 alpha subcomplex subunit 4.
(TIF)

S3 Fig Characterization of *MISTRAV* KO A549 cells. **A)** Proliferation rates of A549 *MISTRAV* KO clonal lines measured using IncuCyte. Changes in % confluence were used as a surrogate marker of cell proliferation. Data represent means ± SD (*n* = 6 replicates). **B)** CellTiter-Glo (luciferase-based) cell viability assay on WT and *MISTRAV* KO cells treated with IFNγ, STS, or both for 16 hours. Data represent means ± SD (*n* = 3 replicates). Statistical significance was determined by a 2-tailed unpaired *t* test, *p ≤ 0.05, **p ≤ 0.01, ***p ≤ 0.001. **C)** Phase contrast images of A549 WT and *MISTRAV* KO cells 18 hours postinfection with VSV-LUC. One set of cells were pretreated with IFNγ 24 hours prior to infection. **D)** A549 WT and *MISTRAV* KO cells were infected with VSV-LUC at an MOI of 0.01. Viral replication was assessed 18 hours postinfection using the Bright-Glo Luciferase Assay System. Data represent means ± SD (*n* = 6 replicates). The underlying data for panels A, B, and D can be found in S1 Data. IFNγ, interferon gamma; KO, knockout; MISTRAV, MItochondrial STress Response AntiViral; MOI, multiplicity of infection; STS, staurosporine; VSV-LUC, vesicular stomatitis virus-luciferase; WT, wild-type.
(TIF)

S4 Fig Zebrafish lack intact *miR-147b*. Clustal omega nucleotide alignment of *MISTRAV* 3′ UTR sequences. Alignment starts with MISTRAV stop codon. Predicted *pre-mir-147b* (blue) relative to human annotation, predicted *miR-147b* (red). Hs, *Homo sapiens* (Human); Mm, *Mus musculus* (mouse); Dr, *Danio rerio* (zebrafish); Lo, *Lepisosteus oculatus* (spotted gar). Accession numbers are for NCBI. MISTRAV, MItochondrial STress Response AntiViral.
(TIF)

S5 Fig Generation and characterization of *MISTR1* (*NDUFA4*) KO A549 cells. **A)** CRISPR/Cas deletion strategy for *MISTR1* (*NDUFA4*). Scissors indicate relative locations of gRNAs designed to target sequences flanking exon 2 of this gene. The exon 2 deletion strategy was employed for ease of genotyping. Gene structure from UCSC genome browser. Sequences of breakpoints identified a 225-bp deletion that included exon 2. Note that identical repaired breakpoints were recovered for both clones. **B)** Agarose gel resolving amplicons from genotyping PCR of A549 KO clones. **C)** Western blot analysis using lysates from WT and *MISTR1* (*NDUFA4*) KO clones. **D)** Measurement of proliferation rates using IncuCyte for *MISTR1* (*NDUFA4*) KO A549 cell line. Changes in % confluence were used as a surrogate marker of cell proliferation. Data represent means ± SD (*n* = 6 replicates). **E)** Western blot analysis of cleaved PARP levels using lysates from WT and *MISTR1* (*NDUFA4*) KO cells following 16 hours of STS treatment, or **F)** 22 hours postinfection with VSV-LUC. Densitometry analysis of PARP levels was performed using Image Lab version 6.0.1 (Bio-Rad). % Cleaved PARP = (cleaved PARP/(Full + Cleaved PARP)) * 100. **G)** A549 WT and *MISTR1 (NDUFA4)* KO cells were infected with VSV-LUC at an MOI of 0.01. Viral replication was assessed 18 hours postinfection using the Bright-Glo Luciferase Assay System. Data represent means ± SD (*n* = 6 replicates). The underlying data for panels D–G can be found in S1 Data. gRNA, guide RNA; KO, knockout; MOI, multiplicity of infection; STS, staurosporine; VSV-LUC, vesicular stomatitis virus-luciferase; WT, wild-type.
(TIF)

S6 Fig Phylogenetic analysis of 157 MISTR amino acid sequences. An inferred tree built using 157 MISTR amino acid sequences by maximum-likelihood analysis using PhyML [50] (http://www.atgc-montpellier.fr/phyml/) with the VT+G model as selected by SMS and 100 bootstrap replicates. Sequences were extracted from the NCBI sequence database, Uniprot (https://www.uniprot.org/) and [14] (S3 Table, S2 Text). Bootstrap percentages from the analysis greater than 50 are indicated by asterisks. Scale for amino acid substitutions per site—bottom. MISTR, MItochondrial STress Response; SMS, Smart Model Selection.
(TIF)

S7 Fig Phylogenetic analysis of 185 MISTR amino acid sequences. An inferred tree built using 185 MISTR amino acid sequences by maximum-likelihood analysis using PhyML [50] (http://www.atgc-montpellier.fr/phyml/) with the VT +G model as selected by SMS and 100 bootstrap replicates. Sequences were extracted from the NCBI sequence database, Uniprot (https://www.uniprot.org/) and [14] (S3 Table, S2 Text). Bootstrap percentages from the analysis greater than 50 are indicated by asterisks. Scale for amino acid substitutions per site—bottom. MISTR, MItochondrial STress Response; SMS, Smart Model Selection.
(TIF)

S8 Fig Characterization of WT A549 cells stably expressing vMISTRAV. **A)** Proliferation rates of EV and vMISTRAV expressing cells measured using IncuCyte. Changes in % confluence were used as a surrogate marker of cell proliferation. Data represent means ± SD (*n* = 6 replicates). **B)** Western blot analysis of cleaved PARP levels using lysates from EV and vMISTRAV expressing cells following treatment with activators of apoptosis. Lysates were collected 16 hours after treatment with STS or ActD and 24 hours after treatment with CPT. **C)** Western blot analysis of cleaved PARP levels using lysates from EV and vMISTRAV-expressing cells 18 hours postinfection with VSV-LUC. Densitometry analysis of PARP levels was performed using Image Lab version 6.0.1 (Bio-Rad). % Cleaved PARP = (cleaved PARP/(Full + Cleaved PARP)) * 100. **D)** EV and vMISTRAV-expressing cells were infected with VSV-LUC at an MOI of 0.01. Viral replication was assessed 18 hours postinfection using the Bright-Glo Luciferase Assay System. Data represent means ± SD (*n* = 6 replicates). The underlying data for panels A–D can be found in S1 Data. ActD, actinomycin D; CPT, camptothecin; EV, empty vector; MOI, multiplicity of infection; STS, staurosporine; VSV-LUC, vesicular stomatitis virus-luciferase; WT, wild-type.
(TIF)

S1 Table Nucleotide sequence information for rapid evolution analysis. (XLSX)

S2 Table *miR-147b* target prediction output from miRDB and TargetScan. (XLSX)

S3 Table Sequence information for evolutionary analysis of MISTR homologs. (XLSX)

S4 Table Primers and oligos used in this study. (XLSX)

S5 Table Key resources table. (XLSX)

S1 Text Blastp analysis and output of *vMISTRAV* (related to Fig 1), summary of *MISTRAV*, *MISTR1*, and *MISTRH* PAML NSsites analysis (related to Fig 2), and input sequences used for evolutionary analysis in Figs 2 and 4. MISTRAV, MItochondrial STress Response AntiViral; MISTRH, MItochondrial STress Response Hypoxia.
(DOC)

S2 Text Amino acid alignments used for phylogenetic analysis of MISTR sequences in Fig 7 and S6 and S7 Figs. MISTR, MItochondrial STress Response. (TXT)

S1 Raw Images Original, uncropped images supporting blot and gel results reported in Figs 1E, 1G–1I, 3B, 3C, 3E, 3H, 5C, 5J, 6C and 6D and S5B, S5C, S5E, S5F, S8B and S8C Figs. (PDF)

S1 Data Excel spreadsheet containing the numerical data presented in Figs 3C–3H, 5B, 5D–5I, 6E, 6F and 8A–8D and S3A, S3B, S3D, S3D–S3G and S8A–S8D Figs. (XLSX)

## Abbreviations

AA: amino acid
ccRCC: clear cell Renal Cell Carcinoma
CS: citrate synthase
dsDNA: double-stranded DNA
ETC: electron transport chain
EV: empty vector
FL: full-length
gRNA: guide RNA
HBV: hepatitis B virus
HCV: hepatitis C virus
HGT: horizontal gene transfer
HIF-1α: hypoxia-inducible factor 1 alpha
HOM: Hominoids
IFNα: interferon alpha
IFNγ: interferon gamma
IL: interleukin
IMM: inner mitochondrial membrane
IMS: intermembrane space
ISG: interferon-stimulated gene
miRNA: microRNA
MISTR: MItochondrial STress Response
MISTRAV: MItochondrial STress Response AntiViral
MISTRH: MItochondrial STress Response Hypoxia
MOI: multiplicity of infection
MRE: microRNA response element
MSA: multiple sequence alignment
NDUFA4: NADH dehydrogenase ubiquinone 1 alpha subcomplex subunit 4
NDUFA4L2: NADH dehydrogenase ubiquinone 1 alpha subcomplex subunit 4 like-2
*NMES1*: *normal mucosal esophageal-specific gene product 1*
nt: nucleotides
NWM: New World monkey
OAS1: oligoadenylate synthetase 1
OWM: Old World monkey
OXPHOS: oxidative phosphorylation
qPCR: quantitative PCR
QTL: quantitative trait locus
RT-PCR: reverse transcription PCR
SMS: Smart Model Selection
TCA cycle: tricarboxylic acid cycle
TetV-1: Tetraselmis virus 1
TMEM: transmembrane
TNF: tumor necrosis factor
VHL: Von Hippel–Lindau
vMISTRA: viral MISTR Algae
vMISTRAV: viral MISTRAV
VSV: vesicular stomatitis virus
WT: wild-type
