# Supplementary figures and images for: Signatures of host–pathogen evolutionary conflict reveal MISTR—A conserved MItochondrial STress Response network
Source: PLoS Biol. 2020 Dec 28;18(12):e3001045. doi: 10.1371/journal.pbio.3001045 (PMC7793259; doi:10.1371/journal.pbio.3001045)

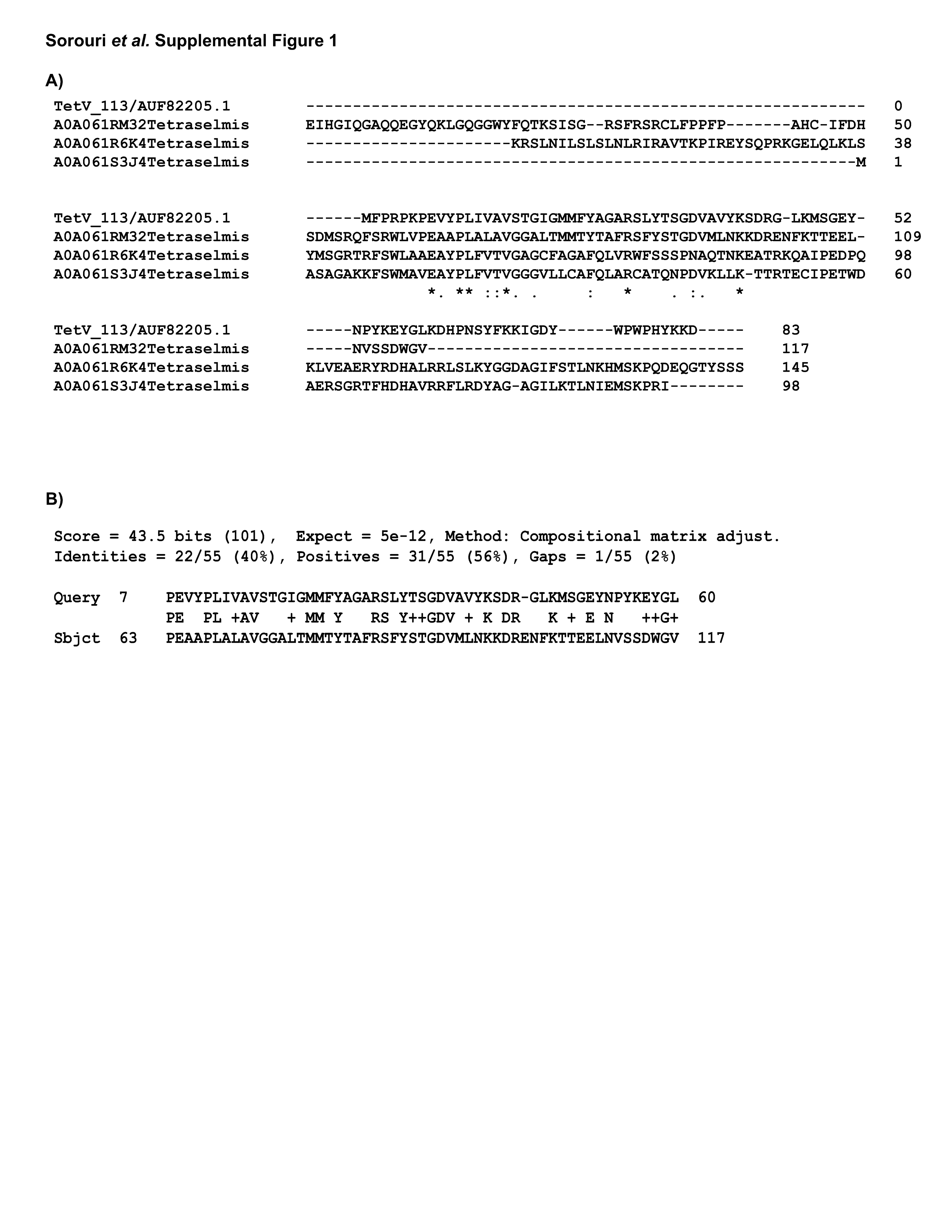

Supplement: S1 Fig — A) Clustal omega amino acid alignment of TetV-1 MISTR with 3 Tetraselmis MISTR protein sequences from the database. B) blastp analysis of TetV-1 MISTR—Query—with Tetraselmis MISTR (A0A061RM32)—Subject. MISTR, MItochondrial STress Response; TetV-1, Tetraselmis virus 1; vMISTRA, viral MISTR Algae. (TIF) [file pbio.3001045.s001.tif]

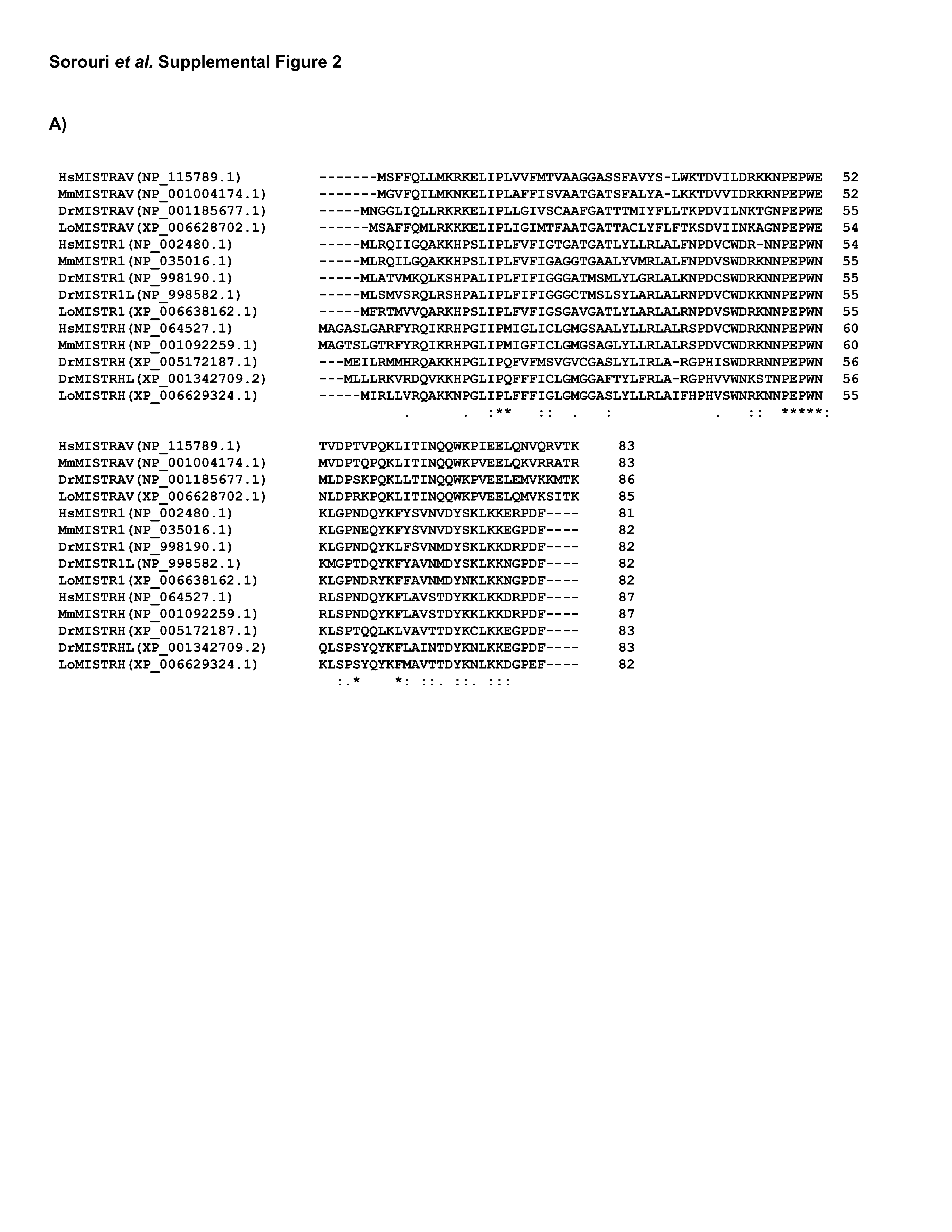

Supplement: S2 Fig — Clustal omega amino acid alignment of MISTRAV, MISTR1 (NDUFA4), and MISTRH sequences. Hs, Homo sapiens (Human); Mm, Mus musculus (mouse); Dr, Danio rerio (zebrafish); Lo, Lepisosteus oculatus (spotted gar). Accession numbers are for NCBI. MISTR, MItochondrial STress Response; MISTRAV, MItochondrial STress Response AntiViral; MISTRH, MItochondrial STress Response Hypoxia; NDUFA4, NADH dehydrogenase ubiquinone 1 alpha subcomplex subunit 4. (TIF) [file pbio.3001045.s002.tif]

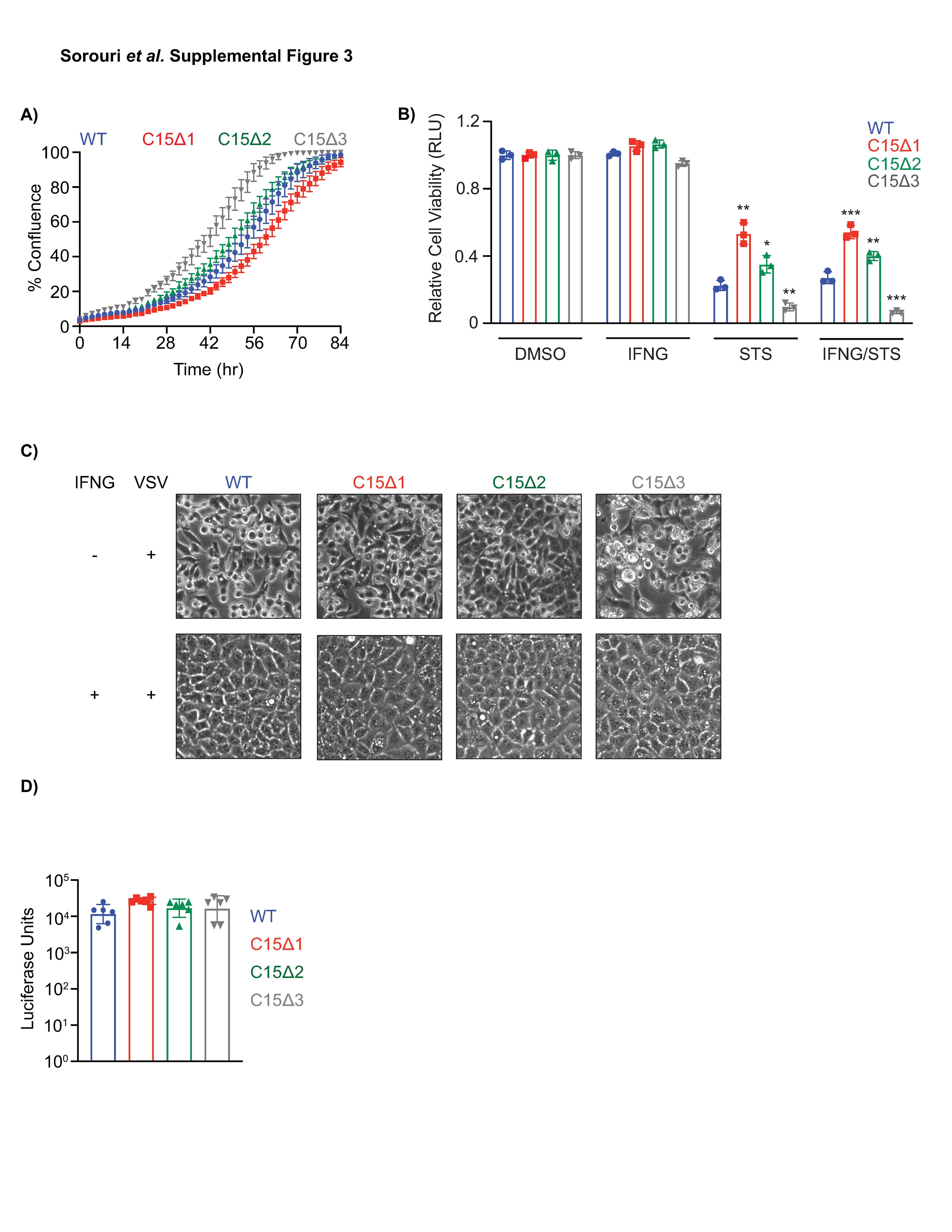

Supplement: S3 Fig — A) Proliferation rates of A549 MISTRAV KO clonal lines measured using IncuCyte. Changes in % confluence were used as a surrogate marker of cell proliferation. Data represent means ± SD (n = 6 replicates). B) CellTiter-Glo (luciferase-based) cell viability assay on WT and MISTRAV KO cells treated with IFNγ, STS, or both for 16 hours. Data represent means ± SD (n = 3 replicates). Statistical significance was determined by a 2-tailed unpaired t test, *p ≤ 0.05, **p ≤ 0.01, ***p ≤ 0.001. C) Phase contrast images of A549 WT and MISTRAV KO cells 18 hours postinfection with VSV-LUC. One set of cells were pretreated with IFNγ 24 hours prior to infection. D) A549 WT and MISTRAV KO cells were infected with VSV-LUC at an MOI of 0.01. Viral replication was assessed 18 hours postinfection using the Bright-Glo Luciferase Assay System. Data represent means ± SD (n = 6 replicates). The underlying data for panels A, B, and D can be found in S1 Data. IFNγ, interferon gamma; KO, knockout; MISTRAV, MItochondrial STress Response AntiViral; MOI, multiplicity of infection; STS, staurosporine; VSV-LUC, vesicular stomatitis virus-luciferase; WT, wild-type. (TIF) [file pbio.3001045.s003.tif]

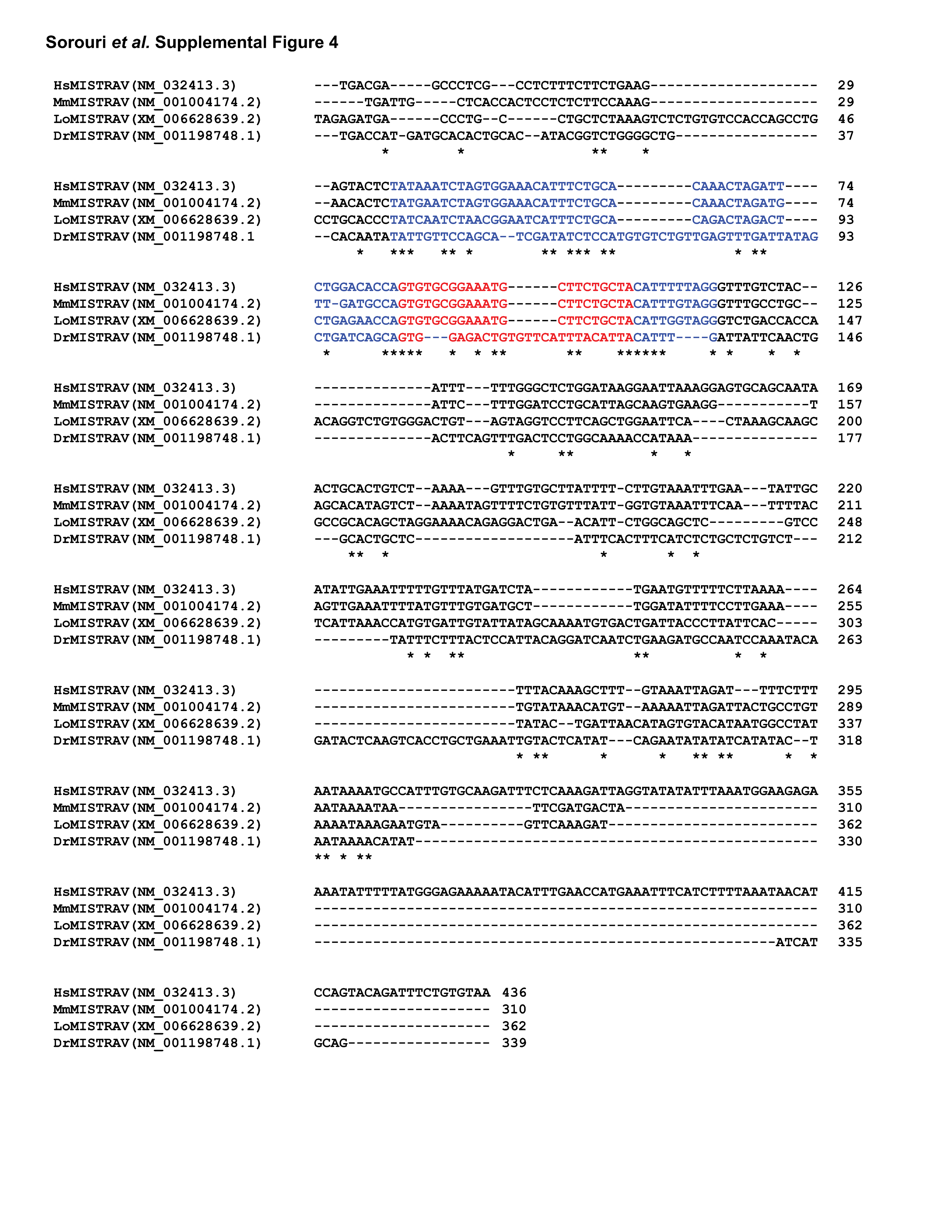

Supplement: S4 Fig — Clustal omega nucleotide alignment of MISTRAV 3′ UTR sequences. Alignment starts with MISTRAV stop codon. Predicted pre-mir-147b (blue) relative to human annotation, predicted miR-147b (red). Hs, Homo sapiens (Human); Mm, Mus musculus (mouse); Dr, Danio rerio (zebrafish); Lo, Lepisosteus oculatus (spotted gar). Accession numbers are for NCBI. MISTRAV, MItochondrial STress Response AntiViral. (TIF) [file pbio.3001045.s004.tif]

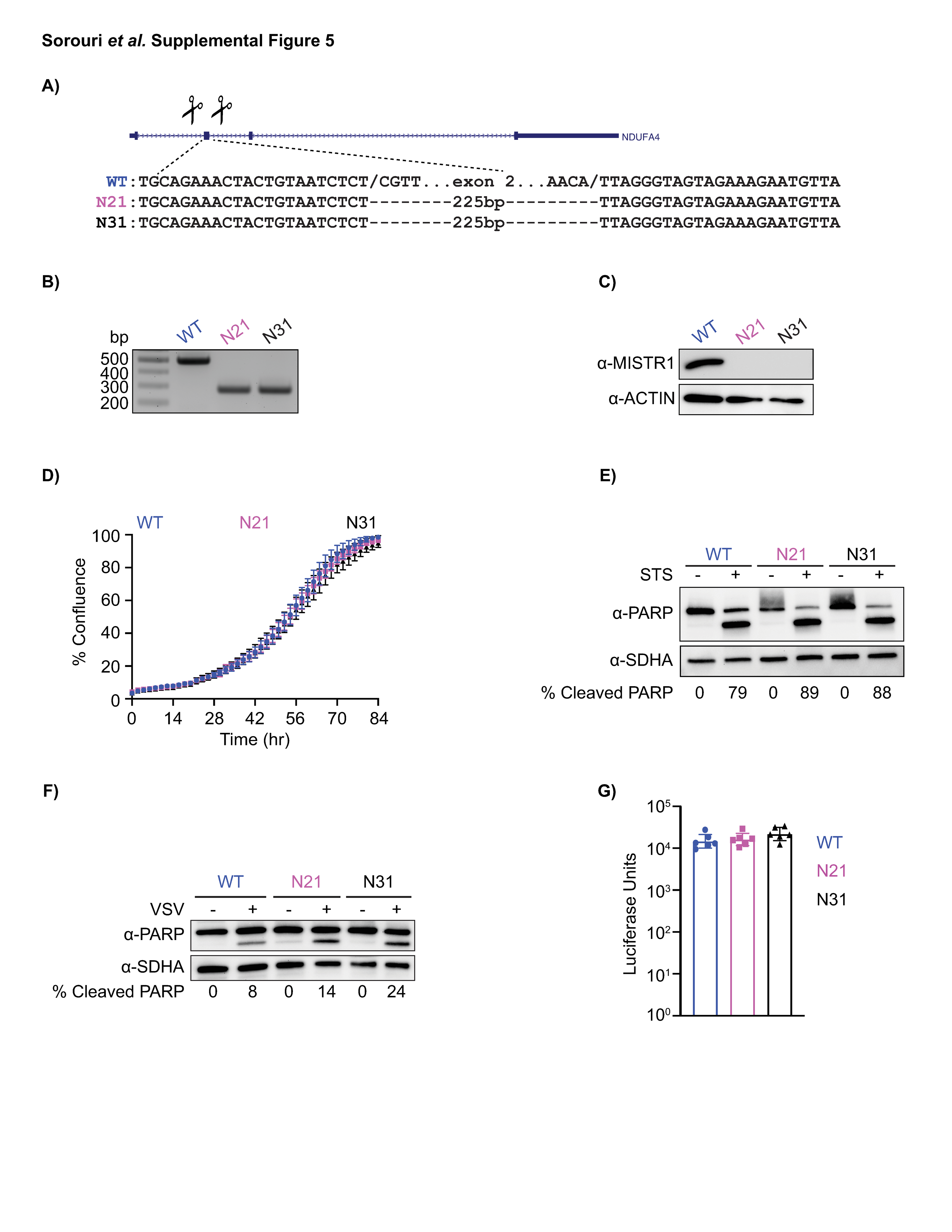

Supplement: S5 Fig — A) CRISPR/Cas deletion strategy for MISTR1 (NDUFA4). Scissors indicate relative locations of gRNAs designed to target sequences flanking exon 2 of this gene. The exon 2 deletion strategy was employed for ease of genotyping. Gene structure from UCSC genome browser. Sequences of breakpoints identified a 225-bp deletion that included exon 2. Note that identical repaired breakpoints were recovered for both clones. B) Agarose gel resolving amplicons from genotyping PCR of A549 KO clones. C) Western blot analysis using lysates from WT and MISTR1 (NDUFA4) KO clones. D) Measurement of proliferation rates using IncuCyte for MISTR1 (NDUFA4) KO A549 cell line. Changes in % confluence were used as a surrogate marker of cell proliferation. Data represent means ± SD (n = 6 replicates). E) Western blot analysis of cleaved PARP levels using lysates from WT and MISTR1 (NDUFA4) KO cells following 16 hours of STS treatment, or F) 22 hours postinfection with VSV-LUC. Densitometry analysis of PARP levels was performed using Image Lab version 6.0.1 (Bio-Rad). % Cleaved PARP = (cleaved PARP/(Full + Cleaved PARP)) * 100. G) A549 WT and MISTR1 (NDUFA4) KO cells were infected with VSV-LUC at an MOI of 0.01. Viral replication was assessed 18 hours postinfection using the Bright-Glo Luciferase Assay System. Data represent means ± SD (n = 6 replicates). The underlying data for panels D–G can be found in S1 Data. gRNA, guide RNA; KO, knockout; MOI, multiplicity of infection; STS, staurosporine; VSV-LUC, vesicular stomatitis virus-luciferase; WT, wild-type. (TIF) [file pbio.3001045.s005.tif]

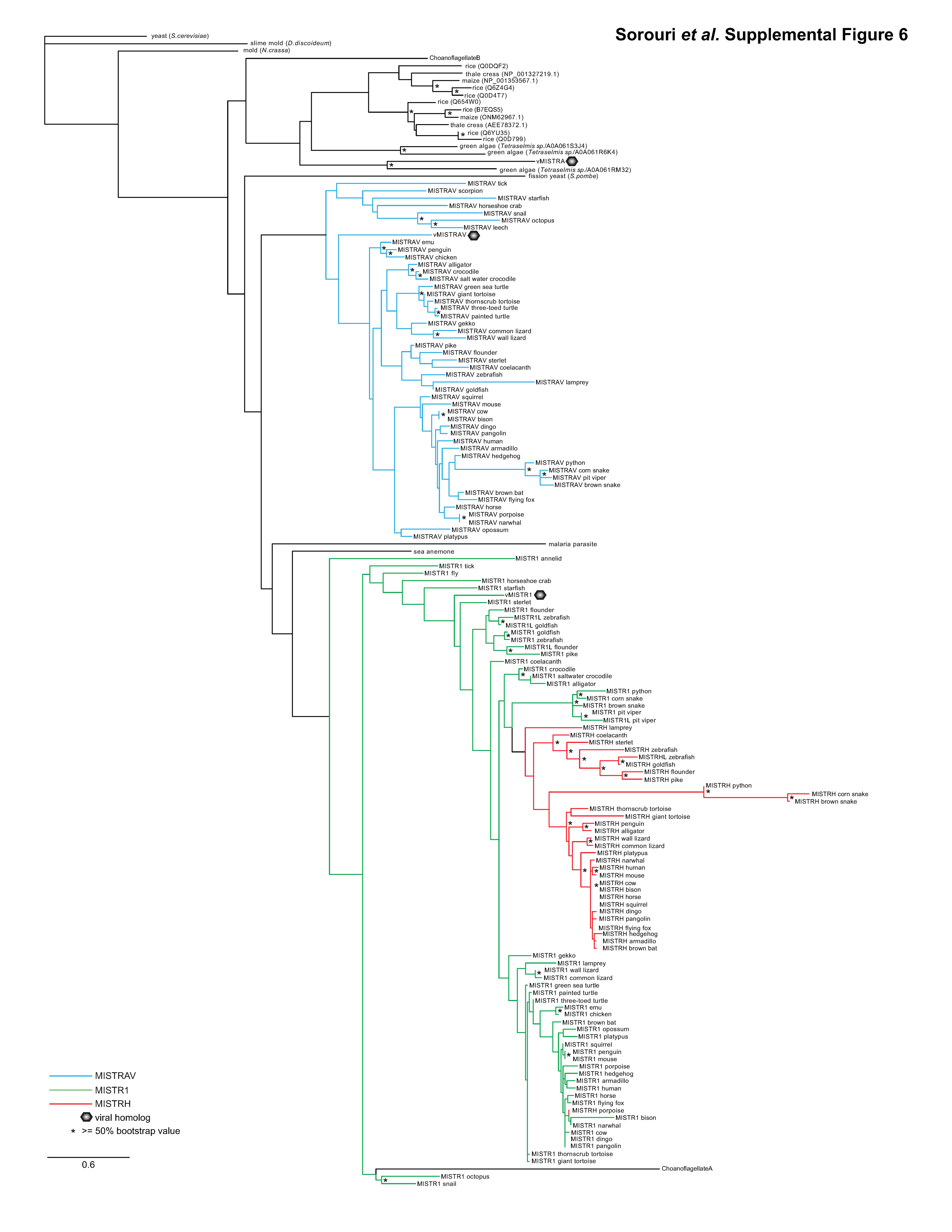

Supplement: S6 Fig — An inferred tree built using 157 MISTR amino acid sequences by maximum-likelihood analysis using PhyML [50] (http://www.atgc-montpellier.fr/phyml/) with the VT+G model as selected by SMS and 100 bootstrap replicates. Sequences were extracted from the NCBI sequence database, Uniprot (https://www.uniprot.org/) and [14] (S3 Table, S2 Text). Bootstrap percentages from the analysis greater than 50 are indicated by asterisks. Scale for amino acid substitutions per site—bottom. MISTR, MItochondrial STress Response; SMS, Smart Model Selection. (TIF) [file pbio.3001045.s006.tif]

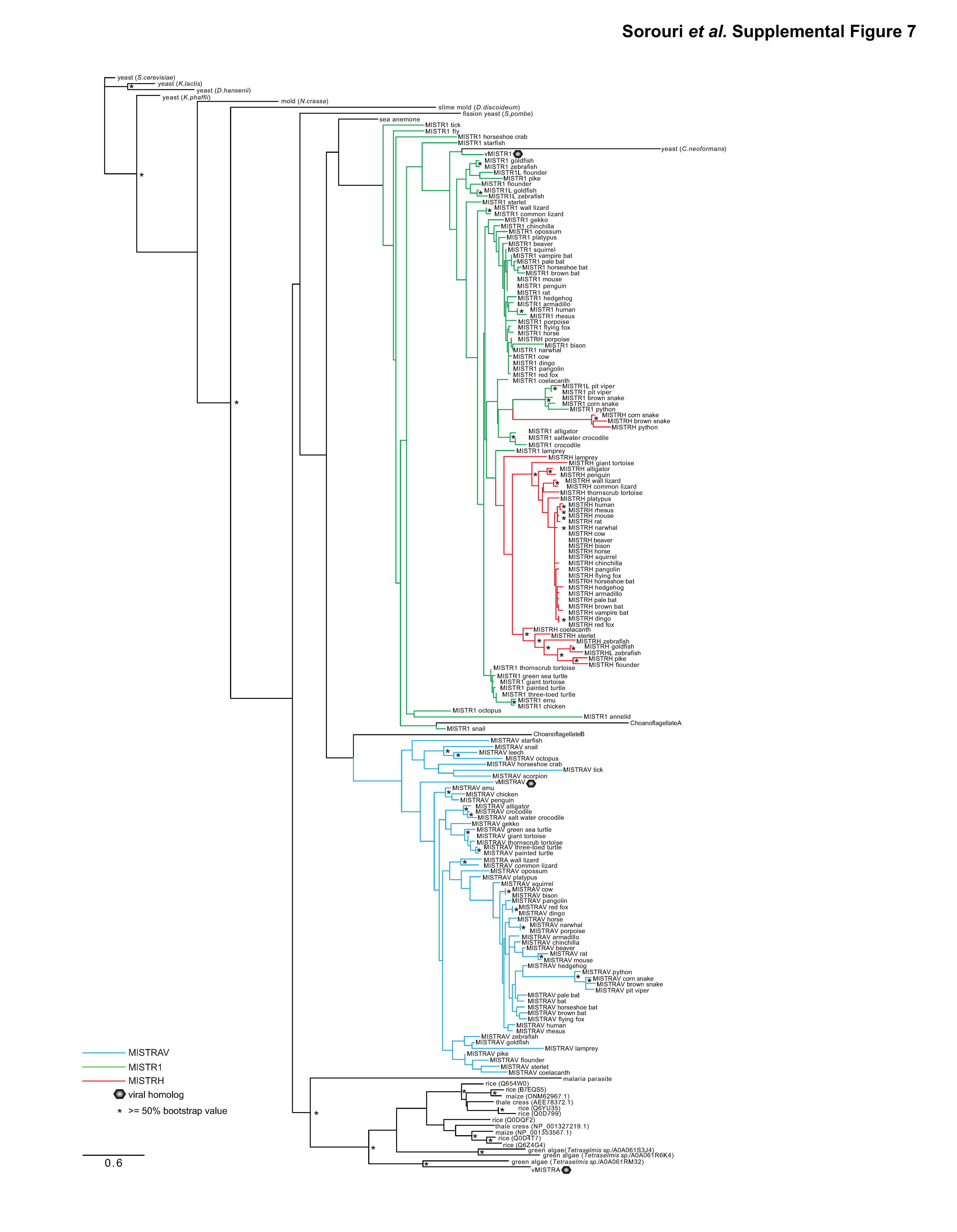

Supplement: S7 Fig — An inferred tree built using 185 MISTR amino acid sequences by maximum-likelihood analysis using PhyML [50] (http://www.atgc-montpellier.fr/phyml/) with the VT +G model as selected by SMS and 100 bootstrap replicates. Sequences were extracted from the NCBI sequence database, Uniprot (https://www.uniprot.org/) and [14] (S3 Table, S2 Text). Bootstrap percentages from the analysis greater than 50 are indicated by asterisks. Scale for amino acid substitutions per site—bottom. MISTR, MItochondrial STress Response; SMS, Smart Model Selection. (TIF) [file pbio.3001045.s007.tif]

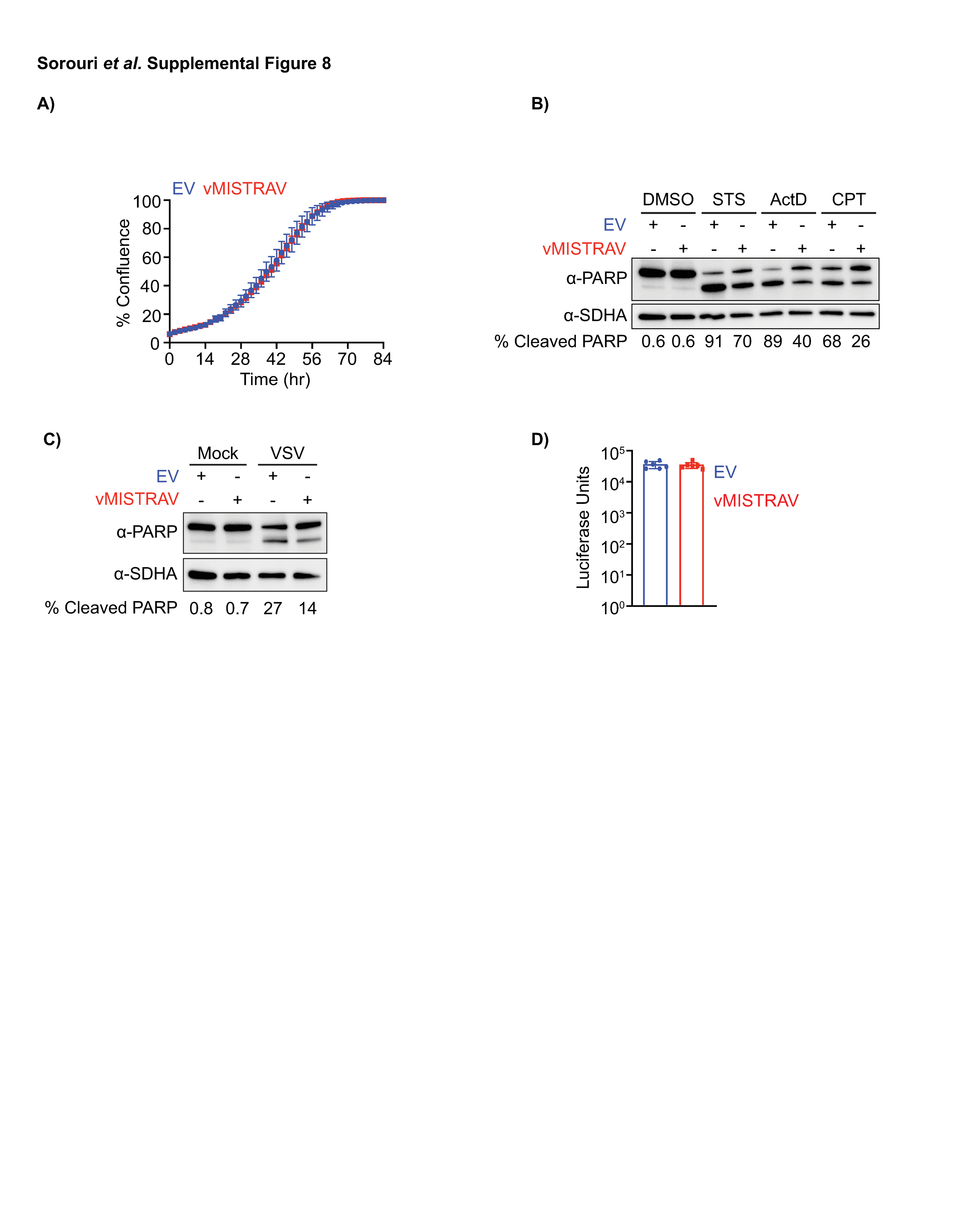

Supplement: S8 Fig — A) Proliferation rates of EV and vMISTRAV expressing cells measured using IncuCyte. Changes in % confluence were used as a surrogate marker of cell proliferation. Data represent means ± SD (n = 6 replicates). B) Western blot analysis of cleaved PARP levels using lysates from EV and vMISTRAV expressing cells following treatment with activators of apoptosis. Lysates were collected 16 hours after treatment with STS or ActD and 24 hours after treatment with CPT. C) Western blot analysis of cleaved PARP levels using lysates from EV and vMISTRAV-expressing cells 18 hours postinfection with VSV-LUC. Densitometry analysis of PARP levels was performed using Image Lab version 6.0.1 (Bio-Rad). % Cleaved PARP = (cleaved PARP/(Full + Cleaved PARP)) * 100. D) EV and vMISTRAV-expressing cells were infected with VSV-LUC at an MOI of 0.01. Viral replication was assessed 18 hours postinfection using the Bright-Glo Luciferase Assay System. Data represent means ± SD (n = 6 replicates). The underlying data for panels A–D can be found in S1 Data. ActD, actinomycin D; CPT, camptothecin; EV, empty vector; MOI, multiplicity of infection; STS, staurosporine; VSV-LUC, vesicular stomatitis virus-luciferase; WT, wild-type. (TIF) [file pbio.3001045.s008.tif]
