# Supplementary material for: Signatures of host–pathogen evolutionary conflict reveal MISTR—A conserved MItochondrial STress Response network
Source: PLoS Biol. 2020 Dec 28;18(12):e3001045. doi: 10.1371/journal.pbio.3001045 (PMC7793259; doi:10.1371/journal.pbio.3001045)
Supplement: S1 Text — MISTRAV, MItochondrial STress Response AntiViral; MISTRH, MItochondrial STress Response Hypoxia. (DOC) [file pbio.3001045.s014.doc]

**Sorouri *et al*. S1_Text**

This file contains:

1. blastp analysis and output of *vMISTRAV*, related to Figure 1.
2. Summary of *MISTRAV* PAML NSsites analysis, related to Figure 2
3. Summary of *MISTR1* PAML NSsites analysis, related to Figure 2
4. Summary of *MISTRH* PAML NSsites analysis, related to Figure 2
5. Primate *MISTRAV*/*C15ORF48* input sequences for evolutionary analysis, related to Figure 2.
6. Primate *MISTRAV*/*C15ORF48* input sequences lacking predicted TMEM for evolutionary analysis, related to Figure 2.
7. Primate *MISTR1*/*NDUFA4* input sequences for evolutionary analysis, related to Figure 2.
8. Primate *MISTR1*/*NDUFA4* input sequences lacking predicted TMEM for evolutionary analysis, related to Figure 2.
9. Primate *MISTRH*/*NDUFA4L2* input sequences for evolutionary analysis, related to Figure 2.
10. Sequences used for *miR-147b* evolutionary analysis, related to Figure 4
11. Sequences used for *miR-210* evolutionary analysis, related to Figure 4
12. Sequences used for *MISTR1*/*NDUFA4* MRE evolutionary analysis, related to Figure 4

**1) blastp analysis and output of *vMISTRAV*, related to Figure 1.**

Reciprocal blastp analysis indicates *vMISTRAV* was presumably acquired by horizontal gene transfer derived from an ortholog of *MISTRAV*. Specifically, using vMISTRAV AA sequence as a query exclusively returns numerous host MISTRAV sequences – and not sequences of MISTRAV paralogs - from diverse species with the top 100 hits displaying amino acid identity ranging from 41-54% and E-values 1x10^-16^ to 1X10^-20^ with a range of query coverage (78-91%)(Supplemental Text). Consistently, domain analysis indicates vMISTRAV has a similar primary structure to host MISTRAV: short N-terminus, single-pass TMEM domain, longer C-terminus, and a B12D domain spanning these features (Figure 1B).

Database: All non-redundant GenBank CDS

translations+PDB+SwissProt+PIR+PRF excluding environmental samples

from WGS projects

154,440,456 sequences; 56,579,877,846 total letters

Query=

Length=91

Score Sequences producing significant alignments: (Bits) Value

XP_024423805.1 normal mucosa of esophagus-specific gene 1 pro... 87.8 8e-21

XP_009961649.1 PREDICTED: normal mucosa of esophagus-specific... 86.7 2e-20

XP_004578169.1 PREDICTED: normal mucosa of esophagus-specific... 84.3 2e-19

XP_009979561.1 PREDICTED: normal mucosa of esophagus-specific... 83.6 4e-19

XP_003791849.1 normal mucosa of esophagus-specific gene 1 pro... 83.2 5e-19

XP_015728419.1 PREDICTED: normal mucosa of esophagus-specific... 83.2 5e-19

XP_009490219.1 PREDICTED: normal mucosa of esophagus-specific... 82.8 8e-19

XP_009468393.1 PREDICTED: normal mucosa of esophagus-specific... 82.4 1e-18

XP_005019536.1 normal mucosa of esophagus-specific gene 1 pro... 82.4 1e-18

XP_009642320.1 PREDICTED: normal mucosa of esophagus-specific... 82.0 1e-18

XP_007933412.1 PREDICTED: normal mucosa of esophagus-specific... 82.0 1e-18

XP_009574734.1 PREDICTED: normal mucosa of esophagus-specific... 81.6 2e-18

XP_009707919.1 PREDICTED: normal mucosa of esophagus-specific... 81.6 2e-18

XP_009884434.1 PREDICTED: normal mucosa of esophagus-specific... 81.3 3e-18

XP_009811572.1 PREDICTED: normal mucosa of esophagus-specific... 81.3 3e-18

XP_010300338.1 PREDICTED: normal mucosa of esophagus-specific... 81.3 3e-18

XP_003756148.1 normal mucosa of esophagus-specific gene 1 pro... 81.3 3e-18

NP_001181937.1 normal mucosa of esophagus-specific gene 1 pro... 80.9 3e-18

XP_008682218.1 PREDICTED: normal mucosa of esophagus-specific... 80.9 3e-18

XP_010186034.1 PREDICTED: normal mucosa of esophagus-specific... 81.3 3e-18

XP_006981453.1 PREDICTED: normal mucosa of esophagus-specific... 80.9 4e-18

NP_001004174.1 normal mucosa of esophagus-specific gene 1 pro... 80.9 4e-18

XP_009864334.1 PREDICTED: normal mucosa of esophagus-specific... 80.9 5e-18

XP_003928954.1 PREDICTED: normal mucosa of esophagus-specific... 80.5 6e-18

XP_009915140.1 PREDICTED: normal mucosa of esophagus-specific... 80.1 7e-18

XP_005436326.1 PREDICTED: normal mucosa of esophagus-specific... 80.1 7e-18

XP_008583327.1 PREDICTED: normal mucosa of esophagus-specific... 80.1 8e-18

XP_013032709.1 PREDICTED: normal mucosa of esophagus-specific... 80.1 9e-18

NP_001008518.1 normal mucosa of esophagus-specific gene 1 pro... 80.1 9e-18

XP_004660917.1 PREDICTED: normal mucosa of esophagus-specific... 79.7 1e-17

XP_005241558.1 PREDICTED: normal mucosa of esophagus-specific... 79.7 1e-17

XP_009512516.1 PREDICTED: normal mucosa of esophagus-specific... 79.7 1e-17

XP_003209505.1 PREDICTED: normal mucosa of esophagus-specific... 79.7 1e-17

XP_012504650.1 PREDICTED: normal mucosa of esophagus-specific... 79.3 1e-17

XP_009274782.1 PREDICTED: normal mucosa of esophagus-specific... 79.7 1e-17

XP_011353361.1 normal mucosa of esophagus-specific gene 1 pro... 79.3 1e-17

XP_010208579.1 PREDICTED: normal mucosa of esophagus-specific... 79.7 1e-17

XP_006920466.1 normal mucosa of esophagus-specific gene 1 pro... 79.3 1e-17

XP_017400911.1 PREDICTED: normal mucosa of esophagus-specific... 79.3 2e-17

XP_007539976.1 PREDICTED: normal mucosa of esophagus-specific... 79.3 2e-17

XP_004461217.1 normal mucosa of esophagus-specific gene 1 pro... 79.3 2e-17

XP_003420174.1 normal mucosa of esophagus-specific gene 1 pro... 79.3 2e-17

XP_010127156.1 PREDICTED: normal mucosa of esophagus-specific... 79.3 2e-17

XP_007169762.1 PREDICTED: normal mucosa of esophagus-specific... 79.0 2e-17

PKU48132.1 hypothetical protein llap_1603 [Limosa lapponica b... 79.0 2e-17

XP_021050663.1 normal mucosa of esophagus-specific gene 1 pro... 79.0 2e-17

XP_008944894.1 PREDICTED: normal mucosa of esophagus-specific... 79.0 2e-17

KFZ50540.1 Normal mucosa of esophagus-specific 1 [Podiceps cr... 78.6 3e-17

XP_011591681.1 PREDICTED: normal mucosa of esophagus-specific... 79.0 3e-17

XP_024088318.1 normal mucosa of esophagus-specific gene 1 pro... 78.6 3e-17

XP_007472560.1 PREDICTED: normal mucosa of esophagus-specific... 78.6 3e-17

KFV12888.1 Normal mucosa of esophagus-specific 1 [Tauraco ery... 78.2 3e-17

XP_021012290.1 normal mucosa of esophagus-specific gene 1 pro... 78.6 3e-17

XP_021570476.1 normal mucosa of esophagus-specific gene 1 pro... 78.2 4e-17

XP_009329209.1 PREDICTED: normal mucosa of esophagus-specific... 78.2 4e-17

KFQ76993.1 Normal mucosa of esophagus-specific 1 [Phoenicopte... 77.8 4e-17

KFR02653.1 Normal mucosa of esophagus-specific 1 [Nipponia ni... 77.8 5e-17

ADK26189.1 NMES1 [Equus caballus] 78.2 5e-17

XP_009938594.1 PREDICTED: normal mucosa of esophagus-specific... 78.2 5e-17

XP_005068597.1 normal mucosa of esophagus-specific gene 1 pro... 78.2 5e-17

XP_005364438.1 PREDICTED: normal mucosa of esophagus-specific... 77.8 6e-17

KFQ05494.1 Normal mucosa of esophagus-specific 1 [Leptosomus ... 77.4 6e-17

XP_010142183.1 PREDICTED: normal mucosa of esophagus-specific... 77.8 6e-17

XP_002753468.1 PREDICTED: normal mucosa of esophagus-specific... 77.8 6e-17

XP_019612223.1 PREDICTED: normal mucosa of esophagus-specific... 77.8 7e-17

XP_004639087.1 normal mucosa of esophagus-specific gene 1 pro... 77.8 7e-17

XP_009895563.1 PREDICTED: normal mucosa of esophagus-specific... 77.8 8e-17

XP_011755367.1 normal mucosa of esophagus-specific gene 1 pro... 77.4 8e-17

XP_006889193.1 PREDICTED: normal mucosa of esophagus-specific... 78.2 8e-17

XP_003900934.1 normal mucosa of esophagus-specific gene 1 pro... 77.4 8e-17

XP_006076379.1 PREDICTED: normal mucosa of esophagus-specific... 77.4 8e-17

XP_020029106.1 normal mucosa of esophagus-specific gene 1 pro... 77.4 9e-17

NP_115789.1 normal mucosa of esophagus-specific gene 1 protei... 77.4 9e-17

KFP56096.1 Normal mucosa of esophagus-specific 1 [Cariama cri... 77.0 9e-17

XP_007116783.1 normal mucosa of esophagus-specific gene 1 pro... 77.4 1e-16

XP_005889441.1 PREDICTED: normal mucosa of esophagus-specific... 77.4 1e-16

XP_002927945.1 PREDICTED: normal mucosa of esophagus-specific... 77.4 1e-16

XP_003266917.1 PREDICTED: normal mucosa of esophagus-specific... 77.4 1e-16

KFW03699.1 Normal mucosa of esophagus-specific 1 [Fulmarus gl... 77.0 1e-16

XP_009992763.1 PREDICTED: normal mucosa of esophagus-specific... 77.4 1e-16

KFV43323.1 Normal mucosa of esophagus-specific 1 [Gavia stell... 76.6 1e-16

XP_023596413.1 normal mucosa of esophagus-specific gene 1 pro... 77.0 1e-16

XP_004281317.1 PREDICTED: normal mucosa of esophagus-specific... 76.6 2e-16

KFP74640.1 Normal mucosa of esophagus-specific 1 [Apaloderma ... 76.3 2e-16

XP_014636168.1 PREDICTED: normal mucosa of esophagus-specific... 77.4 2e-16

XP_021500755.1 normal mucosa of esophagus-specific gene 1 pro... 76.3 2e-16

XP_022442896.1 normal mucosa of esophagus-specific gene 1 pro... 76.3 2e-16

XP_007081828.1 PREDICTED: normal mucosa of esophagus-specific... 76.3 2e-16

XP_004386338.2 normal mucosa of esophagus-specific gene 1 pro... 76.6 2e-16

XP_012297828.1 normal mucosa of esophagus-specific gene 1 pro... 76.3 2e-16

XP_024617130.1 normal mucosa of esophagus-specific gene 1 pro... 76.3 3e-16

XP_010630801.1 PREDICTED: normal mucosa of esophagus-specific... 76.3 3e-16

XP_008014946.1 PREDICTED: normal mucosa of esophagus-specific... 76.3 3e-16

XP_010351257.1 PREDICTED: normal mucosa of esophagus-specific... 76.3 3e-16

XP_005559496.1 PREDICTED: normal mucosa of esophagus-specific... 75.9 3e-16

XP_011281153.1 normal mucosa of esophagus-specific gene 1 pro... 75.9 4e-16

KFP93937.1 Normal mucosa of esophagus-specific 1 [Haliaeetus ... 75.5 4e-16

XP_004709465.1 PREDICTED: normal mucosa of esophagus-specific... 75.9 4e-16

XP_008141320.1 PREDICTED: normal mucosa of esophagus-specific... 75.9 4e-16

NP_001193905.1 normal mucosa of esophagus-specific gene 1 pro... 75.5 4e-16

ALIGNMENTS

>XP_024423805.1 normal mucosa of esophagus-specific gene 1 protein [Desmodus

rotundus]

Length=83

Score = 87.8 bits (216), Expect = 8e-21, Method: Compositional matrix adjust.

Identities = 42/78 (54%), Positives = 55/78 (71%), Gaps = 1/78 (1%)

Query 4 LLTLIGKHKELIPLVAAVGGAAVGATSFALYSLGKPG-LVARRDGGDLWEDVDPERPQKL 62

L+ K KELIPLV + AA GATSFA+YSL K ++ R++ + WE VDP PQKL

Sbjct 3 FFQLLMKKKELIPLVVIMTAAASGATSFAVYSLKKSDVIIDRKNNPEPWESVDPNVPQKL 62

Query 63 LTVHQLWRAIPELEEVRR 80

+TV+Q W+AI EL++VRR

Sbjct 63 ITVNQQWKAIEELQKVRR 80

>XP_009961649.1 PREDICTED: normal mucosa of esophagus-specific gene 1 protein

[Tyto alba]

Length=85

Score = 86.7 bits (213), Expect = 2e-20, Method: Compositional matrix adjust.

Identities = 41/83 (49%), Positives = 54/83 (65%), Gaps = 1/83 (1%)

Query 2 ARLLTLIGKHKELIPLVAAVGGAAVGATSFALYSLGKPGLVARRDGG-DLWEDVDPERPQ 60

A ++ KELIPLV V AAVGA SF+ YSL K ++ + G + WE VDP +PQ

Sbjct 3 ASFFQILKAKKELIPLVGVVTFAAVGALSFSAYSLTKSDVIINKSGNPEPWETVDPNKPQ 62

Query 61 KLLTVHQLWRAIPELEEVRRIER 83

KLLT+HQ W+ I ELE VR++ +

Sbjct 63 KLLTIHQKWKPIEELENVRKLTK 85

>XP_004578169.1 PREDICTED: normal mucosa of esophagus-specific gene 1 protein

[Ochotona princeps]

XP_012784477.1 PREDICTED: normal mucosa of esophagus-specific gene 1 protein

[Ochotona princeps]

Length=83

Score = 84.3 bits (207), Expect = 2e-19, Method: Compositional matrix adjust.

Identities = 41/78 (53%), Positives = 56/78 (72%), Gaps = 1/78 (1%)

Query 4 LLTLIGKHKELIPLVAAVGGAAVGATSFALYSLGKPG-LVARRDGGDLWEDVDPERPQKL 62

L L+ K KELIPLVA + AAVGA+SFA+YSL K ++ R+ + WE VDP PQKL

Sbjct 3 LFQLLMKKKELIPLVAFISFAAVGASSFAVYSLQKSDVIIDRKRNPEPWESVDPTVPQKL 62

Query 63 LTVHQLWRAIPELEEVRR 80

+T++Q W+ I EL++VR+

Sbjct 63 VTINQQWKPIEELQKVRK 80

>XP_009979561.1 PREDICTED: normal mucosa of esophagus-specific gene 1 protein

[Tauraco erythrolophus]

Length=87

Score = 83.6 bits (205), Expect = 4e-19, Method: Compositional matrix adjust.

Identities = 41/85 (48%), Positives = 54/85 (64%), Gaps = 2/85 (2%)

Query 2 ARLLTLIGKHKELIPLVAAVGGAAVGATSFALYSL-GKPGLVARRDGG-DLWEDVDPERP 59

++ KELIPLV V AAVGA SF++YSL K ++ + G + WE VDP +P

Sbjct 3 TSFFQILKAKKELIPLVGVVSFAAVGALSFSVYSLFSKSDVIINKSGNPEPWETVDPTKP 62

Query 60 QKLLTVHQLWRAIPELEEVRRIERG 84

QKLLTVHQ W+ I ELE VR++ +

Sbjct 63 QKLLTVHQKWKPIEELENVRKLTKS 87

>XP_003791849.1 normal mucosa of esophagus-specific gene 1 protein [Otolemur

garnettii]

Length=83

Score = 83.2 bits (204), Expect = 5e-19, Method: Compositional matrix adjust.

Identities = 39/78 (50%), Positives = 56/78 (72%), Gaps = 1/78 (1%)

Query 4 LLTLIGKHKELIPLVAAVGGAAVGATSFALYSLGKPG-LVARRDGGDLWEDVDPERPQKL 62

+ L+ K KELIPL + AAVGA+SFA+YSLGK ++ R+ + WE+VDP PQKL

Sbjct 3 VFQLLMKKKELIPLAVFITFAAVGASSFAVYSLGKSDVIIDRKRNPEPWENVDPNVPQKL 62

Query 63 LTVHQLWRAIPELEEVRR 80

+T++Q W+ I EL+++RR

Sbjct 63 VTINQQWKPIEELQKIRR 80

>XP_015728419.1 PREDICTED: normal mucosa of esophagus-specific gene 1 protein

[Coturnix japonica]

Length=86

Score = 83.2 bits (204), Expect = 5e-19, Method: Compositional matrix adjust.

Identities = 39/84 (46%), Positives = 56/84 (67%), Gaps = 2/84 (2%)

Query 2 ARLLTLIGKHKELIPLVAAVGGAAVGATSFALYSL-GKPGLVARRDGG-DLWEDVDPERP 59

A L+ K KELIPL+ + AAVGA +F++YSL K ++ + G + WE +DP RP

Sbjct 3 ASFFQLLRKKKELIPLIGVLSCAAVGAAAFSVYSLVSKSDVIINKSGNPEPWETIDPTRP 62

Query 60 QKLLTVHQLWRAIPELEEVRRIER 83

QKLLT+HQ W+ I ELE V+++ +

Sbjct 63 QKLLTIHQKWKPIEELESVKKLTK 86

>XP_009490219.1 PREDICTED: normal mucosa of esophagus-specific gene 1 protein

[Pelecanus crispus]

Length=86

Score = 82.8 bits (203), Expect = 8e-19, Method: Compositional matrix adjust.

Identities = 40/84 (48%), Positives = 54/84 (64%), Gaps = 2/84 (2%)

Query 2 ARLLTLIGKHKELIPLVAAVGGAAVGATSFALYSL-GKPGLVARRDGG-DLWEDVDPERP 59

A ++ KELIPLV V AA+GA SF+ YSL K ++ + G + WE VDP +P

Sbjct 3 ASFFQILKAKKELIPLVGVVSFAAIGALSFSAYSLFSKSDVIVNKSGNPEPWETVDPTKP 62

Query 60 QKLLTVHQLWRAIPELEEVRRIER 83

QKLLT+HQ W+ I ELE VR++ +

Sbjct 63 QKLLTIHQKWKPIEELENVRKLTK 86

>XP_009468393.1 PREDICTED: normal mucosa of esophagus-specific gene 1 protein

[Nipponia nippon]

Length=86

Score = 82.4 bits (202), Expect = 1e-18, Method: Compositional matrix adjust.

Identities = 40/84 (48%), Positives = 53/84 (63%), Gaps = 2/84 (2%)

Query 2 ARLLTLIGKHKELIPLVAAVGGAAVGATSFALYSL-GKPGLVARRDGG-DLWEDVDPERP 59

++ KELIPLV V AAVGA SF +YSL K ++ + G + WE VDP +P

Sbjct 3 TSFFQILKAKKELIPLVGVVSFAAVGALSFCVYSLFSKSDVIINKSGNPEPWETVDPTKP 62

Query 60 QKLLTVHQLWRAIPELEEVRRIER 83

QKLLT+HQ W+ I ELE VR++ +

Sbjct 63 QKLLTIHQKWKPIEELENVRKLTK 86

>XP_005019536.1 normal mucosa of esophagus-specific gene 1 protein [Anas platyrhynchos]

Length=86

Score = 82.4 bits (202), Expect = 1e-18, Method: Compositional matrix adjust.

Identities = 40/84 (48%), Positives = 53/84 (63%), Gaps = 2/84 (2%)

Query 2 ARLLTLIGKHKELIPLVAAVGGAAVGATSFALYSL-GKPGLVARRDGG-DLWEDVDPERP 59

+I KELIPLV V AAVGA +F+ YSL K ++ + G + WE +DP RP

Sbjct 3 TNFFHIIKSKKELIPLVGVVSFAAVGAVAFSAYSLFSKSDVIINKTGNPEPWETIDPTRP 62

Query 60 QKLLTVHQLWRAIPELEEVRRIER 83

QKLLT+HQ W+ I ELE VR++ +

Sbjct 63 QKLLTIHQKWKPIEELENVRKLTK 86

>XP_009642320.1 PREDICTED: normal mucosa of esophagus-specific gene 1 protein

[Egretta garzetta]

Length=86

Score = 82.0 bits (201), Expect = 1e-18, Method: Compositional matrix adjust.

Identities = 39/85 (46%), Positives = 55/85 (65%), Gaps = 2/85 (2%)

Query 1 MARLLTLIGKHKELIPLVAAVGGAAVGATSFALYSL-GKPGLVARRDGG-DLWEDVDPER 58

+ ++ KELIPLV V AAVGA SF++YS+ K ++ + G + WE VDP +

Sbjct 2 ITSFFQILKAKKELIPLVGVVSFAAVGALSFSVYSIFSKSDVIINKSGNPEPWETVDPTK 61

Query 59 PQKLLTVHQLWRAIPELEEVRRIER 83

PQKLLT+HQ W+ I ELE VR++ +

Sbjct 62 PQKLLTIHQKWKPIEELENVRKLTK 86

>XP_007933412.1 PREDICTED: normal mucosa of esophagus-specific gene 1 protein

[Orycteropus afer afer]

Length=83

Score = 82.0 bits (201), Expect = 1e-18, Method: Compositional matrix adjust.

Identities = 42/82 (51%), Positives = 55/82 (67%), Gaps = 1/82 (1%)

Query 3 RLLTLIGKHKELIPLVAAVGGAAVGATSFALYSLGKPGLVA-RRDGGDLWEDVDPERPQK 61

L+ K KELIPLVA + AA GATSFA+YSLGK ++ R+ + WE VDP P K

Sbjct 2 NFFQLLMKKKELIPLVAIMTFAASGATSFAVYSLGKTDVILDRKKNPEPWEHVDPTVPAK 61

Query 62 LLTVHQLWRAIPELEEVRRIER 83

L+T++Q W+ I EL++VRR R

Sbjct 62 LVTINQEWKPIEELQKVRRATR 83

>XP_009574734.1 PREDICTED: normal mucosa of esophagus-specific gene 1 protein

[Fulmarus glacialis]

Length=86

Score = 81.6 bits (200), Expect = 2e-18, Method: Compositional matrix adjust.

Identities = 40/84 (48%), Positives = 53/84 (63%), Gaps = 2/84 (2%)

Query 2 ARLLTLIGKHKELIPLVAAVGGAAVGATSFALYSL-GKPGLVARRDGG-DLWEDVDPERP 59

++ KELIPLV V AAVGA SF+ YSL K ++ + G + WE VDP +P

Sbjct 3 TSFFQILKAKKELIPLVGVVSFAAVGAFSFSAYSLFSKSDVIINKSGNPEPWETVDPTKP 62

Query 60 QKLLTVHQLWRAIPELEEVRRIER 83

QKLLT+HQ W+ I ELE VR++ +

Sbjct 63 QKLLTIHQKWKPIEELENVRKLTK 86

>XP_009707919.1 PREDICTED: normal mucosa of esophagus-specific gene 1 protein

[Cariama cristata]

Length=86

Score = 81.6 bits (200), Expect = 2e-18, Method: Compositional matrix adjust.

Identities = 39/84 (46%), Positives = 54/84 (64%), Gaps = 2/84 (2%)

Query 2 ARLLTLIGKHKELIPLVAAVGGAAVGATSFALYSL-GKPGLVARRDGG-DLWEDVDPERP 59

++ KELIPLV V AAVGA SF++YS+ K ++ + G + WE VDP +P

Sbjct 3 TSFFQILKAKKELIPLVGVVSFAAVGALSFSVYSIFSKSDVIINKSGNPEPWETVDPTKP 62

Query 60 QKLLTVHQLWRAIPELEEVRRIER 83

QKLLT+HQ W+ I ELE VR++ +

Sbjct 63 QKLLTIHQKWKPIEELENVRKLTK 86

>XP_009884434.1 PREDICTED: normal mucosa of esophagus-specific gene 1 protein

[Charadrius vociferus]

Length=86

Score = 81.3 bits (199), Expect = 3e-18, Method: Compositional matrix adjust.

Identities = 39/83 (47%), Positives = 54/83 (65%), Gaps = 2/83 (2%)

Query 3 RLLTLIGKHKELIPLVAAVGGAAVGATSFALYSL-GKPGLVARRDGG-DLWEDVDPERPQ 60

++ KELIPLV + AAVGA SF++YSL K ++ + G + WE VDP +PQ

Sbjct 4 NFFQILKAKKELIPLVGVLSFAAVGAASFSVYSLFSKSDVIINKSGNPEPWETVDPSKPQ 63

Query 61 KLLTVHQLWRAIPELEEVRRIER 83

KLLT+HQ W+ I ELE VR++ +

Sbjct 64 KLLTIHQKWKPIEELENVRKLTK 86

>XP_009811572.1 PREDICTED: normal mucosa of esophagus-specific gene 1 protein

[Gavia stellata]

XP_010288096.1 PREDICTED: normal mucosa of esophagus-specific gene 1 protein

[Phaethon lepturus]

Length=86

Score = 81.3 bits (199), Expect = 3e-18, Method: Compositional matrix adjust.

Identities = 40/84 (48%), Positives = 53/84 (63%), Gaps = 2/84 (2%)

Query 2 ARLLTLIGKHKELIPLVAAVGGAAVGATSFALYSL-GKPGLVARRDGG-DLWEDVDPERP 59

++ KELIPLV V AAVGA SF+ YSL K ++ + G + WE VDP +P

Sbjct 3 TSFFQILKAKKELIPLVGVVSFAAVGALSFSAYSLFSKSDVIINKSGNPEPWETVDPTKP 62

Query 60 QKLLTVHQLWRAIPELEEVRRIER 83

QKLLT+HQ W+ I ELE VR++ +

Sbjct 63 QKLLTIHQKWKPIEELENVRKLTK 86

>XP_010300338.1 PREDICTED: normal mucosa of esophagus-specific gene 1 protein

[Balearica regulorum gibbericeps]

Length=86

Score = 81.3 bits (199), Expect = 3e-18, Method: Compositional matrix adjust.

Identities = 39/84 (46%), Positives = 55/84 (65%), Gaps = 2/84 (2%)

Query 2 ARLLTLIGKHKELIPLVAAVGGAAVGATSFALYSL-GKPGLVARRDGG-DLWEDVDPERP 59

A ++ KELIPLV V AAVGA SF+ YS+ K ++ + G + WE VDP +P

Sbjct 3 ASFFQILKAKKELIPLVGVVSFAAVGALSFSAYSIFSKSDVIINKSGNPEPWETVDPTKP 62

Query 60 QKLLTVHQLWRAIPELEEVRRIER 83

QKLLT+HQ W+ I ELE+V+++ +

Sbjct 63 QKLLTIHQKWKPIEELEDVKKLTK 86

>XP_003756148.1 normal mucosa of esophagus-specific gene 1 protein [Sarcophilus

harrisii]

Length=83

Score = 81.3 bits (199), Expect = 3e-18, Method: Compositional matrix adjust.

Identities = 37/82 (45%), Positives = 56/82 (68%), Gaps = 1/82 (1%)

Query 3 RLLTLIGKHKELIPLVAAVGGAAVGATSFALYSLGKPG-LVARRDGGDLWEDVDPERPQK 61

+L L+ K KELIPL+A V A GA S LYSL K ++ +R + WE+V+P +PQK

Sbjct 2 NILELMMKRKELIPLIAFVSVAGFGAISMGLYSLSKSDVIINKRKNPEPWENVNPNKPQK 61

Query 62 LLTVHQLWRAIPELEEVRRIER 83

L+T++Q WR + EL++VR++ +

Sbjct 62 LITINQKWRPVEELQKVRKLTK 83

>NP_001181937.1 normal mucosa of esophagus-specific gene 1 protein [Equus caballus]

XP_008523061.1 PREDICTED: normal mucosa of esophagus-specific gene 1 protein

[Equus przewalskii]

XP_014702514.1 PREDICTED: normal mucosa of esophagus-specific gene 1 protein

isoform X1 [Equus asinus]

ADK26190.1 NMES1 [Equus caballus]

ADK26191.1 NMES1 [Equus caballus]

Length=83

Score = 80.9 bits (198), Expect = 3e-18, Method: Compositional matrix adjust.

Identities = 41/78 (53%), Positives = 53/78 (68%), Gaps = 1/78 (1%)

Query 4 LLTLIGKHKELIPLVAAVGGAAVGATSFALYSLGKPG-LVARRDGGDLWEDVDPERPQKL 62

L+ K KELIPLVA + AA GATSFALYSL K ++ R+ + WE VDP P KL

Sbjct 3 FFQLLRKKKELIPLVAFMTVAATGATSFALYSLRKTDVIIDRKRNPEPWETVDPSVPTKL 62

Query 63 LTVHQLWRAIPELEEVRR 80

+T++Q W+ I EL++VRR

Sbjct 63 ITINQEWKPIEELQKVRR 80

>XP_008682218.1 PREDICTED: normal mucosa of esophagus-specific gene 1 protein

[Ursus maritimus]

Length=83

Score = 80.9 bits (198), Expect = 3e-18, Method: Compositional matrix adjust.

Identities = 40/78 (51%), Positives = 55/78 (71%), Gaps = 1/78 (1%)

Query 4 LLTLIGKHKELIPLVAAVGGAAVGATSFALYSLGKPG-LVARRDGGDLWEDVDPERPQKL 62

+ L+ K KELIPLV + AAVGA+SFA+YSL K ++ R+ + WE VDP P+KL

Sbjct 3 IFQLLRKKKELIPLVLIMTTAAVGASSFAVYSLQKTDVIIDRKRNPEPWETVDPNVPRKL 62

Query 63 LTVHQLWRAIPELEEVRR 80

LT++Q W+ I EL++VRR

Sbjct 63 LTINQQWKPIEELQKVRR 80

>XP_010186034.1 PREDICTED: normal mucosa of esophagus-specific gene 1 protein

[Mesitornis unicolor]

Length=86

Score = 81.3 bits (199), Expect = 3e-18, Method: Compositional matrix adjust.

Identities = 39/84 (46%), Positives = 54/84 (64%), Gaps = 2/84 (2%)

Query 2 ARLLTLIGKHKELIPLVAAVGGAAVGATSFALYSL-GKPGLVARRDGG-DLWEDVDPERP 59

+ +I + KELIPLV V AAVGA SF +YSL K ++ + G + WE V+P P

Sbjct 3 TSIFQMIKRKKELIPLVGVVSFAAVGALSFCVYSLFSKSDVIINKSGNPEPWETVNPTTP 62

Query 60 QKLLTVHQLWRAIPELEEVRRIER 83

QKLLT+HQ W+ I ELE V+++ +

Sbjct 63 QKLLTIHQKWQPIEELENVKKLTK 86

>XP_006981453.1 PREDICTED: normal mucosa of esophagus-specific gene 1 protein

[Peromyscus maniculatus bairdii]

Length=83

Score = 80.9 bits (198), Expect = 4e-18, Method: Compositional matrix adjust.

Identities = 39/75 (52%), Positives = 53/75 (71%), Gaps = 1/75 (1%)

Query 10 KHKELIPLVAAVGGAAVGATSFALYSLGKPGLVA-RRDGGDLWEDVDPERPQKLLTVHQL 68

K+KELIPL + AA GATSFALY+L K +V R+ + WE VDP +PQKL+T++Q

Sbjct 9 KNKELIPLAVIITTAATGATSFALYALKKTDVVIDRKRNPEPWERVDPTQPQKLITINQQ 68

Query 69 WRAIPELEEVRRIER 83

W+ + EL++VRR R

Sbjct 69 WKPVEELQKVRRATR 83

>NP_001004174.1 normal mucosa of esophagus-specific gene 1 protein [Mus musculus]

XP_006499936.1 PREDICTED: normal mucosa of esophagus-specific gene 1 protein

isoform X1 [Mus musculus]

Q810Q5.1 RecName: Full=Normal mucosa of esophagus-specific gene 1 protein

AAH49633.1 Expressed sequence AA467197 [Mus musculus]

BAE29660.1 unnamed protein product [Mus musculus]

BAE24640.1 unnamed protein product [Mus musculus]

EDL28107.1 expressed sequence AA467197, isoform CRA_a [Mus musculus]

EDL28108.1 expressed sequence AA467197, isoform CRA_a [Mus musculus]

Length=83

Score = 80.9 bits (198), Expect = 4e-18, Method: Compositional matrix adjust.

Identities = 39/81 (48%), Positives = 56/81 (69%), Gaps = 1/81 (1%)

Query 4 LLTLIGKHKELIPLVAAVGGAAVGATSFALYSLGKPGLVA-RRDGGDLWEDVDPERPQKL 62

+ ++ K+KELIPL + AA GATSFALY+L K +V R+ + WE VDP +PQKL

Sbjct 3 VFQILMKNKELIPLAFFISVAATGATSFALYALKKTDVVIDRKRNPEPWEMVDPTQPQKL 62

Query 63 LTVHQLWRAIPELEEVRRIER 83

+T++Q W+ + EL++VRR R

Sbjct 63 ITINQQWKPVEELQKVRRATR 83

>XP_009864334.1 PREDICTED: normal mucosa of esophagus-specific gene 1 protein

[Apaloderma vittatum]

Length=84

Score = 80.9 bits (198), Expect = 5e-18, Method: Compositional matrix adjust.

Identities = 38/82 (46%), Positives = 53/82 (65%), Gaps = 2/82 (2%)

Query 4 LLTLIGKHKELIPLVAAVGGAAVGATSFALYSL-GKPGLVARRDGG-DLWEDVDPERPQK 61

++ KELIPLV V AAVG+ SF +YSL K ++ + G + WE +DP +PQK

Sbjct 3 FFQILKAKKELIPLVGVVSFAAVGSFSFCIYSLFSKSDVIINKTGNPEPWETIDPAKPQK 62

Query 62 LLTVHQLWRAIPELEEVRRIER 83

LLT+HQ W+ I ELE VR++ +

Sbjct 63 LLTIHQKWKPIEELENVRKLTK 84

>XP_003928954.1 PREDICTED: normal mucosa of esophagus-specific gene 1 protein

[Saimiri boliviensis boliviensis]

Length=83

Score = 80.5 bits (197), Expect = 6e-18, Method: Compositional matrix adjust.

Identities = 40/78 (51%), Positives = 55/78 (71%), Gaps = 1/78 (1%)

Query 4 LLTLIGKHKELIPLVAAVGGAAVGATSFALYSLGKPG-LVARRDGGDLWEDVDPERPQKL 62

L+ K+KELIPLV + AA GA+SFA+YSL K ++ R+ + WE+VDP PQKL

Sbjct 3 FFQLLKKNKELIPLVVFMSVAAGGASSFAVYSLRKTDVIIDRKRNPEPWENVDPTVPQKL 62

Query 63 LTVHQLWRAIPELEEVRR 80

LT++Q W+ I ELE+VR+

Sbjct 63 LTINQEWKPIEELEKVRK 80

>XP_009915140.1 PREDICTED: normal mucosa of esophagus-specific gene 1 protein

[Haliaeetus albicilla]

XP_010570191.1 PREDICTED: normal mucosa of esophagus-specific gene 1 protein

[Haliaeetus leucocephalus]

XP_010570192.1 PREDICTED: normal mucosa of esophagus-specific gene 1 protein

[Haliaeetus leucocephalus]

Length=86

Score = 80.1 bits (196), Expect = 7e-18, Method: Compositional matrix adjust.

Identities = 39/84 (46%), Positives = 54/84 (64%), Gaps = 2/84 (2%)

Query 2 ARLLTLIGKHKELIPLVAAVGGAAVGATSFALYSL-GKPGLVARRDGG-DLWEDVDPERP 59

++ KELIPLV V AAVGA SF++YSL K ++ + G + WE V+P +P

Sbjct 3 TSFFQILKAKKELIPLVGVVSFAAVGALSFSVYSLFSKSDVIINKTGNPEPWETVEPTKP 62

Query 60 QKLLTVHQLWRAIPELEEVRRIER 83

QKLLT+HQ W+ I ELE VR++ +

Sbjct 63 QKLLTIHQKWKPIEELESVRKLTK 86

>XP_005436326.1 PREDICTED: normal mucosa of esophagus-specific gene 1 protein

[Falco cherrug]

Length=86

Score = 80.1 bits (196), Expect = 7e-18, Method: Compositional matrix adjust.

Identities = 40/84 (48%), Positives = 54/84 (64%), Gaps = 2/84 (2%)

Query 2 ARLLTLIGKHKELIPLVAAVGGAAVGATSFALYSL-GKPGLVARRDGG-DLWEDVDPERP 59

A ++ KELIPLV V AAVGA SF++YSL K ++ + G + WE V+P +P

Sbjct 3 ASFFQILKAKKELIPLVGVVSFAAVGALSFSVYSLFSKSDVIINKSGNPEPWETVNPTKP 62

Query 60 QKLLTVHQLWRAIPELEEVRRIER 83

QKLLT+HQ W I ELE VR++ +

Sbjct 63 QKLLTIHQKWEPIEELEHVRKLMK 86

>XP_008583327.1 PREDICTED: normal mucosa of esophagus-specific gene 1 protein

[Galeopterus variegatus]

Length=83

Score = 80.1 bits (196), Expect = 8e-18, Method: Compositional matrix adjust.

Identities = 38/79 (48%), Positives = 54/79 (68%), Gaps = 1/79 (1%)

Query 3 RLLTLIGKHKELIPLVAAVGGAAVGATSFALYSLGKPG-LVARRDGGDLWEDVDPERPQK 61

L+ K KELIPLV AA GA+SFA+YSLGK ++ ++ + WE+VDP P+K

Sbjct 2 NFFQLLMKKKELIPLVVFTAVAATGASSFAVYSLGKSDVIIDKKRNPEPWENVDPSVPRK 61

Query 62 LLTVHQLWRAIPELEEVRR 80

L+T++Q W+ I EL++VRR

Sbjct 62 LITINQEWKPIEELQKVRR 80

>XP_013032709.1 PREDICTED: normal mucosa of esophagus-specific gene 1 protein

[Anser cygnoides domesticus]

Length=86

Score = 80.1 bits (196), Expect = 9e-18, Method: Compositional matrix adjust.

Identities = 38/84 (45%), Positives = 53/84 (63%), Gaps = 2/84 (2%)

Query 2 ARLLTLIGKHKELIPLVAAVGGAAVGATSFALYSL-GKPGLVARRDGG-DLWEDVDPERP 59

+I KELIPLV V AAVGA +F+ YSL K ++ + G + WE +DP RP

Sbjct 3 TNFFHIIKSKKELIPLVGVVSFAAVGAVAFSAYSLFSKSDVIINKTGNPEPWETIDPTRP 62

Query 60 QKLLTVHQLWRAIPELEEVRRIER 83

QKLLT+HQ W+ I +LE V+++ +

Sbjct 63 QKLLTIHQKWKPIEDLENVKKLTK 86

>NP_001008518.1 normal mucosa of esophagus-specific gene 1 protein [Rattus norvegicus]

Q5RK28.1 RecName: Full=Normal mucosa of esophagus-specific gene 1 protein

AAH86337.1 Hypothetical LOC302884 [Rattus norvegicus]

Length=83

Score = 80.1 bits (196), Expect = 9e-18, Method: Compositional matrix adjust.

Identities = 39/75 (52%), Positives = 52/75 (69%), Gaps = 1/75 (1%)

Query 10 KHKELIPLVAAVGGAAVGATSFALYSLGKPGLVA-RRDGGDLWEDVDPERPQKLLTVHQL 68

K+KELIPL + AA GA+SFALY+L K +V R+ + WE VDP +PQKLLT++Q

Sbjct 9 KNKELIPLAFFISAAATGASSFALYALKKTDVVIDRKRNPEPWETVDPTQPQKLLTINQE 68

Query 69 WRAIPELEEVRRIER 83

W+ + EL+ VRR R

Sbjct 69 WKPVEELQRVRRATR 83

>XP_004660917.1 PREDICTED: normal mucosa of esophagus-specific gene 1 protein

[Jaculus jaculus]

Length=83

Score = 79.7 bits (195), Expect = 1e-17, Method: Compositional matrix adjust.

Identities = 38/81 (47%), Positives = 54/81 (67%), Gaps = 1/81 (1%)

Query 4 LLTLIGKHKELIPLVAAVGGAAVGATSFALYSLGKPGLVARRDGG-DLWEDVDPERPQKL 62

+ + KHKELIPL + AA GA++F++Y+L K ++ R G + WE VDP PQKL

Sbjct 3 IFQTLMKHKELIPLAVIMITAATGASTFSMYALKKSDVIIDRKGNPEPWETVDPTVPQKL 62

Query 63 LTVHQLWRAIPELEEVRRIER 83

LT++Q W+ I EL++VRR R

Sbjct 63 LTINQQWKPIEELQKVRRATR 83

>XP_005241558.1 PREDICTED: normal mucosa of esophagus-specific gene 1 protein

[Falco peregrinus]

Length=86

Score = 79.7 bits (195), Expect = 1e-17, Method: Compositional matrix adjust.

Identities = 40/84 (48%), Positives = 54/84 (64%), Gaps = 2/84 (2%)

Query 2 ARLLTLIGKHKELIPLVAAVGGAAVGATSFALYSL-GKPGLVARRDGG-DLWEDVDPERP 59

A ++ KELIPLV V AAVGA SF++YSL K ++ + G + WE V+P +P

Sbjct 3 ASFFQILKAKKELIPLVGVVSFAAVGALSFSVYSLFSKSDVIINKSGNPEPWEAVNPTKP 62

Query 60 QKLLTVHQLWRAIPELEEVRRIER 83

QKLLT+HQ W I ELE VR++ +

Sbjct 63 QKLLTIHQKWEPIEELEHVRKLMK 86

>XP_009512516.1 PREDICTED: normal mucosa of esophagus-specific gene 1 protein

[Phalacrocorax carbo]

Length=86

Score = 79.7 bits (195), Expect = 1e-17, Method: Compositional matrix adjust.

Identities = 40/84 (48%), Positives = 53/84 (63%), Gaps = 2/84 (2%)

Query 2 ARLLTLIGKHKELIPLVAAVGGAAVGATSFALYSL-GKPGLVARRDGG-DLWEDVDPERP 59

++ KELIPLV V AAVGA SF+ YSL GK ++ + G + WE VDP +P

Sbjct 3 TSFFQILKAKKELIPLVGVVSFAAVGALSFSAYSLFGKSDVIINKSGNPEPWETVDPTKP 62

Query 60 QKLLTVHQLWRAIPELEEVRRIER 83

QKLLT+ Q W+ I ELE VR++ +

Sbjct 63 QKLLTIQQKWKPIEELENVRKLMK 86

>XP_003209505.1 PREDICTED: normal mucosa of esophagus-specific gene 1 protein

[Meleagris gallopavo]

Length=86

Score = 79.7 bits (195), Expect = 1e-17, Method: Compositional matrix adjust.

Identities = 39/84 (46%), Positives = 53/84 (63%), Gaps = 2/84 (2%)

Query 2 ARLLTLIGKHKELIPLVAAVGGAAVGATSFALYSL-GKPGLVARRDGG-DLWEDVDPERP 59

A L+ KELIPL+ V AAVGA +F+ YSL K ++ + + WE VDP RP

Sbjct 3 ASFFQLLRTKKELIPLIGVVSCAAVGALAFSAYSLVSKSDVIINKSANPEPWETVDPTRP 62

Query 60 QKLLTVHQLWRAIPELEEVRRIER 83

QKLLT+HQ W+ I ELE V+++ +

Sbjct 63 QKLLTIHQKWKPIEELESVKKLTK 86

>XP_012504650.1 PREDICTED: normal mucosa of esophagus-specific gene 1 protein

[Propithecus coquereli]

Length=83

Score = 79.3 bits (194), Expect = 1e-17, Method: Compositional matrix adjust.

Identities = 40/78 (51%), Positives = 52/78 (67%), Gaps = 1/78 (1%)

Query 4 LLTLIGKHKELIPLVAAVGGAAVGATSFALYSLGKPGLVARRDGG-DLWEDVDPERPQKL 62

L+ K KELIPLV AA GA+SFA+YSL K ++ R G + WE VDP PQKL

Sbjct 3 FFQLLMKKKELIPLVVFTTFAAGGASSFAVYSLRKSDVIIDRKGNPEPWETVDPNVPQKL 62

Query 63 LTVHQLWRAIPELEEVRR 80

+T++Q W+ I EL++VRR

Sbjct 63 ITINQQWKPIEELQQVRR 80

>XP_009274782.1 PREDICTED: normal mucosa of esophagus-specific gene 1 protein

[Aptenodytes forsteri]

Length=86

Score = 79.7 bits (195), Expect = 1e-17, Method: Compositional matrix adjust.

Identities = 38/84 (45%), Positives = 54/84 (64%), Gaps = 2/84 (2%)

Query 2 ARLLTLIGKHKELIPLVAAVGGAAVGATSFALYSL-GKPGLVARRDGG-DLWEDVDPERP 59

++ KELIPLV V AAVGA SF++YSL K ++ + G + WE VDP +P

Sbjct 3 TSFFQILKAKKELIPLVGVVSFAAVGALSFSVYSLFSKSDVIINKSGNPEPWETVDPTKP 62

Query 60 QKLLTVHQLWRAIPELEEVRRIER 83

QKLLT++Q W+ + ELE VR++ +

Sbjct 63 QKLLTINQKWKPVEELENVRKLTK 86

>XP_011353361.1 normal mucosa of esophagus-specific gene 1 protein [Pteropus

vampyrus]

Length=83

Score = 79.3 bits (194), Expect = 1e-17, Method: Compositional matrix adjust.

Identities = 39/81 (48%), Positives = 53/81 (65%), Gaps = 1/81 (1%)

Query 4 LLTLIGKHKELIPLVAAVGGAAVGATSFALYSLGKPG-LVARRDGGDLWEDVDPERPQKL 62

L L+ K KELIPL + AA GA SFALYSL K ++ R+ + WE+VDP P+KL

Sbjct 3 LFQLLMKKKELIPLAVIMTAAACGALSFALYSLKKTDVIIDRKRNPEPWENVDPNVPRKL 62

Query 63 LTVHQLWRAIPELEEVRRIER 83

+T++Q W+ I EL++ RR R

Sbjct 63 ITINQQWKPIEELQKARRASR 83

>XP_010208579.1 PREDICTED: normal mucosa of esophagus-specific gene 1 protein

[Colius striatus]

Length=86

Score = 79.7 bits (195), Expect = 1e-17, Method: Compositional matrix adjust.

Identities = 40/84 (48%), Positives = 56/84 (67%), Gaps = 2/84 (2%)

Query 2 ARLLTLIGKHKELIPLVAAVGGAAVGATSFALYSL-GKPGLVARRDGG-DLWEDVDPERP 59

A ++ KELIPLV + AAVGA SF++YSL K ++ + G + WE VDP +P

Sbjct 3 AGFFQILKAKKELIPLVGVLSFAAVGAFSFSVYSLFSKSDVIINKSGNPEPWETVDPTKP 62

Query 60 QKLLTVHQLWRAIPELEEVRRIER 83

QKLLTV+Q W+ I ELE+VR++ +

Sbjct 63 QKLLTVNQKWKPIEELEDVRKLTK 86

>XP_006920466.1 normal mucosa of esophagus-specific gene 1 protein [Pteropus

alecto]

Length=83

Score = 79.3 bits (194), Expect = 1e-17, Method: Compositional matrix adjust.

Identities = 40/81 (49%), Positives = 53/81 (65%), Gaps = 1/81 (1%)

Query 4 LLTLIGKHKELIPLVAAVGGAAVGATSFALYSLGKPG-LVARRDGGDLWEDVDPERPQKL 62

L L+ K KELIPL + AA GA SFALYSL K ++ R+ + WE VDP P+KL

Sbjct 3 LFQLLMKKKELIPLAVIMTAAASGALSFALYSLKKTDVIIDRKRNPEPWESVDPNVPRKL 62

Query 63 LTVHQLWRAIPELEEVRRIER 83

+T++Q W+ I EL++VRR R

Sbjct 63 ITINQQWKPIEELQKVRRASR 83

>XP_017400911.1 PREDICTED: normal mucosa of esophagus-specific gene 1 protein

[Cebus capucinus imitator]

Length=83

Score = 79.3 bits (194), Expect = 2e-17, Method: Compositional matrix adjust.

Identities = 38/78 (49%), Positives = 54/78 (69%), Gaps = 1/78 (1%)

Query 4 LLTLIGKHKELIPLVAAVGGAAVGATSFALYSLGKPGLVARRDGG-DLWEDVDPERPQKL 62

L+ K+KELIPLV + AA GA+SFA+YSL K ++ R G + WE VDP PQKL

Sbjct 3 FFQLLKKNKELIPLVVFMAVAASGASSFAVYSLRKTDVIIDRKGNPEPWETVDPTVPQKL 62

Query 63 LTVHQLWRAIPELEEVRR 80

+T++Q W+ + EL++VR+

Sbjct 63 ITINQEWKPVEELQKVRK 80

>XP_007539976.1 PREDICTED: normal mucosa of esophagus-specific gene 1 protein-like

[Erinaceus europaeus]

XP_007517367.1 PREDICTED: normal mucosa of esophagus-specific gene 1 protein

[Erinaceus europaeus]

Length=83

Score = 79.3 bits (194), Expect = 2e-17, Method: Compositional matrix adjust.

Identities = 39/79 (49%), Positives = 55/79 (70%), Gaps = 1/79 (1%)

Query 3 RLLTLIGKHKELIPLVAAVGGAAVGATSFALYSLGKPG-LVARRDGGDLWEDVDPERPQK 61

L L+ K KELIPLV + AA GA+SFA+YSL K ++ R++ + WE+VD PQK

Sbjct 2 NLFQLLMKKKELIPLVLIMTTAAGGASSFAVYSLSKSDVIIDRKNNPEPWENVDLNVPQK 61

Query 62 LLTVHQLWRAIPELEEVRR 80

L+T++Q W+ I EL++VRR

Sbjct 62 LITINQQWKPIEELQKVRR 80

>XP_004461217.1 normal mucosa of esophagus-specific gene 1 protein-like [Dasypus

novemcinctus]

XP_004461218.1 normal mucosa of esophagus-specific gene 1 protein-like [Dasypus

novemcinctus]

XP_004461219.1 normal mucosa of esophagus-specific gene 1 protein-like [Dasypus

novemcinctus]

Length=83

Score = 79.3 bits (194), Expect = 2e-17, Method: Compositional matrix adjust.

Identities = 41/78 (53%), Positives = 52/78 (67%), Gaps = 1/78 (1%)

Query 4 LLTLIGKHKELIPLVAAVGGAAVGATSFALYSLGKPGLVARRDGG-DLWEDVDPERPQKL 62

L+ K KELIPLV + AA GA+SFA+YSL K ++ R G + WE VDP PQKL

Sbjct 3 FFQLLMKKKELIPLVVIMTFAAGGASSFAVYSLRKTDVIIDRKGNPEPWESVDPNIPQKL 62

Query 63 LTVHQLWRAIPELEEVRR 80

LT++Q WR I EL++ RR

Sbjct 63 LTINQEWRPIEELQKARR 80

>XP_003420174.1 normal mucosa of esophagus-specific gene 1 protein [Loxodonta

africana]

Length=83

Score = 79.3 bits (194), Expect = 2e-17, Method: Compositional matrix adjust.

Identities = 40/81 (49%), Positives = 53/81 (65%), Gaps = 1/81 (1%)

Query 4 LLTLIGKHKELIPLVAAVGGAAVGATSFALYSLGKPGLVA-RRDGGDLWEDVDPERPQKL 62

L+ K+KELIPLV V AA GA+ FALYSL K ++ R+ + WE VDP PQKL

Sbjct 3 FFQLLMKNKELIPLVVLVTAAASGASWFALYSLRKSDVITDRKRNPEPWETVDPTVPQKL 62

Query 63 LTVHQLWRAIPELEEVRRIER 83

LT++Q W+ I EL+ VR+ +

Sbjct 63 LTINQQWKPIEELQMVRKASK 83

>XP_010127156.1 PREDICTED: normal mucosa of esophagus-specific gene 1 protein

[Chlamydotis macqueenii]

Length=86

Score = 79.3 bits (194), Expect = 2e-17, Method: Compositional matrix adjust.

Identities = 37/84 (44%), Positives = 54/84 (64%), Gaps = 2/84 (2%)

Query 2 ARLLTLIGKHKELIPLVAAVGGAAVGATSFALYSL-GKPGLVARRDGG-DLWEDVDPERP 59

++ KELIPLV V AAVGA +F+ YSL K ++ + G + WE +DP +P

Sbjct 3 TSFFQILKTKKELIPLVGVVSFAAVGALAFSAYSLFSKSDVIINKSGNPEPWETIDPTKP 62

Query 60 QKLLTVHQLWRAIPELEEVRRIER 83

QKLLT+HQ W+ I ELE+V+++ +

Sbjct 63 QKLLTIHQKWKPIEELEDVKKLTK 86

>XP_007169762.1 PREDICTED: normal mucosa of esophagus-specific gene 1 protein

[Balaenoptera acutorostrata scammoni]

Length=83

Score = 79.0 bits (193), Expect = 2e-17, Method: Compositional matrix adjust.

Identities = 40/81 (49%), Positives = 53/81 (65%), Gaps = 1/81 (1%)

Query 4 LLTLIGKHKELIPLVAAVGGAAVGATSFALYSLGKPG-LVARRDGGDLWEDVDPERPQKL 62

L+ K KELIPLV AA GA SFALYSL KP ++ R+ + WE VDP P+KL

Sbjct 3 FFQLLMKRKELIPLVLFTTVAATGALSFALYSLRKPDVIIDRKRNPEPWETVDPTAPRKL 62

Query 63 LTVHQLWRAIPELEEVRRIER 83

+T++Q W+ I EL++VR+ R

Sbjct 63 ITINQEWKPIEELQKVRKATR 83

>PKU48132.1 hypothetical protein llap_1603 [Limosa lapponica baueri]

Length=86

Score = 79.0 bits (193), Expect = 2e-17, Method: Compositional matrix adjust.

Identities = 38/84 (45%), Positives = 56/84 (67%), Gaps = 2/84 (2%)

Query 2 ARLLTLIGKHKELIPLVAAVGGAAVGATSFALYSL-GKPGLVARRDGG-DLWEDVDPERP 59

A + ++ KELIPLV + AAVGA SF++YSL K ++ + G + WE VDP +P

Sbjct 3 ANFIQILKAKKELIPLVGVLSFAAVGALSFSVYSLFTKSDVIINKSGNPEPWETVDPSKP 62

Query 60 QKLLTVHQLWRAIPELEEVRRIER 83

QKLLT++Q W+ I ELE V+++ +

Sbjct 63 QKLLTINQKWKPIEELESVKKLTK 86

>XP_021050663.1 normal mucosa of esophagus-specific gene 1 protein [Mus pahari]

XP_021050664.1 normal mucosa of esophagus-specific gene 1 protein [Mus pahari]

Length=83

Score = 79.0 bits (193), Expect = 2e-17, Method: Compositional matrix adjust.

Identities = 38/81 (47%), Positives = 56/81 (69%), Gaps = 1/81 (1%)

Query 4 LLTLIGKHKELIPLVAAVGGAAVGATSFALYSLGKPGLVA-RRDGGDLWEDVDPERPQKL 62

+ ++ K+KELIPL + AA GA+SFALY+L K +V R+ + WE VDP +PQKL

Sbjct 3 VFQILMKNKELIPLAFFISVAATGASSFALYALKKTDVVIDRKRNPEPWELVDPTQPQKL 62

Query 63 LTVHQLWRAIPELEEVRRIER 83

+T++Q W+ + EL++VRR R

Sbjct 63 ITINQQWKPVEELQKVRRATR 83

>XP_008944894.1 PREDICTED: normal mucosa of esophagus-specific gene 1 protein

[Merops nubicus]

Length=86

Score = 79.0 bits (193), Expect = 2e-17, Method: Compositional matrix adjust.

Identities = 39/84 (46%), Positives = 56/84 (67%), Gaps = 2/84 (2%)

Query 2 ARLLTLIGKHKELIPLVAAVGGAAVGATSFALYSL-GKPGLVARRDGG-DLWEDVDPERP 59

A L ++ KELIPLV V AAVGA SF+ YSL K ++ + G + WE VDP +P

Sbjct 3 ASFLHILKSKKELIPLVGIVSLAAVGALSFSAYSLFSKSDVIINKSGNPEPWETVDPTKP 62

Query 60 QKLLTVHQLWRAIPELEEVRRIER 83

QKLLT++Q W+ + ELE+V+++ +

Sbjct 63 QKLLTINQKWKPVEELEQVKKLTK 86

>KFZ50540.1 Normal mucosa of esophagus-specific 1, partial [Podiceps cristatus]

Length=73

Score = 78.6 bits (192), Expect = 3e-17, Method: Compositional matrix adjust.

Identities = 39/73 (53%), Positives = 51/73 (70%), Gaps = 2/73 (3%)

Query 13 ELIPLVAAVGGAAVGATSFALYSL-GKPGLVARRDGG-DLWEDVDPERPQKLLTVHQLWR 70

+LIPLV V AAVGA SF +YSL K +V + G + WE VDP +PQKLLT+HQ W+

Sbjct 1 QLIPLVGVVSFAAVGAFSFCVYSLFSKSDVVINKSGNPEPWETVDPTKPQKLLTIHQKWK 60

Query 71 AIPELEEVRRIER 83

I ELE+VR++ +

Sbjct 61 PIEELEDVRKLTK 73

>XP_011591681.1 PREDICTED: normal mucosa of esophagus-specific gene 1 protein

[Aquila chrysaetos canadensis]

XP_011591689.1 PREDICTED: normal mucosa of esophagus-specific gene 1 protein

[Aquila chrysaetos canadensis]

XP_011591695.1 PREDICTED: normal mucosa of esophagus-specific gene 1 protein

[Aquila chrysaetos canadensis]

Length=86

Score = 79.0 bits (193), Expect = 3e-17, Method: Compositional matrix adjust.

Identities = 38/84 (45%), Positives = 53/84 (63%), Gaps = 2/84 (2%)

Query 2 ARLLTLIGKHKELIPLVAAVGGAAVGATSFALYSL-GKPGLVARRDGG-DLWEDVDPERP 59

++ KELIPL V AAVGA SF++YSL K ++ + G + WE V+P +P

Sbjct 3 TSFFQILKAKKELIPLAGVVSFAAVGALSFSVYSLFSKSDVIINKSGNPEPWETVEPTKP 62

Query 60 QKLLTVHQLWRAIPELEEVRRIER 83

QKLLT+HQ W+ I ELE VR++ +

Sbjct 63 QKLLTIHQKWKPIEELENVRKLTK 86

>XP_024088318.1 normal mucosa of esophagus-specific gene 1 protein [Pongo abelii]

PNJ15552.1 C15orf48 isoform 2 [Pongo abelii]

PNJ15553.1 C15orf48 isoform 3 [Pongo abelii]

Length=83

Score = 78.6 bits (192), Expect = 3e-17, Method: Compositional matrix adjust.

Identities = 39/81 (48%), Positives = 55/81 (68%), Gaps = 1/81 (1%)

Query 4 LLTLIGKHKELIPLVAAVGGAAVGATSFALYSLGKPGLVA-RRDGGDLWEDVDPERPQKL 62

L+ K KELIPLV V AA GA+SFA+YSL K ++ R+ + WE VDP PQKL

Sbjct 3 FFQLLMKRKELIPLVVFVTVAAGGASSFAVYSLWKTDVILDRKKNPEPWETVDPTVPQKL 62

Query 63 LTVHQLWRAIPELEEVRRIER 83

+T++Q W+ I EL++V+R+ +

Sbjct 63 ITINQQWKPIEELQKVQRVTK 83

>XP_007472560.1 PREDICTED: normal mucosa of esophagus-specific gene 1 protein

[Monodelphis domestica]

Length=83

Score = 78.6 bits (192), Expect = 3e-17, Method: Compositional matrix adjust.

Identities = 34/82 (41%), Positives = 53/82 (65%), Gaps = 1/82 (1%)

Query 3 RLLTLIGKHKELIPLVAAVGGAAVGATSFALYSLGKPGLV-ARRDGGDLWEDVDPERPQK 61

+ + K KELIPL+A V A GA + Y+L KP ++ RR + WE+V+P PQK

Sbjct 2 NFVQFLMKRKELIPLIAFVSMAGCGAVGISFYTLAKPDVILNRRQNPEPWENVNPNHPQK 61

Query 62 LLTVHQLWRAIPELEEVRRIER 83

L+T++Q W+ I EL++VR++ +

Sbjct 62 LITINQKWKPIEELQKVRKLTK 83

>KFV12888.1 Normal mucosa of esophagus-specific 1, partial [Tauraco erythrolophus]

Length=73

Score = 78.2 bits (191), Expect = 3e-17, Method: Compositional matrix adjust.

Identities = 39/73 (53%), Positives = 51/73 (70%), Gaps = 2/73 (3%)

Query 13 ELIPLVAAVGGAAVGATSFALYSL-GKPGLVARRDGG-DLWEDVDPERPQKLLTVHQLWR 70

+LIPLV V AAVGA SF++YSL K ++ + G + WE VDP +PQKLLTVHQ W+

Sbjct 1 QLIPLVGVVSFAAVGALSFSVYSLFSKSDVIINKSGNPEPWETVDPTKPQKLLTVHQKWK 60

Query 71 AIPELEEVRRIER 83

I ELE VR++ +

Sbjct 61 PIEELENVRKLTK 73

>XP_021012290.1 normal mucosa of esophagus-specific gene 1 protein [Mus caroli]

Length=83

Score = 78.6 bits (192), Expect = 3e-17, Method: Compositional matrix adjust.

Identities = 38/81 (47%), Positives = 55/81 (68%), Gaps = 1/81 (1%)

Query 4 LLTLIGKHKELIPLVAAVGGAAVGATSFALYSLGKPGLVA-RRDGGDLWEDVDPERPQKL 62

+ ++ K+KELIPL + AA GATSF LY+L K +V R+ + WE VDP +PQKL

Sbjct 3 VFQILMKNKELIPLAFFISVAATGATSFGLYALKKTDVVIDRKRNPEPWEMVDPTQPQKL 62

Query 63 LTVHQLWRAIPELEEVRRIER 83

+T++Q W+ + EL++VRR R

Sbjct 63 ITINQQWKPVEELQKVRRATR 83

>XP_021570476.1 normal mucosa of esophagus-specific gene 1 protein [Carlito syrichta]

Length=83

Score = 78.2 bits (191), Expect = 4e-17, Method: Compositional matrix adjust.

Identities = 39/78 (50%), Positives = 52/78 (67%), Gaps = 1/78 (1%)

Query 4 LLTLIGKHKELIPLVAAVGGAAVGATSFALYSLGKPGLVARRDGG-DLWEDVDPERPQKL 62

L+ K KEL+PLV + AA GA SFA+YSL K ++ R G + WE VDP PQKL

Sbjct 3 FFQLLMKKKELVPLVLIMTFAASGALSFAVYSLRKSDVIIDRKGNPEPWETVDPTVPQKL 62

Query 63 LTVHQLWRAIPELEEVRR 80

LT++Q W+ + EL++VRR

Sbjct 63 LTINQQWKPVEELQKVRR 80

>XP_009329209.1 PREDICTED: normal mucosa of esophagus-specific gene 1 protein

[Pygoscelis adeliae]

Length=86

Score = 78.2 bits (191), Expect = 4e-17, Method: Compositional matrix adjust.

Identities = 38/84 (45%), Positives = 53/84 (63%), Gaps = 2/84 (2%)

Query 2 ARLLTLIGKHKELIPLVAAVGGAAVGATSFALYSL-GKPGLVARRDGG-DLWEDVDPERP 59

++ KELIPLV V AAVGA SF+ YSL K ++ + G + WE VDP +P

Sbjct 3 TSFFQILKAKKELIPLVGVVSFAAVGALSFSAYSLFSKSDVIINKSGNPEPWETVDPTKP 62

Query 60 QKLLTVHQLWRAIPELEEVRRIER 83

QKLLT++Q W+ + ELE VR++ +

Sbjct 63 QKLLTINQKWKPVEELESVRKLTK 86

>KFQ76993.1 Normal mucosa of esophagus-specific 1, partial [Phoenicopterus

ruber ruber]

Length=73

Score = 77.8 bits (190), Expect = 4e-17, Method: Compositional matrix adjust.

Identities = 38/73 (52%), Positives = 51/73 (70%), Gaps = 2/73 (3%)

Query 13 ELIPLVAAVGGAAVGATSFALYSL-GKPGLVARRDGG-DLWEDVDPERPQKLLTVHQLWR 70

+LIPLV V AAVGA SF +YSL K ++ + G + WE VDP +PQKLLT+HQ W+

Sbjct 1 QLIPLVGVVSFAAVGALSFCVYSLFSKSDVIINKSGNPEPWETVDPTKPQKLLTIHQKWK 60

Query 71 AIPELEEVRRIER 83

I ELE+VR++ +

Sbjct 61 PIEELEDVRKLTK 73

>KFR02653.1 Normal mucosa of esophagus-specific 1, partial [Nipponia nippon]

Length=73

Score = 77.8 bits (190), Expect = 5e-17, Method: Compositional matrix adjust.

Identities = 38/73 (52%), Positives = 50/73 (68%), Gaps = 2/73 (3%)

Query 13 ELIPLVAAVGGAAVGATSFALYSL-GKPGLVARRDGG-DLWEDVDPERPQKLLTVHQLWR 70

+LIPLV V AAVGA SF +YSL K ++ + G + WE VDP +PQKLLT+HQ W+

Sbjct 1 QLIPLVGVVSFAAVGALSFCVYSLFSKSDVIINKSGNPEPWETVDPTKPQKLLTIHQKWK 60

Query 71 AIPELEEVRRIER 83

I ELE VR++ +

Sbjct 61 PIEELENVRKLTK 73

>ADK26189.1 NMES1 [Equus caballus]

Length=83

Score = 78.2 bits (191), Expect = 5e-17, Method: Compositional matrix adjust.

Identities = 40/78 (51%), Positives = 52/78 (67%), Gaps = 1/78 (1%)

Query 4 LLTLIGKHKELIPLVAAVGGAAVGATSFALYSLGKPG-LVARRDGGDLWEDVDPERPQKL 62

L+ K KELIPLVA + AA GATSFALYS K ++ R+ + WE VDP P KL

Sbjct 3 FFQLLRKKKELIPLVAFMTVAATGATSFALYSPRKTDVIIDRKRNPEPWETVDPSVPTKL 62

Query 63 LTVHQLWRAIPELEEVRR 80

+T++Q W+ I EL++VRR

Sbjct 63 ITINQEWKPIEELQKVRR 80

>XP_009938594.1 PREDICTED: normal mucosa of esophagus-specific gene 1 protein

[Opisthocomus hoazin]

Length=87

Score = 78.2 bits (191), Expect = 5e-17, Method: Compositional matrix adjust.

Identities = 38/79 (48%), Positives = 54/79 (68%), Gaps = 2/79 (3%)

Query 7 LIGKHKELIPLVAAVGGAAVGATSFALYSL-GKPGLVARRDGG-DLWEDVDPERPQKLLT 64

++ + KELIPLV V AAVGA +F+ YSL K ++ + G + WE +DP +PQKLLT

Sbjct 9 VLNRRKELIPLVGVVSFAAVGALAFSAYSLFSKSDVIINKSGNPEPWETIDPTQPQKLLT 68

Query 65 VHQLWRAIPELEEVRRIER 83

+HQ W+ I ELE VR++ +

Sbjct 69 IHQKWKPIEELENVRKLTK 87

>XP_005068597.1 normal mucosa of esophagus-specific gene 1 protein [Mesocricetus

auratus]

Length=83

Score = 78.2 bits (191), Expect = 5e-17, Method: Compositional matrix adjust.

Identities = 38/75 (51%), Positives = 53/75 (71%), Gaps = 1/75 (1%)

Query 10 KHKELIPLVAAVGGAAVGATSFALYSLGKPGLVA-RRDGGDLWEDVDPERPQKLLTVHQL 68

K+KELIPL + AA GA+SFALY+L K +V R+ + WE VDP +PQKL+T++Q

Sbjct 9 KNKELIPLAFIITVAATGASSFALYALKKTDVVIDRKRNPEPWEMVDPSQPQKLITINQQ 68

Query 69 WRAIPELEEVRRIER 83

W+ + EL++VRR R

Sbjct 69 WKPVEELQKVRRATR 83

>XP_005364438.1 PREDICTED: normal mucosa of esophagus-specific gene 1 protein

[Microtus ochrogaster]

Length=83

Score = 77.8 bits (190), Expect = 6e-17, Method: Compositional matrix adjust.

Identities = 37/77 (48%), Positives = 54/77 (70%), Gaps = 1/77 (1%)

Query 8 IGKHKELIPLVAAVGGAAVGATSFALYSLGKPGLVA-RRDGGDLWEDVDPERPQKLLTVH 66

+ K+KELIPL + AA GA+SFA+Y+L K +V R+ + WE VDP +PQKL+T++

Sbjct 7 LSKNKELIPLAFIITLAATGASSFAMYALKKTDVVIDRKRNPEPWEMVDPTQPQKLITIN 66

Query 67 QLWRAIPELEEVRRIER 83

Q W+ + EL++VRR R

Sbjct 67 QQWKPVEELQKVRRATR 83

>KFQ05494.1 Normal mucosa of esophagus-specific 1, partial [Leptosomus discolor]

Length=73

Score = 77.4 bits (189), Expect = 6e-17, Method: Compositional matrix adjust.

Identities = 38/73 (52%), Positives = 50/73 (68%), Gaps = 2/73 (3%)

Query 13 ELIPLVAAVGGAAVGATSFALYSL-GKPGLVARRDGG-DLWEDVDPERPQKLLTVHQLWR 70

+LIPLV V AAVGA SF++YSL K ++ + G + WE VDP +PQKLLT+HQ W+

Sbjct 1 QLIPLVGVVSFAAVGALSFSVYSLFSKSDVIVNKSGNPEPWETVDPTKPQKLLTIHQKWK 60

Query 71 AIPELEEVRRIER 83

I ELE VR + +

Sbjct 61 PIEELENVRNLTK 73

>XP_010142183.1 PREDICTED: normal mucosa of esophagus-specific gene 1 protein

[Buceros rhinoceros silvestris]

Length=86

Score = 77.8 bits (190), Expect = 6e-17, Method: Compositional matrix adjust.

Identities = 37/84 (44%), Positives = 53/84 (63%), Gaps = 2/84 (2%)

Query 2 ARLLTLIGKHKELIPLVAAVGGAAVGATSFALYSLGKPG--LVARRDGGDLWEDVDPERP 59

+ ++ KELIPLV + AAVGA SF+ YSL ++ + + WE+VDP +P

Sbjct 3 SSFFHILKAKKELIPLVGIISFAAVGALSFSAYSLFSKSDVILNKSSNPEPWENVDPTKP 62

Query 60 QKLLTVHQLWRAIPELEEVRRIER 83

QKLLT+ Q W+ I ELE+VR+I +

Sbjct 63 QKLLTIRQKWKPIEELEDVRKITK 86

>XP_002753468.1 PREDICTED: normal mucosa of esophagus-specific gene 1 protein

[Callithrix jacchus]

Length=83

Score = 77.8 bits (190), Expect = 6e-17, Method: Compositional matrix adjust.

Identities = 38/78 (49%), Positives = 53/78 (68%), Gaps = 1/78 (1%)

Query 4 LLTLIGKHKELIPLVAAVGGAAVGATSFALYSLGKPGLVARRDGG-DLWEDVDPERPQKL 62

+ K+KELIPLV + AA GA+SFA+YSL K ++ R G + WE VDP PQKL

Sbjct 3 FFQFLRKNKELIPLVVFMSLAASGASSFAVYSLRKTDVIIDRKGNPEPWETVDPTVPQKL 62

Query 63 LTVHQLWRAIPELEEVRR 80

+T++Q W+ I EL++V+R

Sbjct 63 ITINQEWKPIEELQKVQR 80

>XP_019612223.1 PREDICTED: normal mucosa of esophagus-specific gene 1 protein

[Rhinolophus sinicus]

Length=83

Score = 77.8 bits (190), Expect = 7e-17, Method: Compositional matrix adjust.

Identities = 38/78 (49%), Positives = 54/78 (69%), Gaps = 1/78 (1%)

Query 4 LLTLIGKHKELIPLVAAVGGAAVGATSFALYSLGKPGLVA-RRDGGDLWEDVDPERPQKL 62

L ++ K KELIPLV + AA GA+SFA+YSL K ++ R+ + WE VDP P+KL

Sbjct 3 LFQMLRKKKELIPLVVIMTVAASGASSFAVYSLKKTDVILDRKRNPEPWESVDPNVPRKL 62

Query 63 LTVHQLWRAIPELEEVRR 80

+T++Q W+ I EL++VRR

Sbjct 63 ITINQEWKPIEELQKVRR 80

>XP_004639087.1 normal mucosa of esophagus-specific gene 1 protein [Octodon degus]

XP_023579860.1 normal mucosa of esophagus-specific gene 1 protein [Octodon degus]

XP_023579861.1 normal mucosa of esophagus-specific gene 1 protein [Octodon degus]

XP_023579862.1 normal mucosa of esophagus-specific gene 1 protein [Octodon degus]

Length=83

Score = 77.8 bits (190), Expect = 7e-17, Method: Compositional matrix adjust.

Identities = 38/78 (49%), Positives = 53/78 (68%), Gaps = 1/78 (1%)

Query 4 LLTLIGKHKELIPLVAAVGGAAVGATSFALYSLGKPG-LVARRDGGDLWEDVDPERPQKL 62

+L ++ K KELIPL V AA GA+SFA+YS K ++ R+ + WE VDP PQKL

Sbjct 3 VLQILMKKKELIPLALFVTMAACGASSFAVYSFQKTDVIIDRKRNPEPWETVDPNIPQKL 62

Query 63 LTVHQLWRAIPELEEVRR 80

+T++Q W+ I EL++VRR

Sbjct 63 ITINQEWKPIEELQKVRR 80

>XP_009895563.1 PREDICTED: normal mucosa of esophagus-specific gene 1 protein

[Picoides pubescens]

Length=86

Score = 77.8 bits (190), Expect = 8e-17, Method: Compositional matrix adjust.

Identities = 35/84 (42%), Positives = 55/84 (65%), Gaps = 2/84 (2%)

Query 2 ARLLTLIGKHKELIPLVAAVGGAAVGATSFALYSL-GKPGLVARRDGG-DLWEDVDPERP 59

++ ++ KELIPL + AA+GA SF++YSL K ++ + G + W+ +DP +P

Sbjct 3 SKFFQILKSKKELIPLAGILSFAALGALSFSVYSLFSKSDVIINKSGNPEPWQTIDPTKP 62

Query 60 QKLLTVHQLWRAIPELEEVRRIER 83

QKLLTVHQ W+ I ELE V+++ +

Sbjct 63 QKLLTVHQKWKPIEELENVKKLTK 86

>XP_011755367.1 normal mucosa of esophagus-specific gene 1 protein [Macaca nemestrina]

EHH27292.1 Protein FOAP-11 [Macaca mulatta]

Length=83

Score = 77.4 bits (189), Expect = 8e-17, Method: Compositional matrix adjust.

Identities = 40/78 (51%), Positives = 53/78 (68%), Gaps = 1/78 (1%)

Query 4 LLTLIGKHKELIPLVAAVGGAAVGATSFALYSLGKPGLVA-RRDGGDLWEDVDPERPQKL 62

L+ K KELIPLV V AA GA+SFA+YSL K ++ R+ + WE VDP PQKL

Sbjct 3 FFQLLMKRKELIPLVLFVSVAAGGASSFAVYSLWKTDVILDRKRNPEPWETVDPTIPQKL 62

Query 63 LTVHQLWRAIPELEEVRR 80

+T++Q W+ I EL++VRR

Sbjct 63 ITINQQWKPIEELQKVRR 80

>XP_006889193.1 PREDICTED: normal mucosa of esophagus-specific gene 1 protein-like

[Elephantulus edwardii]

Length=103

Score = 78.2 bits (191), Expect = 8e-17, Method: Compositional matrix adjust.

Identities = 39/84 (46%), Positives = 53/84 (63%), Gaps = 1/84 (1%)

Query 1 MARLLTLIGKHKELIPLVAAVGGAAVGATSFALYSLGKPGLV-ARRDGGDLWEDVDPERP 59

+ L+ K KELIPLV + A GA+SFA+YSL K ++ R+ + WEDVDP P

Sbjct 20 ITNFFQLLMKKKELIPLVLFMTMAGTGASSFAVYSLRKSDMIINRKRNPEPWEDVDPTVP 79

Query 60 QKLLTVHQLWRAIPELEEVRRIER 83

QKL+T+ Q W+ I EL+ VR+ R

Sbjct 80 QKLITIKQEWKPIEELQMVRKATR 103

>XP_003900934.1 normal mucosa of esophagus-specific gene 1 protein [Papio anubis]

Length=83

Score = 77.4 bits (189), Expect = 8e-17, Method: Compositional matrix adjust.

Identities = 40/78 (51%), Positives = 53/78 (68%), Gaps = 1/78 (1%)

Query 4 LLTLIGKHKELIPLVAAVGGAAVGATSFALYSLGKPGLVA-RRDGGDLWEDVDPERPQKL 62

L+ K KELIPLV V AA GA+SFA+YSL K ++ R+ + WE VDP PQKL

Sbjct 3 FFQLLMKRKELIPLVLFVSVAAGGASSFAVYSLRKTDVILDRKRNPEPWETVDPTIPQKL 62

Query 63 LTVHQLWRAIPELEEVRR 80

+T++Q W+ I EL++VRR

Sbjct 63 ITINQQWKPIEELQKVRR 80

>XP_006076379.1 PREDICTED: normal mucosa of esophagus-specific gene 1 protein

[Bubalus bubalis]

Length=83

Score = 77.4 bits (189), Expect = 8e-17, Method: Compositional matrix adjust.

Identities = 39/81 (48%), Positives = 54/81 (67%), Gaps = 1/81 (1%)

Query 4 LLTLIGKHKELIPLVAAVGGAAVGATSFALYSLGKPGLVA-RRDGGDLWEDVDPERPQKL 62

L+ K KELIPLV + AA GA+SFA+YSL K ++ R+ + WE+VDP P KL

Sbjct 3 FFQLLMKKKELIPLVFFMTVAAAGASSFAMYSLRKSDVILDRKRNPEPWENVDPTVPTKL 62

Query 63 LTVHQLWRAIPELEEVRRIER 83

+T++Q W+ I EL++VRR R

Sbjct 63 VTINQEWKPIEELQKVRRATR 83

>XP_020029106.1 normal mucosa of esophagus-specific gene 1 protein [Castor canadensis]

XP_020029107.1 normal mucosa of esophagus-specific gene 1 protein [Castor canadensis]

Length=83

Score = 77.4 bits (189), Expect = 9e-17, Method: Compositional matrix adjust.

Identities = 39/75 (52%), Positives = 52/75 (69%), Gaps = 1/75 (1%)

Query 10 KHKELIPLVAAVGGAAVGATSFALYSLGKPG-LVARRDGGDLWEDVDPERPQKLLTVHQL 68

K KELIPL + AA GA+SFALYSL K +V R+ + WE VDP PQKL+T++Q

Sbjct 9 KKKELIPLAVIMTVAAGGASSFALYSLKKTDVIVDRKRNPEPWETVDPTVPQKLITINQQ 68

Query 69 WRAIPELEEVRRIER 83

W+ + EL++VRR+ R

Sbjct 69 WKPVEELQKVRRVTR 83

>NP_115789.1 normal mucosa of esophagus-specific gene 1 protein [Homo sapiens]

NP_922946.1 normal mucosa of esophagus-specific gene 1 protein [Homo sapiens]

NP_001231606.1 normal mucosa of esophagus-specific gene 1 protein [Pan troglodytes]

XP_003831513.1 normal mucosa of esophagus-specific gene 1 protein [Pan paniscus]

XP_014196971.1 normal mucosa of esophagus-specific gene 1 protein [Pan paniscus]

Q9C002.1 RecName: Full=Normal mucosa of esophagus-specific gene 1 protein;

AltName: Full=Protein FOAP-11

AAK00708.1 normal mucosa of esophagus specific 1 [Homo sapiens]

BAB61021.1 FOAP-11 protein [Homo sapiens]

AAH21173.1 Chromosome 15 open reading frame 48 [Homo sapiens]

EAW77312.1 chromosome 15 open reading frame 48, isoform CRA_a [Homo sapiens]

EAW77313.1 chromosome 15 open reading frame 48, isoform CRA_a [Homo sapiens]

ABM82120.1 chromosome 15 open reading frame 48 [synthetic construct]

ABM85294.1 chromosome 15 open reading frame 48, partial [synthetic construct]

AIC52577.1 C15orf48, partial [synthetic construct]

PNI75061.1 C15orf48 isoform 2 [Pan troglodytes]

PNI75062.1 C15orf48 isoform 3 [Pan troglodytes]

Length=83

Score = 77.4 bits (189), Expect = 9e-17, Method: Compositional matrix adjust.

Identities = 38/81 (47%), Positives = 54/81 (67%), Gaps = 1/81 (1%)

Query 4 LLTLIGKHKELIPLVAAVGGAAVGATSFALYSLGKPGLVA-RRDGGDLWEDVDPERPQKL 62

L+ K KELIPLV + AA GA+SFA+YSL K ++ R+ + WE VDP PQKL

Sbjct 3 FFQLLMKRKELIPLVVFMTVAAGGASSFAVYSLWKTDVILDRKKNPEPWETVDPTVPQKL 62

Query 63 LTVHQLWRAIPELEEVRRIER 83

+T++Q W+ I EL+ V+R+ +

Sbjct 63 ITINQQWKPIEELQNVQRVTK 83

>KFP56096.1 Normal mucosa of esophagus-specific 1, partial [Cariama cristata]

Length=73

Score = 77.0 bits (188), Expect = 9e-17, Method: Compositional matrix adjust.

Identities = 37/73 (51%), Positives = 51/73 (70%), Gaps = 2/73 (3%)

Query 13 ELIPLVAAVGGAAVGATSFALYSL-GKPGLVARRDGG-DLWEDVDPERPQKLLTVHQLWR 70

+LIPLV V AAVGA SF++YS+ K ++ + G + WE VDP +PQKLLT+HQ W+

Sbjct 1 QLIPLVGVVSFAAVGALSFSVYSIFSKSDVIINKSGNPEPWETVDPTKPQKLLTIHQKWK 60

Query 71 AIPELEEVRRIER 83

I ELE VR++ +

Sbjct 61 PIEELENVRKLTK 73

>XP_007116783.1 normal mucosa of esophagus-specific gene 1 protein [Physeter

catodon]

XP_023974239.1 normal mucosa of esophagus-specific gene 1 protein [Physeter

catodon]

Length=83

Score = 77.4 bits (189), Expect = 1e-16, Method: Compositional matrix adjust.

Identities = 40/81 (49%), Positives = 52/81 (64%), Gaps = 1/81 (1%)

Query 4 LLTLIGKHKELIPLVAAVGGAAVGATSFALYSLGKPG-LVARRDGGDLWEDVDPERPQKL 62

L+ K KELIPLV AA GA SFALYSL K ++ R+ + WE VDP P+KL

Sbjct 3 FFQLLMKRKELIPLVLFTTVAATGALSFALYSLRKTDVIIDRKRNPEPWETVDPTAPRKL 62

Query 63 LTVHQLWRAIPELEEVRRIER 83

+T++Q W+ I EL+EVR+ R

Sbjct 63 ITINQEWKPIEELQEVRKATR 83

>XP_005889441.1 PREDICTED: normal mucosa of esophagus-specific gene 1 protein

[Bos mutus]

ELR60979.1 Normal mucosa of esophagus-specific 1 protein [Bos mutus]

Length=83

Score = 77.4 bits (189), Expect = 1e-16, Method: Compositional matrix adjust.

Identities = 40/81 (49%), Positives = 54/81 (67%), Gaps = 1/81 (1%)

Query 4 LLTLIGKHKELIPLVAAVGGAAVGATSFALYSLGKPGLVA-RRDGGDLWEDVDPERPQKL 62

L+ K KELIPLV + AAVGA+SFA+YSL K ++ R+ + WE VDP P KL

Sbjct 3 FFQLLVKKKELIPLVFFMTVAAVGASSFAVYSLRKSDVILDRKRNPEPWETVDPTVPTKL 62

Query 63 LTVHQLWRAIPELEEVRRIER 83

+T++Q W+ I EL++VRR R

Sbjct 63 VTINQEWKPIEELQKVRRATR 83

>XP_002927945.1 PREDICTED: normal mucosa of esophagus-specific gene 1 protein

[Ailuropoda melanoleuca]

Length=83

Score = 77.4 bits (189), Expect = 1e-16, Method: Compositional matrix adjust.

Identities = 38/78 (49%), Positives = 53/78 (68%), Gaps = 1/78 (1%)

Query 4 LLTLIGKHKELIPLVAAVGGAAVGATSFALYSLGKPG-LVARRDGGDLWEDVDPERPQKL 62

+ L+ K KELIPLV + AA GA+SFA+YSL K ++ R+ + WE VDP P+KL

Sbjct 3 IFQLLRKKKELIPLVLIMTTAAAGASSFAVYSLQKTDVIIDRKRNPEPWETVDPNVPRKL 62

Query 63 LTVHQLWRAIPELEEVRR 80

LT++Q W+ I EL++ RR

Sbjct 63 LTINQQWKPIEELQKARR 80

>XP_003266917.1 PREDICTED: normal mucosa of esophagus-specific gene 1 protein

[Nomascus leucogenys]

XP_012363002.1 PREDICTED: normal mucosa of esophagus-specific gene 1 protein

[Nomascus leucogenys]

Length=83

Score = 77.4 bits (189), Expect = 1e-16, Method: Compositional matrix adjust.

Identities = 39/79 (49%), Positives = 54/79 (68%), Gaps = 1/79 (1%)

Query 4 LLTLIGKHKELIPLVAAVGGAAVGATSFALYSLGKPGLVA-RRDGGDLWEDVDPERPQKL 62

L+ K KELIPLV V AA GA+SFA+YSL K ++ R+ + WE VDP PQKL

Sbjct 3 FFQLLMKRKELIPLVVFVSVAAGGASSFAVYSLWKTDVILDRKRNPEPWETVDPTIPQKL 62

Query 63 LTVHQLWRAIPELEEVRRI 81

+T++Q W+ I EL++V+R+

Sbjct 63 ITINQRWKPIEELQKVQRV 81

>KFW03699.1 Normal mucosa of esophagus-specific 1, partial [Fulmarus glacialis]

Length=73

Score = 77.0 bits (188), Expect = 1e-16, Method: Compositional matrix adjust.

Identities = 38/73 (52%), Positives = 50/73 (68%), Gaps = 2/73 (3%)

Query 13 ELIPLVAAVGGAAVGATSFALYSL-GKPGLVARRDGG-DLWEDVDPERPQKLLTVHQLWR 70

+LIPLV V AAVGA SF+ YSL K ++ + G + WE VDP +PQKLLT+HQ W+

Sbjct 1 QLIPLVGVVSFAAVGAFSFSAYSLFSKSDVIINKSGNPEPWETVDPTKPQKLLTIHQKWK 60

Query 71 AIPELEEVRRIER 83

I ELE VR++ +

Sbjct 61 PIEELENVRKLTK 73

>XP_009992763.1 PREDICTED: normal mucosa of esophagus-specific gene 1 protein

[Chaetura pelagica]

Length=86

Score = 77.4 bits (189), Expect = 1e-16, Method: Compositional matrix adjust.

Identities = 38/84 (45%), Positives = 52/84 (62%), Gaps = 2/84 (2%)

Query 2 ARLLTLIGKHKELIPLVAAVGGAAVGATSFALYSL-GKPGLVARRDGG-DLWEDVDPERP 59

++ KELIPLV V A VGA SF +YSL K +V + G + WE VDP +P

Sbjct 3 TSFFQMLRTKKELIPLVGVVSCAGVGAFSFCIYSLFCKSDVVINKSGNPEPWETVDPAKP 62

Query 60 QKLLTVHQLWRAIPELEEVRRIER 83

QKLLT++Q W+ I ELE V+++ +

Sbjct 63 QKLLTINQKWKPIEELEHVKKLTK 86

>KFV43323.1 Normal mucosa of esophagus-specific 1, partial [Gavia stellata]

Length=73

Score = 76.6 bits (187), Expect = 1e-16, Method: Compositional matrix adjust.

Identities = 38/73 (52%), Positives = 50/73 (68%), Gaps = 2/73 (3%)

Query 13 ELIPLVAAVGGAAVGATSFALYSL-GKPGLVARRDGG-DLWEDVDPERPQKLLTVHQLWR 70

+LIPLV V AAVGA SF+ YSL K ++ + G + WE VDP +PQKLLT+HQ W+

Sbjct 1 QLIPLVGVVSFAAVGALSFSAYSLFSKSDVIINKSGNPEPWETVDPTKPQKLLTIHQKWK 60

Query 71 AIPELEEVRRIER 83

I ELE VR++ +

Sbjct 61 PIEELENVRKLTK 73

>XP_023596413.1 normal mucosa of esophagus-specific gene 1 protein isoform X2

[Trichechus manatus latirostris]

Length=83

Score = 77.0 bits (188), Expect = 1e-16, Method: Compositional matrix adjust.

Identities = 41/81 (51%), Positives = 51/81 (63%), Gaps = 1/81 (1%)

Query 4 LLTLIGKHKELIPLVAAVGGAAVGATSFALYSLGKPG-LVARRDGGDLWEDVDPERPQKL 62

L+ K KELIPLV AA GA+SFALYSL K +V R+ + WE VDP PQKL

Sbjct 3 FFQLLMKKKELIPLVLITSVAAGGASSFALYSLRKTDVIVDRKRNPEPWEAVDPSVPQKL 62

Query 63 LTVHQLWRAIPELEEVRRIER 83

+T++Q WR I EL+ R+ R

Sbjct 63 ITINQQWRPIEELQSARKASR 83

>XP_004281317.1 PREDICTED: normal mucosa of esophagus-specific gene 1 protein

[Orcinus orca]

XP_004324616.2 PREDICTED: normal mucosa of esophagus-specific gene 1 protein

[Tursiops truncatus]

Length=83

Score = 76.6 bits (187), Expect = 2e-16, Method: Compositional matrix adjust.

Identities = 38/81 (47%), Positives = 52/81 (64%), Gaps = 1/81 (1%)

Query 4 LLTLIGKHKELIPLVAAVGGAAVGATSFALYSLGKPG-LVARRDGGDLWEDVDPERPQKL 62

++ K KELIPL AA GA SFALYSL KP ++ R+ + WE VDP P+KL

Sbjct 3 FFQVLMKRKELIPLAFFTTVAATGALSFALYSLRKPDVIIDRKRNPEPWETVDPTAPRKL 62

Query 63 LTVHQLWRAIPELEEVRRIER 83

+T++Q W+ I EL++VR+ R

Sbjct 63 ITINQEWKPIEELQQVRKATR 83

>KFP74640.1 Normal mucosa of esophagus-specific 1, partial [Apaloderma vittatum]

Length=73

Score = 76.3 bits (186), Expect = 2e-16, Method: Compositional matrix adjust.

Identities = 36/73 (49%), Positives = 50/73 (68%), Gaps = 2/73 (3%)

Query 13 ELIPLVAAVGGAAVGATSFALYSL-GKPGLVARRDGG-DLWEDVDPERPQKLLTVHQLWR 70

+LIPLV V AAVG+ SF +YSL K ++ + G + WE +DP +PQKLLT+HQ W+

Sbjct 1 QLIPLVGVVSFAAVGSFSFCIYSLFSKSDVIINKTGNPEPWETIDPAKPQKLLTIHQKWK 60

Query 71 AIPELEEVRRIER 83

I ELE VR++ +

Sbjct 61 PIEELENVRKLTK 73

>XP_014636168.1 PREDICTED: normal mucosa of esophagus-specific gene 1 protein

[Ceratotherium simum simum]

Length=116

Score = 77.4 bits (189), Expect = 2e-16, Method: Compositional matrix adjust.

Identities = 40/81 (49%), Positives = 52/81 (64%), Gaps = 1/81 (1%)

Query 1 MARLLTLIGKHKELIPLVAAVGGAAVGATSFALYSLGKPG-LVARRDGGDLWEDVDPERP 59

+ L+ K KELIPLVA + AA GA SFALYSL K ++ R+ + WE VDP P

Sbjct 33 IMNFFQLLRKKKELIPLVAFMSVAATGAVSFALYSLKKTDVIIDRKRNPEPWETVDPSVP 92

Query 60 QKLLTVHQLWRAIPELEEVRR 80

KL+T++Q W+ I EL+ VRR

Sbjct 93 GKLITINQEWKPIEELQNVRR 113

>XP_021500755.1 normal mucosa of esophagus-specific gene 1 protein [Meriones

unguiculatus]

Length=83

Score = 76.3 bits (186), Expect = 2e-16, Method: Compositional matrix adjust.

Identities = 38/81 (47%), Positives = 53/81 (65%), Gaps = 1/81 (1%)

Query 4 LLTLIGKHKELIPLVAAVGGAAVGATSFALYSLGKPGLVA-RRDGGDLWEDVDPERPQKL 62

+ + K+KELIPL + AA GA SF LY+L K +V R+ + WE VDP +PQKL

Sbjct 3 IFQTLMKNKELIPLALIISTAATGALSFGLYALKKTDVVLDRKKNPEPWEMVDPTQPQKL 62

Query 63 LTVHQLWRAIPELEEVRRIER 83

+T++Q W+ I EL++VRR R

Sbjct 63 ITINQEWKPIEELQKVRRATR 83

>XP_022442896.1 normal mucosa of esophagus-specific gene 1 protein [Delphinapterus

leucas]

Length=83

Score = 76.3 bits (186), Expect = 2e-16, Method: Compositional matrix adjust.

Identities = 39/81 (48%), Positives = 52/81 (64%), Gaps = 1/81 (1%)

Query 4 LLTLIGKHKELIPLVAAVGGAAVGATSFALYSLGKPG-LVARRDGGDLWEDVDPERPQKL 62

L+ K KELIPLV AA GA SFALYSL K ++ R+ + WE VDP P+KL

Sbjct 3 FFQLMMKRKELIPLVLFTTVAATGALSFALYSLRKTDVIIDRKRNPEPWETVDPTAPRKL 62

Query 63 LTVHQLWRAIPELEEVRRIER 83

+T++Q W+ I EL++VR+ R

Sbjct 63 ITINQEWKPIEELQQVRKATR 83

>XP_007081828.1 PREDICTED: normal mucosa of esophagus-specific gene 1 protein

[Panthera tigris altaica]

XP_014939936.1 PREDICTED: normal mucosa of esophagus-specific gene 1 protein

[Acinonyx jubatus]

XP_014939937.1 PREDICTED: normal mucosa of esophagus-specific gene 1 protein

[Acinonyx jubatus]

XP_019297788.1 PREDICTED: normal mucosa of esophagus-specific gene 1 protein

[Panthera pardus]

Length=83

Score = 76.3 bits (186), Expect = 2e-16, Method: Compositional matrix adjust.

Identities = 38/79 (48%), Positives = 53/79 (67%), Gaps = 1/79 (1%)

Query 3 RLLTLIGKHKELIPLVAAVGGAAVGATSFALYSLGKPG-LVARRDGGDLWEDVDPERPQK 61

L L+ K KELIPLV + AA GA++FA+YSL K ++ R+ + WE VDP P K

Sbjct 2 NLFQLLRKKKELIPLVLMMTTAAGGASAFAVYSLQKTDVIIDRKKNPEPWETVDPNVPSK 61

Query 62 LLTVHQLWRAIPELEEVRR 80

L+T++Q W+ I EL++VRR

Sbjct 62 LITINQQWKPIEELQKVRR 80

>XP_004386338.2 normal mucosa of esophagus-specific gene 1 protein isoform X1

[Trichechus manatus latirostris]

Length=98

Score = 76.6 bits (187), Expect = 2e-16, Method: Compositional matrix adjust.

Identities = 41/81 (51%), Positives = 51/81 (63%), Gaps = 1/81 (1%)

Query 4 LLTLIGKHKELIPLVAAVGGAAVGATSFALYSLGKPG-LVARRDGGDLWEDVDPERPQKL 62

L+ K KELIPLV AA GA+SFALYSL K +V R+ + WE VDP PQKL

Sbjct 18 FFQLLMKKKELIPLVLITSVAAGGASSFALYSLRKTDVIVDRKRNPEPWEAVDPSVPQKL 77

Query 63 LTVHQLWRAIPELEEVRRIER 83

+T++Q WR I EL+ R+ R

Sbjct 78 ITINQQWRPIEELQSARKASR 98

>XP_012297828.1 normal mucosa of esophagus-specific gene 1 protein [Aotus nancymaae]

Length=83

Score = 76.3 bits (186), Expect = 2e-16, Method: Compositional matrix adjust.

Identities = 37/77 (48%), Positives = 52/77 (68%), Gaps = 1/77 (1%)

Query 4 LLTLIGKHKELIPLVAAVGGAAVGATSFALYSLGKPGLVARRDGG-DLWEDVDPERPQKL 62

+ K+KEL+PLV + AA GA+SFA+YSL K ++ R G + WE VDP PQKL

Sbjct 3 FFQFLKKNKELVPLVVFMTLAASGASSFAVYSLRKTDVIIDRKGNPEPWETVDPTVPQKL 62

Query 63 LTVHQLWRAIPELEEVR 79

+T++Q W+ I EL++VR

Sbjct 63 VTINQEWKPIEELQKVR 79

>XP_024617130.1 normal mucosa of esophagus-specific gene 1 protein [Neophocaena

asiaeorientalis asiaeorientalis]

XP_024617131.1 normal mucosa of esophagus-specific gene 1 protein [Neophocaena

asiaeorientalis asiaeorientalis]

Length=83

Score = 76.3 bits (186), Expect = 3e-16, Method: Compositional matrix adjust.

Identities = 39/81 (48%), Positives = 52/81 (64%), Gaps = 1/81 (1%)

Query 4 LLTLIGKHKELIPLVAAVGGAAVGATSFALYSLGKPG-LVARRDGGDLWEDVDPERPQKL 62

L+ K KELIPLV AA GA SFALYSL K ++ R+ + WE VDP P+KL

Sbjct 3 FFQLLMKRKELIPLVLFTTVAATGALSFALYSLRKTDVIIDRKRNPEPWETVDPTAPRKL 62

Query 63 LTVHQLWRAIPELEEVRRIER 83

+T++Q W+ I EL++VR+ R

Sbjct 63 ITINQEWKPIEELQQVRKATR 83

>XP_010630801.1 PREDICTED: normal mucosa of esophagus-specific gene 1 protein

[Fukomys damarensis]

XP_010630802.1 PREDICTED: normal mucosa of esophagus-specific gene 1 protein

[Fukomys damarensis]

XP_010630803.1 PREDICTED: normal mucosa of esophagus-specific gene 1 protein

[Fukomys damarensis]

XP_010630804.1 PREDICTED: normal mucosa of esophagus-specific gene 1 protein

[Fukomys damarensis]

XP_010630806.1 PREDICTED: normal mucosa of esophagus-specific gene 1 protein

[Fukomys damarensis]

XP_010630807.1 PREDICTED: normal mucosa of esophagus-specific gene 1 protein

[Fukomys damarensis]

Length=83

Score = 76.3 bits (186), Expect = 3e-16, Method: Compositional matrix adjust.

Identities = 37/78 (47%), Positives = 53/78 (68%), Gaps = 1/78 (1%)

Query 4 LLTLIGKHKELIPLVAAVGGAAVGATSFALYSLGKPGLVARRDGG-DLWEDVDPERPQKL 62

+ ++ K KELIPL + AA GA+SFA+YS K ++ R G + WE+VDP PQKL

Sbjct 3 VFQILMKKKELIPLAVIMTVAAGGASSFAVYSFCKSDVIIDRKGNPEPWENVDPSVPQKL 62

Query 63 LTVHQLWRAIPELEEVRR 80

LT++Q W+ I EL++VR+

Sbjct 63 LTINQEWKPIEELQKVRK 80

>XP_008014946.1 PREDICTED: normal mucosa of esophagus-specific gene 1 protein

[Chlorocebus sabaeus]

XP_008014947.1 PREDICTED: normal mucosa of esophagus-specific gene 1 protein

[Chlorocebus sabaeus]

Length=83

Score = 76.3 bits (186), Expect = 3e-16, Method: Compositional matrix adjust.

Identities = 39/78 (50%), Positives = 53/78 (68%), Gaps = 1/78 (1%)

Query 4 LLTLIGKHKELIPLVAAVGGAAVGATSFALYSLGKPGLVA-RRDGGDLWEDVDPERPQKL 62

L+ K KELIPLV V AA GA+SFA+YSL K ++ R+ + WE VDP PQKL

Sbjct 3 FFQLLMKRKELIPLVLFVSVAAGGASSFAVYSLWKTDVILDRKRNPEPWETVDPTIPQKL 62

Query 63 LTVHQLWRAIPELEEVRR 80

+T++Q W+ I +L++VRR

Sbjct 63 ITINQQWKPIEDLQKVRR 80

>XP_010351257.1 PREDICTED: normal mucosa of esophagus-specific gene 1 protein

[Rhinopithecus roxellana]

XP_017728312.1 PREDICTED: normal mucosa of esophagus-specific gene 1 protein

[Rhinopithecus bieti]

Length=83

Score = 76.3 bits (186), Expect = 3e-16, Method: Compositional matrix adjust.

Identities = 38/81 (47%), Positives = 54/81 (67%), Gaps = 1/81 (1%)

Query 4 LLTLIGKHKELIPLVAAVGGAAVGATSFALYSLGKPGLVA-RRDGGDLWEDVDPERPQKL 62

L+ K KELIPLV AA GA+SFA+YSL K ++ R+ + WE VDP PQKL

Sbjct 3 FFQLLMKRKELIPLVLFTSVAAGGASSFAVYSLWKTDVILDRKRNPEPWETVDPTIPQKL 62

Query 63 LTVHQLWRAIPELEEVRRIER 83

+T++Q W+ I EL++V+R+ +

Sbjct 63 ITINQQWKPIEELQKVQRVTK 83

>XP_005559496.1 PREDICTED: normal mucosa of esophagus-specific gene 1 protein

[Macaca fascicularis]

XP_005559497.1 PREDICTED: normal mucosa of esophagus-specific gene 1 protein

[Macaca fascicularis]

EHH63053.1 Protein FOAP-11 [Macaca fascicularis]

Length=83

Score = 75.9 bits (185), Expect = 3e-16, Method: Compositional matrix adjust.

Identities = 39/78 (50%), Positives = 53/78 (68%), Gaps = 1/78 (1%)

Query 4 LLTLIGKHKELIPLVAAVGGAAVGATSFALYSLGKPGLVA-RRDGGDLWEDVDPERPQKL 62

L+ K KELIPLV V AA GA+SFA+YSL K ++ R+ + WE VDP PQKL

Sbjct 3 FFQLLMKRKELIPLVLFVSVAAGGASSFAVYSLWKTDVILDRKRNPEPWETVDPTIPQKL 62

Query 63 LTVHQLWRAIPELEEVRR 80

+T++Q W+ I EL++V+R

Sbjct 63 ITINQQWKPIEELQKVQR 80

>XP_011281153.1 normal mucosa of esophagus-specific gene 1 protein [Felis catus]

XP_019688062.1 normal mucosa of esophagus-specific gene 1 protein [Felis catus]

Length=83

Score = 75.9 bits (185), Expect = 4e-16, Method: Compositional matrix adjust.

Identities = 38/79 (48%), Positives = 53/79 (67%), Gaps = 1/79 (1%)

Query 3 RLLTLIGKHKELIPLVAAVGGAAVGATSFALYSLGKPG-LVARRDGGDLWEDVDPERPQK 61

L L+ K KELIPLV + AA GA++FA+YSL K ++ R+ + WE VDP P K

Sbjct 2 NLFQLLRKKKELIPLVLMMTTAAGGASAFAVYSLQKTDVIIDRKKNPEPWETVDPNVPSK 61

Query 62 LLTVHQLWRAIPELEEVRR 80

L+T++Q W+ I EL++VRR

Sbjct 62 LITINQEWKPIEELQKVRR 80

>KFP93937.1 Normal mucosa of esophagus-specific 1, partial [Haliaeetus albicilla]

Length=73

Score = 75.5 bits (184), Expect = 4e-16, Method: Compositional matrix adjust.

Identities = 37/73 (51%), Positives = 51/73 (70%), Gaps = 2/73 (3%)

Query 13 ELIPLVAAVGGAAVGATSFALYSL-GKPGLVARRDGG-DLWEDVDPERPQKLLTVHQLWR 70

+LIPLV V AAVGA SF++YSL K ++ + G + WE V+P +PQKLLT+HQ W+

Sbjct 1 QLIPLVGVVSFAAVGALSFSVYSLFSKSDVIINKTGNPEPWETVEPTKPQKLLTIHQKWK 60

Query 71 AIPELEEVRRIER 83

I ELE VR++ +

Sbjct 61 PIEELESVRKLTK 73

>XP_004709465.1 PREDICTED: normal mucosa of esophagus-specific gene 1 protein

[Echinops telfairi]

Length=83

Score = 75.9 bits (185), Expect = 4e-16, Method: Compositional matrix adjust.

Identities = 37/78 (47%), Positives = 52/78 (67%), Gaps = 1/78 (1%)

Query 4 LLTLIGKHKELIPLVAAVGGAAVGATSFALYSLGKPGLVA-RRDGGDLWEDVDPERPQKL 62

L+ K KELIPL + AA GA+SFA+YSL K ++ R++ + WE VDP PQKL

Sbjct 3 FFRLLMKKKELIPLALIMTVAASGASSFAVYSLRKSDVITDRKNNPEPWETVDPTVPQKL 62

Query 63 LTVHQLWRAIPELEEVRR 80

+TV+Q W+ I EL++ R+

Sbjct 63 ITVNQQWKPIEELQKARK 80

>XP_008141320.1 PREDICTED: normal mucosa of esophagus-specific gene 1 protein

[Eptesicus fuscus]

Length=84

Score = 75.9 bits (185), Expect = 4e-16, Method: Compositional matrix adjust.

Identities = 39/83 (47%), Positives = 53/83 (64%), Gaps = 2/83 (2%)

Query 3 RLLTLIGKHKELIPLVAAVGGAAVGATSFALYSLGKPG--LVARRDGGDLWEDVDPERPQ 60

L L+ K KELIPL + AA GA+SFA+YSL K ++ R+ + WE VDP P

Sbjct 2 NLFQLLMKKKELIPLALIMTVAASGASSFAVYSLNKKSDVIIDRKGNPEPWETVDPSVPG 61

Query 61 KLLTVHQLWRAIPELEEVRRIER 83

KL+T++Q W+ I EL++VRR R

Sbjct 62 KLITINQQWKPIEELQKVRRATR 84

>NP_001193905.1 normal mucosa of esophagus-specific gene 1 protein [Bos taurus]

XP_010846057.1 PREDICTED: normal mucosa of esophagus-specific gene 1 protein

[Bison bison bison]

XP_019824461.1 PREDICTED: normal mucosa of esophagus-specific gene 1 protein

[Bos indicus]

AAI42380.1 MGC165862 protein [Bos taurus]

Length=83

Score = 75.5 bits (184), Expect = 4e-16, Method: Compositional matrix adjust.

Identities = 39/81 (48%), Positives = 53/81 (65%), Gaps = 1/81 (1%)

Query 4 LLTLIGKHKELIPLVAAVGGAAVGATSFALYSLGKPGLVA-RRDGGDLWEDVDPERPQKL 62

L+ K KELIPLV + AA GA+SFA+YSL K ++ R+ + WE VDP P KL

Sbjct 3 FFQLLMKKKELIPLVFFMTVAAAGASSFAVYSLRKSDVILDRKRNPEPWETVDPTVPTKL 62

Query 63 LTVHQLWRAIPELEEVRRIER 83

+T++Q W+ I EL++VRR R

Sbjct 63 VTINQEWKPIEELQKVRRATR 83

Database: All non-redundant GenBank CDS translations+PDB+SwissProt+PIR+PRF

excluding environmental samples from WGS projects

Posted date: May 25, 2018 10:40 AM

Number of letters in database: 55,783,618,688

Number of sequences in database: 152,239,973

Lambda K H

0.319 0.140 0.420

Gapped

Lambda K H

0.267 0.0410 0.140

Matrix: BLOSUM62

Gap Penalties: Existence: 11, Extension: 1

Number of Sequences: 152239973

Number of Hits to DB: 13679937

Number of extensions: 12837

Number of successful extensions: 3929

Number of sequences better than 100: 32

Number of HSP's better than 100 without gapping: 0

Number of HSP's gapped: 3914

Number of HSP's successfully gapped: 32

Length of query: 91

Length of database: 55783618688

Length adjustment: 61

Effective length of query: 30

Effective length of database: 46496980335

Effective search space: 1394909410050

Effective search space used: 1394909410050

T: 21

A: 40

X1: 16 (7.4 bits)

X2: 38 (14.6 bits)

X3: 64 (24.7 bits)

S1: 41 (20.4 bits)

S2: 76 (33.9 bits)

ka-blk-alpha gapped: 1.9

ka-blk-alpha ungapped: 0.7916

ka-blk-alpha_v gapped: 42.6028

ka-blk-alpha_v ungapped: 4.96466

ka-blk-sigma gapped: 43.6362

**2) Summary of *MISTRAV* PAML NSsites analysis, related to Figure 2**

**A. Log Likelihood Scores and Parameter Estimates for Four Models of Variable *ω*'s Among Sites Assuming** the F3x4 Model of Codon Frequencies

| Model |  | Parameter Estimates | Sites* with *ω* > 1 |  |
| --- | --- | --- | --- | --- |
| M0: one ratio |  | average dN/dS for each branch = 1.111 |  | **-773.434** |
| **Site models** |  |  |  |  |
| M1: neutral |  | (*ω*_0_ = 0) *f*_0_ = 0.477 | Not allowed | **-765.916** |
| M2: selection |  | (*ω*_0_ = 0) *f*_0_ = 0.490  (*ω*_1_ = 1) *f*_1_ = 0.305  ***ω*_2_ = 3.907** ( *f*_2_ = 0.206)  average dN/dS for each branch = 1.107 | 21T 0.970, 46K 0.969, **79Q 0.996** | **-759.184** |
| M3:discrete |  | *ω*_0_ = 0.000 *f*_0_ = 0.566  *ω*_1_ = 2.264 (*f*_1_ = 0.408)  ***ω*_2_ = 9.723 ( *f*_2_ = 0.026)**  average dN/dS for each branch = 1.179 |  | **-758.796** |
| M7:Beta |  | *p* = 0.005 *q* = 0.005  average dN/dS for each branch 0.500 | Not allowed | **-765.946** |
| M8:Beta&*ω* |  | *p* = 0.005 *q* = 0.008  *f*_0_ = 0.803  ***ω*_1_ = 4.038 (*f*_1_ =0.197)**  average dN/dS for each branch = 1.118 | 21T 0.969, 46K 0.969, **79Q 0.996** | **-759.184** |

**B. Log Likelihood Scores and Parameter Estimates for Four Models of Variable *ω*'s Among Sites Assuming the F61 Model** of Codon Frequencies

| Model |  | Parameter Estimates | Sites* with *ω* > 1 |  |
| --- | --- | --- | --- | --- |
| M0: one ratio |  | average dN/dS for each branch = 0.8966 |  | **-753.956** |
| **Site models** |  |  |  |  |
| M1: neutral |  | (*ω*_0_ = 0) *f*_0_ = 0.540 | Not allowed | **-741.360** |
|  |  | (*ω*_1_ = 1) (*f*_1_ = 0.460) |  |  |
|  |  | average dN/dS for each branch = 0.460 |  |  |
| M2: selection |  | (*ω*_0_ = 0) *f*_0_ = 0.576  (*ω*_1_ = 1) *f*_1_ = 0.143  ***ω*_2_ = 3.028** ( *f*_2_ = 0.281)  average dN/dS for each branch = 0.993 | 2S 0.959, 21T 0.982, 46K 0.962, **79Q 0.992**, 81V 0.960 | **-735.692** |
| M3:discrete |  | *ω*_0_ = 0.000 *f*_0_ = 0.557  *ω*_1_ = 0.686 (*f*_1_ = 0.138)  ***ω*_2_ = 2.937 ( *f*_2_ = 0.305)**  average dN/dS for each branch = 0.990 |  | **-735.686** |
| M7:Beta |  | *p* = 0.005 *q* = 0.005 | Not allowed | **-741.481** |
|  |  | average dN/dS for each branch = 0.500 |  |  |
| M8:Beta&*ω* |  | *p* = 0.008 *q* = 0.045  *f*_0_ = 0.705  ***ω*_1_ = 2.974 (*f*_1_ = 0.295)**  average dN/dS for each branch = 0.991 | 2S 0.974, 20M 0.951, 21T 0.989, 46K 0.977, 58V 0.967, **79Q 0.996**, 81V 0.976 | **-735.688** |

Note: *p* and *q* are parameters of the beta distribution. *f* is the proportion of sites assigned to an individual *ω* category or to a beta distribution with shape parameters *p* and *q*. The proportion *f*_1_ in parentheses is not a free parameter. Sites assigned to *ω*_1_ are those with posterior probabilities (*P*) > 0.95 , and those with *P* > 0.99 are in bold. Note: analyses conducted using *κ* as a free parameter.

**C. Likelihood ratio test statistics (2****) for models of variable selective pressure among sites.

|  | 2*δ* | df | *P*-value * |
| --- | --- | --- | --- |
| **F3x4** |  |  |  |
| One ratio vs. M3 (*k* = 3) | 118.254 | 4 | ***P* <** 0.0001 |
| M1 vs. M2 | 34.766 | 2 | ***P* <** 0.0012 |
| M7 vs. M8 | 16.026 | 2 | ***P* <** 0.0012 |
| **F61** |  |  |  |
| One ratio vs. M3 (*k* = 3) | 106.004 | 4 | ***P* <** 0.0001 |
| M1 vs. M2 | 35.156 | 2 | ***P* <** 0.0035 |
| M7 vs. M8 | 17.938 | 2 | ***P* <** 0.0031 |

**3) Summary of *MISTR1* PAML NSsites analysis, related to Figure 2**

**A. Log Likelihood Scores and Parameter Estimates for Four Models of Variable *ω*'s Among Sites Assuming** the F3x4 Model of Codon Frequencies

| Model |  | Parameter Estimates | Sites* with *ω* > 1 |  |
| --- | --- | --- | --- | --- |
| M0: one ratio |  | average dN/dS for each branch = 0.597 |  | **-709.162** |
| **Site models** |  |  |  |  |
| M1: neutral |  | (*ω*_0_ = 0) *f*_0_ = 0.622 | Not allowed | **-698.008** |
| M2: selection |  | (*ω*_0_ = 0) *f*_0_ = 0.680  (*ω*_1_ = 1) *f*_1_ = 0.241  ***ω*_2_ = 4.860** ( *f*_2_ = 0.078)  average dN/dS for each branch = 0.679 | **6I 0.996**, 27T 0.987 | **-692.601** |
| M3:discrete |  | *ω*_0_ = 0.145 *f*_0_ = 0.800  *ω*_1_ = 2.14 (*f*_1_ = 0.167)  ***ω*_2_ = 7.439 ( *f*_2_ = 0.033)**  average dN/dS for each branch = 0.719 |  | **-692.922** |
| M7:Beta |  | *p* = 0.005 *q* = 0.007  average dN/dS for each branch 0.400 | Not allowed | **-698.030** |
| M8:Beta&*ω* |  | *p* = 0.114 *q* = 0.231  *f*_0_ = 0.922  ***ω*_1_ = 4.954 (*f*_1_ = 0.078)**  average dN/dS for each branch = 0.690 | **6I 0.997**, 27T 0.989 | **-692.631** |

**B. Log Likelihood Scores and Parameter Estimates for Four Models of Variable ****'s Among Sites Assuming the F61 Model of Codon Frequencies

| Model |  | Parameter Estimates | Sites* with *ω* > 1 |  |
| --- | --- | --- | --- | --- |
| M0: one ratio |  | average dN/dS for each branch = 0.684 |  | **-710.290** |
| **Site models** |  |  |  |  |
| M1: neutral |  | (*ω*_0_ = 0) *f*_0_ = 0.597 | Not allowed | **-699.206** |
|  |  | (*ω*_1_ = 1) (*f*_1_ = 0.403) |  |  |
|  |  | average dN/dS for each branch = 0.403 | **6I 0.995**, **27T 0.991** |  |
| M2: selection |  | (*ω*_0_ = 0) *f*_0_ = 0.629  (*ω*_1_ = 1) *f*_1_ = 0.275  ***ω*_2_ = 4.545** ( *f*_2_ = 0.095)  average dN/dS for each branch = 0.741 |  | **-693.650** |
| M3:discrete |  | *ω*_0_ = 0.104 *f*_0_ = 0.737  *ω*_1_ = 1.916 (*f*_1_ = 0.228)  ***ω*_2_ = 7.417 ( *f*_2_ = 0.035)**  average dN/dS for each branch = 0.774 |  | **-693.350** |
| M7:Beta |  | *p* = 0.005 *q* = 0.007 | Not allowed | **-699.207** |
|  |  | average dN/dS for each branch = 0.400 |  |  |
| M8:Beta&*ω* |  | *p* = 0.057 *q* = 0.109  *f*_0_ = 0.905  ***ω*_1_ = 4.570 (*f*_1_ = 0.095)**  average dN/dS for each branch = 0.746 | **6I 0.996**, **27T 0.991** | **-693.684** |

Note: *p* and *q* are parameters of the beta distribution. *f* is the proportion of sites assigned to an individual *ω* category or to a beta distribution with shape parameters *p* and *q*. The proportion *f*_1_ in parentheses is not a free parameter. Sites assigned to *ω*_1_ are those with posterior probabilities (*P*) > 0.95 , and those with *P* > 0.99 are in bold. Note: analyses conducted using *κ* as a free parameter.

**C. Likelihood ratio test statistics (2****) for models of variable selective pressure among sites.

|  | 2*δ* | df | *P*-value * |
| --- | --- | --- | --- |
| **F3x4** |  |  |  |
| One ratio vs. M3 (*k* = 3) | 118.254 | 4 | ***P* <** 0.0001 |
| M1 vs. M2 | 34.766 | 2 | ***P* <** 0.0045 |
| M7 vs. M8 | 16.026 | 2 | ***P* <** 0.0046 |
| **F61** |  |  |  |
| One ratio vs. M3 (*k* = 3) | 106.004 | 4 | ***P* <** 0.0039 |
| M1 vs. M2 | 35.156 | 2 | ***P* <** 0.0040 |
| M7 vs. M8 | 17.938 | 2 | ***P* <** 0.004 |

**4) Summary of *MISTRH* PAML NSsites analysis, related to Figure 2**

**A. Log Likelihood Scores and Parameter Estimates for Four Models of Variable *ω*'s Among Sites Assuming** the F3x4 Model of Codon Frequencies

| Model |  | Parameter Estimates | Sites* with *ω* > 1 |  |
| --- | --- | --- | --- | --- |
| M0: one ratio |  | average dN/dS for each branch = 0.149 |  | **-645.887** |
| **Site models** |  |  |  |  |
| M1: neutral |  | (*ω*_0_ = 0) *f*_0_ = 0.895 | Not allowed | **-644.150** |
| M2: selection |  | (*ω*_0_ = 0) *f*_0_ = 0.895  (*ω*_1_ = 1) *f*_1_ = 0.059  ***ω*_2_ = 1.000** ( *f*_2_ = 0.047)  average dN/dS for each branch = 0.182 | No Sites Predicted | **-644.150** |
| M3:discrete |  | *ω*_0_ = 0.062 *f*_0_ = 0.271  *ω*_1_ = 0.062 (*f*_1_ = 0.545)  ***ω*_2_ = 0.670 ( *f*_2_ = 0.183)**  average dN/dS for each branch = 0.173 |  | **-644.079** |
| M7:Beta |  | *p* = 0.288 *q* = 1.362  average dN/dS for each branch 0.172 | Not allowed | **-644.115** |
| M8:Beta&*ω* |  | *p* = 0.288 *q* = 1.362  *f*_0_ = 0.999  ***ω*_1_ = 1.000 (*f*_1_ =0.000)**  average dN/dS for each branch = 0.172 | No Sites Predicted | **-644.115** |

**B. Log Likelihood Scores and Parameter Estimates for Four Models of Variable *ω*'s Among Sites Assumin**g the F61 Model of Codon Frequencies

| Model |  | Parameter Estimates | Sites* with *ω* > 1 |  |
| --- | --- | --- | --- | --- |
| M0: one ratio |  | average dN/dS for each branch = 0.161 |  | **-613.578** |
| **Site models** |  |  |  |  |
| M1: neutral |  | (*ω*_0_ = 0) *f*_0_ = 0.834 | Not allowed | **-611.107** |
|  |  | (*ω*_1_ = 1) (*f*_1_ = 0.166) |  |  |
|  |  | average dN/dS for each branch = 0.220 |  |  |
| M2: selection |  | (*ω*_0_ = 0) *f*_0_ = 0.861  (*ω*_1_ = 1) *f*_1_ = 0.000  ***ω*_2_ = 1.179** ( *f*_2_ = 0.139)  average dN/dS for each branch = 0.2278 | 35A 0.96 | **-611.092** |
| M3:discrete |  | *ω*_0_ = 0.075 *f*_0_ 0.419  *ω*_1_ = 0.075 (*f*_1_ = 0.442)  ***ω*_2_ = 1.179 ( *f*_2_ = 0.139)**  average dN/dS for each branch = 0.228 |  | **-611.092** |
| M7:Beta |  | *p* = 0.161 *q* = 0.594 | Not allowed | **-611.226** |
|  |  | average dN/dS for each branch = 0.214 |  |  |
| M8:Beta&*ω* |  | *p* = 8.198 *q* = 99.00  *f*_0_ = 0.863  ***ω*_1_ = 1.185 (*f*_1_ = 0.137)**  average dN/dS for each branch = 0.228 | 35A 0.96 | **-611.094** |

Note: *p* and *q* are parameters of the beta distribution. *f* is the proportion of sites assigned to an individual *ω* category or to a beta distribution with shape parameters *p* and *q*. The proportion *f*_1_ in parentheses is not a free parameter. Sites assigned to *ω*_1_ are those with posterior probabilities (*P*) > 0.95 , and those with *P* > 0.99 are in bold. Note: analyses conducted using *κ* as a free parameter.

**C. Likelihood ratio test statistics (2*δ*) for models of variable sel**ective pressure among sites.

|  | 2*δ* | df | *P*-value * |
| --- | --- | --- | --- |
| **F3x4** |  |  |  |
| One ratio vs. M3 (*k* = 3) | 118.254 | 4 | ***P* <** 0.461 |
| M1 vs. M2 | 34.766 | 2 | ***P* <** 1.000 |
| M7 vs. M8 | 16.026 | 2 | ***P* <** 1.000 |
| **F61** |  |  |  |
| One ratio vs. M3 (*k* = 3) | 106.004 | 4 | ***P* <** 0.290 |
| M1 vs. M2 | 35.156 | 2 | ***P* <** 0.986 |
| M7 vs. M8 | 17.938 | 2 | ***P* <** 0.876 |

**5)** **Primate *MISTRAV*/*C15ORF48* input sequences for evolutionary analysis, related to Figure 2.**

>human

ATGAGCTTTTTCCAACTCCTGATGAAAAGGAAGGAACTCATTCCCTTGGTGGTGTTCATGACTGTGGCGGCGGGTGGAGC

CTCATCTTTCGCTGTGTATTCTCTTTGGAAAACCGATGTGATCCTTGATCGAAAAAAAAATCCAGAACCTTGGGAAACTG

TGGACCCTACTGTACCTCAAAAGCTTATAACAATCAACCAACAATGGAAACCCATTGAAGAGTTGCAAAATGTCCAAAGG

GTGACCAAA

>chimp

ATGAGCTTTTTCCAACTCCTGATGAAAAGGAAGGAACTCATTCCCTTGGTGGTGTTCATGACTGTGGCGGCGGGTGGAGC

CTCATCTTTCGCTGTGTATTCTCTTTGGAAAACCGATGTGATCCTTGATCGAAAAAAAAATCCAGAACCTTGGGAAACTG

TGGACCCTACTGTACCTCAAAAGCTTATAACAATCAACCAACAATGGAAACCCATTGAAGAGTTGCAAAATGTCCAAAGG

GTGACCAAA

>bonobo

ATGAGCTTTTTCCAACTCCTGATGAAAAGGAAGGAACTCATTCCCTTGGTGGTGTTCATGACTGTGGCGGCGGGTGGAGC

CTCATCTTTCGCTGTGTATTCTCTTTGGAAAACCGATGTGATCCTTGATCGAAAAAAAAATCCAGAACCTTGGGAAACTG

TGGACCCTACTGTACCTCAAAAGCTTATAACAATCAACCAACAATGGAAACCCATTGAAGAGTTGCAAAATGTCCAAAGG

GTGACCAAA

>gorilla

ATGAGCTTTTTCCAACTCCTGATGAAAAGGAAGGAACTCATTCCCTTGGTGGTGTTCATGACTGTGGCGGCGGGTGGAGC

CTCATCTTTCGCTGTGTATTCTCTTTGGAAAACCGATGTGATCCTTGATCGAAAAAGAAATCCAGAACCTTGGGAAACTG

TGGACCCTACTGTACCTCAAAAGCTTATAACAATCAACCAACAATGGAAACCCATTGAAGAGTTGCAAAATGTCCAAAGG

GCGACCAAA

>orang

ATGAGCTTTTTCCAACTCCTGATGAAAAGGAAGGAACTCATTCCCTTGGTGGTGTTCGTGACTGTGGCGGCGGGTGGAGC

CTCATCGTTCGCTGTGTATTCTCTTTGGAAAACCGATGTGATCCTTGATCGAAAAAAAAATCCAGAACCTTGGGAAACTG

TGGACCCTACTGTACCTCAAAAGCTTATAACAATCAACCAACAGTGGAAACCCATTGAAGAGTTGCAAAAGGTCCAAAGG

GTGACCAAA

>gibbon

ATGAGCTTTTTCCAACTCCTGATGAAAAGGAAGGAACTCATTCCCTTGGTGGTGTTCGTGAGTGTGGCGGCGGGTGGAGC

CTCATCTTTCGCTGTGTATTCTCTTTGGAAAACCGATGTGATCCTTGATCGAAAAAGAAATCCAGAACCTTGGGAAACTG

TGGACCCTACTATACCTCAAAAGCTTATAACAATCAACCAACGGTGGAAACCCATTGAAGAGTTGCAAAAGGTCCAAAGA

GTGACCATA

>AGM

ATGAGCTTTTTCCAACTCCTGATGAAAAGGAAGGAACTCATTCCCTTGGTGCTGTTCGTGAGTGTGGCGGCGGGTGGAGC

CTCGTCTTTTGCTGTGTATTCTCTTTGGAAAACCGATGTGATCCTTGATCGAAAAAGAAATCCAGAACCTTGGGAAACCG

TGGATCCTACTATACCGCAAAAGCTTATAACAATCAACCAACAATGGAAGCCCATTGAAGACCTGCAAAAGGTCCGAAGG

GCGACCAAA

>crabMac

ATGAGCTTTTTCCAACTCCTGATGAAAAGGAAGGAACTCATTCCCTTGGTGCTGTTCGTGAGTGTGGCGGCGGGTGGAGC

CTCGTCTTTTGCTGTGTATTCTCTTTGGAAAACCGATGTGATCCTTGATCGAAAAAGAAATCCAGAACCTTGGGAAACTG

TGGACCCTACTATACCGCAAAAGCTTATAACAATCAACCAACAATGGAAGCCCATTGAAGAGCTGCAAAAGGTCCAAAGG

GCGACCAAA

>rhesus

ATGAGCTTTTTCCAACTCCTGATGAAAAGGAAGGAACTCATTCCCTTGGTGCTGTTCGTGAGTGTGGCGGCGGGTGGAGC

CTCGTCTTTTGCTGTGTATTCTCTTTGGAAAACCGATGTGATCCTTGATCGAAAAAGAAATCCAGAACCTTGGGAAACTG

TGGACCCTACTATACCGCAAAAGCTTATAACAATCAACCAACAATGGAAGCCCATTGAAGAGCTGCAAAAGGTCCGAAGG

GCGACCAAA

>pigMac

ATGAGCTTTTTCCAACTCCTGATGAAAAGGAAGGAACTCATTCCCTTGGTGCTGTTCGTGAGTGTGGCGGCGGGTGGAGC

CTCGTCTTTTGCTGTGTATTCTCTTTGGAAAACCGATGTGATCCTTGATCGAAAAAGAAATCCAGAACCTTGGGAAACTG

TGGACCCTACTATACCGCAAAAGCTTATAACAATCAACCAACAATGGAAGCCCATTGAAGAGCTGCAAAAGGTCCGAAGG

GCGACCAAA

>baboonH

ATGAGCTTTTTCCAACTCCTGATGAAAAGGAAGGAACTCATTCCCTTGGTGCTGTTCGTGAGTGTGGCGGCGGGTGGAGC

CTCGTCTTTTGCTGTGTATTCTCTTCGGAAAACCGATGTGATCCTTGATCGAAAAAGAAATCCAGAACCTTGGGAAACTG

TGGACCCTACTATACCGCAAAAGCTTATAACAATCAACCAACAATGGAAGCCCATTGAAGAGCTGCAAAAGGTCCGAAGG

GCGACCAAA

>baboonA

ATGAGCTTTTTCCAACTCCTGATGAAAAGGAAGGAACTCATTCCCTTGGTGCTGTTCGTGAGTGTGGCGGCGGGTGGAGC

CTCGTCTTTTGCTGTGTATTCTCTTCGGAAAACCGATGTGATCCTTGATCGAAAAAGAAATCCAGAACCTTGGGAAACTG

TGGACCCTACTATACCGCAAAAGCTTATAACAATCAACCAACAATGGAAGCCCATTGAAGAGCTGCAAAAGGTCCGAAGG

GCGACCAAA

>drill

ATGAGCTTTTTCCAACTCCTGATGAAAAGGAAGGAACTCCTTCCCTTGGTGCTGTTCGTGAGTGTGGCGGCGGGTGGAGC

CTCGTCTTTTGCTGTGTATTCTCTTTGGAAAACCGATGTGATCCTTGATCGAAAAAGAAATCCAGAACCTTGGGAAACTG

TGGACCCTACTATACCGCAAAAGCTTATAACAATCAACCAACAATGGAAGCCCATTGAGGAGCTGCAAAAGGTCCAAAGG

GCGACCAAA

>mangabey

ATGAGCTTTTTCCAACTCCTGATGAAAAGGAAGGAACTCCTTCCCTTGGTGCTGTTCGTGAGTGTGGCGGCGGGTGGAGC

CTCGTCTTTTGCTGTGTATTCTCTTTGGAAAACCGATGTGATCCTTGATCGAAAAAGAAATCCAGAACCTTGGGAAACTG

TGGACCCTACTATACCGCAAAAGCTTATAACAATCAACCAACAATGGAAGCCCATTGAAGAGCTGCAAAAGGTCCAAAGG

GCGACCAAA

>colobus

ATGAGCTTTTTCCAACTCCTGATGAAAAGGAAGGAACTCATTCCCTTGGTGCTGTTCACGAGTGTGGCGGCGGGTGGAGC

CTCGTCTTTTGCTGTGTATTCTTTTTGGAAAACCGATGTGATCCTTGATCGGAAAAGAAATCCAGAACCTTGGGAAACTG

TGGACCCTACTACACCGCAAAAGCTTATAACAATCAACCAACAATGGAAGCCCATTGAAGAGTTGCAAAAGGTCCAAAGG

GCGACCAAA

>colobusR

ATGACCTTTTTCCAACTCCTGATGAAAAGGAAGGAACTCATTCCCTTGGTGCTGTTCACGAGTGTGGCGGCGGGTGGAGC

CTCGTCTTTTGCTGTGTATTCTTTTTGGAAAACCGATGTGATCCTTGATCGGAAAAGAAATCCAGAACCTTGGGAAACTG

TGGACCCTACTATACCGCAAAAGCTTATAACAATCAACCAACAATGGAAGCCCATTGAAGAGTTGCAAAAGGTCCAAAGG

GCGACCAAA

>proboscis

ATGAGCTTTTTCCAACTCCTGATGAAAAGGAAGGAACTCATTCCCTTGGTGCTGTTCACGAGTGTGGCGGCGGGTGGAGC

CTCGTCTTTTGCTGTGTATTCTCTTTGGAAAACCGATGTGATCCTTGATCGAAAAAGAAATCCAGAACCTTGGGAAACTG

TGGACCCTACTATACCGCAAAAGCTTATAACAATCAACCAACAATGGAAGCCCATTGAAGAGTTGCAAAAGGTCCAAAGG

GTGACCAAA

>snubR

ATGAGCTTTTTCCAACTCCTGATGAAAAGGAAGGAACTCATTCCCTTGGTGCTGTTCACGAGTGTGGCGGCGGGTGGAGC

CTCGTCTTTTGCTGTGTATTCTCTTTGGAAAACCGATGTGATCCTTGATCGAAAAAGAAATCCAGAACCTTGGGAAACTG

TGGACCCTACTATACCGCAAAAGCTTATAACAATCAACCAACAATGGAAGCCCATTGAAGAGTTGCAAAAGGTCCAAAGG

GTGACCAAA

>snubB

ATGAGCTTTTTCCAACTCCTGATGAAAAGGAAGGAACTCATTCCCTTGGTGCTGTTCACGAGTGTGGCGGCGGGTGGAGC

CTCGTCTTTTGCTGTGTATTCTCTTTGGAAAACCGATGTGATCCTTGATCGAAAAAGAAATCCAGAACCTTGGGAAACTG

TGGACCCTACTATACCGCAAAAGCTTATAACAATCAACCAACAATGGAAGCCCATTGAAGAGTTGCAAAAGGTCCAAAGG

GTGACCAAA

>marmoset

ATGGGCTTTTTCCAATTCCTGAGGAAAAACAAGGAACTCATTCCCTTGGTGGTGTTCATGAGTCTGGCGGCCAGTGGAGC

CTCATCTTTCGCTGTGTATTCTCTTCGGAAAACCGATGTGATCATTGATCGAAAAGGAAATCCAGAACCTTGGGAAACTG

TGGACCCTACTGTACCTCAAAAGCTTATAACAATCAACCAAGAATGGAAGCCCATTGAAGAGTTGCAGAAGGTCCAAAGG

GCAACCAAA

>owl

ATGGGCTTTTTCCAATTCCTGAAGAAAAACAAGGAACTCGTTCCCTTGGTGGTGTTCATGACTCTGGCGGCCAGTGGAGC

CTCATCTTTCGCTGTGTATTCTCTTCGGAAAACCGATGTGATCATTGATCGAAAAGGAAATCCAGAACCTTGGGAAACTG

TGGACCCTACTGTACCTCAAAAGCTTGTAACAATCAACCAAGAATGGAAGCCCATTGAAGAGTTGCAGAAGGTCCGAAGC

GCGACCAAA

>capuchin

ATGGGCTTTTTCCAATTGCTGAAGAAAAATAAGGAACTCATTCCCTTGGTGGTGTTCATGGCTGTGGCGGCCAGTGGAGC

CTCATCTTTCGCTGTGTATTCTCTTCGGAAAACCGATGTGATCATTGATCGAAAAGGAAATCCAGAACCTTGGGAAACTG

TGGACCCTACCGTACCTCAAAAGCTTATAACAATCAACCAAGAATGGAAGCCTGTTGAAGAGTTGCAGAAGGTCCGAAAG

GCGACCAAA

>SQM

ATGAGCTTTTTCCAATTGCTGAAGAAAAACAAGGAGCTCATTCCCTTGGTGGTGTTCATGAGTGTGGCGGCTGGTGGAGC

CTCATCTTTCGCTGTGTATTCTCTTCGGAAAACCGATGTGATCATTGATCGAAAAAGAAATCCAGAACCTTGGGAAAATG

TGGACCCTACTGTACCTCAAAAGCTTTTAACAATCAACCAAGAATGGAAGCCTATTGAAGAGTTGGAGAAGGTCCGAAAG

GCGACCAAA

**6) Primate *MISTRAV*/*C15ORF48* input sequences lacking predicted TMEM for evolutionary analysis, related to Figure 2.**

>human

ATGAGCTTTTTCCAACTCCTGATGAAAAGGAAGGAATGGAAAACCGATGTGATCCTTGATCGAAAAAAAAATCCAGAACC

TTGGGAAACTGTGGACCCTACTGTACCTCAAAAGCTTATAACAATCAACCAACAATGGAAACCCATTGAAGAGTTGCAAA

ATGTCCAAAGGGTGACCAAA

>chimp

ATGAGCTTTTTCCAACTCCTGATGAAAAGGAAGGAATGGAAAACCGATGTGATCCTTGATCGAAAAAAAAATCCAGAACC

TTGGGAAACTGTGGACCCTACTGTACCTCAAAAGCTTATAACAATCAACCAACAATGGAAACCCATTGAAGAGTTGCAAA

ATGTCCAAAGGGTGACCAAA

>bonobo

ATGAGCTTTTTCCAACTCCTGATGAAAAGGAAGGAATGGAAAACCGATGTGATCCTTGATCGAAAAAAAAATCCAGAACC

TTGGGAAACTGTGGACCCTACTGTACCTCAAAAGCTTATAACAATCAACCAACAATGGAAACCCATTGAAGAGTTGCAAA

ATGTCCAAAGGGTGACCAAA

>gorilla

ATGAGCTTTTTCCAACTCCTGATGAAAAGGAAGGAATGGAAAACCGATGTGATCCTTGATCGAAAAAGAAATCCAGAACC

TTGGGAAACTGTGGACCCTACTGTACCTCAAAAGCTTATAACAATCAACCAACAATGGAAACCCATTGAAGAGTTGCAAA

ATGTCCAAAGGGCGACCAAA

>orang

ATGAGCTTTTTCCAACTCCTGATGAAAAGGAAGGAATGGAAAACCGATGTGATCCTTGATCGAAAAAAAAATCCAGAACC

TTGGGAAACTGTGGACCCTACTGTACCTCAAAAGCTTATAACAATCAACCAACAGTGGAAACCCATTGAAGAGTTGCAAA

AGGTCCAAAGGGTGACCAAA

>gibbon

ATGAGCTTTTTCCAACTCCTGATGAAAAGGAAGGAATGGAAAACCGATGTGATCCTTGATCGAAAAAGAAATCCAGAACC

TTGGGAAACTGTGGACCCTACTATACCTCAAAAGCTTATAACAATCAACCAACGGTGGAAACCCATTGAAGAGTTGCAAA

AGGTCCAAAGAGTGACCATA

>AGM

ATGAGCTTTTTCCAACTCCTGATGAAAAGGAAGGAATGGAAAACCGATGTGATCCTTGATCGAAAAAGAAATCCAGAACC

TTGGGAAACCGTGGATCCTACTATACCGCAAAAGCTTATAACAATCAACCAACAATGGAAGCCCATTGAAGACCTGCAAA

AGGTCCGAAGGGCGACCAAA

>crabMac

ATGAGCTTTTTCCAACTCCTGATGAAAAGGAAGGAATGGAAAACCGATGTGATCCTTGATCGAAAAAGAAATCCAGAACC

TTGGGAAACTGTGGACCCTACTATACCGCAAAAGCTTATAACAATCAACCAACAATGGAAGCCCATTGAAGAGCTGCAAA

AGGTCCAAAGGGCGACCAAA

>rhesus

ATGAGCTTTTTCCAACTCCTGATGAAAAGGAAGGAATGGAAAACCGATGTGATCCTTGATCGAAAAAGAAATCCAGAACC

TTGGGAAACTGTGGACCCTACTATACCGCAAAAGCTTATAACAATCAACCAACAATGGAAGCCCATTGAAGAGCTGCAAA

AGGTCCGAAGGGCGACCAAA

>pigMac

ATGAGCTTTTTCCAACTCCTGATGAAAAGGAAGGAATGGAAAACCGATGTGATCCTTGATCGAAAAAGAAATCCAGAACC

TTGGGAAACTGTGGACCCTACTATACCGCAAAAGCTTATAACAATCAACCAACAATGGAAGCCCATTGAAGAGCTGCAAA

AGGTCCGAAGGGCGACCAAA

>baboonH

ATGAGCTTTTTCCAACTCCTGATGAAAAGGAAGGAACGGAAAACCGATGTGATCCTTGATCGAAAAAGAAATCCAGAACC

TTGGGAAACTGTGGACCCTACTATACCGCAAAAGCTTATAACAATCAACCAACAATGGAAGCCCATTGAAGAGCTGCAAA

AGGTCCGAAGGGCGACCAAA

>baboonA

ATGAGCTTTTTCCAACTCCTGATGAAAAGGAAGGAACGGAAAACCGATGTGATCCTTGATCGAAAAAGAAATCCAGAACC

TTGGGAAACTGTGGACCCTACTATACCGCAAAAGCTTATAACAATCAACCAACAATGGAAGCCCATTGAAGAGCTGCAAA

AGGTCCGAAGGGCGACCAAA

>drill

ATGAGCTTTTTCCAACTCCTGATGAAAAGGAAGGAATGGAAAACCGATGTGATCCTTGATCGAAAAAGAAATCCAGAACC

TTGGGAAACTGTGGACCCTACTATACCGCAAAAGCTTATAACAATCAACCAACAATGGAAGCCCATTGAGGAGCTGCAAA

AGGTCCAAAGGGCGACCAAA

>mangabey

ATGAGCTTTTTCCAACTCCTGATGAAAAGGAAGGAATGGAAAACCGATGTGATCCTTGATCGAAAAAGAAATCCAGAACC

TTGGGAAACTGTGGACCCTACTATACCGCAAAAGCTTATAACAATCAACCAACAATGGAAGCCCATTGAAGAGCTGCAAA

AGGTCCAAAGGGCGACCAAA

>colobus

ATGAGCTTTTTCCAACTCCTGATGAAAAGGAAGGAATGGAAAACCGATGTGATCCTTGATCGGAAAAGAAATCCAGAACC

TTGGGAAACTGTGGACCCTACTACACCGCAAAAGCTTATAACAATCAACCAACAATGGAAGCCCATTGAAGAGTTGCAAA

AGGTCCAAAGGGCGACCAAA

>colobusR

ATGACCTTTTTCCAACTCCTGATGAAAAGGAAGGAATGGAAAACCGATGTGATCCTTGATCGGAAAAGAAATCCAGAACC

TTGGGAAACTGTGGACCCTACTATACCGCAAAAGCTTATAACAATCAACCAACAATGGAAGCCCATTGAAGAGTTGCAAA

AGGTCCAAAGGGCGACCAAA

>proboscis

ATGAGCTTTTTCCAACTCCTGATGAAAAGGAAGGAATGGAAAACCGATGTGATCCTTGATCGAAAAAGAAATCCAGAACC

TTGGGAAACTGTGGACCCTACTATACCGCAAAAGCTTATAACAATCAACCAACAATGGAAGCCCATTGAAGAGTTGCAAA

AGGTCCAAAGGGTGACCAAA

>snubR

ATGAGCTTTTTCCAACTCCTGATGAAAAGGAAGGAATGGAAAACCGATGTGATCCTTGATCGAAAAAGAAATCCAGAACC

TTGGGAAACTGTGGACCCTACTATACCGCAAAAGCTTATAACAATCAACCAACAATGGAAGCCCATTGAAGAGTTGCAAA

AGGTCCAAAGGGTGACCAAA

>snubB

ATGAGCTTTTTCCAACTCCTGATGAAAAGGAAGGAATGGAAAACCGATGTGATCCTTGATCGAAAAAGAAATCCAGAACC

TTGGGAAACTGTGGACCCTACTATACCGCAAAAGCTTATAACAATCAACCAACAATGGAAGCCCATTGAAGAGTTGCAAA

AGGTCCAAAGGGTGACCAAA

>marmoset

ATGGGCTTTTTCCAATTCCTGAGGAAAAACAAGGAACGGAAAACCGATGTGATCATTGATCGAAAAGGAAATCCAGAACC

TTGGGAAACTGTGGACCCTACTGTACCTCAAAAGCTTATAACAATCAACCAAGAATGGAAGCCCATTGAAGAGTTGCAGA

AGGTCCAAAGGGCAACCAAA

>owl

ATGGGCTTTTTCCAATTCCTGAAGAAAAACAAGGAACGGAAAACCGATGTGATCATTGATCGAAAAGGAAATCCAGAACC

TTGGGAAACTGTGGACCCTACTGTACCTCAAAAGCTTGTAACAATCAACCAAGAATGGAAGCCCATTGAAGAGTTGCAGA

AGGTCCGAAGCGCGACCAAA

>capuchin

ATGGGCTTTTTCCAATTGCTGAAGAAAAATAAGGAACGGAAAACCGATGTGATCATTGATCGAAAAGGAAATCCAGAACC

TTGGGAAACTGTGGACCCTACCGTACCTCAAAAGCTTATAACAATCAACCAAGAATGGAAGCCTGTTGAAGAGTTGCAGA

AGGTCCGAAAGGCGACCAAA

>SQM

ATGAGCTTTTTCCAATTGCTGAAGAAAAACAAGGAGCGGAAAACCGATGTGATCATTGATCGAAAAAGAAATCCAGAACC

TTGGGAAAATGTGGACCCTACTGTACCTCAAAAGCTTTTAACAATCAACCAAGAATGGAAGCCTATTGAAGAGTTGGAGA

AGGTCCGAAAGGCGACCAAA

**7) Primate *MISTR1*/*NDUFA4* input sequences for evolutionary analysis, related to Figure 2.**

>human

ATGCTCCGCCAGATCATCGGTCAGGCCAAGAAGCATCCGAGCTTGATCCCCCTCTTTGTATTTATTGGAACTGGAGCTAC

TGGAGCAACACTGTATCTCTTGCGTCTGGCATTGTTCAATCCAGATGTTTGTTGGGACAGAAATAACCCAGAGCCCTGGA

ACAAACTGGGTCCCAATGATCAATACAAGTTCTACTCAGTGAATGTGGATTACAGCAAGCTGAAGAAGGAACGTCCAGAT

TTC

>chimp

ATGCTCCGCCAGATCATCGGTCAGGCCAAGAAGCATCCGAGCTTGATCCCCCTCTTTGTATTTATTGGAACTGGAGCTAC

TGGAGCAACACTGTATCTCTTGCGTCTGGCATTGTTCAATCCAGATGTTTGTTGGGACAGAAATAACCCAGAGCCCTGGA

ACAAACTGGGTCCCAATGATCAATACAAGTTCTACTCAGTGAATGTGGATTACAGCAAGCTGAAGAAGGAACGTCCAGAT

TTC

>bonobo

ATGCTCCGCCAGATCATCGGTCAGGCCAAGAAGCATCCGAGCTTGATCCCCCTCTTTGTATTTATTGGAACTGGAGCTAC

TGGAGCGACACTGTATCTCTTGCGTCTGGCATTGTTCAATCCAGATGTTTGTTGGGACAGAAATAACCCAGAGCCCTGGA

ACAAACTGGGTCCCAATGATCAATACAAGTTCTACTCAGTGAATGTGGATTACAGCAAGCTGAAGAAGGAACGTCCAGAT

TTC

>gorilla

ATGCTCCGCCAGATCATCGGTCAGGCCAAGAAGCATCCGAGCTTGATCCCCCTCTTTGTATTTATTGGAACTGGAGCTAC

TGGAGCAACACTGTATCTCTTGCGTCTGGCATTGTTCAATCCAGATGTTTGTTGGGACAAAAGTAAACCAGAGCCCTGGA

ACAAACTGGGTCCCAATGATCAATACAAGTTCTACTCAGTGAATGTGGACTACAGCAAGCTGAAGAAGGAACGTCCAGAT

TTC

>orang

ATGCTCCGCCAGATCCTCAGTCAGGCCAAGAAGCATCCGAGCTTGATCCCCCTCTTTGTATTTATTGGAACTGGAGCTTC

TGGAGCAACACTGTATCTCTTGCGTCTGGCATTGTTCAATCCAGATGTTTGTTGGGACAGAAATAACCCAGAGCCCTGGA

ACAAACTGGGTCCCAATGATCAATACAAGTTCTACTCAGTGAATGTGGATTACAGCAAGCTGAAGAAGGAACGTCCAGAT

TTC

>gibbon

ATGCTTCGCCAGATCATCAGTCAGGCCAAGAAGCATCCGAGCTTGATCCCCCTCTTTGTATTTATTGGAACTGGAGCTTC

TGGAGCAACACTGTATCTCTTGCGTCTGGCATTGTTCAATCCAGATGTTTGTTGGGACAGAAATAACCCAGAGCCCTGGA

ACAAACTGGGTCCCAATGATCAATACAAGTTCTACTCAGTGAATGTGGATTACAGCAAACTGAAGAAGGAACGTCCAGAT

TTC

>rhesus

ATGCTCCGCCACATCCTCGGTCTGGCCAAGAAGCATCCGAGCTTGATCCCCCTCTTTGTATTTCTTGGAACTGGAGCTAC

TGGAGCAACACTGTATCTCTTGCGTCTGGCCTTGTTCAGTCCAGATGTTTGTTGGGACAGAAATAACCCAGAGCCCTGGA

ACAAACTGGGTCCCAATGATCAATACAAGTTCTACTCAGTGAATGTGGATTACGACAAACTGAAGAAGGAACGTCCAGAT

TTC

>crabMac

ATGCTCCGCCACATCCTCGGTCTGGCCAAGAAGCATCCGAGCTTGATCCCCCTCTTTGTATTTCTTGGAACTGGAGCTAC

TGGAGCAACACTGTATCTCTTGCGTCTGGCCTTGTTCAGTCCAGATGTTTGTTGGGACAGAAATAACCCAGAGCCCTGGA

ACAAACTGGGTCCCAATGATCAATACAAGTTCTACTCAGTGAATGTGGATTACGACAAACTGAAGAAGGAACGTCCAGAT

TTC

>pigMac

ATGCTCCGCCACATCCTCGGTCTGGCCAAGAAGCATCCGAGCTTGATCCCCCTCTTTGTATTTCTTGGAACTGGAGCTAC

TGGAGCAACACTGTATCTCTTGCGTCTGGCCTTGTTCAGTCCAGATGTTTGTTGGGACAGAAATAACCCAGAGCCCTGGA

ACAAACTGGGTCCCAATGATCAATACAAGTTCTACTCAGTGAATGTGGATTACGACAAACTGAAGAAGGAACGTCCAGAT

TTC

>baboonA

ATGCTCCGCCAGATCCTCAGTCTGTCCAAGAAGCATCCGAGCTTGATGCCCCTCTTTGTATTTATTGGAACTGGAGCTAC

TGGAGCAACACTGTATCTCTTGCGTCTGGCCTTGTTCAGTCCAGATGTTTGTTGGGACAGAAATAACCCAGAGCCCTGGA

ACAAACTGGGTCCCAATGATCAATACAAGTTCTACTCAGTGAATGTGGATTACAACAAACTGAAGAAGGAACGTCCAGAT

TTC

>baboonH

ATGCTCCGCCAGATCCTCAGTCTGTCCAAGAAGCATCCGAGCTTGATGCCCCTCTTTGTATTTATTGGAACTGGAGCTAC

TGGAGCAACACTGTATCTCTTGCGTCTGGCCTTGTTCAGTCCAGATGTTTGTTGGGACAGAAATAACCCAGAGCCCTGGA

ACAAACTGGGTCCCAATGATCAATACAAGTTCTACTCAGTGAATGTGGATTACAACAAACTGAAGAAGGAACGTCCAGAT

TTC

>drill

ATGCTCCGCCAGATCCTCGGTTTGGCCAAGAAGCATCCGAGCTTGATCCCCCTCTTTGTATTTCTTGGAACTGGAGCTAC

TGGAGCAACACTGTATCTCTTGCGTCTGGCCTTGTTCAGTCCAGATGTTTGTTGGGACAGAAATAACCCAGAGCCCTGGA

ACAAACTGGGTCCCAATGATCAATACAAGTTCTACTCAGTGAATGTGGATTACAACAAACTGAAGAAGGAACGTCCAGAT

TTC

>mangabey

ATGCTCCGCCAGATCTTCGGTTTGGCCAAGAAGCATCCGAGCTTGATCCCCCTCTTTGTATTTCTTGGAACTGGAGCTAC

TGGAGCAACACTGTATCTCTTGCGTCTGGCCTTGTTCAGTCCAGATGTTTGTTGGGACAGAAATAACCCAGAGCCCTGGA

ACAAACTGGGTCCCAATGATCAATACAAGTTCTACTCAGTGAATGTGGATTACAACAAACTGAAGAAGGAACGTCCAGAT

TTC

>AGM

ATGCTCCGCCATATGGTCGGTCTGGCCAAGAAGCATCCGAGCTTGATCCCCCTCTTTGTATTTCTTGGAACTGGAGCTAC

TGGAGCAACACTGTATCTCTTGCGTCTGGCCTTGTTCAGTCCAGATGTTTGTTGGGACAGAAATAACCCAGAGCCCTGGA

ACAAACTGGGTCCCAATGATCGATACAAGTTCTACTCAGTGAATGTGGATTACGACAAACTGAAGAAGGAACGTCCAGAT

TTC

>colobusR

ATGTTCCGCCAGATCCTCGGTCAGGCCAAGAAGCATCCGAGCTTGATCCCCCTCTTTGTATTTCTTGGAACCGGAGCTGC

TGGAGCAACACTGTATCTCTTGCGTCTGGCATTGTTCAATCCAGATGTTTGTTGGGACAGAAATAACCCAGAGCCCTGGA

ACAAACTGGGTCCCAATGATCAATACAAGTTCTACTCAGTGAATGTGGATTACAGCAAACTGAAGAAGGAACGTCCAGAT

TTC

>colobus

ATGCTCCGCCAGATCCTCGGTCAGGCCAAGAAGCATCCGAGCTTGATCCCCCTCTTTGTATTTCTTGGAACTGGAGCTAC

TGGAGCAACACTGTATCTCTTGCGTCTGGCATTGTTCAATCCAGATGTTTGTTGGGACAGAAATAACCCAGAGCCCTGGA

ACAAACTGGGTCCCAATGATCAATACAAGTTCTACTCAGTGAATGTGGATTACAGCAAACTGAAGAAGGAACGTCCAGAT

TTC

>snubR

ATGCTCCGCCAGATCCTCGGTCAGGCCAAGAAGCATCCGAGCTTGATCCCCCTCTTTGTATTTCTTGGAACTGGAGCTAC

TGGAGCAACACTGTATCTCTTGCGTCTGGCATTGTTCAATCCAGATGTTTGTTGGGACAGAAATAACCCAGAGCCCTGGA

ACAAACTGGGTCCCAATGATCAATACAAGTTCTACTCAGTGAATGTGGATTACAGCAAACTGAAGAAGGAACGTCCAGAT

TTC

>snubB

ATGCTCCGCCAGATCCTCGGTCAGGCCAAGAAGCATCCGAGCTTGATCCCCCTCTTTGTATTTCTTGGAACTGGAGCTAC

TGGAGCGACACTGTATCTCTTGCGTCTGGCATTGTTCAATCCAGATGTTTGTTGGGACAGAAATAACCCAGAGCCCTGGA

ACAAACTGGGTCCCAATGATCAATACAAGTTCTACTCAGTGAATGTGGATTACAGCAAACTGAAGAAGGAACGTCCAGAT

TTC

>proboscis

ATGCTCCGCCAGATCCTCGGTCAGGCCAAGAAGCATCCGAGCTTGATCCCCCTCTTTGTATTTCTTGGAACTGGAGCTAC

TGGAGCAACACTGTATCTCTTGCGCCTGGCATTGTTCAATCCAGATGTTTGTTGGGACAGAAATAACCCAGAGCCCTGGA

ACAAACTGGGTCCCAATGATCAATACAAGTTCTACTCAGTGAATGTGGATTACAGCAAACTGAAGAAGGAACGTCCAGAT

TTC

>marmoset

ATGCTCCGCCACATCTTAGGTCAGGCCAAGAAGCATCCGAGCTTGATCCCCCTGTTTGTATTTATTGGAGCTGGAGGTGG

TGGAGCAGCCCTGTATCTCTTGCGTTTGGCATTGTTCAATCCAGATGTTTGTTGGGACAAAAATAACCCAGAGCCCTGGA

ACAAACTGGGTCCCAATGATCAATACAAGTTCTACTCAGTGAATGTGGATTACAGCAAACTGAAGAAAGAACGTCCAGAT

TTC

>owl

ATGCTCCGCCACATCTTAGGTCAGGCCAAGAAGCATCCGAGCTTGATCCCCCTCTTTGTATTTATTGGAGCTGGAGGTGG

TGGAGCAGCTCTGTATCTCTTGCGTTTGGCATTGTTCAATCCAGATGTTTGTTGGGACAAAAATAACCCAGAGCCCTGGA

ACAAACTGGGTCCTAATGATCAATACAAGTTCTACTCAGTGAATGTGGACTACAGCAAACTGAAGAAAGAACGTCCAGAT

TTC

>capuchin

ATGCTCCGCCACATCTTAGGTCAGGCCAAGAAGCATCCGAGCTTGATCCCGCTCTTTGTATTTATTGGAGCTGGAGGTGG

GGGAGCAGCCCTGTATCTCTTGCGTTTGGCATTGTTCAATCCAGATGTTTGTTGGGACAGAAATAACCCAGAGCCCTGGA

ACAAACTAGGTCCCAATGATCAATATAAGTTCTACTCTGTGAATGTGGATTATAGCAAACTGAAGAAAGAACGTCCAGAT

TTC

>SQM

ATGCTCCGCCACATCTTAGGTCAGGCCAAGAAGCATCCGAGCTTGATCCCCCTCTTTGTATTTATTGGAGCTGGAGGTGG

TGGAGCAACCCTGTATCTCTTGCGTTTGGCATTGTTCAGTCCAGATGTTTGTTGGGACAAAAATAACCCAGAGCCCTGGA

ACAAAATGGGTCCCAATGATCAGTACAAGTTCTACTCTGTGAATGTGGATTACAGCAAACTGAAGAAAGAACGTCCAGAT

TTC

**8) Primate *MISTR1*/*NDUFA4* input sequences lacking predicted TMEM for evolutionary analysis,**

**related to Figure 2.**

>human

ATGCTCCGCCAGATCATCGGTCAGGCCAAGAAGCATCCGAGCTTGTTCAATCCAGATGTTTGTTGGGACAGAAATAACCC

AGAGCCCTGGAACAAACTGGGTCCCAATGATCAATACAAGTTCTACTCAGTGAATGTGGATTACAGCAAGCTGAAGAAGG

AACGTCCAGATTTC

>chimp

ATGCTCCGCCAGATCATCGGTCAGGCCAAGAAGCATCCGAGCTTGTTCAATCCAGATGTTTGTTGGGACAGAAATAACCC

AGAGCCCTGGAACAAACTGGGTCCCAATGATCAATACAAGTTCTACTCAGTGAATGTGGATTACAGCAAGCTGAAGAAGG

AACGTCCAGATTTC

>bonobo

ATGCTCCGCCAGATCATCGGTCAGGCCAAGAAGCATCCGAGCTTGTTCAATCCAGATGTTTGTTGGGACAGAAATAACCC

AGAGCCCTGGAACAAACTGGGTCCCAATGATCAATACAAGTTCTACTCAGTGAATGTGGATTACAGCAAGCTGAAGAAGG

AACGTCCAGATTTC

>gorilla

ATGCTCCGCCAGATCATCGGTCAGGCCAAGAAGCATCCGAGCTTGTTCAATCCAGATGTTTGTTGGGACAAAAGTAAACC

AGAGCCCTGGAACAAACTGGGTCCCAATGATCAATACAAGTTCTACTCAGTGAATGTGGACTACAGCAAGCTGAAGAAGG

AACGTCCAGATTTC

>orang

ATGCTCCGCCAGATCCTCAGTCAGGCCAAGAAGCATCCGAGCTTGTTCAATCCAGATGTTTGTTGGGACAGAAATAACCC

AGAGCCCTGGAACAAACTGGGTCCCAATGATCAATACAAGTTCTACTCAGTGAATGTGGATTACAGCAAGCTGAAGAAGG

AACGTCCAGATTTC

>gibbon

ATGCTTCGCCAGATCATCAGTCAGGCCAAGAAGCATCCGAGCTTGTTCAATCCAGATGTTTGTTGGGACAGAAATAACCC

AGAGCCCTGGAACAAACTGGGTCCCAATGATCAATACAAGTTCTACTCAGTGAATGTGGATTACAGCAAACTGAAGAAGG

AACGTCCAGATTTC

>rhesus

ATGCTCCGCCACATCCTCGGTCTGGCCAAGAAGCATCCGAGCTTGTTCAGTCCAGATGTTTGTTGGGACAGAAATAACCC

AGAGCCCTGGAACAAACTGGGTCCCAATGATCAATACAAGTTCTACTCAGTGAATGTGGATTACGACAAACTGAAGAAGG

AACGTCCAGATTTC

>crabMac

ATGCTCCGCCACATCCTCGGTCTGGCCAAGAAGCATCCGAGCTTGTTCAGTCCAGATGTTTGTTGGGACAGAAATAACCC

AGAGCCCTGGAACAAACTGGGTCCCAATGATCAATACAAGTTCTACTCAGTGAATGTGGATTACGACAAACTGAAGAAGG

AACGTCCAGATTTC

>pigMac

ATGCTCCGCCACATCCTCGGTCTGGCCAAGAAGCATCCGAGCTTGTTCAGTCCAGATGTTTGTTGGGACAGAAATAACCC

AGAGCCCTGGAACAAACTGGGTCCCAATGATCAATACAAGTTCTACTCAGTGAATGTGGATTACGACAAACTGAAGAAGG

AACGTCCAGATTTC

>baboonA

ATGCTCCGCCAGATCCTCAGTCTGTCCAAGAAGCATCCGAGCTTGTTCAGTCCAGATGTTTGTTGGGACAGAAATAACCC

AGAGCCCTGGAACAAACTGGGTCCCAATGATCAATACAAGTTCTACTCAGTGAATGTGGATTACAACAAACTGAAGAAGG

AACGTCCAGATTTC

>baboonH

ATGCTCCGCCAGATCCTCAGTCTGTCCAAGAAGCATCCGAGCTTGTTCAGTCCAGATGTTTGTTGGGACAGAAATAACCC

AGAGCCCTGGAACAAACTGGGTCCCAATGATCAATACAAGTTCTACTCAGTGAATGTGGATTACAACAAACTGAAGAAGG

AACGTCCAGATTTC

>drill

ATGCTCCGCCAGATCCTCGGTTTGGCCAAGAAGCATCCGAGCTTGTTCAGTCCAGATGTTTGTTGGGACAGAAATAACCC

AGAGCCCTGGAACAAACTGGGTCCCAATGATCAATACAAGTTCTACTCAGTGAATGTGGATTACAACAAACTGAAGAAGG

AACGTCCAGATTTC

>mangabey

ATGCTCCGCCAGATCTTCGGTTTGGCCAAGAAGCATCCGAGCTTGTTCAGTCCAGATGTTTGTTGGGACAGAAATAACCC

AGAGCCCTGGAACAAACTGGGTCCCAATGATCAATACAAGTTCTACTCAGTGAATGTGGATTACAACAAACTGAAGAAGG

AACGTCCAGATTTC

>AGM

ATGCTCCGCCATATGGTCGGTCTGGCCAAGAAGCATCCGAGCTTGTTCAGTCCAGATGTTTGTTGGGACAGAAATAACCC

AGAGCCCTGGAACAAACTGGGTCCCAATGATCGATACAAGTTCTACTCAGTGAATGTGGATTACGACAAACTGAAGAAGG

AACGTCCAGATTTC

>colobusR

ATGTTCCGCCAGATCCTCGGTCAGGCCAAGAAGCATCCGAGCTTGTTCAATCCAGATGTTTGTTGGGACAGAAATAACCC

AGAGCCCTGGAACAAACTGGGTCCCAATGATCAATACAAGTTCTACTCAGTGAATGTGGATTACAGCAAACTGAAGAAGG

AACGTCCAGATTTC

>colobus

ATGCTCCGCCAGATCCTCGGTCAGGCCAAGAAGCATCCGAGCTTGTTCAATCCAGATGTTTGTTGGGACAGAAATAACCC

AGAGCCCTGGAACAAACTGGGTCCCAATGATCAATACAAGTTCTACTCAGTGAATGTGGATTACAGCAAACTGAAGAAGG

AACGTCCAGATTTC

>snubR

ATGCTCCGCCAGATCCTCGGTCAGGCCAAGAAGCATCCGAGCTTGTTCAATCCAGATGTTTGTTGGGACAGAAATAACCC

AGAGCCCTGGAACAAACTGGGTCCCAATGATCAATACAAGTTCTACTCAGTGAATGTGGATTACAGCAAACTGAAGAAGG

AACGTCCAGATTTC

>snubB

ATGCTCCGCCAGATCCTCGGTCAGGCCAAGAAGCATCCGAGCTTGTTCAATCCAGATGTTTGTTGGGACAGAAATAACCC

AGAGCCCTGGAACAAACTGGGTCCCAATGATCAATACAAGTTCTACTCAGTGAATGTGGATTACAGCAAACTGAAGAAGG

AACGTCCAGATTTC

>proboscis

ATGCTCCGCCAGATCCTCGGTCAGGCCAAGAAGCATCCGAGCTTGTTCAATCCAGATGTTTGTTGGGACAGAAATAACCC

AGAGCCCTGGAACAAACTGGGTCCCAATGATCAATACAAGTTCTACTCAGTGAATGTGGATTACAGCAAACTGAAGAAGG

AACGTCCAGATTTC

>marmoset

ATGCTCCGCCACATCTTAGGTCAGGCCAAGAAGCATCCGAGCTTGTTCAATCCAGATGTTTGTTGGGACAAAAATAACCC

AGAGCCCTGGAACAAACTGGGTCCCAATGATCAATACAAGTTCTACTCAGTGAATGTGGATTACAGCAAACTGAAGAAAG

AACGTCCAGATTTC

>owl

ATGCTCCGCCACATCTTAGGTCAGGCCAAGAAGCATCCGAGCTTGTTCAATCCAGATGTTTGTTGGGACAAAAATAACCC

AGAGCCCTGGAACAAACTGGGTCCTAATGATCAATACAAGTTCTACTCAGTGAATGTGGACTACAGCAAACTGAAGAAAG

AACGTCCAGATTTC

>capuchin

ATGCTCCGCCACATCTTAGGTCAGGCCAAGAAGCATCCGAGCTTGTTCAATCCAGATGTTTGTTGGGACAGAAATAACCC

AGAGCCCTGGAACAAACTAGGTCCCAATGATCAATATAAGTTCTACTCTGTGAATGTGGATTATAGCAAACTGAAGAAAG

AACGTCCAGATTTC

>SQM

ATGCTCCGCCACATCTTAGGTCAGGCCAAGAAGCATCCGAGCTTGTTCAGTCCAGATGTTTGTTGGGACAAAAATAACCC

AGAGCCCTGGAACAAAATGGGTCCCAATGATCAGTACAAGTTCTACTCTGTGAATGTGGATTACAGCAAACTGAAGAAAG

AACGTCCAGATTTC

**9) Primate *MISTRH*/*NDUFA4L2* input sequences for evolutionary analysis, related to Figure 2.**

>human

ATGGCAGGAGCCAGTCTTGGGGCCCGCTTCTACCGGCAGATCAAAAGACATCCGGGGATCATCCCGATGATCGGCTTAAT

CTGCCTGGGCATGGGCAGCGCTGCGCTTTACTTGCTGCGACTCGCCCTTCGCAGCCCCGACGTCTGCTGGGACAGAAAGA

ACAACCCGGAGCCCTGGAACCGCCTGAGCCCCAATGACCAATACAAGTTCCTTGCAGTTTCCACTGACTATAAGAAGCTG

AAGAAGGACCGGCCAGACTTC

>chimp

ATGGCAGGAGCCAGTCTTGGGGCCCGCTTCTACCGGCAGATCAAAAGACATCCGGGGATCATCCCGATGATCGGCTTAAT

CTGCCTGGGCATGGGCAGCGCTGCGCTCTACTTGCTGCGACTCGCCCTTCGCAGCCCCGACGTCTGCTGGGACAGAAAGA

ACAACCCGGAGCCCTGGAACCGCCTGAGCCCCAATGACCAATACAAGTTCCTTGCAGTTTCCACTGACTATAAGAAGCTG

AAGAAGGACCGGCCAGACTTC

>bonobo

ATGGCAGGAGCCAGTCTTGGGGCCCGCTTCTACCGGCAGATCAAAAGACATCCGGGGATCATCCCGATGATCGGCTTAAT

CTGCCTGGGCATGGGCAGCGCTGCGCTCTACTTGCTGCGACTCGCCCTTCGCAGCCCCGACGTCTGCTGGGACAGAAAGA

ACAACCCGGAGCCCTGGAACCGCCTGAGCCCCAATGACCAATACAAGTTCCTTGCAGTTTCCACTGACTATAAGAAGCTG

AAGAAGGACCGGCCAGACTTC

>gorilla

ATGGCAGGAGCCAGTCTTGGGGCCCGCTTCTACCGGCAGATCAAAAGACATCCGGGGATCATCCCGATGATCGGCTTAAT

CTGCCTGGGCATGGGCAGCGCTGCGCTCTACTTGCTGCGACTCGCCCTTCGCAGCCCCGACGTCTGCTGGGACAGAAAGA

ACAACCCGGAGCCCTGGAACCGCCTGAGCCCCAATGACCAATACAAGTTCCTTGCAGTTTCCACTGACTATAAGAAGCTG

AAGAAGGACCGGCCAGACTTC

>orang

ATGGCAGGAGCCAGTCTTGGGGCCCGCTTCTACCGGCAGATCAAAAGACATCCCGGGATCATCCCGATGATCGGCTTAAT

CTGCCTGGGCATGGGCAGCGCTGCGCTCTACTTGCTGCGACTGGCCCTTCGCAGCCCCGACGTCTGCTGGGACAGAAAGA

ACAACCCAGAGCCCTGGAACCGCCTGAGCCCCAATGACCAATACAAGTTCCTTGCAGTTTCCACTGACTATAAGAAGCTA

AAGAAGGACCGGCCAGACTTC

>gibbon

ATGGCAGGAGCCAGTATTGGGGCCCGCTTCTACCGGCAGATCAAAAGACATCCGGGGATCATCCCGATGATCGGCTTAAC

CTGCCTGGGCATGGGCAGCGCTGCGCTCTACTTGCTGCGACTCGCCCTTCGCAGCCCCGACGTCTGCTGGGACAGAAAGA

ACAACCCGGAGCCCTGGAACCGCCTGAGCCCCAATGACCAATACAAGTTCCTTGCAGTTTCCACTGACTATAAGAAGCTA

AAGAAGGACCGGCCAGACTTC

>crabMac

ATGGCAGGAGCCAGTCTTGGGGCCCGCTTCTACCAGCAGATCAAAAGACATCCAGGGATCATCCCGATGATTGGCTTCAT

CTGCCTGGGCATGGGCAGCGCTGGGCTCTACTTGCTGCGACTCGCCCTGCGCAGCCCCGACGTCTGCTGGGACAGAAAGA

ACAACCCGGAGCCCTGGAACCGCCTGAGTCCCAATGACCAATACAAGTTCCTTGCAGTTTCCACTGACTATAAGAAGCTA

AAGAAGGACCGGCCAGACTTC

>rhesus

ATGGCAGGAGCCAGTCTTGGGGCCCGCTTCTACCAGCAGATCAAAAGACATCCGGGGATCATCCCGATGATTGGCTTCAT

CTGCCTGGGCATGGGCAGCGCTGGGCTCTACTTGCTGCGACTCGCCCTGCGCAGCCCCGACGTCTGCTGGGACAGAAAGA

ACAACCCGGAGCCCTGGAACCGCCTGAGTCCCAATGACCAATACAAGTTCCTTGCAGTTTCCACTGACTATAAGAAGCTA

AAGAAGGACCGGCCAGACTTC

>pigMac

ATGGCAGGAGCCAGTCTTGGGGCCCGCTTCTACCAGCAGATCAAAAGACATCCGGGGATCATCCCGATGATTGGCTTCAT

CTGCCTGGGCATGGGCAGCGCTGGGCTCTACTTGCTGCGACTCGCCCTGCGCAGCCCCGACGTCTGCTGGGACAGAAAGA

ACAACCCGGAGCCCTGGAACCGCCTGAGTCCCAATGACCAATACAAGTTCCTTGCAGTTTCCACTGACTATAAGAAGCTA

AAGAAGGACCGGCCAGACTTC

>baboonA

ATGGCAGGAGCCAGTCTTGGGGCCCGCTTCTACCAGCAGATCAAAAGACATCCGGGGATCATCCCGATGATTGGCTTCAT

CTGCCTGGGCATGGGCAGCGCTGGGCTCTACTTGCTGCGACTCGCCCTGCGCAGCCCCGACGTCTGCTGGGACAGAAAGA

ACAACCCGGAGCCCTGGAACCGCCTGAGTCCCAATGACCAATACAAGTTCCTTGCAGTTTCCACTGACTATAAGAAGCTA

AAGAAGGACCGGCCGGACTTC

>baboonH

ATGGCAGGAGCCAGTCTTGGGGCCCGCTTCTACCAGCAGATCAAAAGACATCCGGGGATCATCCCGATGATTGGCTTCAT

CTGCCTGGGCATGGGCAGCGCTGGGCTCTACTTGCTGCGACTCGCCCTGCGCAGCCCCGACGTCTGCTGGGACAGAAAGA

ACAACCCGGAGCCCTGGAACCGCCTGAGTCCCAATGACCAATACAAGTTCCTTGCAGTTTCCACTGACTATAAGAAGCTA

AAGAAGGACCGGCCGGACTTC

>mangabey

ATGGCAGGAGCCAGTCTTGGGGCCCGCTTCTACCAGCAGATCAAAAGACATCCGGGGATCATCCCGATGATTGGCTTCAT

CTGCCTGGGCATGGGCAGCGCTGGGCTCTACTTGCTGCGACTCGCCCTGCGCAGCCCCGACGTCTGCTGGGACAGAAAGA

ACAACCCGGAGCCCTGGAACCGCCTGAGTCCCAATGACCAATACAAGTTCCTTGCAGTTTCCACTGACTATAAGAAGCTA

AAGAAGGACCGGCCGGACTTC

>drill

ATGGCAGGAGCCAGTCTTGGGGCCCGCTTCTACCAGCAGATCAAAAGACATCCAGGGATCATCCCGATGATTGGCTTCAT

CTGCCTGGGCATGGGCAGTGCTGGGCTCTACTTGCTGCGACTCGCCCTGCGCAGCCCCGACGTCTGCTGGGACAGAAAGA

ACAACCCAGAGCCCTGGAACCGCCTGAGTCCCAATGACCAATACAAGTTCCTTGCAGTTTCCACTGACTATAAGAAGCTA

AAGAAGGACCGGCCAGACTTC

>AGM

ATGGCAGGAGCCAGTCTTGGGGCCCGCTTCTACCGGCAGATCAAAAGACATCCAGGGATCATCCCGATGATTGGCTTCAT

CTGCCTGGGCATGGGCAGTGCTGCACTCTACTTGCTGCGACTCGCCCTGCGCAGCCCCGACGTCTGCTGGGACAGAAAGA

ACAACCCGGAGCCCTGGAACCGCCTGAGTCCCAATGACCAATACAAGTTCCTTGCAGTTTCCACTGACTATAAGAAGCTA

AAGAAGGACCGGCCAGACTTC

>snubR

ATGGCAGGAGCCAGTCTTGGGGCCCGCTTCTACCAGCGGATCAAAAGACATCCGGGGATCATCCCGATGATCGGCTTCAT

CTGCCTGGGCATGGGCAGCGCTGCGCTCTACTTGCTGCGACTCGCCCTGCGCAGCCCCGACGTCTGCTGGGACAGAAAGA

ACAACCCGGAGCCCTGGAACCGCCTGAGCCCCAATGACCAATACAAGTTCCTTGCAGTTTCCACTGATTATAAGAAGCTA

AAGAAGGACCGGCCAGACTTC

>snubB

ATGGCAGGAGCCAGTCTTGGGGCCCGCTTCTACCAGCAGATCAAAAGACATCCGGGGATCATCCCGATGATCGGCTTCAT

CTGCCTGGGCATGGGCAGCGCTGCGCTCTACTTGCTGCGACTCGCCCTGCGCAGCCCCGACGTCTGCTGGGACAGAAAGA

ACAACCCGGAGCCCTGGAACCGCCTGAGCCCCAATGACCAATACAAGTTCCTTGCAGTTTCCACTGATTATAAGAAGCTA

AAGAAGGACCGGCCAGACTTC

>colobusR

ATGGCAGGAGCCAGTCTTGGGGCCCGCTTCTACCAGCAGATCAAAAGACATCCGGGGATCATCCCGATGATTGGCTTCAT

CTGCCTGGGCATGGGCAGCGCTGCGCTCTACTTGCTGCGACTCGCCCTGCGCAGCCCCGACGTCTGCTGGGACAGAAAGA

ACAACCCGGAGCCCTGGAACCGCCTGAGCCCCAATGACCAATACAAGTTCCTTGCAGTTTCCACTGACTATAAGAAGCTA

AAGAAGGACCGGCCAGACTTC

>colobus

ATGGCAGGAGCCAGTCTTGGGGCCCGCTTCTACCAGCAGATCAAAAGACATCCGGGGATCATCCCGATGATCGGCTTCAT

CTGCCTGGGCATGGGCAGCGCTGCGCTCTACTTGCTGCGACTCGCCCTGCGCAGCCCCGACGTCTGCTGGGACAGAAAGA

ACAACCCGGAGCCCTGGAACCGCCTGAGCCCCAATGACCAATACAAGTTCCTTGCAGTTTCCACTGACTATAAGAAGCTA

AAGAAGGACCGGCCAGACTTC

>proboscis

ATGGCAGGAGCCAGTCTTGGGGCCCGCTTCTACCAGCAGATCAAAAGACATCCGGGGATCATCCCGATGATCGGCTTCAT

CTGCCTGGGCATGGGCAGCGCTGCGCTCTACTTGCTGCGACTCGCCCTGCGCAGCCCCGACGTCTGCTGGGACAGAAAGA

ACAACCCGGAGCCCTGGAACCGCCTGAGCCCCAATGACCAATACAAGTTCCTTGCAGTTTCCACTGACTATAAGAAGCTA

AAGAAGGACCGGCCAGACTTC

>marmoset

ATGGCAGGAGCCAGTCTTGGGGCCCGCTTGTACCGGCAGATCAAAAGACATCCGGGGCTCATCCCGATGATCGGCTTCAT

CTGCCTGGGCATGGGCAGCGCTGGGCTCTACTTGCTTCGACTTGCCCTGAAAAGCCCCGATGTCTGCTGGGACAGAAAGA

ACAACCCGGAGCCCTGGAACCGTCTGAGCCCCAATGACCAATACAAGTTCTTCGCAGTTACCACTGACTATAAGAACCTG

AAGAAGGACCGGCCAGACTTC

>owl

ATGGCAGGAGCCAGTCTTGGGGCCCGCTTGTACCGGCAGATCAAAAGACATCCGGGGCTCATCCCAATGATCGGCTTCAT

CTGCCTGGGCATGGGCAGCGCTGGGCTCTACTTGCTTCGACTTGCCCTGAAAAGCCCTGACGTCTGCTGGGACAGAAAGA

ACAACCCGGAGCCCTGGAACCGTCTGAGCCCCAATGACCAATACAAGTTCTTCGCAGTTACCACTGACTATAAGAACCTG

AAGAAGGACCGGCCAGACTTC

>SQM

ATGGCAGGAACCAGTCTTGGGGGCCGCTTGTACCGGCAGATCAAAAGACATCCAGAGCTCATCCCGATGATCAGCTTCAT

CTGCTTGGGCATGGGCAGTGCTGGGCTCTACTTGCTTCGACTTGCCCTGAAAAGCCCCGACGTCTGCTGGGACAGAAAGA

ACAACCCGGAGCCCTGGAACCGTCTGAGCCCCAATGACCAATACAAGTTCTTTGCAGTTACCACTGACTATAAGAGCCTG

AAGAAGGACCGGCCAGACTTC

>capuchin

ATGGCAGGAGCCAGTCTTGGGGGCCGCTTGTACCGGCAGATCAAAAGACATCCGGGGCTCATCCCGATGATCGGCTTCAT

CTGCCTGGGCATGGGCAGCGCTGGGCTCTACTTGCTTCGACTTGCCCTGAAAAGCCCCGACGTCTGCTGGGACAGAAAGA

ACAACCCGGAGCCCTGGAACCGTCTGAGCCCCAATGACCAATACAAGTTCTTCGCAGTTACCACTGACTATAAGAACCTG

AAGAAGGACCGGCCAGACTTC

**10) Sequences used for *miR-147b* evolutionary analysis, related to Figure 4**

>NM_032413.3 Homo sapiens chromosome 15 open reading frame 48 (

C15orf48), transcript variant 2, mRNA

GCGTGACCAGAGCGCGCTGGCCCGGCCCACCCGGGGCGGTTGTGGTCGCTATATATAAGGTGGGGAGGCC

GCCGGCCCGTTCGGTTCCGGGCGTTACCATCGTCCGTGCGCACCGCCCGGCGTCCAGGTGAGTCTCCCAT

CTGCAGAGACGCGGACGCGCCGGCCCGCAGTTGGCCTGCGGAGCGCGGTGGACGGTTTGGCGCCCACCAG

GCGATCAATACTTTGGATTTTTAATTTCTAGATTTGGCAATTCTTCGCTGAAGTCATCATGAGCTTTTTC

CAACTCCTGATGAAAAGGAAGGAACTCATTCCCTTGGTGGTGTTCATGACTGTGGCGGCGGGTGGAGCCT

CATCTTTCGCTGTGTATTCTCTTTGGAAAACCGATGTGATCCTTGATCGAAAAAAAAATCCAGAACCTTG

GGAAACTGTGGACCCTACTGTACCTCAAAAGCTTATAACAATCAACCAACAATGGAAACCCATTGAAGAG

TTGCAAAATGTCCAAAGGGTGACCAAATGACGAGCCCTCGCCTCTTTCTTCTGAAGAGTACTCTATAAAT

CTAGTGGAAACATTTCTGCACAAACTAGATTCTGGACACCAGTGTGCGGAAATGCTTCTGCTACATTTTT

AGGGTTTGTCTACATTTTTTGGGCTCTGGATAAGGAATTAAAGGAGTGCAGCAATAACTGCACTGTCTAA

AAGTTTGTGCTTATTTTCTTGTAAATTTGAATATTGCATATTGAAATTTTTGTTTATGATCTATGAATGT

TTTTCTTAAAATTTACAAAGCTTTGTAAATTAGATTTTCTTTAATAAAATGCCATTTGTGCAAGATTTCT

CAAAGATTAGGTATATATTTAAATGGAAGAGAAAATATTTTTATGGGAGAAAAATACATTTGAACCATGA

AATTTCATCTTTTAAATAACATCCAGTACAGATTTCTGTGTAA

>NM_001004174.2 Mus musculus expressed sequence AA467197 (AA467197),

mRNA

ATATAAGGGCCGCTCGCTTCTGGGCGTTAACATCTCCTGGCCGCAGCCGCCCTAGATTTGGAATTCTACA

CTAAAGTCATCATGGGCGTTTTCCAGATATTGATGAAGAATAAGGAACTCATTCCTTTGGCGTTTTTTAT

AAGCGTGGCCGCCACCGGAGCCACATCTTTCGCTTTGTATGCGTTGAAAAAAACCGACGTGGTTATTGAT

CGGAAAAGAAACCCAGAGCCTTGGGAAATGGTGGATCCTACTCAACCCCAAAAGCTTATAACCATCAACC

AGCAATGGAAGCCCGTTGAGGAGCTGCAAAAAGTCCGGAGGGCAACCAGATGATTGCTCACCACTCCTCT

CTTCCAAAGAACACTCTATGAATCTAGTGGAAACATTTCTGCACAAACTAGATGTTGATGCCAGTGTGCG

GAAATGCTTCTGCTACATTTGTAGGGTTTGCCTGCATTCTTTGGATCCTGCATTAGCAAGTGAAGGTAGC

ACATAGTCTAAAATAGTTTTCTGTGTTTATTGGTGTAAATTTCAATTTTACAGTTGAAATTTTATGTTTG

TGATGCTTGGATATTTTCCTTGAAATGTATAAACATGTAAAAATTAGATTACTGCCTGTAATAAAATAAT

TCGATGACTA

>NM_001008518.2 Rattus norvegicus hypothetical LOC302884 (MGC105649),

mRNA

GACACCCTCTGGCCGCAGCCGCCTCAGATTTGGAATTCTACACTAAAGTCATCATGGGCATTTTCCAGAG

ATTGATGAAAAATAAGGAACTCATTCCTTTGGCGTTTTTTATAAGCGCGGCAGCCACTGGAGCCTCATCT

TTTGCTTTGTATGCTTTGAAAAAAACCGACGTGGTTATTGATCGAAAAAGAAACCCAGAACCTTGGGAAA

CGGTGGATCCTACTCAACCCCAAAAGCTTTTAACCATCAACCAGGAATGGAAGCCTGTTGAGGAGCTGCA

GAGAGTCCGGAGAGCGACCAGATGATTACTCGCTGCTCCTTTCTTCCAAAGAACACTCTATGAATCTAGT

GGAAACACTTCTGCACAAACTCGATTTTGATGCCAGTGTGCGGAAATGCTTCTGCTACATTTTTAGGGTT

TGCCTGCATTCTTTGGATCCTGCATAAGCAAGCGAAGGTAGCACATAGTCTAAAATGGTTTTTCGTGTTT

ATTGGTGTAAATTTCAATTTTACAGTTGAAATTTTATGTTTGTGATGGTTGGATATTTTCCTTGAAATGT

ATAAACATGTAAAAATTAGATTACTGCCTGTAATAAATAATTAGATGGCTATCTGTAAAACGTATGACCT

GTGCAAGAGATATCAAAAATTACTTAACTTTAAAAGTGGAGGGGGCATCTTTTTATGGGAACAAGCACAT

TTTGAAGTATAAAACTTCAACTTTTAAATATCAAGTGCATATTTCTGAGTTTTATTTCCAAGTCCATTCA

CTCTCACCCCAGAAACTTCTTGAGCATGTATTGTTTGCAGTAAAGAAAAAGGGTGCAAAGTTAGCAAGGG

TTTGGATATGGTCCGTTTGGAGCATGGAGACAGCTGAGAGGGCTGCCTGGGAATGGCTGGTGACCAGGAA

CCTGAGGCATGCAGAGACCAGCCCTGCAGATCTCCGTGGGACTGCTGCTCCCCAGGAGGATGGAGAGAGG

AGCCTGGGGAGCCCTGGCCAGCACCCAGTACAGTCTGACCAAACAGAGACCAGGCGATTGTTTAATTCTT

TCCAGAATGTGCCCCCAACCCCATTCCTTGCCGACTCCTAAAAATGGGACGAAGTAGGAAGTTCCTTTTC

ATTGACTGTAAAATAAATACAGCTATAATTCAAAAAAAAAAAAAAAAAAAAAAA

>NR_034989.1 Monodelphis domestica microRNA mir-147b (MIR147B),

microRNA

TCTATGAATCTAGTGGAAACATCTCCGCACAAACTAGACTACTGAAACCAGTGTGCGGAAGTGCTTCTGC

TACATTTTTAGG

>BX950500.1 Gallus gallus finished cDNA, clone ChEST541b1

AGCTTTCTTGCCTTAAGATGGCACGTTATGCTTGAACCAGATTGGGTGTTTGGTGCTCTGGAAGCCCCAG

GAAAACAAGCATAATTGATTCCCATCCAGCACTGAAAAGAGAAATTCCCAAAGCTGTATAGATGTGCTTC

AGGGAACTGCTTACCTACAGGAGTAGCTGTGCTGATCATAGATTGCTCATACAGGAAAAGCAGATGTAAG

TTCACAGCCTAAAACACGGTACCTCTTTGCTTTATCTTCTCTTAAAGCATTAACAAGAGTGGCAATCCGG

AGCCATGGGAAACCGTTGATCCTACCCGGCCTCAGAAGCTCTTAACAATCCATCAGAAATGGAAACCTAT

CGAAGAGTTGGAAAGTGTCAAAAAGCTTACGAAGTGACAAGTTTCTGTTGCCTCTAAAGGTACTCTATGA

ATCTAGTGGAATCACTTCTGCACAAACTTGACTACTGAAATCAGTGTGCGGAAATGCTTCTGCTACATTT

TTAGGGTCTCCCTCCATTTTTTGGGCTCTGGATGAGGAGTTGCATGTTGCCTTAGAAGTTGGCATACAAC

ATCCACTTAGTCCAAAAATCTGTGTCAAAGCTCATTTTCAGCAGGCTTTTGCCATCAGATGTTGAACGTT

CGGATGGTAATTGAATATGGAAAAAGTTTTTGTGCTTTTTACAATAGAATTTTGGAGTTTGTTTTCTCAA

AGTTAATACTGTAGTTTTATCATAATAAAATCTGTTTAACATTTTGCTT

>anoCar2_dna range=chrUn_GL343506:18009-18194 5'pad=0 3'pad=0 strand=- repeatMasking=none

CTGATAACAATCAACCAGGAATGGAAGCCCATAGAAGAGCTGGAGAAAGTCAAAAAAATGATGAAGTGAT

GGGTGCCTGCCTCTTCACGTGACACTCTATGAATCTAGTGGAATCACTTCCGCACAAACTAGATGAATGA

AACCAGTGTGCGGAAATGCTTCTGCTACATTTTTAGGGTCTCCCTC

>XM_004066897.4 PREDICTED: Oryzias latipes chromosome 3 C15orf48

homolog (c3h15orf48), mRNA

TGTCATCCGCAGGTGGGATCTGTTGCTCACTACATCCCCTACATTTCAAACAGCAACTTTTTTACATAGT

GGATGCTACACTTTAAATAAAAAATAAAAATACCCATCCTCAGGTGTTTGAAAACTCTGCAAAGATGTCT

GGCTTTTTCCAGATGTTGAAGAAAAGAAAGGAGCTGATCCCTCTCATAGGATTCATGGCTTTTGCTGCAA

CAGGTGCCACAACTGCTTCTCTCTACTTTTTATTTACTAAACCGGATGTTATTCTAAACAAGTCATCCAA

CCCGGAACCATGGGAAAGACTGGATCCATCCAAACCCCAAAAGCTTATCACCATCAATCAGGAGTGGAAA

CCTGTGAAGGAGCTGGAGATAGTGAGGTCCCTCACCAAGTAAAAAGAAAAAAAAGAAAAGTAAGAAGAAA

AGGGTTCAAGATGCAGAATCCGACTCCATGGCCCCCTCATCAGTCTAACAGAATCCTTTCTGCACAAACT

AGGATGTTTTTACGGTCAGTGTGCGGAAATGCTTCTGCTTAACTGGTGGGGTTTCACCCTGCTCATTGGC

TTCAAAGAAGAAACTTCCATCCTCCTCATCTGTGTGATACACGCGTGTAAACGGTAGCTATAGGAATGTT

TTTGCACTGAAATCAGTCATGATTGTTGTAATGAGTTTGTTTTGTGCAGATGTTCTGCTGTAATTGCTGT

CTGTTAATGTTCTGTTTGAGTATATGTACTTTACAGTGTCAATAAAACATTGGTCCATGTA

>NM_001198748.1 Danio rerio zgc:153317 (zgc:153317), mRNA

ATCAAACGCATATCAAAGTCAATATAAGAGCAGGAGAGTCACCATGATGCTGCATTTACACCTCCAGCTC

TGTTCTCTCCCATCATCAGCTCATCGAGACTCGTCTTTACAGAGTAATCTGTAACTGCTGAAAGATGAAC

GGAGGATTGATACAGTTGCTCAGGAAAAGGAAAGAGTTAATTCCTCTGCTTGGGATTGTTAGTTGTGCTG

CATTTGGAGCGACAACAACCATGATTTACTTCCTGCTGACCAAACCCGATGTCATTTTAAACAAGACGGG

AAATCCCGAGCCCTGGGAGATGCTGGATCCATCAAAACCACAGAAGCTCCTCACCATTAACCAGCAGTGG

AAACCAGTGGAGGAGCTGGAGATGGTCAAGAAGATGACCAAATGACCATGATGCACACTGCACATACGGT

CTGGGGCTGCACAATATATTGTTCCAGCATCGATATCTCCATGTGTCTGTTGAGTTTGATTATAGCTGAT

CAGCAGTGGAGACTGTGTTCATTTACATTACATTTGATTATTCAACTGACTTCAGTTTGACTCCTGGCAA

AACCATAAAGCACTGCTCATTTCACTTTCATCTCTGCTCTGTCTTATTTCTTTACTCCATTACAGGATCA

ATCTGAAGATGCCAATCCAAATACAGATACTCAAGTCACCTGCTGAAATTGTACTCATATCAGAATATAT

ATCATATACTAATAAAACATATATCATGCAG

>XM_006628639.2 PREDICTED: Lepisosteus oculatus chromosome LG3 open

reading frame, human C15orf48 (clg3h15orf48), mRNA

GTGCGTATGACTGTCTACGTGACTTGGACCTCGTCTCAATTTCCGAAAACGATATAAGGGGGGAGTGGCG

GCGCAGGCAATCAGAGATCTGCCAGACAACTTTCGCAGCGGGTTGAAGGCACCACCAGGAAATAAAAATG

TCTGCGTTTTTCCAAATGTTAAGGAAAAAGAAAGAACTTATTCCGTTAATTGGTATTATGACCTTTGCAG

CAACTGGTGCCACTACCGCCTGCCTTTACTTCTTGTTCACTAAATCGGATGTCATCATTAACAAGGCAGG

GAATCCCGAACCGTGGGAGAACCTAGATCCAAGAAAACCCCAAAAGCTCATTACAATAAACCAGCAGTGG

AAGCCCGTGGAGGAGCTGCAGATGGTGAAGAGCATAACCAAGTAGAGATGACCCTGCCTGCTCTAAAGTC

TCTGTGTCCACCAGCCTGCCTGCACCCTATCAATCTAACGGAATCATTTCTGCACAGACTAGACTCTGAG

AACCAGTGTGCGGAAATGCTTCTGCTACATTGGTAGGGTCTGACCACCAACAGGTCTGTGGGACTGTAGT

AGGTCCTTCAGCTGGAATTCACTAAAGCAAGCGCCGCACAGCTAGGAAAACAGAGGACTGAACATTCTGG

CAGCTCGTCCTCATTAAACCATGTGATTGTATTATAGCAAAATGTGACTGATTACCCTTATTCACTATAC

TGATTAACATAGTGTACATAATGGCCTATAAAATAAAGAATGTAGTTCAAAGAT

**11) Sequences used for *miR-210* evolutionary analysis, related to Figure 4**

>NR_029623.1 Homo sapiens microRNA 210 (MIR210), microRNA

ACCCGGCAGTGCCTCCAGGCGCAGGGCAGCCCCTGCCCACCGCACACTGCGCTGCCCCAGACCCACTGTG

CGTGTGACAGCGGCTGATCTGTGCCTGGGCAGCGCGACCC

>NR_029793.1 Mus musculus microRNA 210 (Mir210), microRNA

CCGGGGCAGTCCCTCCAGGCTCAGGACAGCCACTGCCCACCGCACACTGCGTTGCTCCGGACCCACTGTG

CGTGTGACAGCGGCTGATCTGTCCCTGGGCAGCGCGAACC

>NR_031923.1 Rattus norvegicus microRNA 210 (Mir210), microRNA

CCGGGGCAGTCCCTCCAGGCTCAGGACAGCCACTGCCCACAGCACACTGCGTTGCTCCGGACCCACTGTG

CGTGTGACAGCGGCTGATCTGTCCCTGGGCAGCGCGAACC

>NR_034982.1 Monodelphis domestica microRNA mir-210 (MIR210), microRNA

CCGGCCGCAGGGGAGCCACTGACTAACGCACATTGCGCTCAGGACCCACTGTGCGTGTGACAGCGGCTAC

CGTGCAACCGGC

>NR_129921.1 Anolis carolinensis microRNA mir-210 (mir210), microRNA

CCAGTTCTCCAGGAGCAGATGAGCCACTGACTAACGCACATTGTGCTTCTCGTGTCCCCACTGTGCGTGT

GACAGCGGCTAACCTGCTTTTCGGAC

>NR_029936.1 Danio rerio microRNA 210 (mir210), microRNA

GCAGGTAAGCCACTGACTAACGCACATTGCGCCTATTCTCCACTCCACTGTGCGTGTGACAGCGGCTAAC

CAG

>NR_107218.1 Oryzias latipes microRNA mir-210 (mir210), microRNA

TCCGAGTTCTAAAAGCAGGTAAGCCACTGACTAACGCACATTGTGCGTGTGACAGATCCACTGTGCGTGT

GACAGCGGCTAACCTGGTTTTGGGAAAACCTCTGA

>NR_036386.1 Strongylocentrotus purpuratus microRNA mir-210 (Mir210),

microRNA

CTCGTTAGTTGCTGTCACGCGGCACAAGAGAGCAATCATGTCTATACACTCTTGTGCGTGCGACAGCGAC

TGATACA

>NR_047831.1 Drosophila melanogaster mir-210 precursor RNA (mir-210),

miRNA

AAAGGTGCTTATTGCAGCTGCTGGCCACTGCACAAGATTAGACTTAAGACTCTTGTGCGTGTGACAGCGG

CTATTGTAAGAGGCCATAGAAGCAACAGCC

**12) Sequences used for *MISTR1*/*NDUFA4* MRE evolutionary analysis, related to Figure 4**

>NM_002489.4 Homo sapiens NDUFA4 mitochondrial complex associated

(NDUFA4), mRNA

GGAAGTCCGTAGTGTCTCATTGCAGATAATTTTTAGCTTAGGGCCTGGTGGCTAGGTCGGTTCTCTCCTT

TCCAGTCGGAGACCTCTGCCGCAAACATGCTCCGCCAGATCATCGGTCAGGCCAAGAAGCATCCGAGCTT

GATCCCCCTCTTTGTATTTATTGGAACTGGAGCTACTGGAGCAACACTGTATCTCTTGCGTCTGGCATTG

TTCAATCCAGATGTTTGTTGGGACAGAAATAACCCAGAGCCCTGGAACAAACTGGGTCCCAATGATCAAT

ACAAGTTCTACTCAGTGAATGTGGATTACAGCAAGCTGAAGAAGGAACGTCCAGATTTCTAAATGAAATG

TTTCACTATAACGCTGCTTTAGAATGAAGGTCTTCCAGAAGCCACATCCGCACAATTTTCCACTTAACCA

GGAAATATTTCTCCTCTAAATGCATGAAATCATGTTGGAGATCTCTATTGTAATCTCTATTGGAGATTAC

AATGATTAAATCAATAAATAACTGAAACTTGATATGTGTCACTTTTTTATGCTGAAAGTATGCTCTGAAC

TTTAGAGTATAGGAAATTAACTATTAGAATTTAAAGAATTTCTTGAATTTCTGTAGTTTGAAAATACGAC

TTTAAGCTGCTTTAGTAAAACACTTCCATTTTGTGTATAGACTGTTGGTAACTTCACTAGAGCATACATA

ACAACTGGAACTGGAAATTATACAAAAGTAAATTGGGAAGGATACTCCAGCATCTGACACTGGCAAAATG

GAAACCTTTGAGTTTCTCTTACTGGCTGTTGAAGTGTGTGCAGTTTTTAACAATGGTTTTTACTTGGCAT

CTCTTTGTTGTGATTTTCAAGGTTATAAGTTGCTTTGGTCCTAGGATTGAAGTTGAAATCTGAGTTTATC

AGTGCTAACCATGGTGCTAGTAGTCAAGAGATCTTGAGAATTTTGGCTGCTGAGTCTTGGTGCAGGGTGC

AGGTTTTCTTTTCTTTTTTCTTTTTTTTTTTTTTGAGATAGTCTCTGTCACCCAGGCTGGAGTGCAGTGG

TACAAACATGGATCACTGCAGCCTCTACCTCCCGGGCTTAAGTGATCCTCCTGCCTCAGCCCCTAAGTAG

CCGGGACTACAGGTATGTGCCACCATGCCCAGTTAATTTTTGTAATTTTTTTTAGAGACAGGGTTTTGCC

ATGTTGCCCAGGCTGGTCTCAAACTCTTGAGCTCAAGCGATCCATTCTCCTCAGCCTCCCAGGGTGCTGG

GATTACAGGCGTGAGCCATTGCGCTTAGCCATGGTGCAGGTTTTCAAAGGCCAGGAAGTATATTCATAAT

TTTAAGATGGGGAATATAGCAAGTTTTCACATAGGTGTGTGTAAGTCATCACATCATAGAAACTTGAGGA

ATTCAGTGACATTAATTTTGGATTTTCATACGTAAGTATACAATTAAATGTTTACAGGGTAGTAGAAGCA

CATTTTAAATGTCAGGAACTGAACTAAGTATTTGAATTACGTGGATTATCTCAAAAATTTTGAAATTGTT

AAACGAGTTGAATTACTTGAATTCATTCTGTTAGTCAAATGGTGGATATTTACACCCATGTAGTTTTGAA

TTTAGAGTGTGTAGAGTGTTTTCAGTTACCAGACTCCATGCTTTTACCTCCTATGTGTCAGGTATAATTT

GAACCTCTAAGAACAGGGTTTCTCAACCTTGCCACTGTTGACTATTTCTGAAAGACAGTTTGGTTTAGCA

GACCATCCCATGCGCTTTAGCTTGTTTAGTAGCTAACTTGGGCTCTGCCACTACAGACAAAAAGCACTCT

TTCCCTCCAATTCCCACAGGCTATGAGAAGAATGGAGACATTACCAAATGTCCATTGGTGGGCAAAATTG

CTTCATTCCTACCTCTGTTGAGAATTACTCTAGATCCTTTGGCACAAATTACCTCAAAGTTTAAAATTGT

GTAAACAAACAGTGTGTCATGTAATTGAAAAACATTAAGCAACTCCAAATAAATGCTACATTAAGAAATT

AGTAA

>NM_010886.2 MUS MUSCULUS NADH DEHYDROGENASE (UBIQUINONE) 1 ALPHA

SUBCOMPLEX, 4 (NDUFA4), MRNA

GTCCGCTCAGCCAGGTTGCAGAAGCGGCTTAGCGTGTGTCCTAATCTTCTCTCTGCGTGTAGGTAGGCCT

GTGCCGCAAACATGCTCCGCCAGATCCTCGGGCAAGCCAAGAAGCATCCCAGCTTGATTCCTCTCTTCGT

ATTTATTGGAGCAGGGGGTACTGGAGCAGCACTGTATGTGATGCGCTTGGCACTGTTTAATCCAGATGTC

AGCTGGGACAGAAAGAACAACCCAGAGCCATGGAACAAACTGGGTCCCAATGAACAATATAAGTTCTACT

CTGTGAATGTGGACTACAGCAAACTGAAGAAAGAAGGCCCAGACTTCTAAACTATGAAGTTCACTGTAAA

GCTGCTGATAATGAAGGTCTTTCAGAAGCCATCCGCACAATTTTCCACTTAAGCAGGAAATATGTCTCTG

AATGCATGAAATCATGTTGATTTTTTTTTTTTTTGGAGTTTATTACACTGATGAATAAATCTCTGAAACT

TG

>NM_001127684.2 RATTUS NORVEGICUS NDUFA4, MITOCHONDRIAL COMPLEX

ASSOCIATED (NDUFA4), MRNA

GGGGTCCTTCAGGTAGGAGGTCCTGGGTGACTTTGGACGTCCGCTCAGCCAGGTTGCAGAAGCGGTTTAG

TGTGTGTCCTAATCTTCTCTCTCGGTGTAGGTAGGCCTGTGCCGCAAACATGCTCCGCCAGATCCTCGGG

CAAGCCAAGAAACATCCCAGCTTGATCCCTCTCTTCGTGTTTATTGGAGCAGGGGGTACTGGAGCAGCAC

TGTATGTGATGCGCTTGGCATTGTTCAATCCAGATGTCAGCTGGGACAGGAAGAATAACCCAGAGCCTTG

GAACAAACTGGGTCCCAATGAACAATATAAGTTCTATTCTGTGAACGTGGACTACAGCAAACTGAAAAAA

GAAGGCCCAGACTTCTAAACTGTGAAGTTCACTGCAAAGCTGCTTACAATGAAGGTCTTTCAGAAGCCAT

CCGCACAATTTTCCACTTAAGCAGGAACTATGTCTCCGAATGCATGAAATCATGTTGATTTTTTTTTTTG

AGTTTATTACACTGATGAATAAATCTCTGAAACTTGATATGTGTCACTATTTAATGCTGAAAATTCATAT

GGGATTTGATAGCTAGGATATAAGAAATAAAGTATCAGGATTTAAAGAATCTCTTGAATTTCTCTTTATA

CATTTGAAAGTGAGACCTATGCTGCTTTAATAAGGGTGTGTGTGTATGTGTATATAGGCCCACACTTGAT

AACTTCAAACTGGAAATTAAAGGGCTTCTGGGAAGAATATTCACGTCTTTGTCAAATGAACACAATAGAA

AGCTCTGGATTTCTG

>XM_001376447.3 PREDICTED: MONODELPHIS DOMESTICA NDUFA4, MITOCHONDRIAL

COMPLEX ASSOCIATED (NDUFA4), MRNA

CGTTTGCGCGTGCGTGCGTTCCGCAGCGCCTGTCCTGCCTTCTCCTCTTCTTTTGCGGTGACATTGGACG

CTGAGCCAGGGACAAGACGCAAAGGAGCTAAGAAGCGAGGGAACGAACGTAGCCGCCGAGTCCCACTGCA

GCCGCCGCTATGTTTCGTATGATCATCAACCAGGCCAAGAAGCACCCCAGCCTGATCCCCCTGTTCCTGT

TTATCGGGGCTGGGGGTGGAGGGGCCACACTCTACGTGATGCGTCTGGCACTCTTCAACCCTGATGTCAG

CTGGGACAGGAAGAACAACCCTGAGCCTTGGAACAAGCTGAATCCCACTGATCAGTACAAGTTCTACTCG

GTCAATGTGGACTACAGCAAGCTGAAGAAGGAAGGGCCGGAATTCTGAGTGAGCCCCAGGCTGCCGAGCC

CCAGTGAAGGTCTTCCAAAAGCCATCCGCACAATTCTCCCTCCACTAGGAGATGCTCCGTCTCCCCTTCC

TTTGTATGCATGAATCATGACAGTGAATAAAGATGTGAAACGTG

>NM_001302107.1 GALLUS GALLUS NDUFA4, MITOCHONDRIAL COMPLEX ASSOCIATED

(NDUFA4), MRNA

GAGGGCACGAGGAGCGATGTTAGCGCGGATGCTGCGGCGGTGCGCCGGCCGGGCTGGGCTTAGCGGCAGG

CGAACAGGCAACCGCCGGAGCTGCTATATCCGGGCAGCGGGCACCTGCCGCGTTGGGGGCTTCCTGTGAC

ATTGGGCCTCGGCAGCAGAAGGGGCAGGACGGTCTCCGGAGCTGGGTAGCGAGGGAGACCTTCACCAGCT

GCAGCTATGTTTCGTGTTATGGTCAACCACGCCAGGAAGCACCCCAGTTTGATCCCTCTGTTTCTGATCA

TTGGATCAGGAGGCATTGGCGCAGCTCTGTACGTCATGCGTTTGGCAGTGTTCAACCCTGATGTCTGCTG

GGACAAGAAAAATAATCCAGAACCTTGGAATAAACTGGCTCCCAATGACCAGTACAAGTTCTACTCAGTC

AACGTAGACTACAGTAAGCTCAAAAAGGATCGTCCTGACTTCTGAAGAACGCACACATCTCCACGAGCTG

CACAGAAAGGTCTTCCGGAAGCTGTCCGCACAATTACCATGCTTAATCATCCAGGAAATATTCAAACATG

CCTCACGCTGCACTTGGTCATGTGTTTGAAAGTGTTTTGATGGGGTTTAATAAACGTCTGAAACTTGAAG

TGTCTTCTGTTTGCTTTAATTTCGCTAAATAAGAACATCCTGACATTAACAAACACTGATTCATATTTGC

TCTACAAGAATTGAAAGTTGTTTTGATGGGCATTATACTCAGTGTGTTGACTTAAAATGAGAACTTGAGA

GCAAATACTTAAATGTGTATGTATATATATTTTTAATTGCACTTAAATTTTTCTAGTTGCTACTTTATAT

AGCAGACAAGTCATTGCTAATAAGTCATTGCCCAACAGCAAAGCAACAGATGTTGCCATATGCTCCTGAA

AATAAAAAACCCAAGTTCTGTGGAAAAAAAAAAAAAAAAAAAA

>XM_008124000.1 PREDICTED: ANOLIS CAROLINENSIS CYTOCHROME C OXIDASE

SUBUNIT NDUFA4 (LOC103281795), MRNA

GGCAAATTCAGAGGGAGGGAAATCCTCCGGTCTGTCTAAACTACTGCCTGATCTGAAGAAAAGCCAGCAA

GGGTTGACTTTGAGCCCGAGCGCATGCGTACCAAAGGAGAGAGACGACGCGGAGAAAGGAGAGAAGGAGA

CTGAGAAGGGCGCGTTACTCTCGTGGAGCTCCGTGTTTTCTCCGCCATCATGTTCCGTCTCATGGCCAGC

CAGGCTAGGAAACATCCCAGCTTGATTCCTCTCTTCATCTTCATCGGAGCTGGAGGGACCGGGGCCGCCC

TCTACATCATGCGTCTTGCTCTCCGAAATCCTGACGTCTGCTGGGACAAAAAGAATAATCCCGAGCCTTG

GAACAAGCTGGGTCCCAATGACCAGTACAAGTTCTTTGCTGTGTCCACCGACTACAGCAAACTGAAGAAA

GAAGGGCCGGACTACTGAGCGCCATCGCCTGGCGTTTCTTACCTTCCCTCCACAACAAGCTGCACAAAAG

AACCCGAAGAAGGTCTGTCTTGAGGTCTTCCAGAAGCCATCCGCACAAGGCATCTCCAATACTTCTCCTC

CTCCATCGTCCAGGAAATTAACCATTTCTCTTTTCGCACGTTGCACTTCAATCATGTTTTCAAAAGGTTA

AGTAGTTAGTCGGGGAGGGAAAAGAAGGAGCTTGTTGGGACTTAATAAAAACCAGTGCTTGAAATCTTAA

>NM_213417.2 Danio rerio NADH:ubiquinone oxidoreductase subunit A4,

like (ndufa4l), mRNA

AAGCCCCCCGCGGACTGTGTCCTTTCTCAGTTCTGAAATAGACCTGCTGGAGCTCGCTAACGGGCCGCGT

TTAATCCCTTTATTTCTCTACAGTTTTGGGAGATTTGATATCATCTCATCTATCACGATGCTGAGCATGG

TCAGCCGGCAACTCAGGAGCCATCCTGCCTTGATCCCACTGTTTATTTTCATTGGCGGCGGATGCACCAT

GTCTCTGTCATATCTGGCTCGCCTGGCCTTGCGTAACCCTGATGTCTGCTGGGACAAAAAGAACAACCCA

GAGCCCTGGAATAAAATGGGGCCCACTGATCAGTACAAGTTCTACGCTGTGAACATGGACTACAGCAAGC

TGAAGAAGAACGGTCCTGACTTCTAAACTCATCCAGACGCCCAGTGCTGGACCAAAGGTTTAAAACAAAC

TAAAGATCAACAAGATGTCCGCACACTTCTCCTCAACCGGCCCCTTGTTTCTATATTAATTTATGCAAAG

AGACTAAATTGCACAGTGTTGTCTTTGCTGGGTCCCTTTTTAACATCTGTGATGTCTCTTGTACTTTTTC

TTGGTTCTTTGTAGTCAGATTTGATTAATAAAGGGTTTTTCCCAACATACATCGAAAAAAAAAAAAAAAA

>XM_004071194.3 PREDICTED: ORYZIAS LATIPES CYTOCHROME C OXIDASE

SUBUNIT NDUFA4 (LOC101155111), MRNA

TGGCATCCCAATCATTCCTCCTTGGCCTTCTTCCGCGAAATCTCGCAGATTACGCTGCGCAGCGTTTTGG

TGTGTGAGGTTATAATCGGTTTATTTCTACACAAACCAATCTGTACGGGCAACATAATGCTCGTTACAAT

CCGCAAACAGCTACGAAACCACCCGGCGCTGATCCCCCTCTTCTTCTTCATTGGGGGAGGCGCCGCCATG

TCCATGCTGTACCTGGCTCGATTGGGCTTGAGAAATCCTGATGTCAGTTGGGACCGCAAGAACAACCCCG

AGCCTTGGAACAAACTCGGCCCGACTGATCAGTACAAGTTCTTCGCTGTAAACATGGACTACAGCAAACT

GAAGAAAGACCGTCCCGACTTCTAAAGATCAAACCTGCTGGACCAAAAGCGCAAGAAGAAAATCCGCACA

ACCATCTCCATTGGGAATTTTCATTTGGGAGAGATGCAGATGCTAAATCCGCACATTTATTTAAGTTTCC

TCATCGCACCTCATCTCATTACCATTTAACTTTTTCTGTGTGCTTCAATAAAAAAAGGAGTTTTCTCCTT

CATACAAGAAAAAAAAAA

>XM_006638099.2 PREDICTED: LEPISOSTEUS OCULATUS NDUFA4, MITOCHONDRIAL

COMPLEX ASSOCIATED (NDUFA4), MRNA

CCACACCGCAGTCCTTTACAGGAAAAGTAAGAAGGAGAGTATACCACAGACGCGTCGAAAGGCCTCGCAG

ACGGCACTTTTTTTTTTGCGTTTCTTTGGGATAAAGAAAACCAAACTACTTTTAAAATGTTCCGTACTAT

GGTTGTCCAGGCTAGAAAGCACCCCAGCTTGATCCCCCTGTTCGTCTTCATCGGCTCAGGGGCGGTGGGG

GCCACCCTCTACCTTGCACGCCTGGCCTTGCGCAATCCTGATGTCTCCTGGGATCGCAAGAATAACCCTG

AGCCCTGGAACAAACTGGGGCCCAATGACCGGTACAAGTTCTTTGCCGTAAACATGGATTACAACAAATT

AAAGAAGAACGGGCCAGACTTCTAAGCTCACCTCTCCTGCCAGCTACAGGACCAAGGTCTTTAGAAGCCA

CTCCGCACAATCAACAGGAGCCAAAAAGAAAAAAACAGAAGCAGAACCGCACAAAAAAGCCATTTGCGTT

CAGGAATGCATCTCGCCCTCAGCTTTCCGCACAGAACTTCGTACCATCCTTCCGATATTTATGGTTTGCT

GCCTCAATAAAATAAAGCAGTTTGCCACCTACTGAATCGGTGTGAAACTG
