# Supplementary material for: Signatures of host–pathogen evolutionary conflict reveal MISTR—A conserved MItochondrial STress Response network
Source: PLoS Biol. 2020 Dec 28;18(12):e3001045. doi: 10.1371/journal.pbio.3001045 (PMC7793259; doi:10.1371/journal.pbio.3001045)

Uncropped Western Blot Images

Notes:
Chemiluminescence Western blots were imaged using the ChemiDoc MP Imager (Bio-Rad).
PVDF membranes were cut to allow for simultaneous immunoblotting of different size proteins.
“L” indicates molecular weight ladder.
“X” indicates a lane excluded from the final figure shown in the paper.
Approximate molecular weight of the bands are indicated on the right side of each blot.

Fig 1G

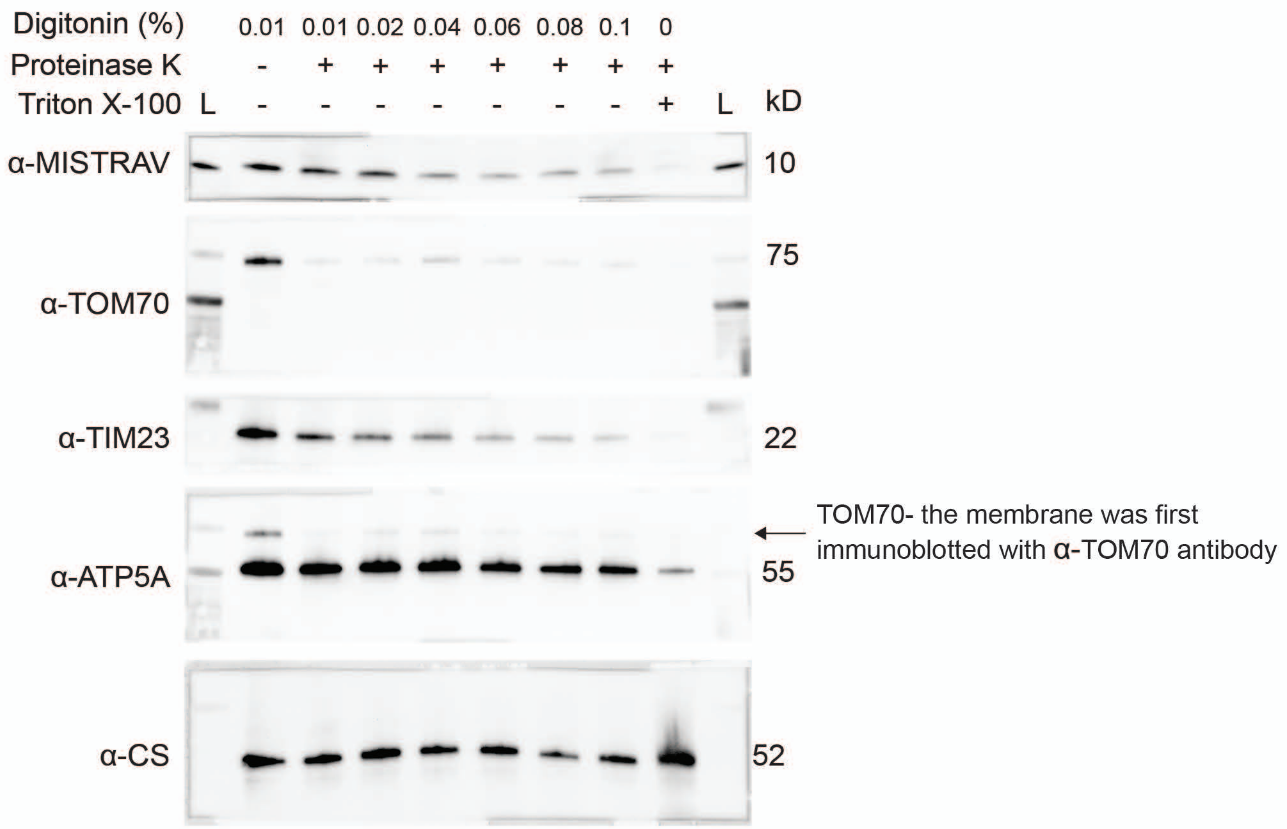

Fig 1H

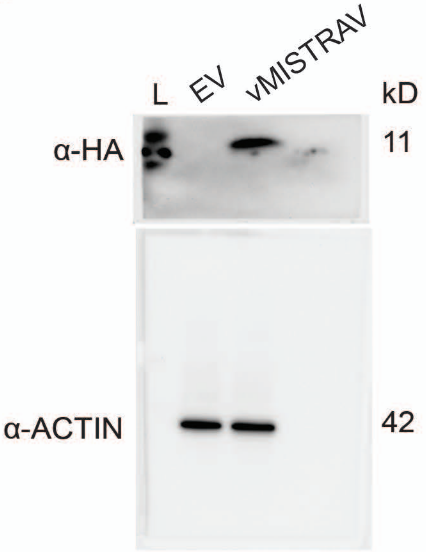

Fig 1I

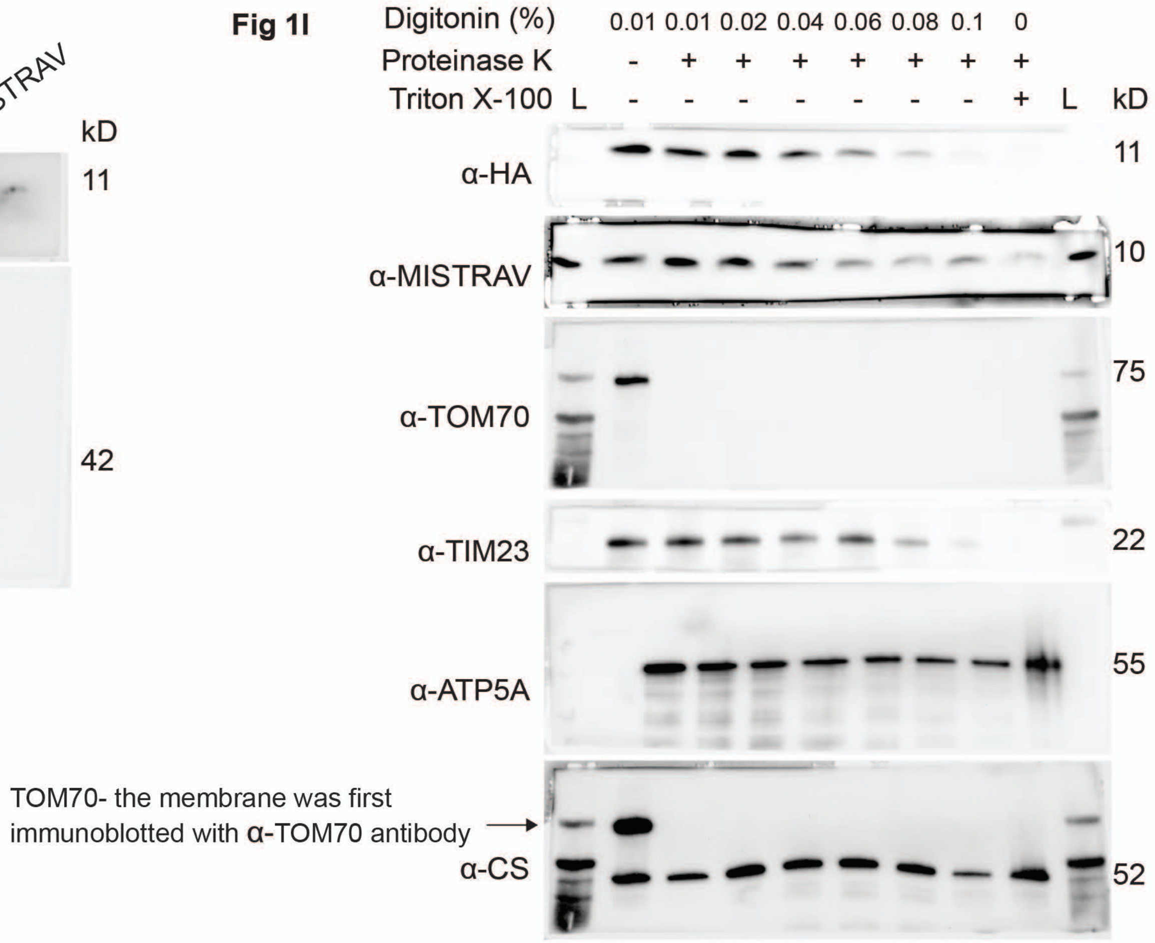

Fig 3B

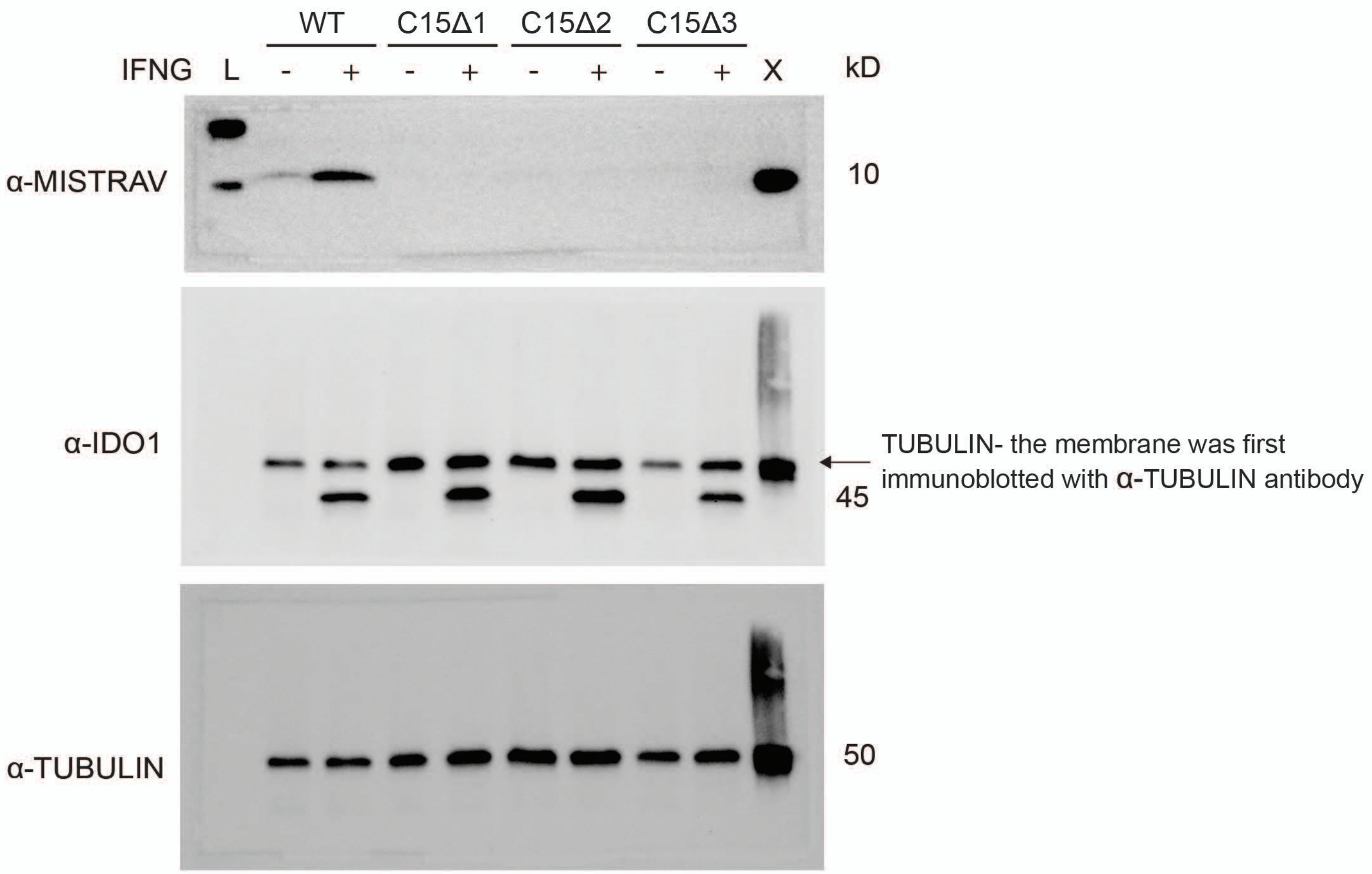

Fig 3E

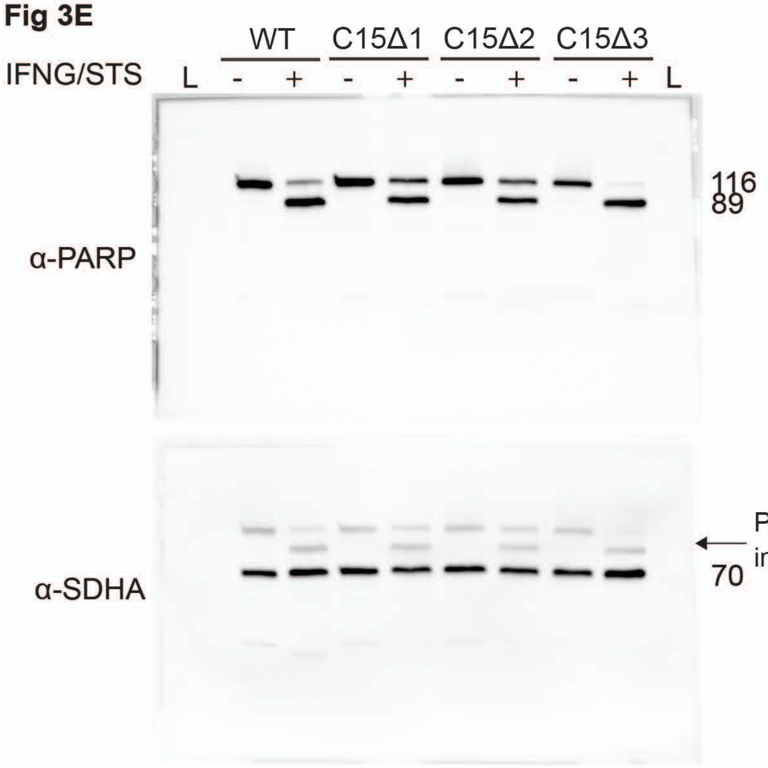

Fig 3H

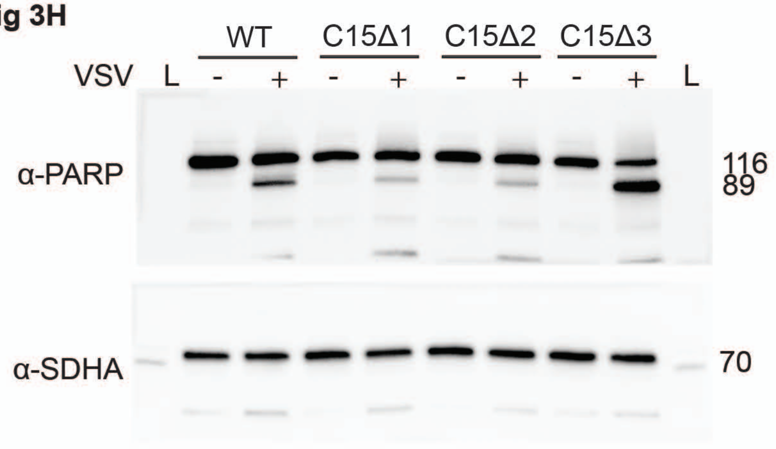

Fig 5C

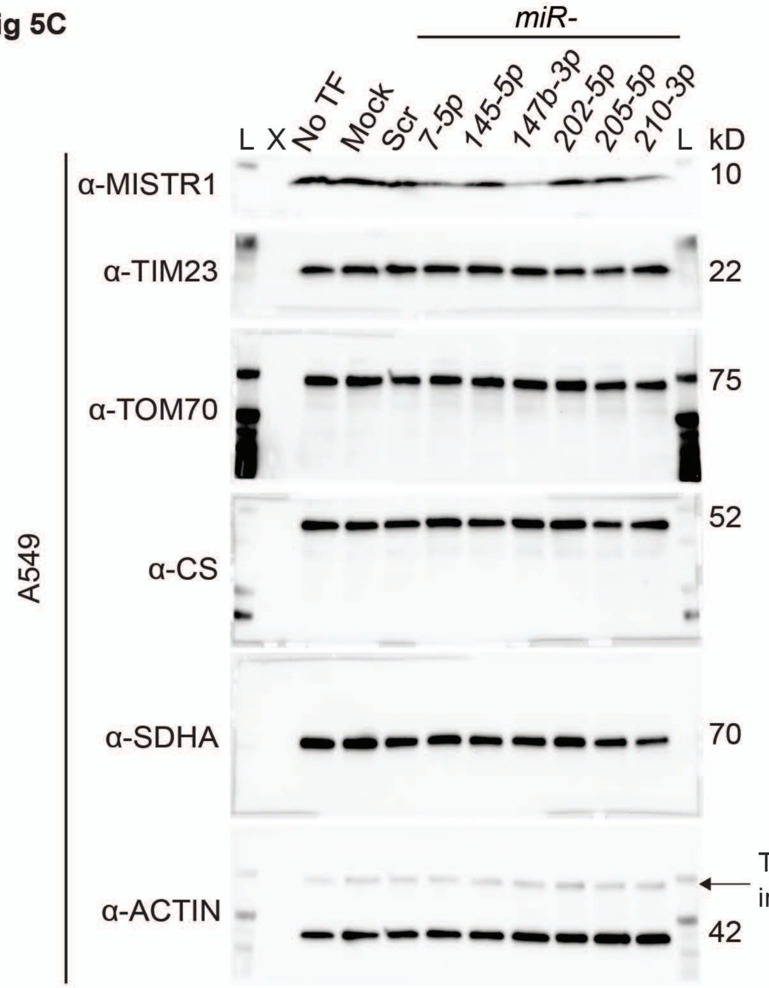

TOM70- the membrane was first immunoblotted with α-TOM70 antibody

Fig 5J

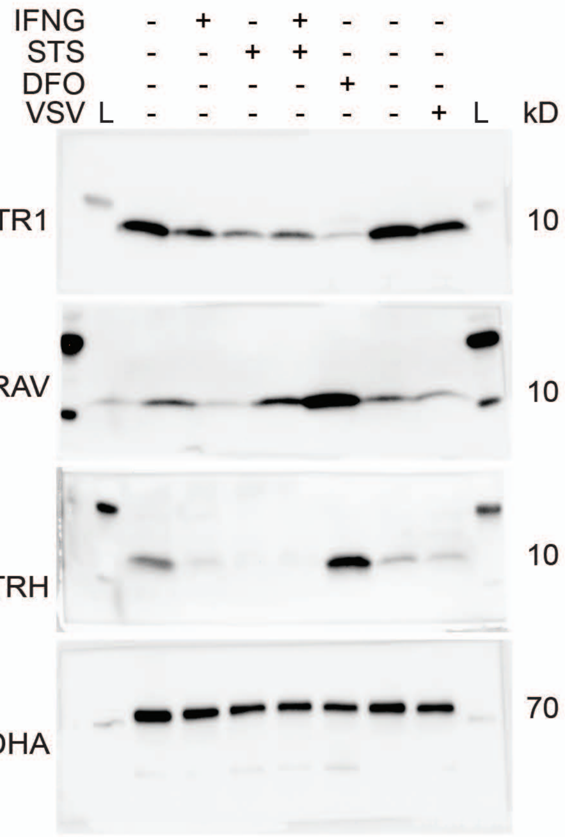

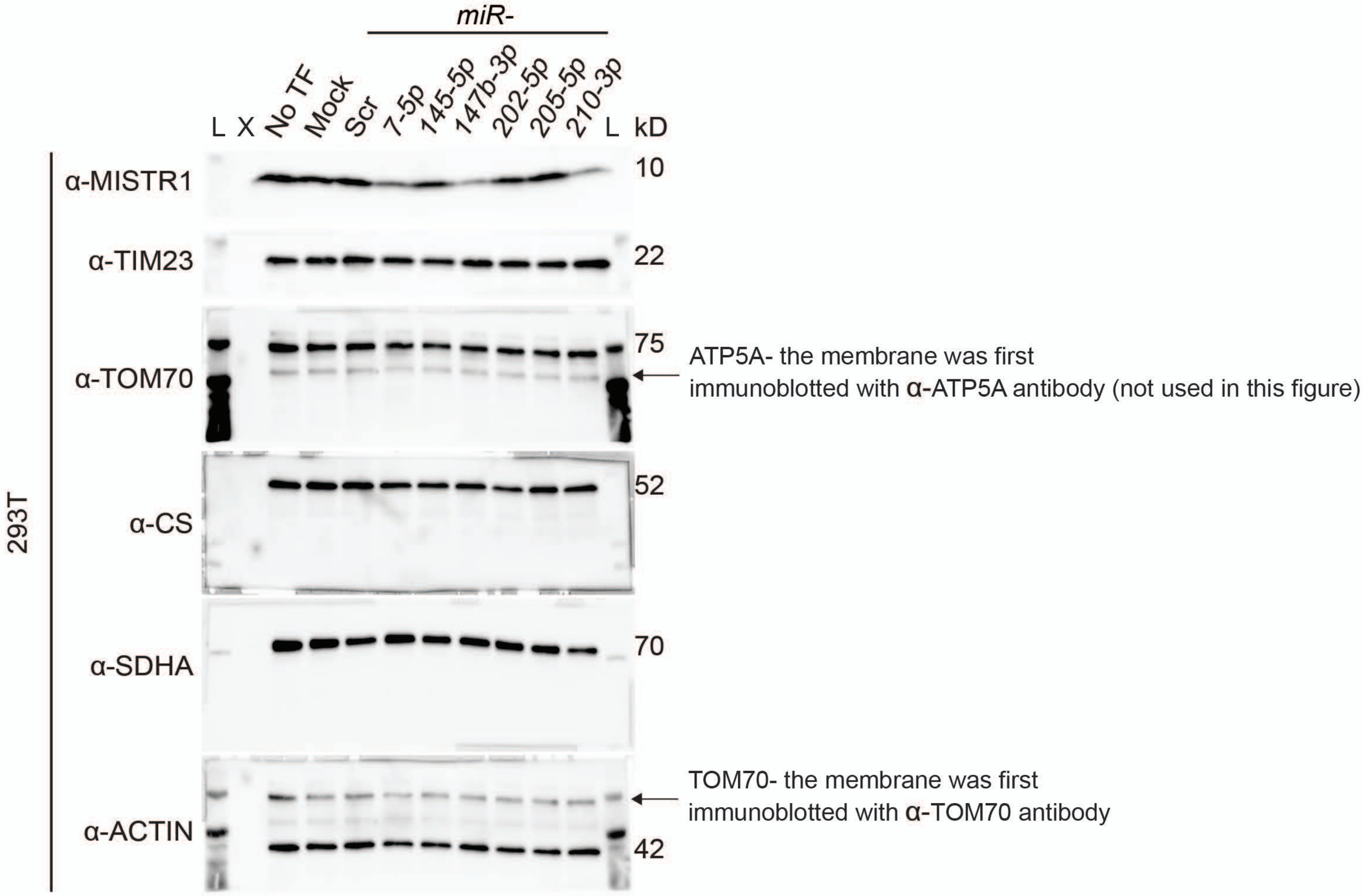

ATP5A- the membrane was first immunoblotted with α-ATP5A antibody (not used in this figure)

TOM70- the membrane was first immunoblotted with α-TOM70 antibody

Fig 6A

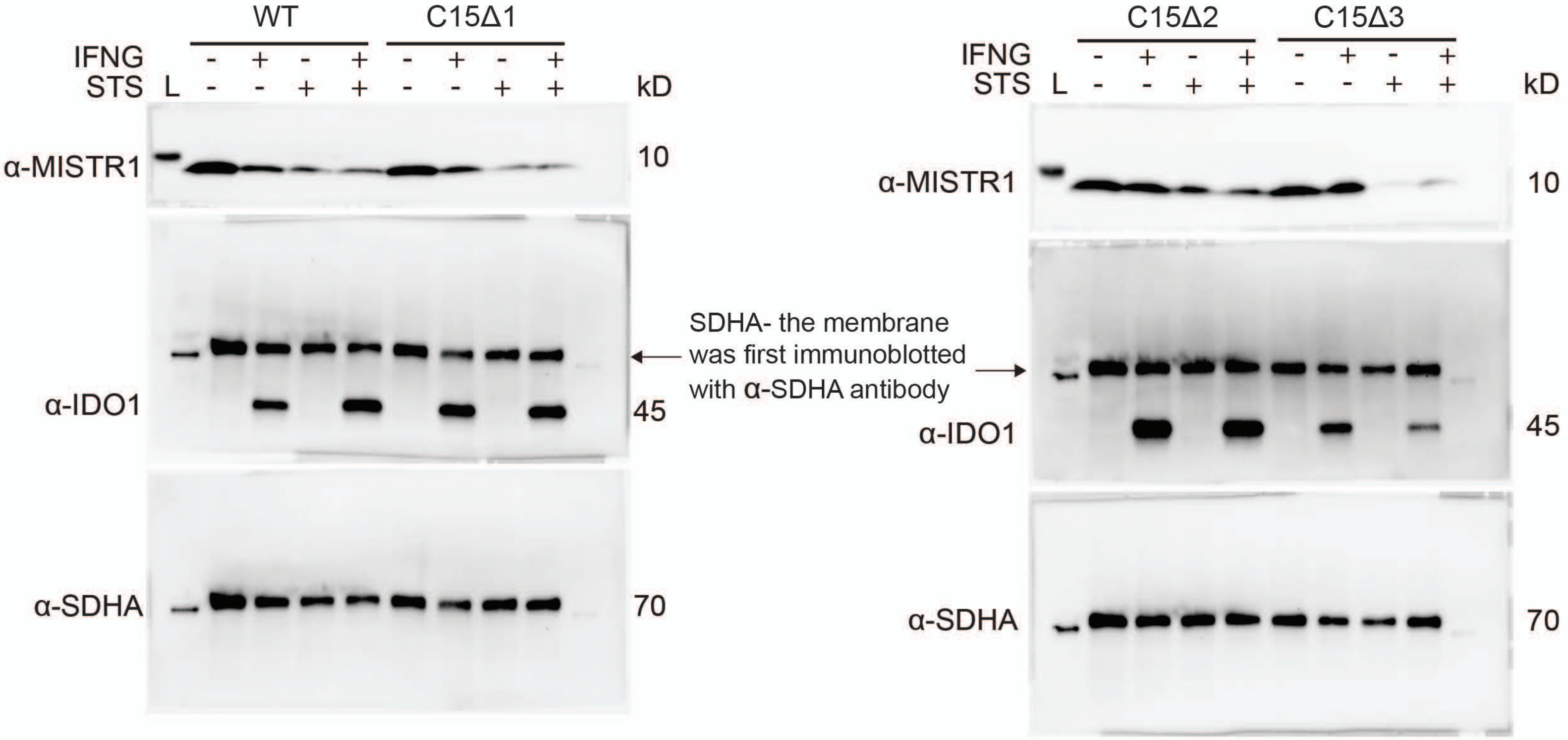

WT
C15Δ1
IFNG - + - + - + - +
STS L - - + + - - + +
kD
α-MISTR1
10
α-IDO1
45
α-SDHA
70
SDHA- the membrane was first immunoblotted with α-SDHA antibody
C15Δ2
C15Δ3
IFNG - + - + - + - +
STS L - - + + - - + +
kD
α-MISTR1
10
α-IDO1
45
α-SDHA
70

Fig 6B

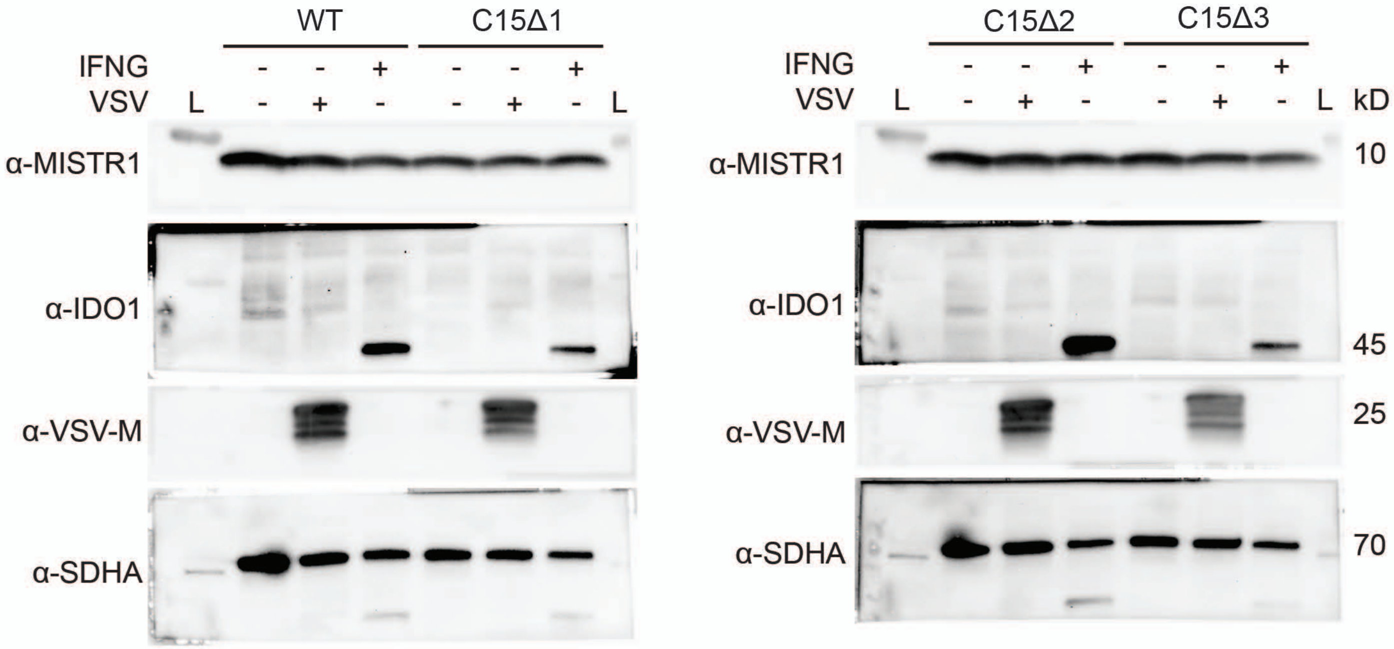

WT
C15Δ1
IFNG - - + - - +
VSV L - + - - + - L
α-MISTR1
α-IDO1
α-VSV-M
α-SDHA
C15Δ2
C15Δ3
IFNG - - + - - +
VSV L - + - - + - L
kD
α-MISTR1
10
α-IDO1
45
α-VSV-M
25
α-SDHA
70

Fig 6C

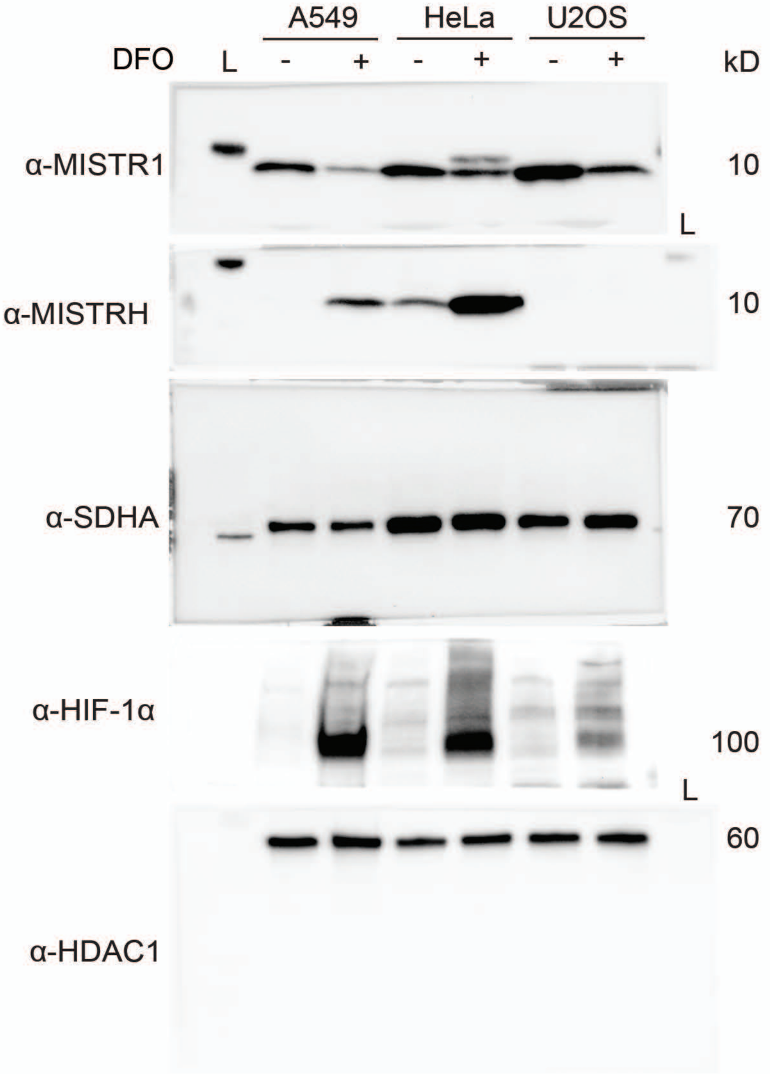
A549
HeLa
U2OS
DFO
L
-
+
-
+
-
+
kD
α-MISTR1
10
L
α-MISTRH
10
α-SDHA
70
α-HIF-1α
100
L
60
α-HDAC1

Fig 6D

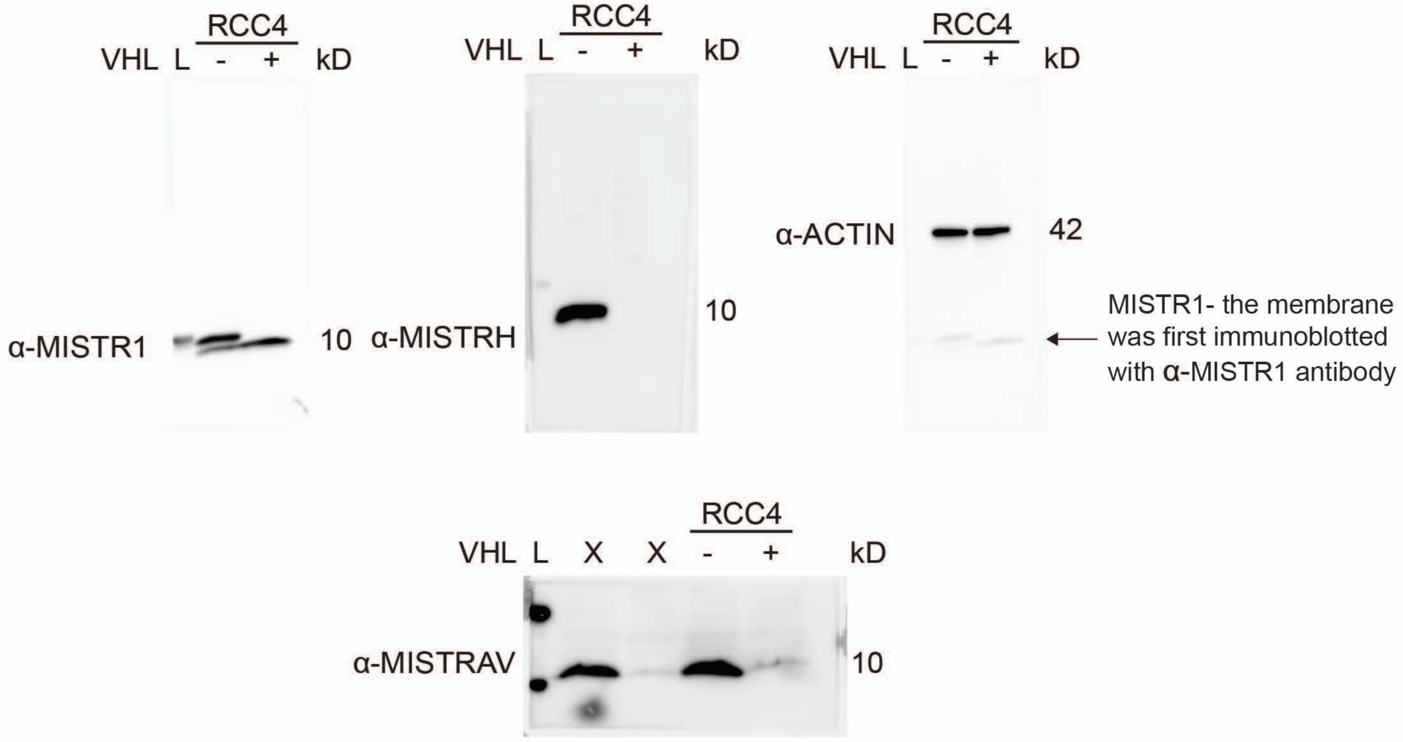
RCC4
VHL
L
-
+
kD
α-MISTR1
10
RCC4
VHL
L
-
+
kD
α-MISTRH
10
RCC4
VHL
L
-
+
kD
α-ACTIN
42
MISTR1- the membrane was first immunoblotted with α-MISTR1 antibody
RCC4
VHL
L
X
X
-
+
kD
α-MISTRAV
10

S5C Fig

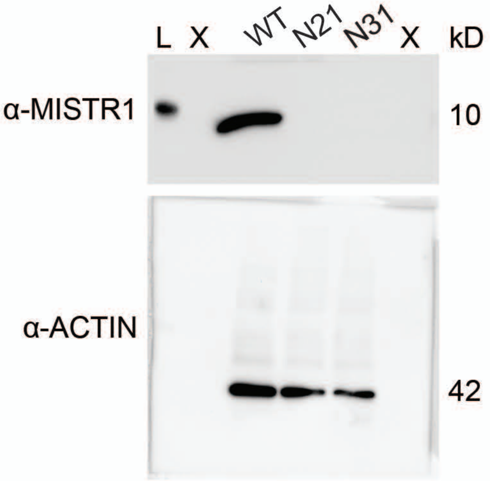

S5E Fig

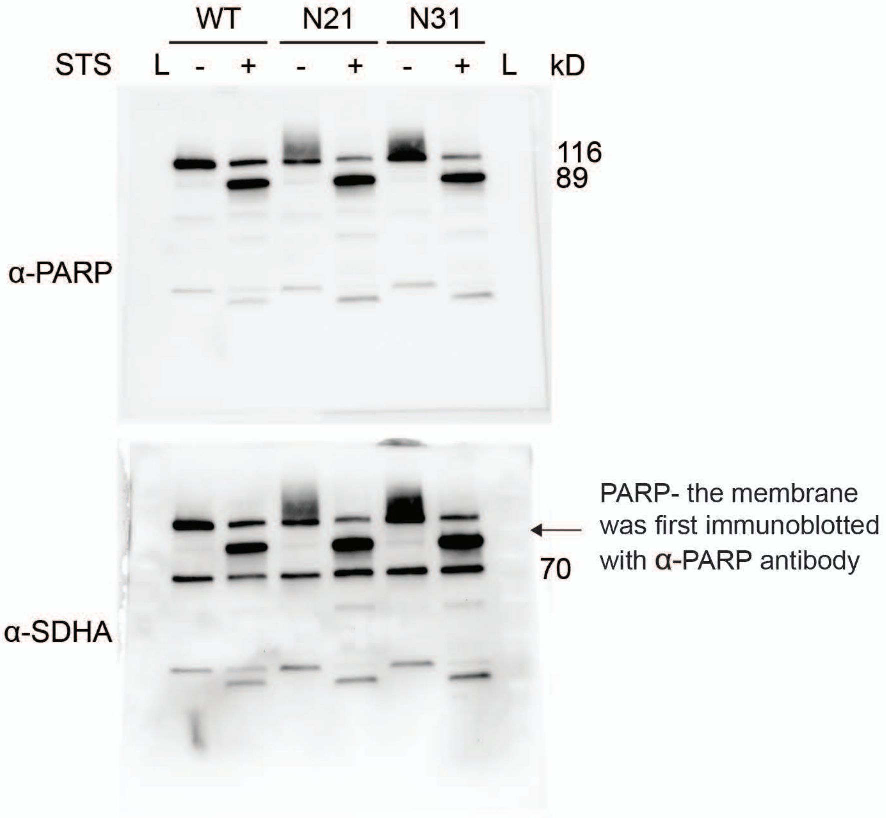

S5F Fig

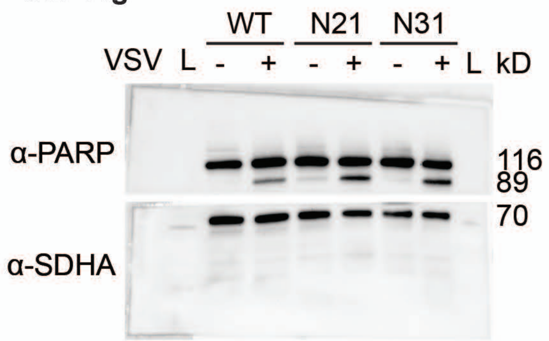

S8B Fig

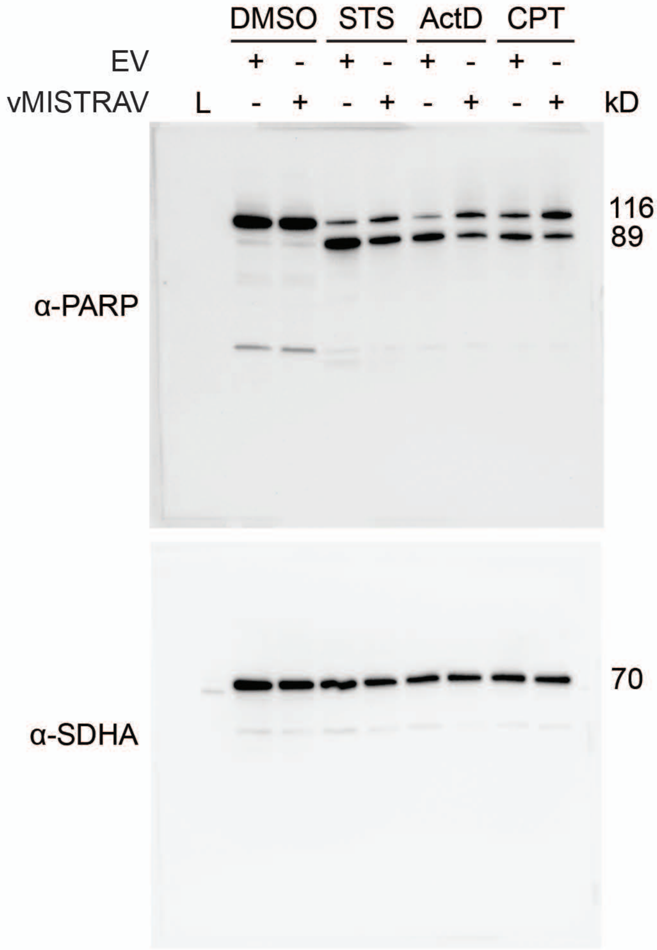

S8C Fig

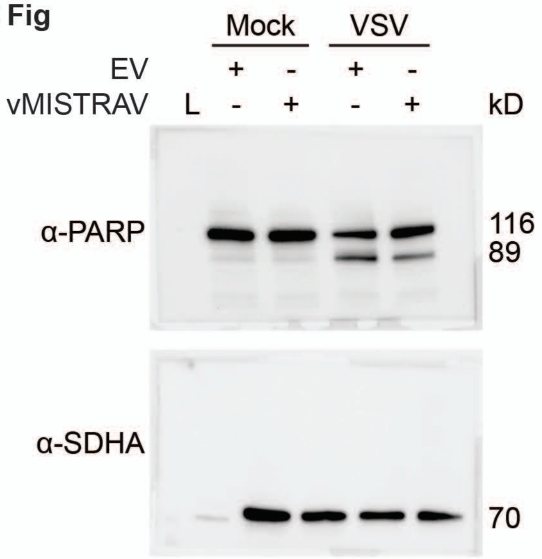

Uncropped Gel Images

Notes:
Gels were imaged using the ChemiDoc MP Imager (Bio-Rad).
“L” indicates molecular weight ladder.
“X” indicates a lane excluded from the final figure shown in the paper.
Relevant molecular weight ladder bands are labeled on the left side of each gel image.

Fig 1E

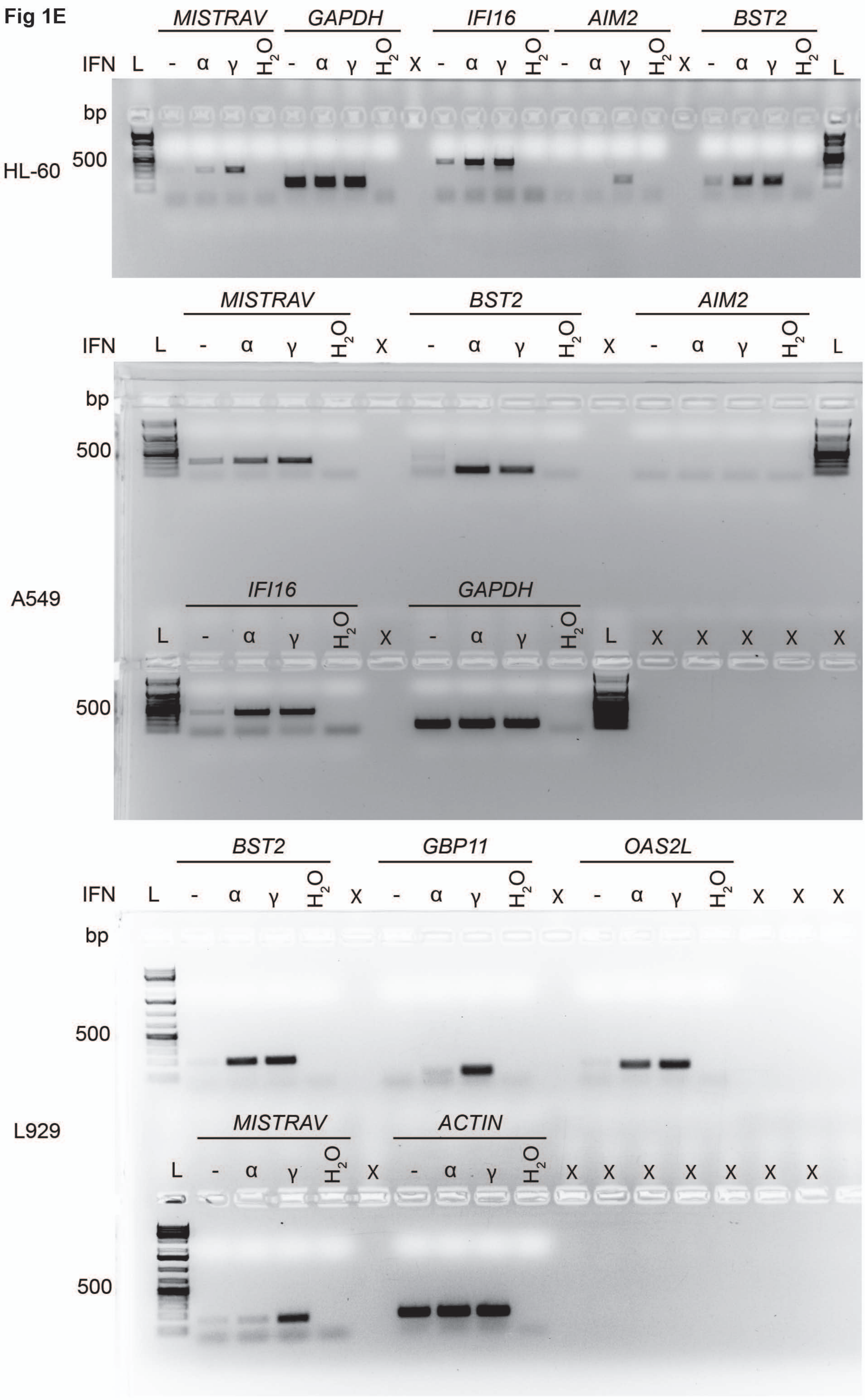

MISTRAV
GAPDH
IFI16
AIM2
BST2
IFN L - α γ H2O - α γ H2O X - α γ H2O - α γ H2O X - α γ H2O L
bp
500
HL-60
MISTRAV
BST2
AIM2
IFN L - α γ H2O X - α γ H2O X - α γ H2O L
bp
500
A549
IFI16
GAPDH
L - α γ H2O X - α γ H2O L X X X X X
500
BST2
GBP11
OAS2L
IFN L - α γ H2O X - α γ H2O X - α γ H2O X X X
bp
500
L929
MISTRAV
ACTIN
L - α γ H2O X - α γ H2O X X X X X X X
500

Fig 1E

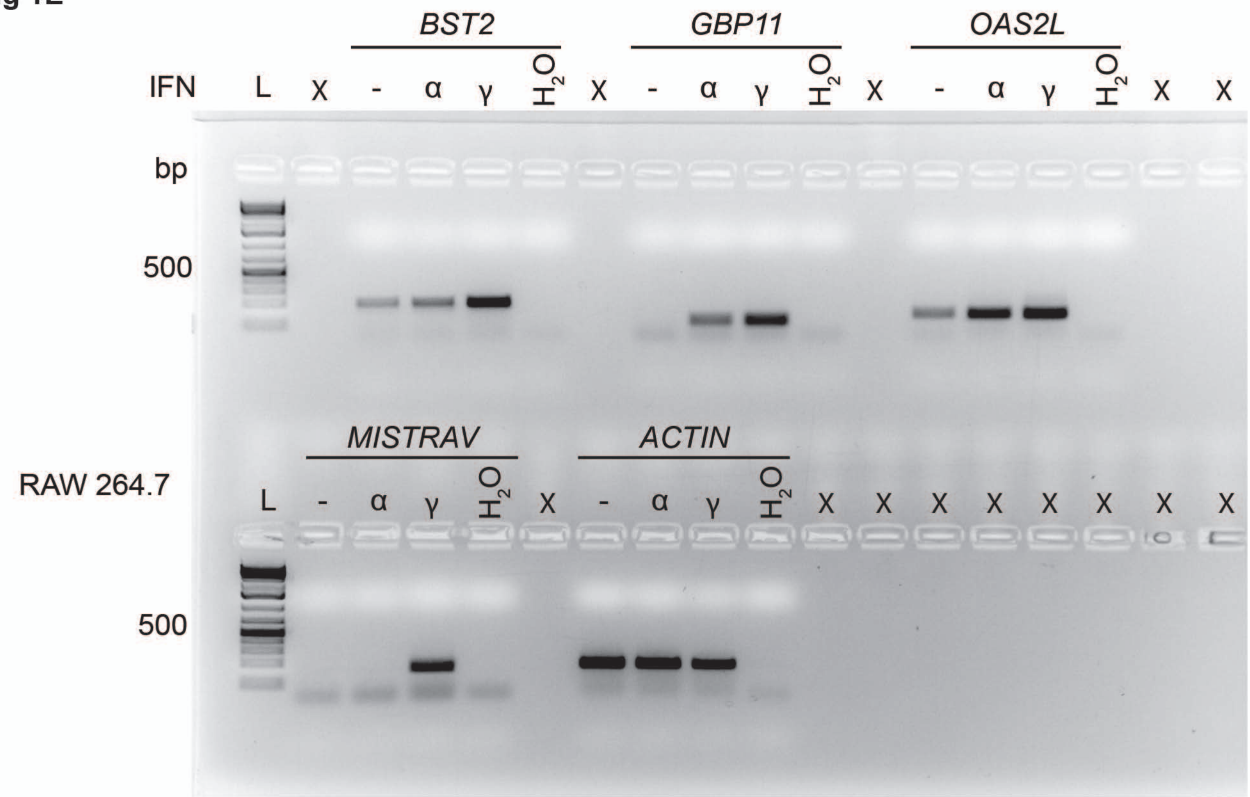

Fig 3C

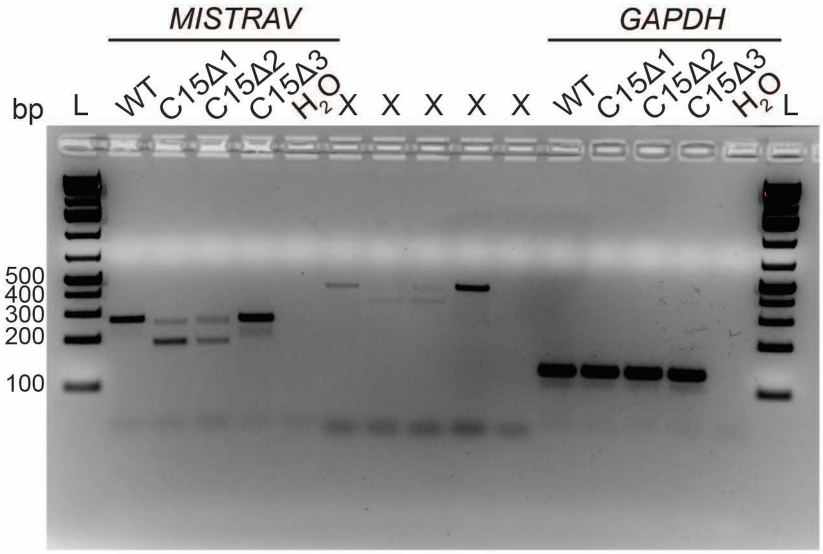

S5B Fig

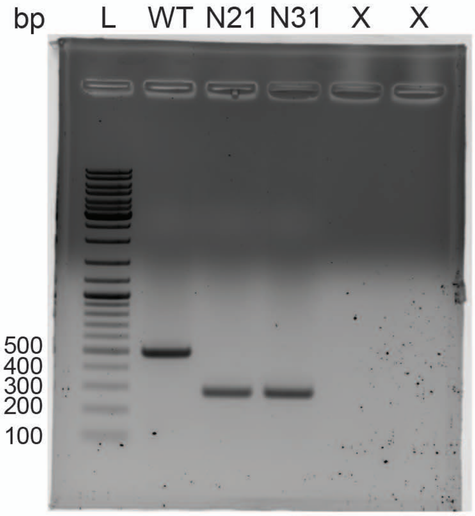

Supplement: S1 Raw Images — (PDF) [file pbio.3001045.s016.pdf]
